# Chromatin de-condensation by switching substrate elasticity

**DOI:** 10.1038/s41598-018-31023-2

**Published:** 2018-08-23

**Authors:** Morgane Rabineau, Florence Flick, Claire Ehlinger, Eric Mathieu, Isabelle Duluc, Matthieu Jung, Bernard Senger, Leyla Kocgozlu, Pierre Schaaf, Philippe Lavalle, Jean-Noël Freund, Youssef Haikel, Dominique Vautier

**Affiliations:** 1 Inserm UMR-S1121, 11 rue Humann, 67085 Strasbourg, France; 2 Université de Strasbourg, Faculté de Chirurgie Dentaire, 8 rue Sainte Elisabeth, 67000 Strasbourg, France; 3 Inserm UMR-S1113, 3 avenue Molière, 67200 Strasbourg, France; 4 Université de Strasbourg, IGBMC Microarray and Sequencing Platform, Illkirch, France; 5 Fédération de Médecine Translationnelle, Strasbourg, France

## Abstract

Mechanical properties of the cellular environment are known to influence cell fate. Chromatin de-condensation appears as an early event in cell reprogramming. Whereas the ratio of euchromatin *versus* heterochromatin can be increased chemically, we report herein for the first time that the ratio can also be increased by purely changing the mechanical properties of the microenvironment by successive 24 h-contact of the cells on a soft substrate alternated with relocation and growth for 7 days on a hard substrate. An initial contact with soft substrate caused massive SW480 cancer cell death by necrosis, whereas approximately 7% of the cells did survived exhibiting a high level of condensed chromatin (21% heterochromatin). However, four consecutive hard/soft cycles elicited a strong chromatin de-condensation (6% heterochromatin) correlating with an increase of cellular survival (approximately 90%). Furthermore, cell survival appeared to be reversible, indicative of an adaptive process rather than an irreversible gene mutation(s). This adaptation process is associated with modifications in gene expression patterns. A completely new approach for chromatin de-condensation, based only on mechanical properties of the microenvironment, without any drug mediation is presented.

## Introduction

Cancer cells are characterized by their proliferative potential, ability to metastasize and high degree of plasticity^1^. This process requires the loss of the molecular characteristics of healthy cells and the acquisition of a new molecular signature that is not necessarily accompanied by modifications in the genomic sequence^2,3^ and called “epigenetic reprogramming”. Recent data have demonstrated the key roles of nuclear organization, chromatin structure, chromatin dynamics and histone modifications in this fundamental process^4^. Nuclear organization refers to the positions adopted by specific regions of the genome. The open, active euchromatin, which is permissive for gene activation, occupies most of the nucleus, whereas the condensed, inactive heterochromatin is limited to an irregular edge located at the nuclear periphery and around the nucleolus, as well as in patches scattered in the nucleoplasm^5^. Recent reports have recognized that chromatin remodelling towards an open chromatin structure as an early event in cell reprogramming^6^. Inhibitors of histone deacetylase and DNA methyltransferases have been identified as major routes for chromatin de-condensation^7^.

Increasing evidences supports the central role of the mechanical properties of the cellular microenvironment in cell fate^8,9^ and in nuclear activity^10^. Indeed, the Young’s modulus of the cellular microenvironment affects the chromatin organization in healthy cells, such that a soft matrix favours chromatin condensation^11,12^. This phenomenon implies that mechanical signals are transmitted across the cytoskeleton to the nucleus^13^ and ultimately propagate to chromatin, which represents a site of signal integration and interpretation for gene expression^4^.

**Figure 1:**
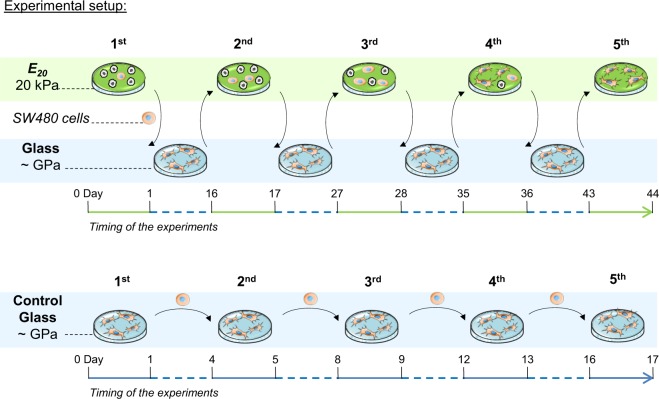
Conditions and timing of the experiments.

In colon cancer, only a small fraction of cancer cells survives the shift from a relatively rigid microenvironment, sustained by the basement membrane, to the liquid lymph and blood, and to adhere again to the stiff tissue at the metastatic site (for example, 175, 918, 320, 120 and 640 Pa for basement membrane, stroma, lymph, lymph node and liver, respectively)^14^. A soft microenvironment seems to be a key parameter in the acquisition of invading properties^15–22^. We have previously shown that reducing the rigidity of an adhesion substrate leads to massive death of human SW480 colon cancer cells. However, some of these cancer cells retain the capacity to survive on soft matrices^23^. Increasing evidence supports that chromatin compaction acts as an early step in tumourigenesis, inducing the downregulation of tumour suppressor genes and activation of pro-oncogenes involved in neoplastic progression^24,25^. However, it remains unknown whether cancer cell survival relies on changes in chromatin organization, such as compaction or opening. This question has never been addressed experimentally and is the purpose of the present study. This possible relationship may provide insight into malignant transformation. For this purpose, we assayed the behaviour of SW480 cancer cells on polyelectrolyte multilayer films with an elastic modulus of 20 kPa (short-hand notation *E*_*20*_) as a selective soft substrate model for cell survival. To highlight the survival process developed by the cancer cells, cells that were resistant on *E*_*20*_ were recovered and amplified on supraphysiologically stiff culture glass slides and replated on *E*_*20*_ substrate (Fig. 1). This process was used as a model of changes in the physical environment faced by cancer cells during malignant cell dissemination. Here, we show that the initial 24 h-contact with *E*_*20*_ leads to a low rate of survival of SW480 cancer cells and that 4 consecutives glass *- E*_*20*_ cycles increase both cellular survival and cellular motility in correlation with the induction of chromatin de-condensation. This work represents a new step towards the understanding of how cancer cells resist mechanical stresses and provides a new approach for chromatin de-condensation without any drug mediation as it is based solely on mechanical cues.

## Increase in cell survival by switching the substrate elasticity

To address whether cell survival on soft matrices relies on a particular chromatin conformation, we first examined the viability of SW480 cancer cells on the soft substrate model *E*_*20*_. This substrate was composed of a 24 bilayers of poly(L-lysine) alternatively deposited with a hyaluronic acid ((PLL/HA)_24_) stratum capped with a second stratum made of poly(sodium styrene sulfonate)/poly(allylamine hydrochloride) (PSS/PAH). The PLL/HA films are known to be non-adhesive to cells^26^, whereas PSS/PAH presents remarkable properties with respect to cell adhesion and cellular proliferation^27^. However, we can point out a limitation of the soft substrate as its Young modulus of 20 kPa is high compared with that of *in vivo* colon tissues (from 175 to 918 Pa^14^). It should be noted that the disparity between these two environments concerns also their dimensionality as well as their chemical compositions. Indeed, in colon, cells are in a specialized 3D tissue with cell-matrix and cell-cell anchoring not reproduced in our 2D *in vitro* substrate. Yet, our system allows non-specific adhesion unlike *in vivo* tissue that contains adhesive ligands targeting specific adhesions. Nevertheless, the main property of our system is to be selective for cell survival. This selection process can be achieved using cells cultured alternatively on our soft and rigid substrates.

**Figure 2:**
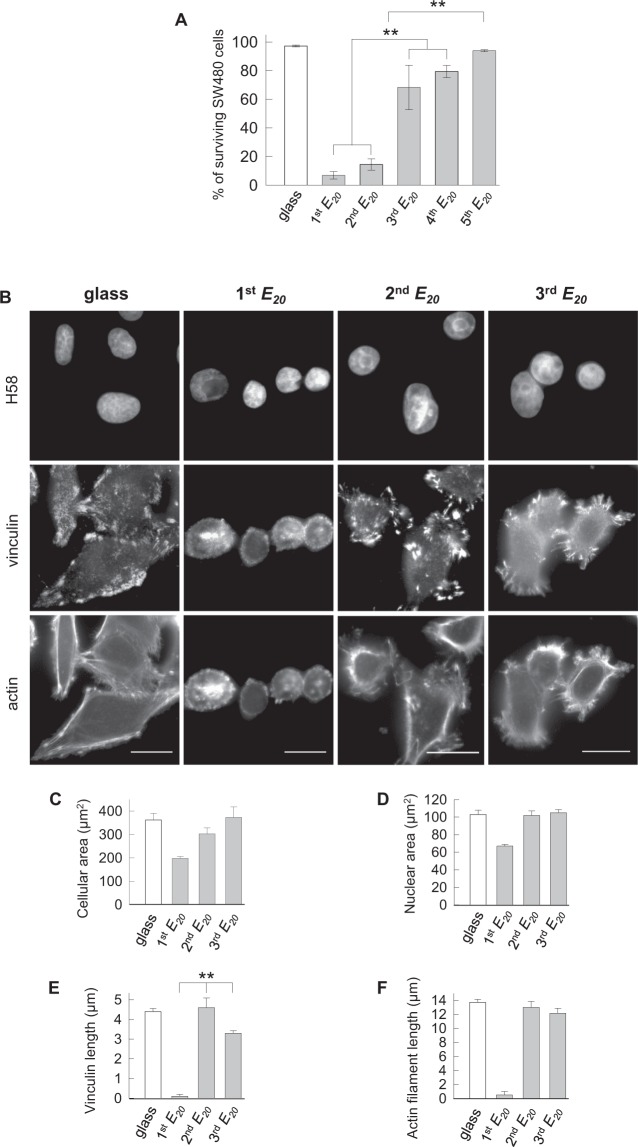
Cell survival and vinculin assembly are influenced by switching the substrate elasticity. **(A)** Percentage of surviving SW480 cells based on the S1 from three pooled independent experiments (error bars represent s.e.m.). **(B)** Representative images of cells cultured for 24 h on glass, 1^st^ *E*_*20*_, 2^nd^ *E*_*20*_ and 3^rd^ *E*_*20*_ immunolabelled with anti-vinculin counterstained with Hoechst 33258 and phalloidin. Scale bars: 10 µm. **(C)** Quantitative data for cell areas (in µm^2^), based on B, were determined using ImageJ. We used 200–300 cells for each condition (glass, 1^st^ *E*_*20*_, 2^nd^ *E*_*20*_ and 3^rd^ *E*_*20*_). **(D)** Quantitative data for nuclear areas (in µm^2^) based on B were determined using ImageJ. We used 200–300 cells for each condition. **(E)** Quantification of vinculin spots (in µm) based on B by measuring 20 contacts per cell in 20 cells for each condition. **(F)** Quantification of actin stress fibers (in µm) using ImageJ based on B by measuring the length of 20 fibers per cell in 20 cells for each condition. **(C–F)** Results for three independent experiments are shown (error bars represent s.e.m.).

In control experiments, fluorescent apoptotic-necrotic-healthy assays showed that 97% of the cells were alive when repeatedly (at least 5 times, 5^th^ glass) seeded on glass (Figs 2A and S1). Conversely, when SW480 cells were seeded for 24 h for the 1^st^ round on *E*_*20*_, the live/dead assay revealed that 93% of the cells were positive for ethidium homodimer III, indicative of massive death by necrosis, whereas only 7% of the cells survived (Fig. 2A). These cells adopted a round-shaped morphology (Fig. 2B) and failed to exhibit vinculin adhesion spots and F-actin stress fibers (Fig. 2B,E,F). We wondered whether the cells that were resistant to death retained their capacity to spread and divide when transferred back to a stiff substrate. Thus, surviving cells were isolated by trypsinization and seeded on a glass surface. The cells were able to adhere and proliferate to reach confluence after 15 days of culture while showing round or spread-shaped morphologies (Fig. S2). Following this 1^st^ *E*_*20*_ - glass sequence, SW480 cells were relocated for a second round to *E*_*20*_ (2^nd^ *E*_*20*_). Under this experimental condition, 14% of the cells survived displaying round or spread-shaped morphologies (Fig. 2B), and their cell area remained greater than on the 1^st^ *E*_*20*_ (Fig. 2C). The spread cells exhibited vinculin spots of 4.6 µm in length localized at the tip of actin microfilaments (Fig. 2B,E). Next, the SW480 cells that survived on the 2^nd^ *E*_*20*_ were transferred to glass for 10 days of amplification and cultured again for 24 h on the 3^rd^ *E*_*20*_. The percentage of surviving cells on the 3^rd^ *E*_*20*_ increased to 68% (Figs 2A and S1), and the vinculin spots at the protrusive extremities were 1.4-fold smaller than those visualized on the 2^nd^ *E*_*20*_ (3.3 µm vs 4.6 µm) (Fig. 2B,E). The cells surviving on the 3^rd^ *E*_*20*_ were seeded back on glass for 7 days, followed by a 4^th^ round of 24 h on *E*_*20*_. The surviving cells were again subjected to a 5^th^ cycle of glass-*E*_*20*_. The percentage of cells that survived the fourth seeding on *E*_*20*_ increased to 79% and finally reached 94% after the fifth seeding on *E*_*20*_, identical to the control cells continuously seeded on the glass surface (Figs 2A and S1).

## Increase in cell motility by switching substrate elasticity

**Figure 3:**
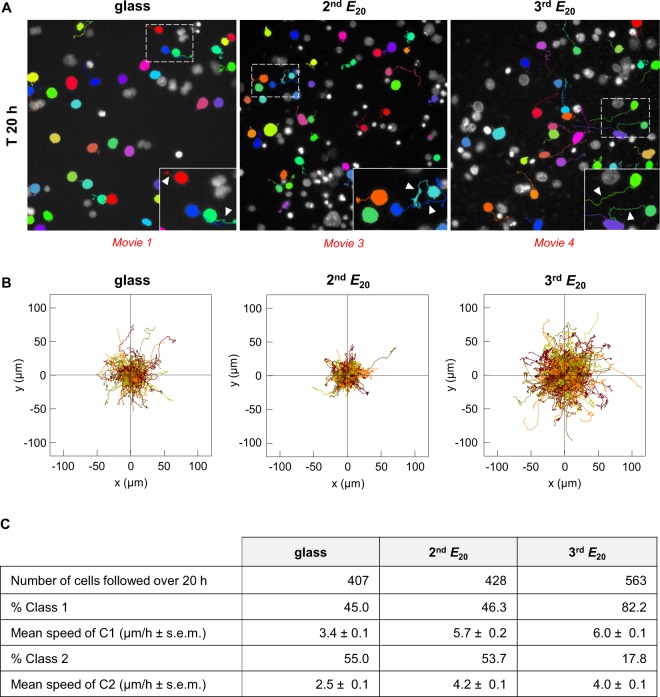
Cell motility influenced by switching the substrate elasticity. Motility of SW480 cells after successive seeding on soft substrate. **(A)** Time-lapse monitoring representative images of cells cultured on glass, 2^nd^ *E*_*20*_ and 3^rd^ *E*_*20*_ for 20 h. Inset: enlargement of the white dotted panel. Arrowhead: coloured cellular trajectory. **(B)** Cell trajectories from A by NIS-Elements 3D tracking. **(C)** Number of cells analysed, percentage of cells in class 1 and class 2, and mean speed of these cells, from A. The results from two pooled independent experiments for each condition.

We examined the behaviour of SW480 cells after successive relocation on soft and rigid matrices by testing their motility, a major feature of aggressive cancer cells. The motility of SW480 cancer cells was first analysed by live imaging over a period of 20 h on the control rigid surface (3^rd^ glass substrate). For this purpose, we defined 2 classes of cells: class 1 represented cells that exited at least once from the radius of the circle corresponding to the mean dimensions of the nuclei (that is 11.4 μm calculated from a series of 100 cells in several experiments on 2^nd^ *E*_*20*_ and 3^rd^ *E*_*20*_); class 2 represented cells which that did not exit from the circle (Fig. S3). The results are illustrated in Fig. 3A,B and Movies 1–4, and quantification of the results is shown in Fig. 2C. On glass substrate, video microscopy showed that 45% of the cells adopted the behaviour of the class 1 because they exited at least once from the virtual circle with a mean velocity of 3.4 µm/h, whereas 55% of the cells did not exit from the circle and therefore belonged to the class 2 with a mean velocity of 2.5 µm/h. As expected from the above results, on the 1^st^ *E*_*20*_, the number of living cells were observed by video microscopy was very small. Indeed, cancer cells massively died during the initial hours through a lytic process characterized by the progressive release of cytoplasmic and nuclear material in the culture medium (see Movie 2–Fig. S5). Consequently, motility parameters were not analysed at this stage. On the 2^nd^ *E*_*20*_, live cell imaging (Fig. 3A–C, Movie 3) showed that the percentages of cells in both classes 1 and 2 (46 and 53% for class 1 and class 2, respectively) were similar to those on the glass substrate (45 and 55% for class 1 and 2, respectively), whereas the mean velocity was 2.3 times higher for the class 1 (5.7 vs 3.4 µm/h on 2^nd^ *E*_*20*_ and glass, respectively) and 1.7 times higher for class 2 (4.2 vs 2.5 µm/h on 2^nd^ *E*_*20*_ and glass, respectively). Moreover, on the 2^nd^ *E*_*20*_, interphase cells were able to alter their morphology several times from a round to spread shape with filopodial extensions and retracting protrusions (Fig. 3A, Movie 3). These changes in cellular morphologies seemed to be used by the cells for movement. Of note, this behaviour contrasted with that of the cells on glass, which, once spread, do no longer changed their morphology (Fig. 3A, Movie 1). As the rates of cell survival on the 3^rd^ and 4^th^ *E*_*20*_ were similar (Fig. 3A), cell motility was tested only on the 3^rd^ *E*_*20*_. In this condition, the percentage of class 1 cells increased compared with that for glass (82 vs 45%, respectively) and that for the 2^nd^ *E*_*20*_ substrate (82 vs 46%, respectively), although their mean velocity remained unchanged compared with that for class 1 cells on the 2^nd^ *E*_*20*_ (6 vs 5.7 µm/h). As in the 2^nd^ *E*_*20*_, interphase cells on the 3^rd^ *E*_*20*_ were able to change several times from round to spread morphologies with filopodial extensions and retracting protrusions (Fig. 3 Movie 3). On the 2^nd^ *E*_*20*_ the reduced percentage of motile cells was associated with cells exhibiting large spots of vinculin. Conversely, on the 3^rd^ *E*_*20*,_ the higher percentage of motile cells corresponded to cells exhibiting smaller vinculin spots. In accordance, it has been previously reported that adhesion strength modulates migration speed by spatiotemporal feedback between actomyosin and focal-adhesion^28^ and that enlarged adhesions are accompanied by reduced cancer cells migration^29^. Therefore, the successive relocation on soft matrices led to the progressive increase in cell velocity and motility.

## Chromatin de-condensation by switching substrate elasticity

**Figure 4:**
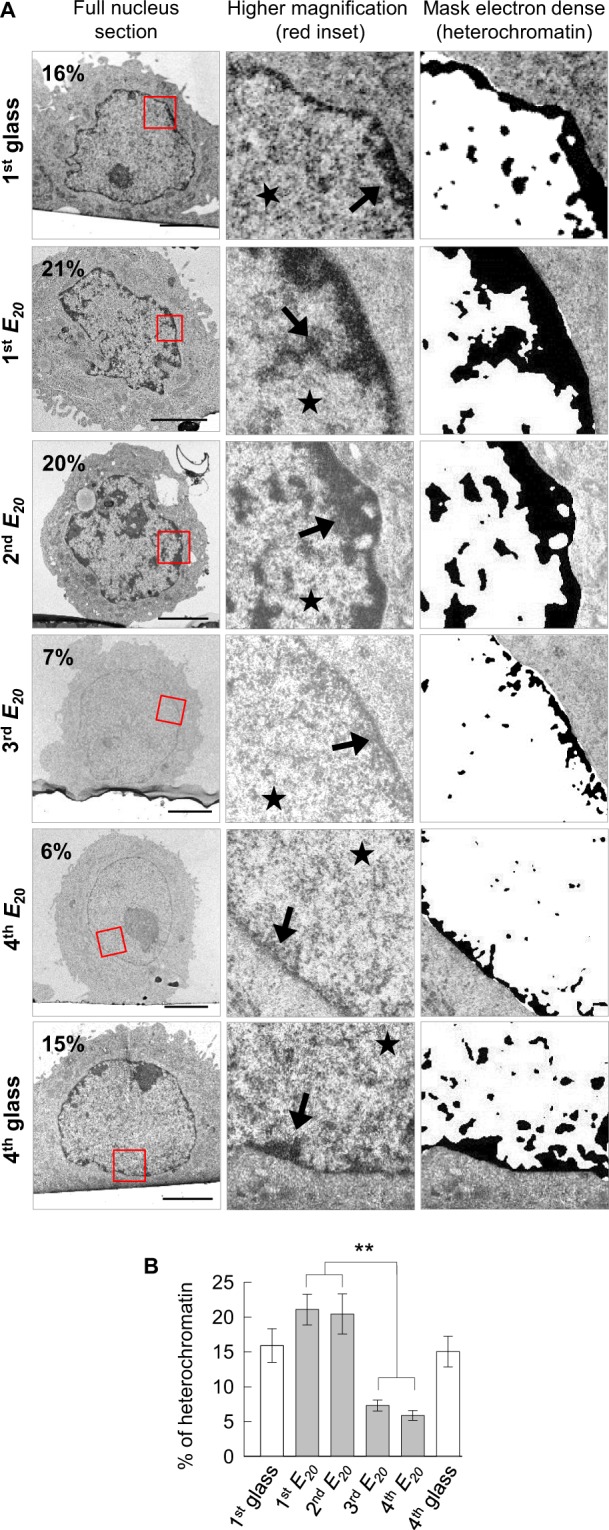
Chromatin de-condensation by switching the substrate elasticity **(A)** Representative ultrastructural images of cells after 24 h of culture on 1^st^ glass, 1^st^ *E*_*20*_, 2^nd^ *E*_*20*_, 3^rd^ *E*_*20*_, 4^th^ *E*_*20*_, and 4^th^ glass. Scale bars: 5 µm, arrow: heterochromatin, star: euchromatin, **(B)** Percentage of heterochromatin in the nucleus of approximately 20 cells for each condition, from A, for two pooled independent experiments (error bars represents s.e.m.).

We next explored whether these cellular behaviours relied on a particular chromatin conformation. To address this issue, the nuclei of SW480 cells undergoing successive glass *-E*_*20*_ cycles were observed by transmission electron microscopy (TEM) (Fig. 4A,B). On control glass substrate (1^st^ glass and 4^th^ glass substrates), SW480 cells displayed euchromatin uniformly distributed within the nucleus, with a thin layer of heterochromatin at the nuclear periphery. Quantification indicated a heterochromatin content of 16% and 15% of the nuclear chromatin, for respectively, 1^st^ and 4^th^ glass conditions. On the 1^st^ *E*_*20*_, surviving cells showed an increased content of heterochromatin, which rose to 21%, localized at the nuclear periphery, as well as discrete nuclear patches. These heterochromatin features are well-known manifestations of colorectal cancer cells, in which heterochromatin localization shows a loss of its typical peripheral association with the nuclear envelope and an increase in chromatin compaction^24,25,30,31^. A similar chromatin pattern was displayed by cells on the 2^nd^ *E*_*20*_, with heterochromatin either at the nuclear periphery or within discrete nuclear patches representing 20% of the total chromatin content. In clear contrast to these results, TEM analysis of cells on the 3^rd^ *E*_*20*_ showed no discrete patches of heterochromatin throughout the nucleus and no obvious enrichment of heterochromatin at the nuclear periphery. Consequently, the percentage of heterochromatin dropped to approximately 7%. Like to the 3^rd^ *E*_*20*_, cells on the 4^th^ *E*_*20*_ were characterized by the absence of discrete patches of heterochromatin and a very thin layer of heterochromatin at the nuclear periphery representing 6% of the total chromatin.

These results underline a remarkable reorganization of the cancer cell chromatin with a high proportion of heterochromatin after the 1^st^ and 2^nd^ relocation on *E*_*20*_ and a strong chromatin de-condensation after the 3^rd^ and 4^th^ relocation on *E*_*20*_.

## Survival and motility are chromatin conformation-dependent

We further explored the hypothesis that increased of cell survival and motility on soft substrates might be linked to cell chromatin de-condensation. For this purpose, we artificially de-condensed the chromatin of cells on the 1^st^ and the 2^nd^ relocation on *E*_*20*_. Thus, the cells were treated with trichostatin A (TSA), a drug that inhibits histone deacetylases, during the 1^st^ or 2^nd^ steps on *E*_*20*_ with the aim of maintaining chromatin in the euchromatin form.

**Figure 5:**
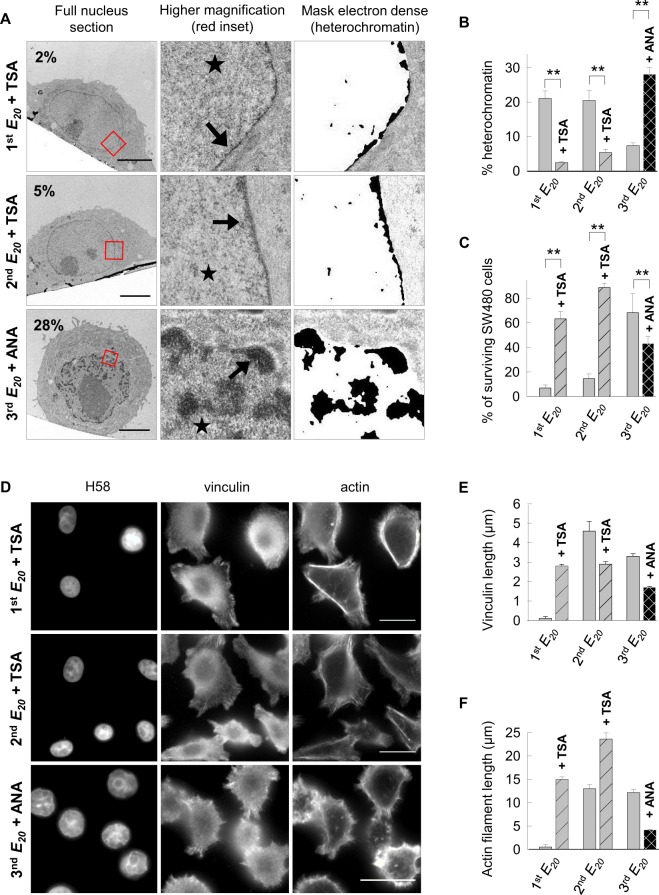
Cell survival is chromatin conformation-dependent **(A)** Representative ultrastructural images of cells after 24 h of culture on the 1^st^ *E*_*20*_ + TSA, 2^nd^ *E*_*20*_ + TSA and 3^rd^ *E*_*20*_ + ANA. Scale bars: 5 µm, arrow: heterochromatin, star: euchromatin. **(B)** Percentage of heterochromatin in the nucleus of approximately 20 cells for each condition, from A, for two pooled independent experiments (error bars represents s.e.m.). **(C)** Percentage of surviving SW480 cells from S4 for two or three pooled independent experiments (error bars represent s.e.m.). **(D)** Representative images of cells cultured for 24 h on 1^st^ *E*_*20*_ + TSA, 2^nd^ *E*_*20*_ + TSA and 3^rd^ *E*_*20*_ + ANA immunolabelled with anti-vinculin andcounterstained with Hoechst 33258 and phalloidin. Scale bars: 10 µm. **(E)** Quantification of vinculin spots (in µm) based on D by measuring 20 contacts per cell in 20 cells for each condition. (**F**) Quantification of actin stress (in µm) using ImageJ based on D by measuring the length of 20 actin stress fibers per cell in 20 cells for each condition. **(D–F)** Results for three independent experiments (error bars represent s.e.m.).

TEM analysis of cells on the 1^st^ *E*_*20*_ + TSA and 2^nd^ *E*_*20*_ + TSA revealed a strong reduction in compacted heterochromatin at the nuclear envelope or in nucleoplasmic patches compared with that for the corresponding untreated cells (1^st^ *E*_*20*_ + TSA 2.4% vs 1^st^ *E*_*20*_ 21%, and 2^nd^ *E*_*20*_ + TSA 5% vs 2^nd^ *E*_*20*_ 20%) (Fig. 5A,B). At the ultrastructural level, the de-condensed chromatin induced by mechanical means appeared to be very similar to those obtained chemically by TSA and at equivalent rates (21 to 6% heterochromatin by mechanics and 5% by TSA) and similarly to the molecule CYT296^32^.

Furthermore, on the 3^rd^ relocation on *E*_*20*_, to maintain chromatin in the heterochromatin form, we artificially condensed the chromatin of cells with anacardic acid (ANA) and investigated the resulting consequences on cell survival and motility. ANA is an inhibitor of p300 and p300/CBP-associated factor histone acetyltransferases^33^.

Cells on the 3^rd^ *E*_*20*_ + ANA exhibited a higher proportion of heterochromatin than that for untreated cells on the 3^rd^ *E*_*20*_ (28 vs 7%) (Fig. 5A,B). Consistently, immunofluorescence analyses of cells using antibodies specific for nuclear acetylated histone H3K14 on the 3^rd^ *E*_*20*_ + ANA showed poor staining with these antibodies compared with that of the untreated cells, revealing a low level of Lys14 acetylation of histone H3 (Fig. S4A,B). These observations attested to the efficacy of the treatments with ANA. Next, we addressed the distribution of vinculin and actin proteins by immunofluorescence (Fig. 5D–F). On the 1^st^ *E*_*20*_ + TSA, the treatment prevented the loss of vinculin spots (2.8 µm in length) and actin fibers (15 µm), in contrast to the untreated cells on the 1^st^ *E*_*20*_. Upon TSA treatment on the 2^nd^ *E*_*20*_, the cell area increased 2 times compared with that of the untreated cells on the 2^nd^ *E*_*20*_ (2^nd^ *E*_*20*_ + TSA 630µm^2^ vs 2^nd^ *E*_*20*_ 303µm^2^). Of note, on the 2^nd^ *E*_*20*_ + TSA, the vinculin spots decreased 2 times compared with those on the 2^nd^ *E*_*20*_ (2.9 vs 4.6 µm). Treatment of cells seeded on the 3^rd^ *E*_*20*_ with the HAT inhibitor ANA led to a decrease in length of the vinculin spots (1.7 vs 4.4 µm) and actin length (4.2 vs 13.7 µm), respectively, by 2, 2.5 and 3 times compared with that for the untreated cells on the 3^rd^ *E*_*20*_ (Fig. 5A,B,D,E). To strengthen these observations, we investigated the consequences of these treatments on cell survival and cell motility. Fluorescent apoptotic-necrotic-healthy assays showed that treatment with TSA compared with untreated cells increased the rate of surviving cells on the 1^st^ *E*_*20*_ and 2^nd^ *E*_*20*_ (1^st^ *E*_*20*_ + TSA 63% vs 1^st^ *E*_*20*_ 6% and 2^nd^ *E*_*20*_ + TSA 88% vs 2^nd^ *E*_*20*_ 14%). Conversely, the rate dropped from 68 to 43% on the 3^rd^ *E*_*20*_ treated with ANA compared with that for the untreated cells (Figs 5C and S4C). These results indicate that cell death induced by seeding on soft substrate and the progressive survival resulting from consecutive passages on the soft substrate are functionally related to the changes in the heterochromatin/euchromatin balance imposed by the culture conditions.

**Figure 6:**
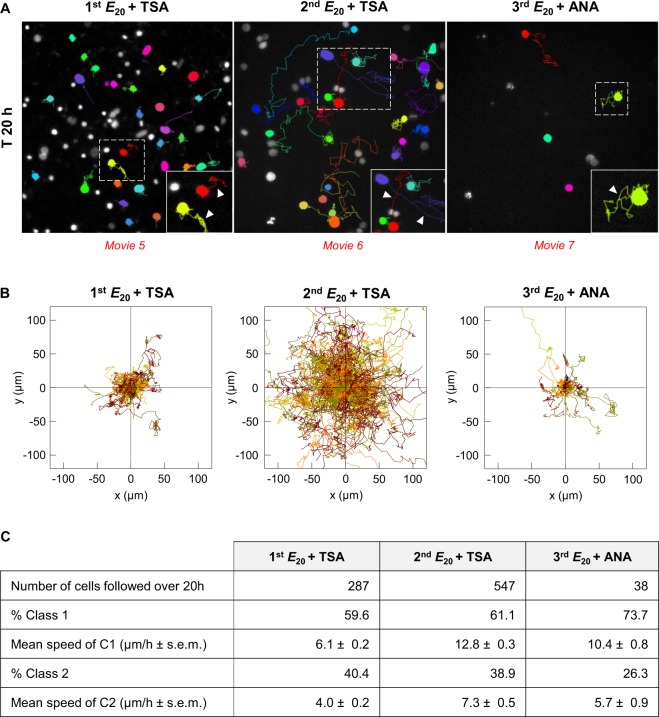
Cell motility is chromatin conformation-dependent. **(A)** Time-lapse monitoring representative images of cells cultured on 1^st^ *E*_*20*_ + TSA, 2^nd^ *E*_*20*_ + TSA and 3^rd^ *E*_*20*_ + ANA for 20 h. Inset: enlargement of the white dotted panel, arrowhead: coloured cellular trajectory. **(B)** Cell trajectories, from A, by NIS-Elements 3D tracking. **(C)** Number of cells analysed, percentage of cells in class 1 and class 2 and mean speed of these cells, from A. The Results from two pooled independent experiments for each condition. Class 1 speed of 1^st^ *E*_*20*_ + TSA vs 2^nd^ *E*_*20*_ + TSA vs 3^rd^ *E*_*20*_ + ANA: **.

Based on these results, we investigated whether the mobility of cells on soft matrices is functionally related to the chromatin conformation by performing time-lapse experiments (Fig. 6A–C and Movies 5–7). Incubation of the cells on the 1^st^ *E*_*20*_ with TSA showed 59% of motile cells in class 1 with a mean velocity of 6.1 µm/h. Movie 5 revealed that on the 1^st^ *E*_*20*_ + TSA, interphase cells were able to alter their morphology several times from a round to spread shape. On the 2^nd^ *E*_*20*_ + TSA, the number of motile cells increased by 1.3 and their mean velocity by 2 times (12.8 vs 5.7 µm/h) compared with that for cells on the 2^nd^ *E*_*20*_ without TSA. Cells on the 2^nd^ *E*_*20*_ + TSA were able to alternate round and spread morphologies like the untreated cells on the 2^nd^ *E*_*20*_ (Movie 6). In complementary experiments performed using cells on the 3^rd^ *E*_*20*_ treated with the HAT inhibitor ANA, the number of motile cells of class 1 slightly decreased compared with that for the 3^rd^ *E*_*20*_ (73 vs 82%), while the cells were still able to alter their morphology (Movie 7).

## Reversible adaptation of chromatin de-condensation

**Figure 7:**
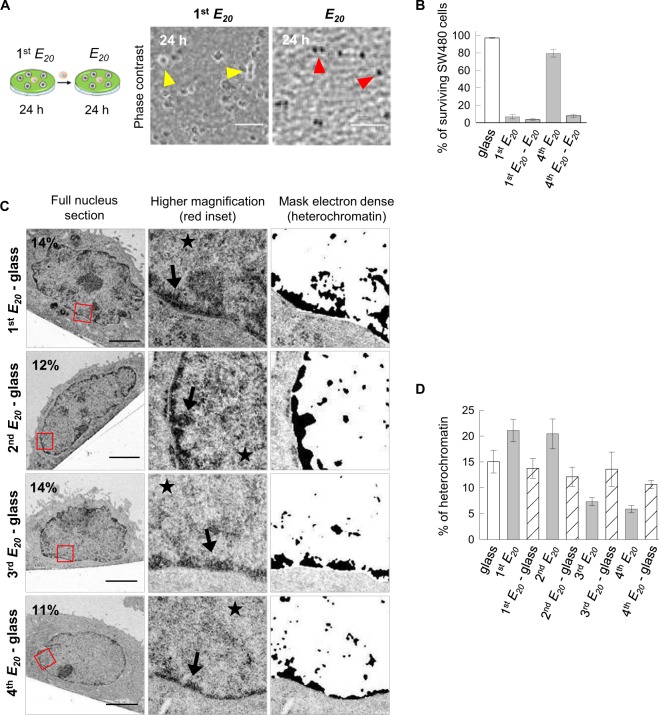
Reversible adaptation of chromatin de-condensation. **(A)** Representative images acquired by phase contrast microscopy: 1^st^ *E*_*20*_ cells cultured for 24 h on *E*_*20*_. Yellow arrowhead: refractive objects corresponding to surviving cells; *E*_*20*_ cells cultured on the 1^st^ *E*_*20*_ for 24 h and transferred to new *E*_*20*_ for 24 h without an intermediate step on glass. Red arrowhead: non-refractive objects corresponding to necrotic cells. Scale bars: 100 µm. **(B)** Percentage of surviving SW480 cells from S4D for two independent experiments (the error bars represent the s.e.m.). **(C)** Representative ultrastructural images of cells cultured on 1^st^ *E*_*20*_ - glass, 2^nd^ *E*_*20*_ - glass, 3^rd^ *E*_*20*_ - glass and 4^th^ *E*_*20*_ - glass. Scale bars: 5 µm. **(D)** Percentage of heterochromatin in the nucleus of approximately 20 cells for each condition from C for two pooled independent experiments (error bars represents s.e.m.).

We demonstrated above that cancer cell survival and motility strongly depend on the chromatin de-condensation and that this phenomenon can be induced either chemically by treatment with TSA or purely mechanically by bringing cells successively and alternatively in contact with soft and hard substrates. Three hypotheses could be considered to account for this phenomenon: either (*i*) pre-existing cancer cells with the capacity to survive on soft substrate were selected during the first seeding on *E*_*20*_, or (*ii*) the survival of cancer cell on *E*_*20*_ was achieved via one or more genetic mutations, or (*iii)* survival resulted from a progressive adaptation linked to epigenetic modification of the chromatin in response to the mechanical change in the environment. In support of the second hypothesis, we have previously reported that SW480 cells display an increasing frequency of chromosomal segregation abnormalities when cultured on synthetic matrices with low stiffness and that a small proportion of cells surviving on *E*_*20*_ can undergo mitosis and a new cycle of replication even when bearing abnormal chromosome segregation^23^; thus, these cells could generate new chromosomal rearrangements that confer the capacity to survive on *E*_*20*_. However, this hypothesis, similar to first one, would imply that once surviving cells have been selected from the original population (according to the first hypothesis) or have emerged through mutation(s) (according to the second hypothesis), then the ability to survive on soft substrate should be irreversibly acquired and shared by almost all the cells in culture. To challenge this hypothesis, we have investigated the rate of cell survival on *E*_*20*_ after a previous seeding on 1^st^ *E*_*20*_ (cells characterized by a high level of heterochromatin), but without any intermediary step on glass. In this case, we observed that none of the cells did survive but instead, all of them died by necrosis (Fig. 7A,B). Moreover, when the same experiment was conducted using cells from the 4^th^ passage on *E*_*20*_ (cells characterized by a high level of euchromatin) transferred to the next *E*_*20*_ without an amplification step on glass, they underwent massive death by necrosis (4^th^ *E*_*20*_ - *E*_*20*_, Figs 7B and S6D). These observations prompted us to investigate what is the conformation of the chromatin in cells during these intermediate phases. TEM analysis showed that the cells that reached confluence on glass at the end of the 1^st^ *E*_*20*_ - glass, 2^nd^ *E*_*20*_ - glass, 3^rd^ *E*_*20*_ - glass and 4^th^ *E*_*20*_ - glass cycles exhibited euchromatin that was uniformly distributed within the nucleus with only a thin layer of heterochromatin at the nuclear periphery (Fig. 7C). The percentage of heterochromatin was, respectively, 13, 12, 13 and 10%, which is in the same range as in control cells continuously plated on glass (Fig. 4), but lower than the high percentage in cells on the 1^st^ *E*_*20*_ and 2^nd^ *E*_*20*_, and higher than the low percentage in cells on the 3^rd^ *E*_*20*_ and 4^th^ *E*_*20*_ (Fig. 7D). Taken together, these data show that although human colon cancer cells SW480 acquire the capacity to survive on soft substrates by successive passages on *E*_*20*_ - glass, this property is reversible and progressively increases with the number of passages on *E*_*20*_ - glass. We cannot formerly rule out that the sequential passages on soft and stiff substrates could lead to the selection of a resistant cell population involving the occurrence of gene mutations. This would require repeated exposures to the mechanical stress induced by the soft substrate. Consequently, the cells would gain irreversible capacity to survive on the soft substrate even without intermediate passages on a stiff substrate. In contrast, in our model, cells, even after their 4^th^ passage on a soft substrate, cannot survive the next step on a soft substrate unless they undergo an intermediate passage on a stiff substrate. Thus, the observations reported here support the hypothesis that the survival capacity acquired on a soft substrate reflects an adaptive process rather than the selection of a resistant cell population.

Furthermore cells growing continuously on hard substrate and those grown on a consecutives soft/hard substrate eventually exhibit similar behaviours. However, cells with soft/hard cycles might be endowed with a mechanical memory that predisposes them to survive the next seeding on the soft substrate. In agreement with our results, a mechanical memory has also been proposed for mesenchymal stem cells (MSCs) by switching the biophysical microenvironment^34,35^, or through changes in chromatin condensation for MSCs cultured on nanofibrous scaffolds and subjected to dynamic tensile loading^15^.

## Adaptation with changes in the gene expression profile

**Figure 8:**
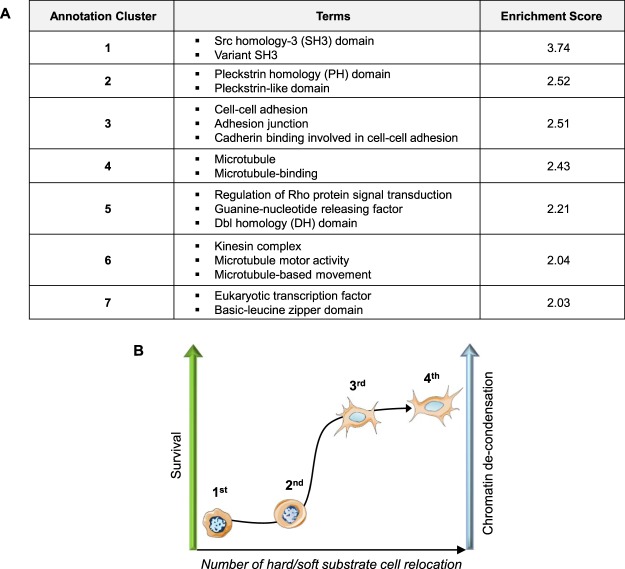
Functional clustering annotation of the differentially expressed genes. (**A**) Seven clusters identified with DAVID 6.8 with an enrichment score >2. (**B**) Cartoon summarizing the influence of the number of hard/soft substrate SW480 cell relocation on survival and chromatin conformation (hard = glass, soft = *E*_*20*_).

Since chromatin organization is related to gene expression, we compared the transcriptome of SW480 cells at the end of the 4^th^ *E*_*20*_ - glass step with cells continuously seeded on glass (4^th^ glass - glass). This experiments led to the identification of 2871 differentially expressed genes (fold change > 1.3; adjusted p-value < 0.01), representing 11.57% of the transcripts identified in this study (Supplementary Table 1). Functional annotation clustering identified terms related to protein interaction, cell adhesion and cytoskeleton, cell signalling and gene transcription (Fig. 8A). GO term analysis revealed that 27.83% (799/2871) of the genes were related to the “nucleus”, 19.71% (566/2871) to “acetylation”, and 11.04% (317/2871) to “transcription”. Among the transcription factor genes were bZIP factors, which were either upregulated (Maf, MafB, Bach2, and JunB) or downregulated (Nfe2l3). Maf factors are involved in the cell stress response and can interact alternatively with Bach or Nfe to promote transcription repression or activation^36^. It is also interesting that expression of the homeobox gene Cdx2 and the nuclear receptor family gene Hnf4α, two major regulators of intestinal homeostasis with tumour suppressor activity^37–39^, was downregulated, as well as stimulation of the Macc1 gene involved in metastasis^40^.

## Conclusion

The results reported herein are summarized schematically in Fig. 8B. Chromatin de-condensation appears to be the earliest early event in cell reprogramming. We demonstrate for the first time that purely mechanical cues elicit chromatin de-condensation similarly to TSA (inhibitor of histone deacetylase) in SW480 cancer cells. This new proposed approach is very easy to achieve by successive cell relocation to soft and glass adhesion substrates. Indeed, 4 glass-soft substrates cycles were effective to induce strong chromatin de-condensation in correlation with an increase of cellular survival. These cell fate changes corresponded to an adaptive and reversible process rather than irreversible gene mutation(s). This adaptive process was emphasized by modifications in gene expression patterns, including the genes for important transcription factors. This work revealsa key role of the mechanical properties of the cellular environment as epigenetic modulators and might suggest a mechanism of cell reprogramming.

## Methods

### Polyelectrolyte multilayer films

PLL (poly-L-lysine, MW = 5.7 × 10^4^ Da, Sigma, St. Quentin Fallavier, France) and HA (hyaluronic acid, MW = 4.0 × 10^5^ Da, BioIberica, Barcelona) were to construct the (PLL/HA)_24_ films, and PSS (poly sodium 4-styrenesulfonate, MW = 7.0 × 10^4^ Da, Sigma, St. Quentin Fallavier) and PAH (poly allylamine hydrochloride, MW = 7.0 × 10^4^ Da, Sigma) for the (PSS/PAH) capping films, which were deposited on top of the (PLL/HA)_24_ strata. PLL, HA, PSS, and PAH were dissolved at 1 mg/mL in a buffer solution containing 150 mM NaCl and 20 mM of tris(hydroxymethyl)-aminomethan (TRIS, Merck) at pH 7.4, and all rinsing steps were performed in the same buffer. (PLL/HA)_24_ strata and (PSS/PAH) capping films were prepared using a dipping machine (Dipping Robot DR3, Riegler & Kirstein GmbH, Berlin, Germany), on glass slides (VWR Scientific, Fontenay sous Bois, France). The apparent modulus of the (PLL/HA)_24_-(PSS/PAH) film was approximately 20 kPa^41,42^. For this film, we shall used the short-hand notation *E*_*20*_ (Fig. S5A,B).

### Cell culture

Colorectal adenocarcinoma SW480 cells (ATCC, CCL-228) were grown in RPMI-1640 medium (Invitrogen, Walthan, MA, USA) supplemented with glutamax, 10% FBS (Invitrogen), 100 µg/mL penicillin, 100 µg/mL streptomycin (Invitrogen), 0.025 U/mL insulin, 50 mg/mL hydrocortisone and 1.25 mg/mL G418 maintained at 37 °C with 5% CO_2_. To maintain the chromatin the euchromatin form on the 1^st^ *E*_*20*_, the cells were treated with 50 nM TSA (Sigma). On the 2^nd^ *E*_*20*_ cells that were cultured without drug during the 1^st^ *E*_*20*_ were treated with TSA during the 2^nd^ steps on *E*_*20*_. To maintain chromatin in the heterochromatin form on the 3^rd^ *E*_*20*_, cells cultured on the 1^st^ and 2^nd^ *E*_*20*_ without drug were treated with 100 µM ANA (Selleckchem) during the 3^rd^ step on *E*_*20*_.

### Cell culture model with changing mechanical properties

We studied the ability of SW480 cancer cells to survive changing mechanical properties by alternatively and repeatedly seeding them on soft and stiff matrices. More precisely, SW480 cells were seeded on *E*_*20*_-coated coverslips (denoted 1^st^ *E*_*20*_) placed in a 24-well plate (Nunc) at 1.10^5^ per cm^2^. The 1^st^ *E*_*20*_ matrices with the plated SW480 cells were moved for 24 h into new 24-well plates to avoid the collection of cells that would be in contact with the plastic surface of the well. The surviving cells were transferred by trypsin addition and amplified for two weeks on glass (denoted 1^st^ *E*_*20*_ - glass). Then, these cells were seeded for the second step of 24 h on *E*_*20*_ (denoted 2^nd^ *E*_*20*_) at 1.10^5^ per cm^2^, followed by amplification of the surviving cells on glass. The control glass substrate corresponded to cells that were seeded 5, 3, 3, 4 and 5 times seeded on glass by trypsinization for the life/dead assay (5^th^ glass), immunofluorescence microscopy (3^rd^ glass), live cell imaging (3^rd^ glass), electron microscopy (4^th^ glass) and transcriptomic analysis (5^th^ glass) respectively. Of note, the amplification time of the cells on glass was shorter due to the greater number of surviving relocated cells. The same procedures were performed up to the 5^th^ *E*_*20*_. The timing and conditions of the experiments are shown schematically in Fig. 1. For the glass + TSA and 1^st^ *E*_*20*_ + TSA conditions, cells in culture dishes were treated for 24 h with TSA and then seeded either on glass or on the 1^st^ *E*_*20*_ for 24 h, always in presence of TSA. For the 3^rd^ *E*_*20*_ + ANA condition, the cells that were amplified on glass after the 2^nd^ step on *E*_*20*_ were treated for 24 h with ANA and then transferred onto the 3^rd^ *E*_*20*_ for 24 h in the presence of ANA. Of note, the location on *E*_*20*_ over 24 h was followed by relocation to a new *E*_*20*_ for 24 h, which differed from the location of cells over 48 h on *E*_*20*_ because, in the first condition, dying cells that could potentially influence the behaviour of the surviving cells were no longer present on the new *E*_*20*_ substrate. Our data emphasize that the progressive acquisition of cell resistance by repeated seeding on soft matrices requires intermediate steps of cell proliferation on rigid substrate.

### Life/dead assay

See Supplementary Methods.

### Immunofluorescence microscopy

Cells were seeded on the matrices (see Fig. 1) at 1.10^5^ per cm^2^ and cultured for 24 h. The cells were then fixed/permeabilized in 3.7% (w/v) PFA in PBS plus 0.1% Triton X-100 for 15 min with 10% decomplemented FBS (Invitrogen). The cells were incubated with anti-vinculin (1:100, clone hVin-1, Sigma) and anti-acetyl-histone-H3-Lys14 (1:100; Millipore) and then incubated with FITC-conjugated secondary antibody (1:500; AnaSpec). The cells were incubated with TRITC-phalloidin (1 µg/mL) for actin staining and with Hoechst 33258 (20 µg/mL) for DNA. Samples were mounted in VectaShield (Vector Laboratories, Burlingame, CA). Fluorescence images were captured using a Nikon Elipse Ti-S with 60x PL APO (1.4 NA) objective equipped with a Nikon Digital Camera (DS-Qi 1Mc with NIS-Elements Br software) and processed with ImageJ. The cross-sectional area of the nucleus was measured in the median focus plane of the nuclei. Simultaneously, the fluorescence intensity in this plane, corresponding to the analysed protein, was recorded. This measurement provides fluorescence intensity per unit area of the nucleus and represents a qualitative indication of the protein level in the nucleus. Confocal microscope observations were performed with a Zeiss LSM 510 microscope using the x40/1.4 oil immersion objective. FITC-fluorescence was detected after excitation at 488 nm with a cut-off dichroic mirror of 488 nm and emission bandpass filter of 505–530 nm. Rho fluorescence was detected after excitation at 543 nm with dichroic mirror of 543 nm and emission long-pass filter of 585 nm.

### Live cell imaging

Cells were plated at 1.10^5^ per cm^2^ on *E*_*20*_ film-coated coverslips (see Fig. 1 for the experimental setup) and mounted in a Ludin Chamber (Life Imaging Services, Basel, Switzerland) at 37 °C, 5% CO_2_. After 24 h of culture in the incubator, the time-lapse sequence during 20 h was initiated on an Nikon Ti-E microscope equipped with a 40x PL PH2 (0.65 NA) objective and with a Andor Zyla sCMOS camera and driven by the Nikon NIS-Elements Ar software. Images were acquired every 15 min for 20 h simultaneously by phase contrast and by fluorescence microscopy with nuclear staining with Hoechst 33342 (2 µg/mL). Cell tracking was performed with the software “NIS-Elements Ar 3D tracking” (Nikon) was carried out in different fields of the substrates. First, the software detected objects, such as “nuclei”, by thresholding, which were then tracked over the 20 hours. Phase contrast images were used to check the viability of the followed cells. Cells that died during the experiment were eliminated. For each cell, we extracted speed data and (x, y) coordinates. From these coordinates, Sigma Plot software allowed the creation of a graph showing the cell trajectories, where the starting position of each cell was registered to the centre of the plot. ImageJ software was used to produce movies. In parallel, we measured the length of the nucleus for 100 cells in the different conditions, and the mean was 11.4 µm. To differentiate the cells that explored a more or less extended area, we chose to define a circle with a diameter equal to 2 times the mean length of the nuclei and centered on the starting point of each cell trajectory. Thus, we could discern two classes: the class 1 contained the cells that exited at least once from the circle with a radius of 11.4 μm, and class 2 represented the cells that did not exit from this circle (Fig. S6).

### Electron microscopy

See Supplementary Methods.

### Morphological analysis

See Supplementary Methods.

### Transcriptomic analysis

Cells amplified on glass for 1 week after the 4^th^ passage on *E*_*20*_ and control cells grown continuously on glass were collected for RNA preparation. RNA was extracted using TRI Reagent (Life Technologies Ambio, Houston, TX) and treated with DNAse (RQ1 RNAse-free DNAse, Promega Inc.). RNA integrity was checked on nano RNA chips with a Bioanalyser 2100 (Agilent Technologies, Santa Clara, CA, USA). Next, 1 µg of total RNA was used to construct the mRNA-seq libraries with the Illumina TruSeq RNA sample kit following the manufacturer’s instructions. Libraries were validated and quantified on DNA1000 chips and a Bioanalyser 2100. All samples were sequenced on the flow cell HS276 in 50-length single read. Reads were mapped onto the hg38 assembly of the Homo sapiens genome using Tophat 2.0.10^43,44^ and Bowtie version 2–2.1.0^45^. Only uniquely aligned reads were retained for further analyses. Quantification of gene expression was performed using HTSeq-0.6.1^46^. Read counts were then normalized across libraries as previously described^47^. Comparisons of interest were performed using the statistical method proposed by Anders and Huber^47,48^. The resulting p-values were adjusted for multiple testing using the Benjamini and Hochberg method^49^. Functional annotation was performed using DAVID 6.8^50^. The transcriptomic data are available under the following reference GSE 106 166.

### Statistical analysis

Statistical significance between different experimental setups for the percentage of heterochromatin, percentage of surviving cells, vinculin length, the mean speed of cell class 1 and fluorescence of Histone H3 acetylation were determined by one-way ANOVA test with Dunn’s method of correction for multiple comparisons. A value of p < 0.05 is noted on the graphs by **, which was considered statistically significant.

## Electronic supplementary material

Supplementary information

Supplementary Table 1

Movie 1

Movie 2

Movie 3

Movie 4

Movie 5

Movie 6

Movie 7
