# Supplementary material for: Chromatin de-condensation by switching substrate elasticity
Source: Sci Rep. 2018 Aug 23;8:12655. doi: 10.1038/s41598-018-31023-2 (PMC6107547; doi:10.1038/s41598-018-31023-2)
Supplement: Supplementary file 1 — Supplementary information [file 41598_2018_31023_MOESM1_ESM.pdf]

Chromatin de-condensation by switching substrate elasticity

Morgane Rabineau[a,b,e,1], Florence Flick[a,b,e,1], Claire Ehlinger[a,b,e], Eric Mathieu[a,b,e], Isabelle Duluc[c,e], Matthieu Jung[d], Bernard Senger[a,b,e], Leyla Kocgozlu[a,b,e], Pierre Schaaf[a,b,e], Philippe Lavalle[a,b,e], Jean-Noël Freund[c,e], Youssef Haikel[a,b,e], Dominique Vautier[a,b,e,*]

[a] Inserm UMR-S1121, 11 rue Humann, 67085 Strasbourg, France;
[b]Université de Strasbourg, Faculté de Chirurgie Dentaire, 8 rue Sainte Elisabeth, 67000 Strasbourg, France;
[c]Inserm UMR-S1113, 3 avenue Molière, 67200 Strasbourg, France;
[d]Université de Strasbourg, IGBMC Microarray and Sequencing Platform, Illkirch, France;
[e]Fédération de Médecine Translationnelle, Strasbourg, France.

[1]Morgane Rabineau and Florence Flick contributed equally to this work

*Corresponding author, Email: vautier@unistra.fr

Supplementary Information

Supplementary methods

Life/dead assay

Apoptotic, necrotic and healthy cells were assessed using the apoptotic/necrotic/healthy cells detection kit (PromoKine) according to the manufacturer's instructions. Cells were observed by fluorescence microscopy and analysed using ImageJ (http://rsb.info.nih.gov/ij/). Briefly, using ImageJ, the 3 channels blue, green and red corresponding, respectively, to the cells labelled with Hoechst 33342 (blue only, DNA), Annexin V-FITC (apoptotic cells) and EthD-III (necrotic cells) were superimposed. Quantification of positive cells in these different signals was performed using the ImageJ plugin "Cell Counter".

Electron microscopy

Cells seeded at 1.10^5 per cm^2 on the soft matrices (for notation see Fig. 1) were cultured for 24h and fixed in 2% PFA-2% glutaraldehyde in 50 mM cacodylate buffer at pH 7.4 for 2h and then fixed in 1% osmium tetroxide in 125 mM cacodylate for 30 min. For the 1st E_{20} - glass, 2nd E_{20} - glass, 3rd E_{20} - glass and 4th E_{20} - glass conditions, cells that reached confluence were fixed as described above at D15, D25, D32 and D39 respectively (see Fig. 1). Samples were dehydrated in solutions with a gradually increasing concentration of ethanol content (50, 70, 95, and 100% three times) for 15 min each. Cells in contact with the E_{20} films after 24h of culture were included in epoxy resin (48.2% epon 812, 34% anhydride nadic methyl, 16.4% anhydride [2-dodecenyl] succinic, and 1.5% 2,4,6-tris dimethylaminoetyl] phenol) for 48h at 60°C. After resin polymerization, to be able to cut them, a heat shock was first performed to remove the glass coverslips. To obtain transverse sections of cells, the cutting surface was reoriented by preparing a small block using a circular saw (Bronwill Scientific, USA) and adhering them to new ones. Ultra-thin cross-sections (100 nm) were acquired using an automatic ultramicrotome (Ultracut-E Ultramicrotome, Reichert Jung, USA). Sections containing the median plane of the nucleus with the nucleolus were selected for observations. Sections were stained with 5% uranyl acetate for 20 min and stained by 4% lead citrate. The specimens were observed with a transmission electron microscope EM208 (FEI Company, Philips, Netherlands) operating with an accelerating voltage of 70 kV. Images were captured on argentic SO163 Kodak films.

Morphological analysis

ImageJ was used to analyse the proportion of heterochromatin in the electron microscopy images. Approximately 20 cell nuclei were analysed for each data set. To determine the proportion of heterochromatin on the nuclear surface, we used a stereological method as we previously reported[11]. Briefly, after delimiting the nuclear compartment and calculating its surface in pixels, the area of the heterochromatin surface was determined by applying an intensity threshold on filtered images by Gaussian blur with radius 2. Then, the percentage of heterochromatin was determined by reporting the heterochromatin area in the total nuclear surface.

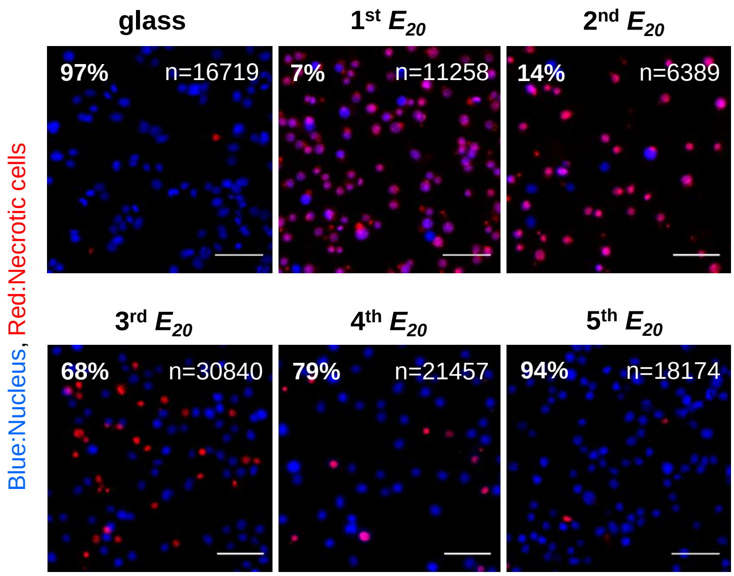

Supplementary Fig. S1

Representative images of cells cultured for 24h on glass, 1st E_{20}, 2nd E_{20}, 3rd E_{20}, 4th E_{20} and 5th E_{20} analysed using the apoptotic/necrotic/healthy cells kit.

Cell superpositions with Hoechst 33342 (blue: nucleus) and EthD-III (red: necrotic cells). %: percentage of surviving cells, n: number of cells counted. Scale bars: 50 µm.

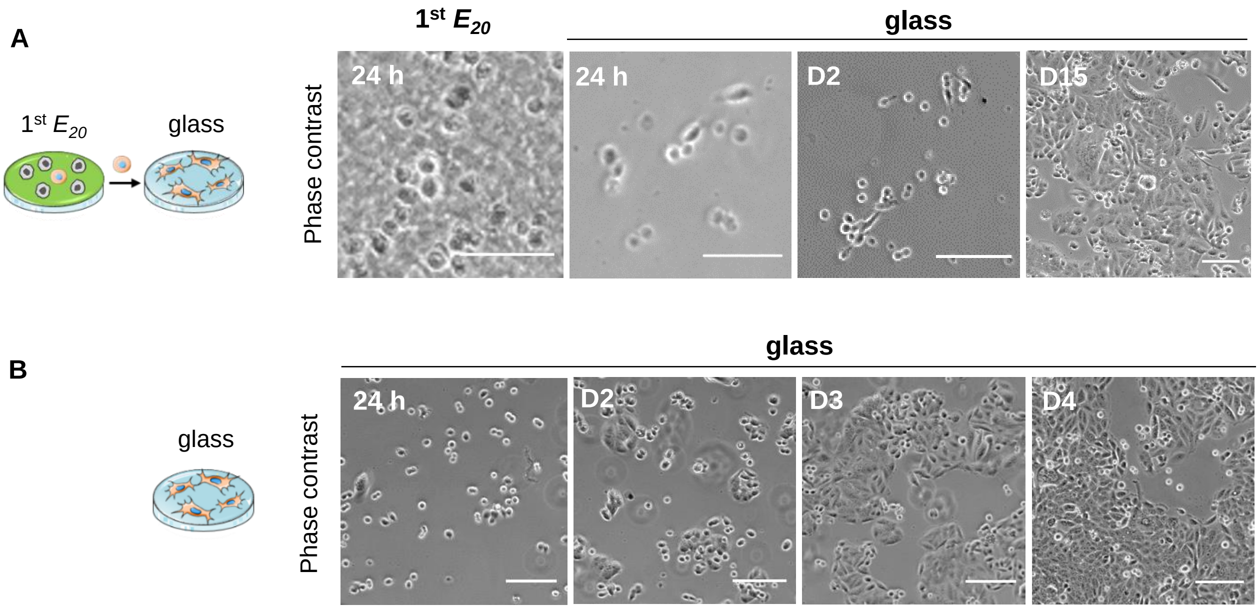

Supplementary Fig. S2

SW480 cells at different times after transfer to glass by phase contrast microscopy.

A) After 24h on E_{20} preceding transfer to glass. B) Without previous seeding on E_{20}. (A and B) Scale bars: 100 µm.

1st E_{20}

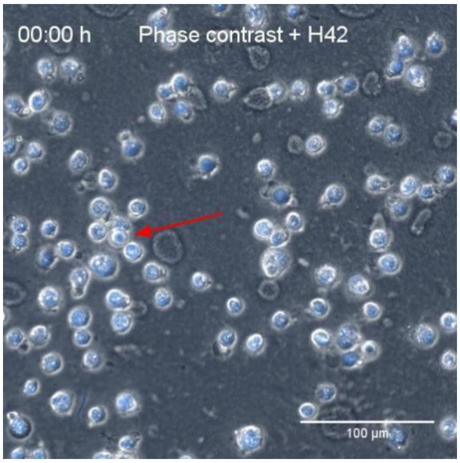

Movie 2

Supplementary Fig. S3

Time-lapse image of SW480 cells on 1st E_{20} by phase contrast microscopy and nuclear staining with Hoechst 33342 acquired every 15 min for two independent experiments. Red arrow=surviving cell.

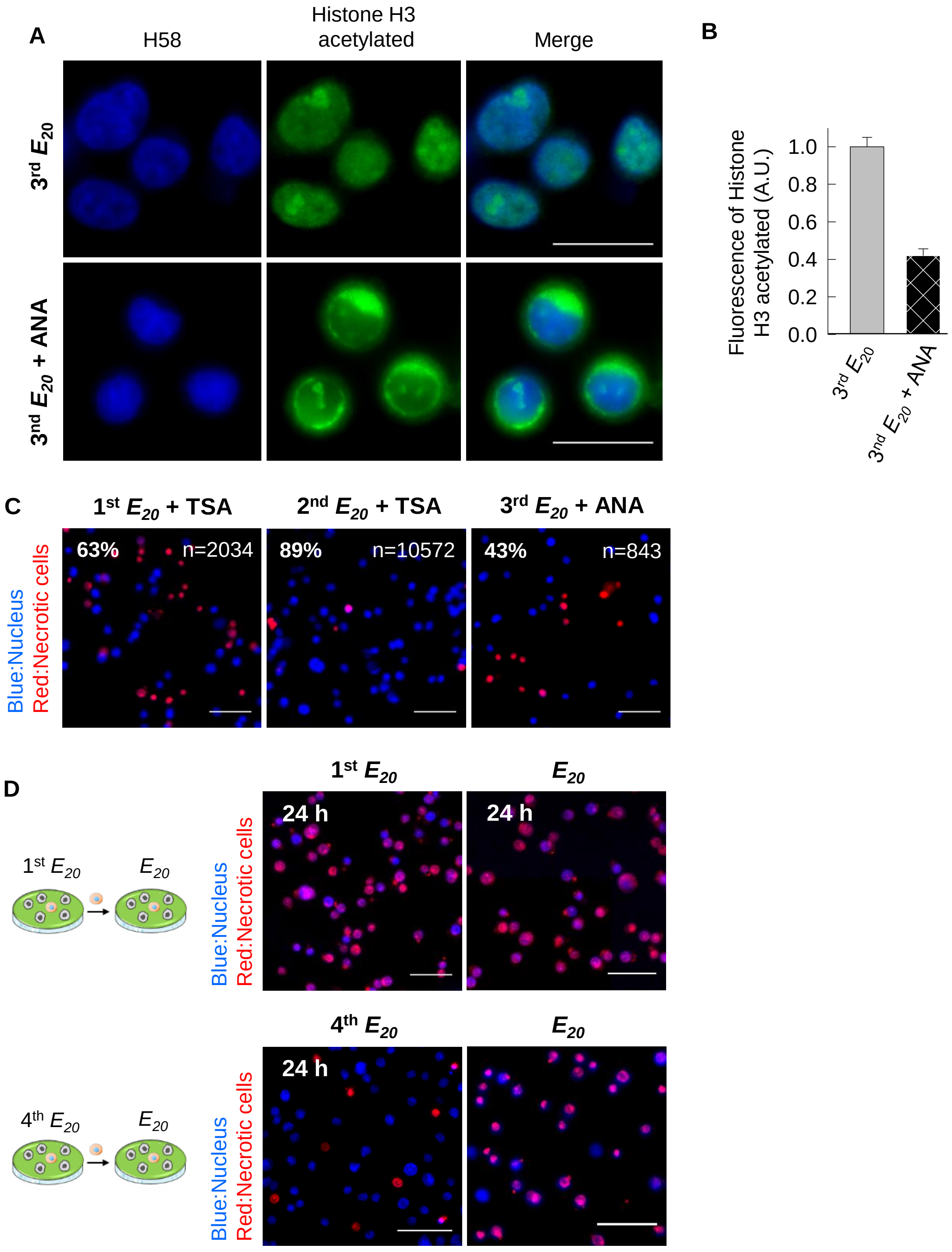

Supplementary Fig. S4

Histone H3 acetylation after ANA inhibition of SW480 cells on the 3nd ***E***$_{20}$**.**

A) Immunolabelling with anti-H3K14 and counterstaining with Hoechst 33258. For histone H3 acetylation inhibition, cells were cultured for 24h on 3nd E_{20} with ANA. Scale bars:20 µm. B) Fluorescence intensity, based on A, for the nuclear histone H3 acetylation signal using ImageJ (the value of 1 was arbitrarily attributed to cells on 3ndE_{20} without ANA). The results from two independent experiments are shown (error bars represent s.e.m.). C) Representative images of cells cultured for 24h on 1st E_{20} + TSA, 2nd E_{20} + TSA and 3rd E_{20} + ANA analysed using the apoptotic/necrotic/healthy cells kit. Cell superposition with Hoechst 33342 (blue: nucleus) and EthD-III (red: necrotic cells). The% and *n* in the images are the percentage of surviving cells and the number of cells counted, respectively. Scale bars: 50 µm. D) Representative images of cells cultured for 24h on the 1st E_{20} followed by 24h on E_{20} or 24h on the 4th E_{20} followed by 24h of culture on E_{20} (without an intermediate step) on glass analysed using the apoptotic/necrotic/healthy cells kit.

A

Structure	E (kPa)<br>Young modulus	Notation
$(PLL/HA)_{24}$-(PSS/PAH)	~ 20	E_{20}

B

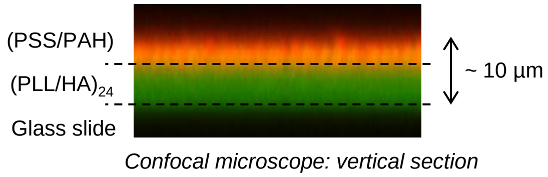

Supplementary Fig. S5

Polyelectrolyte multilayer films characterization.

A) Elastic modulus of $(PLL/HA)_{24}$-(PSS/PAH). B) Vertical section image of a $(PLL/HA)_{23}$-PLL^{FITC}-HA-$(PSS/PAH)_2$-PSS^{Rho}-PAH multi-layered film observed by confocal microscopy.

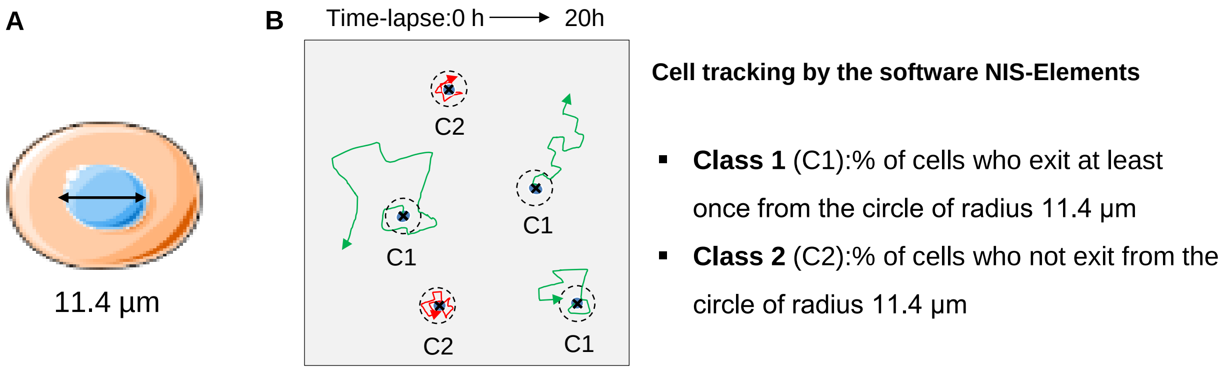

Supplementary Fig. S6

Method to discern two classes of cells.

A) Cartoon of a cell showing the mean size of the nuclei measured in 100 SW480 cells in different conditions (see Fig. 1). B) Example of different tracked cells..

***Supplementary* Table S1**

Differentially-expressed genes in cells at the 4th E_{20} - glass step versus cells at the 4th glass - glass step. Thresholds were:absolute Fold Change > 1.3;adjusted p-value < 0.01. RNAseq data were obtained from 3 independent samples in each case.
