# Supplementary material for: Chromatin de-condensation by switching substrate elasticity
Source: Sci Rep. 2018 Aug 23;8:12655. doi: 10.1038/s41598-018-31023-2 (PMC6107547; doi:10.1038/s41598-018-31023-2)
Supplement: Supplementary file 2 — Supplementary Table 1 [file 41598_2018_31023_MOESM2_ESM.pdf]

Ensembl gene id	Gene name	Description	log2(Select/CTRL)	FC (Select/CTRL)	P-value (Select vs CTRL)	Adjusted p-value (Select vs CTRL)
ENSG00000175899	A2M	alpha-2-macroglobulin	1,2039	2,3036	0,0000	0,0000
ENSG00000109576	AADAT	aminoadipate aminotransferase	-0,8066	0,5717	0,0000	0,0000
ENSG00000090861	AARS	alanyl-tRNA synthetase	1,0278	2,0390	0,0000	0,0000
ENSG00000157426	AASDH	aminoadipate-semialdehyde dehydrogenase	0,5177	1,4316	0,0000	0,0000
ENSG00000183044	ABAT	4-aminobutyrate aminotransferase	-1,0978	0,4672	0,0000	0,0000
ENSG00000107331	ABCA2	ATP-binding cassette, sub-family A (ABC1), member 2	0,6920	1,6155	0,0008	0,0079
ENSG00000167972	ABCA3	ATP-binding cassette, sub-family A (ABC1), member 3	0,7036	1,6286	0,0001	0,0018
ENSG00000005471	ABCB4	ATP-binding cassette, sub-family B (MDR/TAP), member 4	-1,4669	0,3618	0,0000	0,0000
ENSG00000125257	ABCC4	ATP-binding cassette, sub-family C (CFTR/MRP), member 4	-0,9326	0,5239	0,0000	0,0000
ENSG00000006071	ABCC8	ATP-binding cassette, sub-family C (CFTR/MRP), member 8	1,4312	2,6966	0,0000	0,0000
ENSG00000164163	ABCE1	ATP-binding cassette, sub-family E (OABP), member 1	-0,5461	0,6849	0,0000	0,0000
ENSG00000033050	ABCF2	ATP-binding cassette, sub-family F (GCN20), member 2	-0,3830	0,7668	0,0000	0,0004
ENSG00000160179	ABCG1	ATP-binding cassette, sub-family G (WHITE), member 1	1,1066	2,1534	0,0000	0,0000
ENSG00000143994	ABHD1	abhydrolase domain containing 1	0,9858	1,9804	0,0000	0,0000
ENSG00000106077	ABHD11	abhydrolase domain containing 11	0,4661	1,3814	0,0000	0,0000
ENSG00000139826	ABHD13	abhydrolase domain containing 13	-0,9917	0,5029	0,0000	0,0000
ENSG00000100439	ABHD4	abhydrolase domain containing 4	0,5024	1,4166	0,0000	0,0000
ENSG00000163995	ABLIM2	actin binding LIM protein family, member 2	0,6095	1,5258	0,0000	0,0000
ENSG00000173210	ABLIM3	actin binding LIM protein family, member 3	0,7856	1,7238	0,0005	0,0056
ENSG00000146386	ABRACL	ABRA C-terminal like	-0,4703	0,7218	0,0000	0,0004
ENSG00000114626	ABTB1	ankyrin repeat and BTB (POZ) domain containing 1	0,8758	1,8350	0,0000	0,0000
ENSG00000005189	AC004381.6	Putative RNA exonuclease NEF-sp	0,3834	1,3044	0,0001	0,0013
ENSG00000278540	ACACA	acetyl-CoA carboxylase alpha	-0,3912	0,7625	0,0000	0,0000
ENSG00000076555	ACACB	acetyl-CoA carboxylase beta	0,5995	1,5152	0,0000	0,0000
ENSG00000111271	ACAD10	acyl-CoA dehydrogenase family, member 10	0,4267	1,3442	0,0000	0,0005
ENSG00000122971	ACADS	acyl-CoA dehydrogenase, C-2 to C-3 short chain	0,9035	1,8706	0,0000	0,0000
ENSG00000072818	ACAP1	ArfGAP with coiled-coil, ankyrin repeat and PH domains 1	0,6641	1,5845	0,0000	0,0003
ENSG00000120437	ACAT2	acetyl-CoA acetyltransferase 2	-0,5997	0,6599	0,0000	0,0000

ENSG00000181513	ACBD4	acyl-CoA binding domain containing 4	0,8333	1,7818	0,0000	0,0000
ENSG00000078124	ACER3	alkaline ceramidase 3	-0,4270	0,7438	0,0000	0,0002
ENSG00000112304	ACOT13	acyl-CoA thioesterase 13	0,3861	1,3069	0,0002	0,0020
ENSG00000123130	ACOT9	acyl-CoA thioesterase 9	-0,4685	0,7227	0,0000	0,0000
ENSG00000168306	ACOX2	acyl-CoA oxidase 2, branched chain	1,1888	2,2796	0,0000	0,0000
ENSG00000111644	ACRBP	acrosin binding protein	1,0974	2,1398	0,0000	0,0000
ENSG00000167107	ACSF2	acyl-CoA synthetase family member 2	0,6754	1,5970	0,0000	0,0000
ENSG00000068366	ACSL4	acyl-CoA synthetase long-chain family member 4	-0,5447	0,6855	0,0000	0,0000
ENSG00000005187	ACSM3	acyl-CoA synthetase medium-chain family member 3	0,9472	1,9281	0,0000	0,0000
ENSG00000111058	ACSS3	acyl-CoA synthetase short-chain family member 3	1,0427	2,0601	0,0001	0,0018
ENSG00000178631	ACTG1P1	actin gamma 1 pseudogene 1	1,0044	2,0062	0,0002	0,0026
ENSG00000117148	ACTL8	actin-like 8	-0,9892	0,5038	0,0002	0,0022
ENSG00000115091	ACTR3	ARP3 actin-related protein 3 homolog (yeast)	-0,5025	0,7059	0,0000	0,0000
ENSG00000184378	ACTRT3	actin-related protein T3	-0,6170	0,6520	0,0001	0,0016
ENSG00000114739	ACVR2B	activin A receptor, type IIB	0,5105	1,4246	0,0000	0,0000
ENSG00000229589	ACVR2B-AS1	ACVR2B antisense RNA 1	0,6895	1,6127	0,0000	0,0000
ENSG00000196839	ADA	adenosine deaminase	0,5118	1,4258	0,0000	0,0000
ENSG00000160323	ADAMTS13	ADAM metallopeptidase with thrombospondin type 1 motif, 13	1,0591	2,0836	0,0000	0,0000
ENSG00000145808	ADAMTS19	ADAM metallopeptidase with thrombospondin type 1 motif, 19	0,6796	1,6017	0,0000	0,0000
ENSG00000087116	ADAMTS2	ADAM metallopeptidase with thrombospondin type 1 motif, 2	-1,2122	0,4316	0,0000	0,0002
ENSG00000185761	ADAMTSL5	ADAMTS-like 5	0,6162	1,5328	0,0001	0,0017
ENSG00000163050	ADCK3	aarF domain containing kinase 3	0,4774	1,3923	0,0000	0,0000
ENSG00000123815	ADCK4	aarF domain containing kinase 4	0,4807	1,3955	0,0000	0,0005
ENSG00000181790	ADGRB1	adhesion G protein-coupled receptor B1	1,4683	2,7670	0,0000	0,0000
ENSG00000272734	ADIRF-AS1	ADIRF antisense RNA 1	0,4416	1,3581	0,0000	0,0004
ENSG00000148926	ADM	adrenomedullin	0,6277	1,5451	0,0000	0,0000
ENSG00000128165	ADM2	adrenomedullin 2	1,6954	3,2387	0,0000	0,0000
ENSG00000170214	ADRA1B	adrenoceptor alpha 1B	-0,6301	0,6461	0,0000	0,0000
ENSG00000173020	ADRBK1	adrenergic, beta, receptor kinase 1	0,5507	1,4648	0,0000	0,0000
ENSG00000100077	ADRBK2	adrenergic, beta, receptor kinase 2	-0,4460	0,7341	0,0000	0,0000
ENSG00000185100	ADSSL1	adenylosuccinate synthase like 1	0,7325	1,6616	0,0000	0,0000

ENSG00000155966	AFF2	AF4/FMR2 family, member 2	-0,7784	0,5830	0,0001	0,0013
ENSG00000144218	AFF3	AF4/FMR2 family, member 3	-0,6186	0,6513	0,0000	0,0000
ENSG00000183077	AFMID	arylformamidase	0,5296	1,4435	0,0000	0,0000
ENSG00000134698	AGO4	argonaute RISC catalytic component 4	0,5166	1,4305	0,0000	0,0000
ENSG00000026652	AGPAT4	1-acylglycerol-3-phosphate O-acyltransferase 4	-0,9514	0,5171	0,0004	0,0046
ENSG00000185567	AHNAK2	AHNAK nucleoprotein 2	0,6906	1,6139	0,0000	0,0000
ENSG00000106546	AHR	aryl hydrocarbon receptor	-0,4504	0,7319	0,0000	0,0000
ENSG00000063438	AHRR	aryl-hydrocarbon receptor repressor	1,1708	2,2514	0,0000	0,0000
ENSG00000100591	AHSA1	AHA1, activator of heat shock 90kDa protein ATPase homolog 1 (yeast)	-0,3850	0,7658	0,0001	0,0018
ENSG00000112297	AIM1	absent in melanoma 1	0,5612	1,4755	0,0005	0,0053
ENSG00000176092	AIM1L	absent in melanoma 1-like	0,5065	1,4206	0,0004	0,0041
ENSG00000110711	AIP	aryl hydrocarbon receptor interacting protein	0,4992	1,4135	0,0001	0,0008
ENSG00000085231	AK6	adenylate kinase 6	-0,5023	0,7060	0,0000	0,0003
ENSG00000165695	AK8	adenylate kinase 8	0,8534	1,8068	0,0005	0,0051
ENSG00000106948	AKNA	AT-hook transcription factor	0,7691	1,7042	0,0000	0,0000
ENSG00000059573	ALDH18A1	aldehyde dehydrogenase 18 family, member A1	-0,4588	0,7276	0,0000	0,0000
ENSG00000136010	ALDH1L2	aldehyde dehydrogenase 1 family, member L2	0,9422	1,9215	0,0000	0,0001
ENSG00000108602	ALDH3A1	aldehyde dehydrogenase 3 family, member A1	1,0385	2,0540	0,0000	0,0000
ENSG00000006534	ALDH3B1	aldehyde dehydrogenase 3 family, member B1	1,1509	2,2206	0,0000	0,0000
ENSG00000119711	ALDH6A1	aldehyde dehydrogenase 6 family, member A1	0,4474	1,3636	0,0000	0,0000
ENSG00000109107	ALDOC	aldolase C, fructose-bisphosphate	1,8633	3,6385	0,0000	0,0000
ENSG00000253710	ALG11	ALG11, alpha-1,2-mannosyltransferase	-0,6093	0,6555	0,0000	0,0000
ENSG00000248671	ALG1L9P	asparagine-linked glycosylation 1-like 9, pseudogene	0,7030	1,6278	0,0004	0,0040
ENSG00000120697	ALG5	ALG5, dolichyl-phosphate beta-glucosyltransferase	-0,7236	0,6056	0,0000	0,0000
ENSG00000159063	ALG8	ALG8, alpha-1,3-glucosyltransferase	-0,3995	0,7581	0,0001	0,0013
ENSG00000125652	ALKBH7	alkB homolog 7	0,8616	1,8171	0,0000	0,0000
ENSG00000137760	ALKBH8	alkB homolog 8, tRNA methyltransferase	-0,4858	0,7141	0,0000	0,0000
ENSG00000179477	ALOX12B	arachidonate 12-lipoxygenase, 12R type	0,4754	1,3903	0,0005	0,0053
ENSG00000179593	ALOX15B	arachidonate 15-lipoxygenase, type B	0,9074	1,8756	0,0003	0,0031
ENSG00000163295	ALPI	alkaline phosphatase, intestinal	2,0234	4,0654	0,0000	0,0000
ENSG00000198796	ALPK2	alpha-kinase 2	-1,1074	0,4641	0,0000	0,0000

ENSG00000163283	ALPP	alkaline phosphatase, placental	0,7795	1,7165	0,0000	0,0000
ENSG00000178038	ALS2CL	ALS2 C-terminal like	0,4648	1,3802	0,0005	0,0053
ENSG00000123505	AMD1	adenosylmethionine decarboxylase 1	-0,8997	0,5360	0,0000	0,0000
ENSG00000162066	AMDHD2	amidohydrolase domain containing 2	0,6681	1,5890	0,0000	0,0005
ENSG00000101935	AMMECR1	Alport syndrome, mental retardation, midface hypoplasia and elliptocytosis chrom	-0,4634	0,7253	0,0000	0,0000
ENSG00000126016	AMOT	angiomotin	0,5088	1,4228	0,0000	0,0000
ENSG00000114019	AMOTL2	angiomotin like 2	-0,5358	0,6898	0,0000	0,0000
ENSG00000145020	AMT	aminomethyltransferase	0,9814	1,9744	0,0000	0,0000
ENSG00000153107	ANAPC1	anaphase promoting complex subunit 1	-0,4374	0,7384	0,0000	0,0000
ENSG00000053900	ANAPC4	anaphase promoting complex subunit 4	-0,3982	0,7588	0,0007	0,0070
ENSG00000196510	ANAPC7	anaphase promoting complex subunit 7	-0,3949	0,7605	0,0000	0,0003
ENSG00000214274	ANG	angiogenin, ribonuclease, RNase A family, 5	0,5887	1,5039	0,0010	0,0094
ENSG00000148677	ANKRD1	ankyrin repeat domain 1 (cardiac muscle)	-0,8677	0,5480	0,0000	0,0000
ENSG00000088448	ANKRD10	ankyrin repeat domain 10	-0,8704	0,5470	0,0000	0,0000
ENSG00000229152	ANKRD10-IT1	ANKRD10 intronic transcript 1	-1,0043	0,4985	0,0000	0,0000
ENSG00000196593	ANKRD20A19P	ankyrin repeat domain 20 family, member A19, pseudogene	1,0959	2,1374	0,0000	0,0000
ENSG00000154065	ANKRD29	ankyrin repeat domain 29	0,7999	1,7410	0,0000	0,0000
ENSG00000235711	ANKRD34C	ankyrin repeat domain 34C	1,0497	2,0701	0,0001	0,0010
ENSG00000186352	ANKRD37	ankyrin repeat domain 37	1,1682	2,2473	0,0000	0,0000
ENSG00000131620	ANO1	anoctamin 1, calcium activated chloride channel	-0,5416	0,6870	0,0000	0,0000
ENSG00000151572	ANO4	anoctamin 4	0,4521	1,3681	0,0000	0,0000
ENSG00000074855	ANO8	anoctamin 8	0,5611	1,4754	0,0007	0,0070
ENSG00000185101	ANO9	anoctamin 9	0,4552	1,3710	0,0007	0,0072
ENSG00000143401	ANP32E	acidic (leucine-rich) nuclear phosphoprotein 32 family, member E	-0,4082	0,7536	0,0000	0,0001
ENSG00000143412	ANXA9	annexin A9	0,7926	1,7322	0,0000	0,0000
ENSG00000100478	AP4S1	adaptor-related protein complex 4, sigma 1 subunit	-0,4710	0,7215	0,0000	0,0005
ENSG00000053770	AP5M1	adaptor-related protein complex 5, mu 1 subunit	-0,5051	0,7046	0,0000	0,0000
ENSG00000166313	APBB1	amyloid beta (A4) precursor protein-binding, family B, member 1 (Fe65)	-0,5068	0,7038	0,0008	0,0074
ENSG00000163697	APBB2	amyloid beta (A4) precursor protein-binding, family B, member 2	0,5522	1,4663	0,0000	0,0000
ENSG00000113108	APBB3	amyloid beta (A4) precursor protein-binding, family B, member 3	0,6085	1,5247	0,0000	0,0000
ENSG00000084234	APLP2	amyloid beta (A4) precursor-like protein 2	-0,4719	0,7210	0,0000	0,0000

ENSG00000244509	APOBEC3C	apolipoprotein B mRNA editing enzyme, catalytic polypeptide-like 3C	-0,5322	0,6915	0,0000	0,0000
ENSG00000130203	APOE	apolipoprotein E	0,7328	1,6619	0,0000	0,0000
ENSG00000100342	APOL1	apolipoprotein L, 1	0,4673	1,3825	0,0008	0,0077
ENSG00000221963	APOL6	apolipoprotein L, 6	0,4105	1,3292	0,0001	0,0017
ENSG00000240583	AQP1	aquaporin 1 (Colton blood group)	0,9415	1,9205	0,0001	0,0013
ENSG00000169083	AR	androgen receptor	1,2528	2,3831	0,0000	0,0000
ENSG00000186635	ARAP1	ArfGAP with RhoGAP domain, ankyrin repeat and PH domain 1	0,4449	1,3613	0,0002	0,0022
ENSG00000134287	ARF3	ADP-ribosylation factor 3	-0,4899	0,7121	0,0000	0,0000
ENSG00000081181	ARG2	arginase 2	0,3862	1,3069	0,0000	0,0001
ENSG00000134884	ARGLU1	arginine and glutamate rich 1	-0,9408	0,5210	0,0000	0,0000
ENSG00000163219	ARHGAP25	Rho GTPase activating protein 25	0,9494	1,9311	0,0005	0,0055
ENSG00000137962	ARHGAP29	Rho GTPase activating protein 29	-0,4038	0,7559	0,0000	0,0000
ENSG00000031081	ARHGAP31	Rho GTPase activating protein 31	-1,1661	0,4456	0,0000	0,0000
ENSG00000004777	ARHGAP33	Rho GTPase activating protein 33	0,6846	1,6073	0,0000	0,0002
ENSG00000089820	ARHGAP4	Rho GTPase activating protein 4	0,5378	1,4517	0,0000	0,0006
ENSG00000124143	ARHGAP40	Rho GTPase activating protein 40	0,8267	1,7737	0,0000	0,0000
ENSG00000165895	ARHGAP42	Rho GTPase activating protein 42	0,4225	1,3402	0,0001	0,0017
ENSG00000123329	ARHGAP9	Rho GTPase activating protein 9	1,2906	2,4462	0,0000	0,0000
ENSG00000074964	ARHGEF10L	Rho guanine nucleotide exchange factor (GEF) 10-like	0,6214	1,5384	0,0001	0,0008
ENSG00000130762	ARHGEF16	Rho guanine nucleotide exchange factor (GEF) 16	0,8038	1,7457	0,0000	0,0000
ENSG00000110237	ARHGEF17	Rho guanine nucleotide exchange factor (GEF) 17	0,6499	1,5691	0,0000	0,0000
ENSG00000240771	ARHGEF25	Rho guanine nucleotide exchange factor (GEF) 25	0,5595	1,4738	0,0000	0,0000
ENSG00000114790	ARHGEF26	Rho guanine nucleotide exchange factor (GEF) 26	1,0835	2,1192	0,0000	0,0000
ENSG00000214944	ARHGEF28	Rho guanine nucleotide exchange factor (GEF) 28	0,4696	1,3847	0,0000	0,0001
ENSG00000163947	ARHGEF3	Rho guanine nucleotide exchange factor (GEF) 3	-0,3809	0,7680	0,0000	0,0001
ENSG00000183111	ARHGEF37	Rho guanine nucleotide exchange factor (GEF) 37	0,6960	1,6200	0,0000	0,0000
ENSG00000236699	ARHGEF38	Rho guanine nucleotide exchange factor (GEF) 38	0,7358	1,6653	0,0002	0,0028
ENSG00000102606	ARHGEF7	Rho guanine nucleotide exchange factor (GEF) 7	-0,6631	0,6315	0,0000	0,0000
ENSG00000122644	ARL4A	ADP-ribosylation factor-like 4A	-0,3940	0,7610	0,0001	0,0018
ENSG00000170540	ARL6IP1	ADP-ribosylation factor-like 6 interacting protein 1	-0,5041	0,7051	0,0000	0,0000
ENSG00000170632	ARMC10	armadillo repeat containing 10	-0,4840	0,7150	0,0000	0,0000

ENSG00000271147	ARMCX5-GPRASP2	ARMCX5-GPRASP2 readthrough	-0,5430	0,6863	0,0009	0,0089
ENSG00000143437	ARNT	aryl hydrocarbon receptor nuclear translocator	0,4686	1,3838	0,0000	0,0000
ENSG00000133794	ARNTL	aryl hydrocarbon receptor nuclear translocator-like	-0,5204	0,6972	0,0000	0,0000
ENSG00000163466	ARPC2	actin related protein 2/3 complex, subunit 2, 34kDa	-0,4056	0,7549	0,0000	0,0001
ENSG00000241553	ARPC4	actin related protein 2/3 complex, subunit 4, 20kDa	-0,3794	0,7688	0,0000	0,0003
ENSG00000136950	ARPC5L	actin related protein 2/3 complex, subunit 5-like	-0,4161	0,7494	0,0000	0,0002
ENSG00000197070	ARRDC1	arrestin domain containing 1	0,4163	1,3345	0,0003	0,0036
ENSG00000113369	ARRDC3	arrestin domain containing 3	0,4326	1,3496	0,0000	0,0000
ENSG00000117407	ARTN	artemin	0,9619	1,9479	0,0000	0,0000
ENSG00000099889	ARVCF	armadillo repeat gene deleted in velocardiofacial syndrome	0,4958	1,4101	0,0009	0,0083
ENSG00000005981	ASB4	ankyrin repeat and SOCS box containing 4	-0,5037	0,7053	0,0000	0,0000
ENSG00000112249	ASCC3	activating signal cointegrator 1 complex subunit 3	-0,4388	0,7377	0,0000	0,0000
ENSG00000110881	ASIC1	acid sensing (proton gated) ion channel 1	0,4414	1,3579	0,0000	0,0004
ENSG00000070669	ASNS	asparagine synthetase (glutamine-hydrolyzing)	1,2432	2,3673	0,0000	0,0000
ENSG00000198363	ASPH	aspartate beta-hydroxylase	-0,4536	0,7302	0,0000	0,0000
ENSG00000128203	ASPHD2	aspartate beta-hydroxylase domain containing 2	-0,6832	0,6228	0,0000	0,0001
ENSG00000066279	ASPM	abnormal spindle microtubule assembly	-0,5620	0,6774	0,0000	0,0000
ENSG00000160072	ATAD3B	ATPase family, AAA domain containing 3B	-0,3846	0,7660	0,0008	0,0077
ENSG00000162772	ATF3	activating transcription factor 3	0,4452	1,3615	0,0001	0,0013
ENSG00000169136	ATF5	activating transcription factor 5	-0,4502	0,7319	0,0000	0,0001
ENSG00000085978	ATG16L1	autophagy related 16-like 1	-0,4555	0,7293	0,0000	0,0000
ENSG00000168010	ATG16L2	autophagy related 16-like 2	0,7844	1,7224	0,0000	0,0000
ENSG00000110046	ATG2A	autophagy related 2A	0,6339	1,5517	0,0000	0,0001
ENSG00000130734	ATG4D	autophagy related 4D, cysteine peptidase	0,5576	1,4719	0,0000	0,0006
ENSG00000198925	ATG9A	autophagy related 9A	0,4025	1,3218	0,0001	0,0009
ENSG00000181652	ATG9B	autophagy related 9B	0,9701	1,9590	0,0004	0,0039
ENSG00000142102	ATHL1	ATH1, acid trehalase-like 1 (yeast)	1,3195	2,4957	0,0000	0,0000
ENSG00000168874	ATOH8	atonal bHLH transcription factor 8	-0,9453	0,5193	0,0005	0,0054
ENSG00000068650	ATP11A	ATPase, class VI, type 11A	-0,7818	0,5816	0,0000	0,0000
ENSG00000101974	ATP11C	ATPase, class VI, type 11C	-0,4048	0,7553	0,0000	0,0001
ENSG00000133657	ATP13A3	ATPase type 13A3	-0,4513	0,7314	0,0000	0,0000

ENSG00000069849	ATP1B3	ATPase, Na+/K+ transporting, beta 3 polypeptide	-0,4656	0,7242	0,0000	0,0001
ENSG00000074370	ATP2A3	ATPase, Ca++ transporting, ubiquitous	0,5389	1,4528	0,0008	0,0073
ENSG00000070961	ATP2B1	ATPase, Ca++ transporting, plasma membrane 1	-0,5324	0,6914	0,0000	0,0000
ENSG00000058668	ATP2B4	ATPase, Ca++ transporting, plasma membrane 4	-0,7507	0,5943	0,0000	0,0000
ENSG00000185344	ATP6V0A2	ATPase, H+ transporting, lysosomal V0 subunit a2	-0,5071	0,7036	0,0000	0,0000
ENSG00000100554	ATP6V1D	ATPase, H+ transporting, lysosomal 34kDa, V1 subunit D	-0,7083	0,6120	0,0000	0,0000
ENSG00000132932	ATP8A2	ATPase, aminophospholipid transporter, class I, type 8A, member 2	-0,7041	0,6138	0,0000	0,0001
ENSG00000081923	ATP8B1	ATPase, aminophospholipid transporter, class I, type 8B, member 1	0,9297	1,9049	0,0000	0,0002
ENSG00000107518	ATRNL1	attractin-like 1	-0,8990	0,5362	0,0000	0,0005
ENSG00000124788	ATXN1	ataxin 1	0,4086	1,3274	0,0000	0,0000
ENSG00000130638	ATXN10	ataxin 10	-0,4150	0,7500	0,0000	0,0007
ENSG00000168488	ATXN2L	ataxin 2-like	0,3837	1,3047	0,0000	0,0001
ENSG00000160862	AZGP1	alpha-2-glycoprotein 1, zinc-binding	2,1983	4,5893	0,0000	0,0000
ENSG00000214313	AZGP1P1	alpha-2-glycoprotein 1, zinc-binding pseudogene 1	0,9457	1,9261	0,0005	0,0049
ENSG00000142920	AZIN2	antizyme inhibitor 2	0,7218	1,6493	0,0000	0,0002
ENSG00000187676	B3GLCT	beta 3-glucosyltransferase	-0,7856	0,5801	0,0000	0,0000
ENSG00000182272	B4GALNT4	beta-1,4-N-acetyl-galactosaminyl transferase 4	0,4496	1,3656	0,0009	0,0089
ENSG00000118276	B4GALT6	UDP-Gal:betaGlcNAc beta 1,4- galactosyltransferase, polypeptide 6	-0,7881	0,5791	0,0000	0,0000
ENSG00000108641	B9D1	B9 protein domain 1	0,4437	1,3601	0,0001	0,0009
ENSG00000112182	BACH2	BTB and CNC homology 1, basic leucine zipper transcription factor 2	1,2665	2,4057	0,0000	0,0000
ENSG00000002330	BAD	BCL2-associated agonist of cell death	0,4269	1,3444	0,0005	0,0054
ENSG00000107262	BAG1	BCL2-associated athanogene	-0,4096	0,7528	0,0001	0,0008
ENSG00000166170	BAG5	BCL2-associated athanogene 5	-0,5979	0,6607	0,0000	0,0000
ENSG00000007516	BAIAP3	BAI1-associated protein 3	1,6897	3,2259	0,0000	0,0000
ENSG00000198604	BAZ1A	bromodomain adjacent to zinc finger domain, 1A	-0,5942	0,6624	0,0000	0,0000
ENSG00000105327	BBC3	BCL2 binding component 3	1,0598	2,0847	0,0000	0,0000
ENSG00000125124	BBS2	Bardet-Biedl syndrome 2	0,6778	1,5998	0,0000	0,0000
ENSG00000075790	BCAP29	B-cell receptor-associated protein 29	-0,5519	0,6821	0,0000	0,0000
ENSG00000141376	BCAS3	breast carcinoma amplified sequence 3	0,9892	1,9852	0,0000	0,0000
ENSG00000105552	BCAT2	branched chain amino-acid transaminase 2, mitochondrial	0,5445	1,4585	0,0000	0,0000
ENSG00000107949	BCCIP	BRCA2 and CDKN1A interacting protein	-0,5674	0,6748	0,0000	0,0000

ENSG00000171791	BCL2	B-cell CLL/lymphoma 2	-0,6384	0,6424	0,0008	0,0074
ENSG00000188761	BCL2L15	BCL2-like 15	-0,7614	0,5899	0,0000	0,0000
ENSG00000113916	BCL6	B-cell CLL/lymphoma 6	0,6041	1,5201	0,0000	0,0006
ENSG00000029363	BCLAF1	BCL2-associated transcription factor 1	-0,5084	0,7030	0,0000	0,0000
ENSG00000236824	BCYRN1	brain cytoplasmic RNA 1	-0,6137	0,6535	0,0000	0,0000
ENSG00000166546	BEAN1	brain expressed, associated with NEDD4, 1	0,9831	1,9767	0,0000	0,0000
ENSG00000178409	BEND3	BEN domain containing 3	-0,3852	0,7657	0,0002	0,0020
ENSG00000167995	BEST1	bestrophin 1	1,5486	2,9253	0,0000	0,0000
ENSG00000133134	BEX2	brain expressed X-linked 2	0,6881	1,6112	0,0000	0,0000
ENSG00000180535	BHLHA15	basic helix-loop-helix family, member a15	0,9417	1,9208	0,0003	0,0034
ENSG00000134107	BHLHE40	basic helix-loop-helix family, member e40	0,4846	1,3992	0,0000	0,0000
ENSG00000123095	BHLHE41	basic helix-loop-helix family, member e41	1,3451	2,5404	0,0000	0,0000
ENSG00000237452	BHMG1	basic helix-loop-helix and HMG box domain containing 1	0,9173	1,8886	0,0008	0,0080
ENSG00000122870	BICC1	BicC family RNA binding protein 1	-0,6061	0,6570	0,0000	0,0000
ENSG00000100290	BIK	BCL2-interacting killer (apoptosis-inducing)	0,4901	1,4046	0,0000	0,0001
ENSG00000147439	BIN3	bridging integrator 3	-0,4138	0,7506	0,0001	0,0012
ENSG00000023445	BIRC3	baculoviral IAP repeat containing 3	-1,2393	0,4236	0,0000	0,0000
ENSG00000089685	BIRC5	baculoviral IAP repeat containing 5	-0,4429	0,7357	0,0000	0,0001
ENSG00000134897	BIVM	basic, immunoglobulin-like variable motif containing	-0,9083	0,5328	0,0000	0,0000
ENSG00000166619	BLCAP	bladder cancer associated protein	-0,5619	0,6774	0,0000	0,0000
ENSG00000090013	BLVRB	biliverdin reductase B	0,3794	1,3008	0,0008	0,0079
ENSG00000104081	BMF	Bcl2 modifying factor	1,0558	2,0788	0,0000	0,0000
ENSG00000168283	BMI1	BMI1 proto-oncogene, polycomb ring finger	-0,5143	0,7001	0,0000	0,0000
ENSG00000125378	BMP4	bone morphogenetic protein 4	-0,5273	0,6938	0,0000	0,0000
ENSG00000153162	BMP6	bone morphogenetic protein 6	-0,6584	0,6336	0,0000	0,0000
ENSG00000116985	BMP8B	bone morphogenetic protein 8b	0,7223	1,6498	0,0000	0,0000
ENSG00000138696	BMPR1B	bone morphogenetic protein receptor, type IB	1,0816	2,1164	0,0000	0,0005
ENSG00000165733	BMS1	BMS1 ribosome biogenesis factor	-0,5357	0,6898	0,0000	0,0000
ENSG00000173068	BNC2	basonuclin 2	-0,4681	0,7229	0,0001	0,0017
ENSG00000178096	BOLA1	bolA family member 1	0,6953	1,6193	0,0000	0,0001
ENSG00000136122	BORA	bora, aurora kinase A activator	-1,1942	0,4370	0,0000	0,0000

ENSG00000139618	BRCA2	breast cancer 2, early onset	-0,4309	0,7418	0,0000	0,0000
ENSG00000185515	BRCC3	BRCA1/BRCA2-containing complex, subunit 3	-0,4813	0,7163	0,0000	0,0000
ENSG00000113460	BRIX1	BRX1, biogenesis of ribosomes	-0,6326	0,6450	0,0000	0,0000
ENSG00000160058	BSDC1	BSD domain containing 1	0,4583	1,3740	0,0000	0,0000
ENSG00000151136	BTBD11	BTB (POZ) domain containing 11	-0,6197	0,6508	0,0000	0,0000
ENSG00000133243	BTBD2	BTB (POZ) domain containing 2	0,5237	1,4376	0,0003	0,0036
ENSG00000183826	BTBD9	BTB (POZ) domain containing 9	0,5195	1,4334	0,0000	0,0000
ENSG00000159388	BTG2	BTG family, member 2	0,5572	1,4714	0,0000	0,0000
ENSG00000026950	BTN3A1	butyrophilin, subfamily 3, member A1	1,3713	2,5869	0,0000	0,0000
ENSG00000186470	BTN3A2	butyrophilin, subfamily 3, member A2	0,9406	1,9193	0,0000	0,0000
ENSG00000111801	BTN3A3	butyrophilin, subfamily 3, member A3	1,3740	2,5918	0,0000	0,0000
ENSG00000005379	BZRAP1	benzodiazepine receptor (peripheral) associated protein 1	0,9743	1,9647	0,0000	0,0000
ENSG00000265148	BZRAP1-AS1	BZRAP1 antisense RNA 1	0,8624	1,8181	0,0000	0,0001
ENSG00000082153	BZW1	basic leucine zipper and W2 domains 1	-0,6400	0,6417	0,0000	0,0000
ENSG00000136261	BZW2	basic leucine zipper and W2 domains 2	-0,4394	0,7375	0,0000	0,0002
ENSG00000171224	C10orf35	chromosome 10 open reading frame 35	0,4283	1,3456	0,0002	0,0026
ENSG00000107738	C10orf54	chromosome 10 open reading frame 54	0,4221	1,3399	0,0008	0,0080
ENSG00000165863	C10orf82	chromosome 10 open reading frame 82	1,1893	2,2804	0,0000	0,0000
ENSG00000211450	C11orf31	chromosome 11 open reading frame 31	0,3832	1,3043	0,0001	0,0012
ENSG00000182919	C11orf54	chromosome 11 open reading frame 54	0,4750	1,3899	0,0000	0,0000
ENSG00000175573	C11orf68	chromosome 11 open reading frame 68	0,7047	1,6298	0,0000	0,0000
ENSG00000180425	C11orf71	chromosome 11 open reading frame 71	0,8895	1,8525	0,0000	0,0000
ENSG00000170270	C14orf142	chromosome 14 open reading frame 142	-0,5738	0,6718	0,0000	0,0000
ENSG00000170468	C14orf169	chromosome 14 open reading frame 169	-0,6213	0,6501	0,0000	0,0000
ENSG00000179476	C14orf28	chromosome 14 open reading frame 28	0,5459	1,4599	0,0006	0,0062
ENSG00000185716	C16orf52	chromosome 16 open reading frame 52	-0,4463	0,7339	0,0000	0,0000
ENSG00000140688	C16orf58	chromosome 16 open reading frame 58	0,4253	1,3428	0,0001	0,0010
ENSG00000162062	C16orf59	chromosome 16 open reading frame 59	0,5804	1,4953	0,0000	0,0000
ENSG00000103544	C16orf62	chromosome 16 open reading frame 62	-0,3995	0,7581	0,0005	0,0048
ENSG00000154102	C16orf74	chromosome 16 open reading frame 74	0,6752	1,5968	0,0002	0,0029
ENSG00000273604	C17orf96	chromosome 17 open reading frame 96	-0,3970	0,7594	0,0000	0,0002

ENSG00000130813	C19orf66	chromosome 19 open reading frame 66	0,4467	1,3629	0,0005	0,0055
ENSG00000174917	C19orf70	chromosome 19 open reading frame 70	0,4638	1,3792	0,0002	0,0027
ENSG00000163362	C1orf106	chromosome 1 open reading frame 106	-0,6154	0,6528	0,0000	0,0000
ENSG00000253313	C1orf210	chromosome 1 open reading frame 210	0,8874	1,8498	0,0000	0,0000
ENSG00000142686	C1orf216	chromosome 1 open reading frame 216	-0,5164	0,6991	0,0000	0,0000
ENSG00000133466	C1QTNF6	C1q and tumor necrosis factor related protein 6	0,5641	1,4784	0,0000	0,0002
ENSG00000205861	C1QTNF9B-AS1	C1QTNF9B antisense RNA 1	-0,8770	0,5445	0,0000	0,0000
ENSG00000174403	C20orf166-AS1	C20orf166 antisense RNA 1	1,2399	2,3618	0,0000	0,0001
ENSG00000125531	C20orf195	chromosome 20 open reading frame 195	0,6816	1,6039	0,0000	0,0006
ENSG00000101084	C20orf24	chromosome 20 open reading frame 24	-0,4194	0,7478	0,0001	0,0018
ENSG00000160298	C21orf58	chromosome 21 open reading frame 58	0,4680	1,3832	0,0000	0,0004
ENSG00000172478	C2orf54	chromosome 2 open reading frame 54	0,5141	1,4281	0,0000	0,0002
ENSG00000168887	C2orf68	chromosome 2 open reading frame 68	0,4171	1,3352	0,0000	0,0000
ENSG00000187833	C2orf78	chromosome 2 open reading frame 78	0,6734	1,5948	0,0001	0,0009
ENSG00000159239	C2orf81	chromosome 2 open reading frame 81	0,6860	1,6088	0,0000	0,0000
ENSG00000125730	C3	complement component 3	-1,1451	0,4522	0,0000	0,0000
ENSG00000088543	C3orf18	chromosome 3 open reading frame 18	0,4412	1,3577	0,0002	0,0022
ENSG00000114529	C3orf52	chromosome 3 open reading frame 52	-0,7652	0,5884	0,0000	0,0000
ENSG00000187068	C3orf70	chromosome 3 open reading frame 70	-1,3734	0,3860	0,0000	0,0000
ENSG00000174749	C4orf32	chromosome 4 open reading frame 32	-0,3804	0,7682	0,0003	0,0032
ENSG00000186577	C6orf1	chromosome 6 open reading frame 1	0,6895	1,6127	0,0000	0,0000
ENSG00000204542	C6orf15	chromosome 6 open reading frame 15	1,1432	2,2087	0,0000	0,0001
ENSG00000181577	C6orf223	chromosome 6 open reading frame 223	0,8645	1,8207	0,0000	0,0000
ENSG00000213563	C8orf82	chromosome 8 open reading frame 82	0,4499	1,3659	0,0006	0,0063
ENSG00000232434	C9orf172	chromosome 9 open reading frame 172	0,8424	1,7930	0,0000	0,0001
ENSG00000275549	C9orf173-AS1	C9orf173 antisense RNA 1	1,3190	2,4949	0,0000	0,0000
ENSG00000135045	C9orf40	chromosome 9 open reading frame 40	-0,4981	0,7080	0,0000	0,0005
ENSG00000165698	C9orf9	chromosome 9 open reading frame 9	0,7098	1,6356	0,0001	0,0014
ENSG00000063180	CA11	carbonic anhydrase XI	0,5885	1,5037	0,0000	0,0001
ENSG00000185015	CA13	carbonic anhydrase XIII	-0,8995	0,5361	0,0001	0,0017
ENSG00000107159	CA9	carbonic anhydrase IX	1,7022	3,2540	0,0000	0,0000

ENSG00000134508	CABLES1	Cdk5 and Abl enzyme substrate 1	0,7531	1,6854	0,0000	0,0000
ENSG00000149679	CABLES2	Cdk5 and Abl enzyme substrate 2	0,4027	1,3220	0,0001	0,0016
ENSG00000160325	CACFD1	calcium channel flower domain containing 1	1,2847	2,4363	0,0000	0,0000
ENSG00000157388	CACNA1D	calcium channel, voltage-dependent, L type, alpha 1D subunit	0,7027	1,6276	0,0000	0,0001
ENSG00000198216	CACNA1E	calcium channel, voltage-dependent, R type, alpha 1E subunit	2,0563	4,1591	0,0000	0,0000
ENSG00000102001	CACNA1F	calcium channel, voltage-dependent, L type, alpha 1F subunit	1,7595	3,3858	0,0000	0,0000
ENSG00000196557	CACNA1H	calcium channel, voltage-dependent, T type, alpha 1H subunit	1,3798	2,6022	0,0000	0,0000
ENSG00000100346	CACNA1I	calcium channel, voltage-dependent, T type, alpha 1I subunit	1,4546	2,7409	0,0000	0,0000
ENSG00000081248	CACNA1S	calcium channel, voltage-dependent, L type, alpha 1S subunit	0,7362	1,6657	0,0000	0,0000
ENSG00000151062	CACNA2D4	calcium channel, voltage-dependent, alpha 2/delta subunit 4	0,4109	1,3295	0,0000	0,0002
ENSG00000182389	CACNB4	calcium channel, voltage-dependent, beta 4 subunit	-0,5597	0,6785	0,0000	0,0000
ENSG00000130433	CACNG6	calcium channel, voltage-dependent, gamma subunit 6	0,6832	1,6057	0,0000	0,0000
ENSG00000142408	CACNG8	calcium channel, voltage-dependent, gamma subunit 8	-0,5849	0,6667	0,0001	0,0016
ENSG00000185933	CALHM1	calcium homeostasis modulator 1	1,8135	3,5149	0,0000	0,0000
ENSG00000138172	CALHM2	calcium homeostasis modulator 2	0,3789	1,3003	0,0006	0,0062
ENSG00000183128	CALHM3	calcium homeostasis modulator 3	0,8938	1,8581	0,0000	0,0000
ENSG00000198668	CALM1	calmodulin 1 (phosphorylase kinase, delta)	-0,6807	0,6239	0,0000	0,0000
ENSG00000143933	CALM2	calmodulin 2 (phosphorylase kinase, delta)	-0,5114	0,7015	0,0000	0,0000
ENSG00000152495	CAMK4	calcium/calmodulin-dependent protein kinase IV	1,7615	3,3905	0,0000	0,0000
ENSG00000004660	CAMKK1	calcium/calmodulin-dependent protein kinase kinase 1, alpha	0,8490	1,8013	0,0000	0,0000
ENSG00000108509	CAMTA2	calmodulin binding transcription activator 2	0,4614	1,3769	0,0000	0,0007
ENSG00000042493	CAPG	capping protein (actin filament), gelsolin-like	0,3793	1,3007	0,0004	0,0046
ENSG00000182472	CAPN12	calpain 12	0,5919	1,5072	0,0000	0,0001
ENSG00000162909	CAPN2	calpain 2, (m/II) large subunit	-0,5006	0,7068	0,0000	0,0000
ENSG00000116489	CAPZA1	capping protein (actin filament) muscle Z-line, alpha 1	-0,5077	0,7033	0,0000	0,0000
ENSG00000165233	CARD19	caspase recruitment domain family, member 19	0,5055	1,4196	0,0000	0,0002
ENSG00000105483	CARD8	caspase recruitment domain family, member 8	0,4592	1,3748	0,0000	0,0000
ENSG00000138380	CARF	calcium responsive transcription factor	0,3870	1,3077	0,0009	0,0089
ENSG00000213995	CARKD	carbohydrate kinase domain containing	-0,5667	0,6752	0,0000	0,0000
ENSG00000156017	CARNMT1	carnosine N-methyltransferase 1	-0,4077	0,7538	0,0000	0,0001
ENSG00000172508	CARNS1	carnosine synthase 1	1,6549	3,1489	0,0000	0,0000

ENSG00000110619	CARS	cysteinyl-tRNA synthetase	0,5797	1,4946	0,0000	0,0000
ENSG00000134905	CARS2	cysteinyl-tRNA synthetase 2, mitochondrial (putative)	-0,8979	0,5367	0,0000	0,0000
ENSG00000204682	CASC10	cancer susceptibility candidate 10	-0,5794	0,6692	0,0000	0,0001
ENSG00000167971	CASKIN1	CASK interacting protein 1	0,8877	1,8503	0,0005	0,0053
ENSG00000196954	CASP4	caspase 4, apoptosis-related cysteine peptidase	-1,2772	0,4126	0,0000	0,0000
ENSG00000121691	CAT	catalase	0,3959	1,3158	0,0000	0,0000
ENSG00000159231	CBR3	carbonyl reductase 3	-0,5523	0,6819	0,0000	0,0000
ENSG00000160200	CBS	cystathionine-beta-synthase	1,1315	2,1908	0,0000	0,0000
ENSG00000122565	CBX3	chromobox homolog 3	-0,5269	0,6940	0,0000	0,0000
ENSG00000141582	CBX4	chromobox homolog 4	0,3868	1,3075	0,0004	0,0043
ENSG00000100307	CBX7	chromobox homolog 7	0,7172	1,6440	0,0000	0,0000
ENSG00000060339	CCAR1	cell division cycle and apoptosis regulator 1	-0,4437	0,7353	0,0000	0,0000
ENSG00000171097	CCBL1	cysteine conjugate-beta lyase, cytoplasmic	0,8527	1,8058	0,0000	0,0000
ENSG00000159884	CCDC107	coiled-coil domain containing 107	0,5531	1,4672	0,0000	0,0000
ENSG00000168491	CCDC110	coiled-coil domain containing 110	0,5011	1,4153	0,0005	0,0055
ENSG00000103021	CCDC113	coiled-coil domain containing 113	0,9242	1,8976	0,0000	0,0000
ENSG00000176714	CCDC121	coiled-coil domain containing 121	0,9275	1,9020	0,0000	0,0000
ENSG00000104957	CCDC130	coiled-coil domain containing 130	0,4014	1,3207	0,0008	0,0078
ENSG00000205212	CCDC144NL	coiled-coil domain containing 144 family, N-terminal like	1,1809	2,2671	0,0000	0,0000
ENSG00000144395	CCDC150	coiled-coil domain containing 150	-0,5827	0,6677	0,0000	0,0002
ENSG00000248712	CCDC153	coiled-coil domain containing 153	0,9929	1,9901	0,0000	0,0000
ENSG00000120262	CCDC170	coiled-coil domain containing 170	0,6034	1,5193	0,0000	0,0000
ENSG00000122483	CCDC18	coiled-coil domain containing 18	-0,5146	0,7000	0,0000	0,0000
ENSG00000197816	CCDC180	coiled-coil domain containing 180	0,9032	1,8702	0,0010	0,0093
ENSG00000234409	CCDC188	coiled-coil domain containing 188	0,9928	1,9900	0,0002	0,0019
ENSG00000163617	CCDC191	coiled-coil domain containing 191	0,9651	1,9521	0,0000	0,0005
ENSG00000024862	CCDC28A	coiled-coil domain containing 28A	0,5547	1,4688	0,0000	0,0003
ENSG00000140481	CCDC33	coiled-coil domain containing 33	2,6405	6,2354	0,0000	0,0000
ENSG00000133773	CCDC59	coiled-coil domain containing 59	-0,4218	0,7465	0,0003	0,0039
ENSG00000108091	CCDC6	coiled-coil domain containing 6	-0,3910	0,7626	0,0000	0,0000
ENSG00000104983	CCDC61	coiled-coil domain containing 61	0,4423	1,3587	0,0008	0,0076

ENSG00000253276	CCDC71L	coiled-coil domain containing 71-like	-0,5501	0,6830	0,0000	0,0000
ENSG00000162004	CCDC78	coiled-coil domain containing 78	0,9120	1,8816	0,0000	0,0005
ENSG00000175602	CCDC85B	coiled-coil domain containing 85B	0,8803	1,8408	0,0001	0,0017
ENSG00000142039	CCDC97	coiled-coil domain containing 97	0,3790	1,3004	0,0001	0,0009
ENSG00000204536	CCHCR1	coiled-coil alpha-helical rod protein 1	0,5529	1,4670	0,0000	0,0000
ENSG00000106178	CCL24	chemokine (C-C motif) ligand 24	1,2291	2,3442	0,0000	0,0000
ENSG00000006606	CCL26	chemokine (C-C motif) ligand 26	1,1517	2,2217	0,0000	0,0000
ENSG00000151882	CCL28	chemokine (C-C motif) ligand 28	0,8661	1,8227	0,0000	0,0000
ENSG00000134057	CCNB1	cyclin B1	-0,7372	0,5999	0,0000	0,0000
ENSG00000157456	CCNB2	cyclin B2	-0,4405	0,7369	0,0001	0,0010
ENSG00000112237	CCNC	cyclin C	-0,6038	0,6580	0,0000	0,0000
ENSG00000118971	CCND2	cyclin D2	-0,6146	0,6531	0,0000	0,0000
ENSG00000105173	CCNE1	cyclin E1	-0,7977	0,5753	0,0000	0,0000
ENSG00000138764	CCNG2	cyclin G2	0,8574	1,8118	0,0000	0,0000
ENSG00000107443	CCNJ	cyclin J	-0,4160	0,7495	0,0000	0,0005
ENSG00000260916	CCPG1	cell cycle progression 1	0,6708	1,5919	0,0000	0,0000
ENSG00000173992	CCS	copper chaperone for superoxide dismutase	0,4084	1,3272	0,0001	0,0008
ENSG00000166226	CCT2	chaperonin containing TCP1, subunit 2 (beta)	-0,4564	0,7288	0,0000	0,0001
ENSG00000150753	CCT5	chaperonin containing TCP1, subunit 5 (epsilon)	-0,5386	0,6884	0,0000	0,0000
ENSG00000146731	CCT6A	chaperonin containing TCP1, subunit 6A (zeta 1)	-0,4239	0,7454	0,0000	0,0003
ENSG00000122674	CCZ1	CCZ1 homolog, vacuolar protein trafficking and biogenesis associated	-0,4810	0,7165	0,0000	0,0000
ENSG00000146574	CCZ1B	CCZ1 homolog B, vacuolar protein trafficking and biogenesis associated	-0,4912	0,7114	0,0000	0,0005
ENSG00000174950	CD164L2	CD164 sialomucin-like 2	1,1224	2,1771	0,0000	0,0001
ENSG00000177455	CD19	CD19 molecule	1,1835	2,2713	0,0000	0,0002
ENSG00000272398	CD24	CD24 molecule	-0,7318	0,6021	0,0000	0,0000
ENSG00000215039	CD27-AS1	CD27 antisense RNA 1	0,4117	1,3303	0,0002	0,0020
ENSG00000198087	CD2AP	CD2-associated protein	-0,3893	0,7635	0,0000	0,0002
ENSG00000117877	CD3EAP	CD3e molecule, epsilon associated protein	-0,8398	0,5587	0,0000	0,0000
ENSG00000169442	CD52	CD52 molecule	1,0017	2,0023	0,0000	0,0000
ENSG00000085063	CD59	CD59 molecule, complement regulatory protein	-0,5081	0,7031	0,0000	0,0000
ENSG00000125726	CD70	CD70 molecule	-0,7793	0,5827	0,0000	0,0000

ENSG00000085117	CD82	CD82 molecule	-0,5806	0,6687	0,0000	0,0000
ENSG00000112149	CD83	CD83 molecule	-0,9964	0,5013	0,0000	0,0000
ENSG00000172116	CD8B	CD8b molecule	0,7263	1,6543	0,0000	0,0000
ENSG00000254126	CD8BP	CD8b molecule pseudogene	0,8090	1,7520	0,0000	0,0000
ENSG00000158825	CDA	cytidine deaminase	0,7184	1,6453	0,0000	0,0000
ENSG00000140326	CDAN1	codanin 1	0,4209	1,3388	0,0000	0,0005
ENSG00000151465	CDC123	cell division cycle 123	-0,4308	0,7419	0,0000	0,0003
ENSG00000130177	CDC16	cell division cycle 16	-0,8897	0,5397	0,0000	0,0000
ENSG00000231007	CDC20P1	cell division cycle 20 pseudogene 1	-0,5749	0,6713	0,0000	0,0005
ENSG00000176386	CDC26	cell division cycle 26	-0,4756	0,7192	0,0000	0,0002
ENSG00000149798	CDC42EP2	CDC42 effector protein (Rho GTPase binding) 2	-0,4960	0,7091	0,0000	0,0006
ENSG00000234844	CDC42P2	cell division cycle 42 pseudogene 2	1,0990	2,1421	0,0001	0,0008
ENSG00000093009	CDC45	cell division cycle 45	0,4233	1,3410	0,0000	0,0000
ENSG00000039068	CDH1	cadherin 1, type 1	0,4603	1,3758	0,0000	0,0001
ENSG00000140937	CDH11	cadherin 11, type 2, OB-cadherin (osteoblast)	-1,9811	0,2533	0,0000	0,0000
ENSG00000128536	CDHR3	cadherin-related family member 3	-0,4827	0,7157	0,0000	0,0000
ENSG00000138395	CDK15	cyclin-dependent kinase 15	0,9761	1,9671	0,0000	0,0000
ENSG00000117266	CDK18	cyclin-dependent kinase 18	0,8507	1,8034	0,0000	0,0000
ENSG00000167797	CDK2AP2	cyclin-dependent kinase 2 associated protein 2	0,3947	1,3147	0,0005	0,0056
ENSG00000132964	CDK8	cyclin-dependent kinase 8	-0,4119	0,7516	0,0000	0,0001
ENSG00000138769	CDKL2	cyclin-dependent kinase-like 2 (CDC2-related kinase)	1,1252	2,1814	0,0000	0,0000
ENSG00000129757	CDKN1C	cyclin-dependent kinase inhibitor 1C (p57, Kip2)	1,0975	2,1398	0,0000	0,0000
ENSG00000147883	CDKN2B	cyclin-dependent kinase inhibitor 2B (p15, inhibits CDK4)	0,6413	1,5597	0,0000	0,0000
ENSG00000129355	CDKN2D	cyclin-dependent kinase inhibitor 2D (p19, inhibits CDK4)	0,4764	1,3912	0,0001	0,0008
ENSG00000100526	CDKN3	cyclin-dependent kinase inhibitor 3	-0,8332	0,5613	0,0000	0,0000
ENSG00000185267	CDNF	cerebral dopamine neurotrophic factor	0,8140	1,7581	0,0011	0,0099
ENSG00000091527	CDV3	CDV3 homolog (mouse)	-0,5754	0,6711	0,0000	0,0000
ENSG00000165556	CDX2	caudal type homeobox 2	-0,6702	0,6284	0,0000	0,0000
ENSG00000079385	CEACAM1	carcinoembryonic antigen-related cell adhesion molecule 1 (biliary glycoprotein)	0,9185	1,8902	0,0000	0,0000
ENSG00000186567	CEACAM19	carcinoembryonic antigen-related cell adhesion molecule 19	0,6772	1,5990	0,0000	0,0001
ENSG00000007129	CEACAM21	carcinoembryonic antigen-related cell adhesion molecule 21	1,3078	2,4757	0,0000	0,0000

ENSG00000172216	CEBPB	CCAAT/enhancer binding protein (C/EBP), beta	0,7784	1,7152	0,0000	0,0000
ENSG00000277449	CEBPB-AS1	CEBPB antisense RNA 1	0,8720	1,8301	0,0000	0,0002
ENSG00000115816	CEBPZ	CCAAT/enhancer binding protein (C/EBP), zeta	-0,4522	0,7309	0,0000	0,0001
ENSG00000103888	CEMIP	cell migration inducing protein, hyaluronan binding	0,4583	1,3739	0,0000	0,0000
ENSG00000138778	CENPE	centromere protein E, 312kDa	-0,5967	0,6613	0,0000	0,0000
ENSG00000117724	CENPF	centromere protein F, 350/400kDa	-0,5209	0,6969	0,0000	0,0000
ENSG00000151849	CENPJ	centromere protein J	-0,5343	0,6905	0,0000	0,0000
ENSG00000126001	CEP250	centrosomal protein 250kDa	0,4600	1,3756	0,0000	0,0001
ENSG00000106477	CEP41	centrosomal protein 41kDa	-0,4436	0,7353	0,0000	0,0003
ENSG00000138180	CEP55	centrosomal protein 55kDa	-0,5885	0,6650	0,0000	0,0000
ENSG00000101624	CEP76	centrosomal protein 76kDa	-0,5916	0,6636	0,0000	0,0000
ENSG00000173588	CEP83	centrosomal protein 83kDa	-0,6353	0,6438	0,0000	0,0000
ENSG00000167123	CERCAM	cerebral endothelial cell adhesion molecule	0,5031	1,4172	0,0000	0,0000
ENSG00000143418	CERS2	ceramide synthase 2	-0,4477	0,7332	0,0000	0,0000
ENSG00000090661	CERS4	ceramide synthase 4	0,8893	1,8523	0,0000	0,0000
ENSG00000172828	CES3	carboxylesterase 3	1,0140	2,0195	0,0000	0,0000
ENSG00000163885	CFAP100	cilia and flagella associated protein 100	1,0621	2,0880	0,0000	0,0003
ENSG00000213085	CFAP45	cilia and flagella associated protein 45	-0,6955	0,6175	0,0003	0,0033
ENSG00000142609	CFAP74	cilia and flagella associated protein 74	0,8486	1,8008	0,0004	0,0040
ENSG00000188523	CFAP77	cilia and flagella associated protein 77	1,1498	2,2188	0,0000	0,0000
ENSG00000165410	CFL2	cofilin 2 (muscle)	-0,4885	0,7128	0,0000	0,0000
ENSG00000143375	CGN	cingulin	-0,5905	0,6641	0,0000	0,0000
ENSG00000128965	CHAC1	ChaC glutathione-specific gamma-glutamylcyclotransferase 1	1,3993	2,6378	0,0000	0,0000
ENSG00000143942	CHAC2	ChaC, cation transport regulator homolog 2 (E. coli)	-0,5684	0,6744	0,0000	0,0000
ENSG00000198824	CHAMP1	chromosome alignment maintaining phosphoprotein 1	-1,0317	0,4891	0,0000	0,0000
ENSG00000163528	CHCHD4	coiled-coil-helix-coiled-coil-helix domain containing 4	-0,5425	0,6866	0,0000	0,0000
ENSG00000089199	CHGB	chromogranin B	-0,6051	0,6574	0,0011	0,0097
ENSG00000115561	CHMP3	charged multivesicular body protein 3	-0,4546	0,7297	0,0000	0,0001
ENSG00000106069	CHN2	chimerin 2	0,5499	1,4640	0,0000	0,0006
ENSG00000110172	CHORDC1	cysteine and histidine-rich domain (CHORD) containing 1	-0,5826	0,6678	0,0000	0,0000
ENSG00000135902	CHRND	cholinergic receptor, nicotinic, delta (muscle)	1,2924	2,4493	0,0000	0,0000

ENSG00000182022	CHST15	carbohydrate (N-acetylgalactosamine 4-sulfate 6-O) sulfotransferase 15	0,7041	1,6291	0,0000	0,0000
ENSG00000175040	CHST2	carbohydrate (N-acetylglucosamine-6-O) sulfotransferase 2	-0,7413	0,5982	0,0000	0,0004
ENSG00000127586	CHTF18	chromosome transmission fidelity factor 18	0,5507	1,4647	0,0002	0,0024
ENSG00000136425	CIB2	calcium and integrin binding family member 2	0,5655	1,4799	0,0010	0,0090
ENSG00000079432	CIC	capicua transcriptional repressor	0,4903	1,4047	0,0003	0,0037
ENSG00000198894	CIPC	CLOCK-interacting pacemaker	-0,4540	0,7300	0,0000	0,0000
ENSG00000141076	CIRH1A	cirrhosis, autosomal recessive 1A (cirhin)	-0,5731	0,6722	0,0000	0,0000
ENSG00000136108	CKAP2	cytoskeleton associated protein 2	-0,7366	0,6002	0,0000	0,0000
ENSG00000131730	CKMT2	creatine kinase, mitochondrial 2 (sarcomeric)	-0,3898	0,7632	0,0000	0,0003
ENSG00000247572	CKMT2-AS1	CKMT2 antisense RNA 1	0,5290	1,4429	0,0001	0,0010
ENSG00000109572	CLCN3	chloride channel, voltage-sensitive 3	-0,4641	0,7249	0,0000	0,0000
ENSG00000103249	CLCN7	chloride channel, voltage-sensitive 7	0,4945	1,4089	0,0002	0,0021
ENSG00000163347	CLDN1	claudin 1	0,4980	1,4122	0,0000	0,0000
ENSG00000165215	CLDN3	claudin 3	1,2247	2,3371	0,0000	0,0000
ENSG00000189143	CLDN4	claudin 4	0,4792	1,3940	0,0000	0,0005
ENSG00000181885	CLDN7	claudin 7	0,4009	1,3203	0,0000	0,0001
ENSG00000213937	CLDN9	claudin 9	0,7129	1,6391	0,0002	0,0022
ENSG00000113282	CLINT1	clathrin interactor 1	-0,3988	0,7585	0,0000	0,0001
ENSG00000106665	CLIP2	CAP-GLY domain containing linker protein 2	0,5332	1,4471	0,0000	0,0006
ENSG00000105270	CLIP3	CAP-GLY domain containing linker protein 3	0,9448	1,9250	0,0000	0,0000
ENSG00000115295	CLIP4	CAP-GLY domain containing linker protein family, member 4	0,9736	1,9637	0,0000	0,0000
ENSG00000165959	CLMN	calmin (calponin-like, transmembrane)	0,7366	1,6662	0,0000	0,0000
ENSG00000102805	CLN5	ceroid-lipofuscinosis, neuronal 5	-0,4180	0,7485	0,0006	0,0057
ENSG00000134852	CLOCK	clock circadian regulator	-0,4769	0,7185	0,0000	0,0000
ENSG00000172409	CLP1	cleavage and polyadenylation factor I subunit 1	-0,3812	0,7678	0,0000	0,0005
ENSG00000049656	CLPTM1L	CLPTM1-like	-0,4292	0,7427	0,0000	0,0000
ENSG00000158258	CLSTN2	calsyntenin 2	-0,4243	0,7452	0,0000	0,0000
ENSG00000141367	CLTC	clathrin, heavy chain (Hc)	-0,3945	0,7608	0,0000	0,0000
ENSG00000120885	CLU	clusterin	0,3995	1,3190	0,0000	0,0001
ENSG00000140931	CMTM3	CKLF-like MARVEL transmembrane domain containing 3	-0,5150	0,6998	0,0002	0,0023
ENSG00000091317	CMTM6	CKLF-like MARVEL transmembrane domain containing 6	-0,4196	0,7477	0,0000	0,0000

ENSG00000153721	CNKSR3	CNKSR family member 3	-0,4846	0,7147	0,0003	0,0035
ENSG00000148842	CNNM2	cyclin and CBS domain divalent metal cation transport mediator 2	0,7477	1,6791	0,0000	0,0000
ENSG00000146910	CNPY1	canopy FGF signaling regulator 1	-0,4306	0,7420	0,0001	0,0014
ENSG00000106714	CNTNAP3	contactin associated protein-like 3	-1,8209	0,2830	0,0000	0,0000
ENSG00000154529	CNTNAP3B	contactin associated protein-like 3B	-1,3056	0,4046	0,0000	0,0000
ENSG00000162377	COA7	cytochrome c oxidase assembly factor 7 (putative)	-0,6135	0,6536	0,0000	0,0000
ENSG00000136152	COG3	component of oligomeric golgi complex 3	-0,4046	0,7554	0,0000	0,0001
ENSG00000197467	COL13A1	collagen, type XIII, alpha 1	-0,3919	0,7621	0,0002	0,0027
ENSG00000196739	COL27A1	collagen, type XXVII, alpha 1	0,5994	1,5151	0,0000	0,0002
ENSG00000163359	COL6A3	collagen, type VI, alpha 3	-0,9660	0,5119	0,0000	0,0000
ENSG00000114270	COL7A1	collagen, type VII, alpha 1	0,5736	1,4882	0,0001	0,0015
ENSG00000171812	COL8A2	collagen, type VIII, alpha 2	1,3873	2,6159	0,0000	0,0000
ENSG00000093010	COMT	catechol-O-methyltransferase	0,4911	1,4055	0,0000	0,0006
ENSG00000106789	CORO2A	coronin, actin binding protein, 2A	0,5832	1,4981	0,0000	0,0000
ENSG00000103647	CORO2B	coronin, actin binding protein, 2B	-0,7590	0,5909	0,0000	0,0000
ENSG00000167549	CORO6	coronin 6	1,5209	2,8696	0,0000	0,0000
ENSG00000133983	COX16	COX16 cytochrome c oxidase assembly homolog	-0,4567	0,7286	0,0000	0,0005
ENSG00000160471	COX6B2	cytochrome c oxidase subunit VIb polypeptide 2 (testis)	0,6349	1,5529	0,0004	0,0041
ENSG00000128510	CPA4	carboxypeptidase A4	0,6502	1,5694	0,0004	0,0047
ENSG00000160111	CPAMD8	C3 and PZP-like, alpha-2-macroglobulin domain containing 8	0,8775	1,8372	0,0003	0,0033
ENSG00000107864	CPEB3	cytoplasmic polyadenylation element binding protein 3	0,7877	1,7263	0,0000	0,0001
ENSG00000145920	CPLX2	complexin 2	-1,2346	0,4250	0,0000	0,0000
ENSG00000178773	CPNE7	copine VII	0,4842	1,3988	0,0001	0,0013
ENSG00000139117	CPNE8	copine VIII	-1,9172	0,2648	0,0000	0,0000
ENSG00000021826	CPS1	carbamoyl-phosphate synthase 1, mitochondrial	1,0521	2,0736	0,0000	0,0000
ENSG00000214076	CPSF1P1	cleavage and polyadenylation specific factor 1, 160kDa pseudogene 1	0,9246	1,8981	0,0004	0,0043
ENSG00000111605	CPSF6	cleavage and polyadenylation specific factor 6, 68kDa	-0,3965	0,7597	0,0000	0,0000
ENSG00000088882	CPXM1	carboxypeptidase X (M14 family), member 1	0,8822	1,8432	0,0000	0,0000
ENSG00000121898	CPXM2	carboxypeptidase X (M14 family), member 2	0,8482	1,8003	0,0001	0,0014
ENSG00000177685	CRACR2B	calcium release activated channel regulator 2B	0,5617	1,4760	0,0000	0,0007
ENSG00000095321	CRAT	carnitine O-acetyltransferase	0,7735	1,7095	0,0000	0,0000

ENSG00000182158	CREB3L2	cAMP responsive element binding protein 3-like 2	0,5453	1,4593	0,0000	0,0000
ENSG00000143578	CREB3L4	cAMP responsive element binding protein 3-like 4	0,6602	1,5803	0,0000	0,0000
ENSG00000164463	CREBRF	CREB3 regulatory factor	0,8995	1,8654	0,0000	0,0000
ENSG00000175874	CREG2	cellular repressor of E1A-stimulated genes 2	1,1676	2,2463	0,0000	0,0000
ENSG00000163703	CRELD1	cysteine-rich with EGF-like domains 1	0,5578	1,4720	0,0001	0,0015
ENSG00000182809	CRIP2	cysteine-rich protein 2	1,2001	2,2976	0,0000	0,0000
ENSG00000167193	CRK	v-crk avian sarcoma virus CT10 oncogene homolog	-0,4322	0,7412	0,0000	0,0000
ENSG00000105662	CRTC1	CREB regulated transcription coactivator 1	0,8705	1,8283	0,0000	0,0000
ENSG00000008405	CRY1	cryptochrome circadian clock 1	-0,3829	0,7669	0,0000	0,0003
ENSG00000109846	CRYAB	crystallin, alpha B	0,9835	1,9773	0,0002	0,0028
ENSG00000165475	CRYL1	crystallin, lambda 1	0,5249	1,4388	0,0000	0,0000
ENSG00000062485	CS	citrate synthase	-0,3908	0,7627	0,0000	0,0004
ENSG00000124207	CSE1L	CSE1 chromosome segregation 1-like (yeast)	-0,4142	0,7504	0,0000	0,0003
ENSG00000182578	CSF1R	colony stimulating factor 1 receptor	1,0230	2,0322	0,0002	0,0022
ENSG00000147408	CSGALNACT1	chondroitin sulfate N-acetylgalactosaminyltransferase 1	0,7736	1,7095	0,0003	0,0029
ENSG00000173546	CSPG4	chondroitin sulfate proteoglycan 4	1,0843	2,1203	0,0000	0,0001
ENSG00000104218	CSPP1	centrosome and spindle pole associated protein 1	-0,3789	0,7690	0,0003	0,0032
ENSG00000159176	CSRP1	cysteine and glycine-rich protein 1	-0,5184	0,6982	0,0000	0,0000
ENSG00000175183	CSRP2	cysteine and glycine-rich protein 2	0,8680	1,8252	0,0000	0,0000
ENSG00000170373	CST1	cystatin SN	1,4750	2,7799	0,0000	0,0000
ENSG00000170369	CST2	cystatin SA	1,2618	2,3980	0,0000	0,0000
ENSG00000101441	CST4	cystatin S	1,2263	2,3396	0,0000	0,0001
ENSG00000144579	CTDSP1	CTD (carboxy-terminal domain, RNA polymerase II, polypeptide A) small phosphata	0,4803	1,3950	0,0000	0,0001
ENSG00000116761	CTH	cystathionine gamma-lyase	0,4153	1,3336	0,0003	0,0034
ENSG00000164932	CTHRC1	collagen triple helix repeat containing 1	-0,7022	0,6146	0,0000	0,0000
ENSG00000117984	CTSD	cathepsin D	0,4516	1,3675	0,0004	0,0041
ENSG00000103811	CTSH	cathepsin H	0,5798	1,4946	0,0002	0,0028
ENSG00000163131	CTSS	cathepsin S	-1,9192	0,2644	0,0000	0,0000
ENSG00000139842	CUL4A	cullin 4A	-0,7035	0,6141	0,0000	0,0000
ENSG00000044090	CUL7	cullin 7	0,6526	1,5720	0,0000	0,0000
ENSG00000112659	CUL9	cullin 9	0,9002	1,8663	0,0000	0,0000

ENSG00000145824	CXCL14	chemokine (C-X-C motif) ligand 14	-1,0055	0,4981	0,0000	0,0002
ENSG00000121966	CXCR4	chemokine (C-X-C motif) receptor 4	-0,5394	0,6880	0,0000	0,0000
ENSG00000147231	CXorf57	chromosome X open reading frame 57	1,2665	2,4058	0,0000	0,0000
ENSG00000167740	CYB5D2	cytochrome b5 domain containing 2	0,5247	1,4386	0,0000	0,0000
ENSG00000172115	CYCS	cytochrome c, somatic	-0,5865	0,6660	0,0000	0,0000
ENSG00000273749	CYFIP1	cytoplasmic FMR1 interacting protein 1	-0,4306	0,7420	0,0000	0,0000
ENSG00000055163	CYFIP2	cytoplasmic FMR1 interacting protein 2	-0,4039	0,7558	0,0000	0,0004
ENSG00000019186	CYP24A1	cytochrome P450, family 24, subfamily A, polypeptide 1	-0,7944	0,5766	0,0000	0,0000
ENSG00000095596	CYP26A1	cytochrome P450, family 26, subfamily A, polypeptide 1	1,0468	2,0660	0,0000	0,0000
ENSG00000186115	CYP4F2	cytochrome P450, family 4, subfamily F, polypeptide 2	0,9643	1,9512	0,0004	0,0047
ENSG00000186529	CYP4F3	cytochrome P450, family 4, subfamily F, polypeptide 3	1,5087	2,8454	0,0000	0,0000
ENSG00000145476	CYP4V2	cytochrome P450, family 4, subfamily V, polypeptide 2	-0,3842	0,7662	0,0003	0,0031
ENSG00000205795	CYS1	cystin 1	-0,5663	0,6754	0,0000	0,0001
ENSG00000146122	DAAM2	dishevelled associated activator of morphogenesis 2	1,5167	2,8613	0,0000	0,0000
ENSG00000136848	DAB2IP	DAB2 interacting protein	0,8024	1,7440	0,0000	0,0000
ENSG00000165617	DACT1	dishevelled-binding antagonist of beta-catenin 1	-0,5252	0,6949	0,0000	0,0004
ENSG00000226950	DANCR	differentiation antagonizing non-protein coding RNA	-0,4993	0,7075	0,0000	0,0001
ENSG00000112977	DAP	death-associated protein	-0,4921	0,7110	0,0000	0,0000
ENSG00000035664	DAPK2	death-associated protein kinase 2	1,0214	2,0299	0,0000	0,0002
ENSG00000006634	DBF4	DBF4 zinc finger	-0,6716	0,6278	0,0000	0,0000
ENSG00000123454	DBH	dopamine beta-hydroxylase (dopamine beta-monooxygenase)	1,5344	2,8967	0,0000	0,0000
ENSG00000225756	DBH-AS1	DBH antisense RNA 1	1,2984	2,4595	0,0000	0,0000
ENSG00000105516	DBP	D site of albumin promoter (albumin D-box) binding protein	1,4000	2,6391	0,0000	0,0000
ENSG00000164934	DCAF13	DDB1 and CUL4 associated factor 13	-0,5184	0,6981	0,0000	0,0000
ENSG00000132017	DCAF15	DDB1 and CUL4 associated factor 15	0,4126	1,3311	0,0006	0,0062
ENSG00000119599	DCAF4	DDB1 and CUL4 associated factor 4	-0,5693	0,6739	0,0000	0,0000
ENSG00000057019	DCBLD2	discoidin, CUB and LCCL domain containing 2	-0,7816	0,5817	0,0000	0,0000
ENSG00000133083	DCLK1	doublecortin-like kinase 1	-1,3891	0,3818	0,0000	0,0000
ENSG00000179958	DCTPP1	dCTP pyrophosphatase 1	-0,3832	0,7667	0,0001	0,0012
ENSG00000150401	DCUN1D2	DCN1, defective in cullin neddylation 1, domain containing 2	-0,5937	0,6626	0,0000	0,0000
ENSG00000188215	DCUN1D3	DCN1, defective in cullin neddylation 1, domain containing 3	-0,5372	0,6891	0,0000	0,0000

ENSG00000137692	DCUN1D5	DCN1, defective in cullin neddylation 1, domain containing 5	-0,4900	0,7120	0,0000	0,0001
ENSG00000153904	DDAH1	dimethylarginine dimethylaminohydrolase 1	-0,4365	0,7389	0,0000	0,0002
ENSG00000213722	DDAH2	dimethylarginine dimethylaminohydrolase 2	0,7495	1,6812	0,0000	0,0000
ENSG00000175197	DDIT3	DNA-damage-inducible transcript 3	1,3983	2,6359	0,0000	0,0000
ENSG00000168209	DDIT4	DNA-damage-inducible transcript 4	1,0638	2,0904	0,0000	0,0000
ENSG00000204580	DDR1	discoidin domain receptor tyrosine kinase 1	0,5532	1,4674	0,0000	0,0000
ENSG00000079785	DDX1	DEAD (Asp-Glu-Ala-Asp) box helicase 1	-0,4757	0,7191	0,0000	0,0000
ENSG00000178105	DDX10	DEAD (Asp-Glu-Ala-Asp) box polypeptide 10	-0,6292	0,6465	0,0000	0,0000
ENSG00000088205	DDX18	DEAD (Asp-Glu-Ala-Asp) box polypeptide 18	-0,4637	0,7251	0,0000	0,0000
ENSG00000064703	DDX20	DEAD (Asp-Glu-Ala-Asp) box polypeptide 20	-0,4118	0,7517	0,0002	0,0019
ENSG00000165732	DDX21	DEAD (Asp-Glu-Ala-Asp) box helicase 21	-0,7437	0,5972	0,0000	0,0000
ENSG00000215301	DDX3X	DEAD (Asp-Glu-Ala-Asp) box helicase 3, X-linked	-0,4353	0,7395	0,0000	0,0000
ENSG00000145833	DDX46	DEAD (Asp-Glu-Ala-Asp) box polypeptide 46	-0,4171	0,7489	0,0000	0,0000
ENSG00000108654	DDX5	DEAD (Asp-Glu-Ala-Asp) box helicase 5	-0,4501	0,7320	0,0000	0,0000
ENSG00000164822	DEFA6	defensin, alpha 6, Paneth cell-specific	1,0088	2,0123	0,0002	0,0021
ENSG00000198837	DENND4B	DENN/MADD domain containing 4B	0,4018	1,3212	0,0000	0,0004
ENSG00000170456	DENND5B	DENN/MADD domain containing 5B	0,4412	1,3578	0,0001	0,0008
ENSG00000035499	DEPDC1B	DEP domain containing 1B	-0,4014	0,7571	0,0000	0,0005
ENSG00000121690	DEPDC7	DEP domain containing 7	0,4412	1,3577	0,0000	0,0002
ENSG00000155792	DEPTOR	DEP domain containing MTOR-interacting protein	0,8635	1,8194	0,0002	0,0022
ENSG00000099958	DERL3	derlin 3	0,8379	1,7874	0,0000	0,0000
ENSG00000175084	DES	desmin	1,1240	2,1796	0,0000	0,0000
ENSG00000095397	DFNB31	deafness, autosomal recessive 31	0,7520	1,6841	0,0000	0,0006
ENSG00000185000	DGAT1	diacylglycerol O-acyltransferase 1	0,3849	1,3057	0,0009	0,0083
ENSG00000102780	DGKH	diacylglycerol kinase, eta	-0,6068	0,6567	0,0000	0,0000
ENSG00000116133	DHCR24	24-dehydrocholesterol reductase	-0,7513	0,5941	0,0000	0,0000
ENSG00000104808	DHDH	dihydrodiol dehydrogenase (dimeric)	0,9995	1,9993	0,0000	0,0000
ENSG00000167536	DHRS13	dehydrogenase/reductase (SDR family) member 13	0,5315	1,4455	0,0000	0,0005
ENSG00000100867	DHRS2	dehydrogenase/reductase (SDR family) member 2	-0,3917	0,7622	0,0001	0,0014
ENSG00000162496	DHRS3	dehydrogenase/reductase (SDR family) member 3	0,6807	1,6030	0,0000	0,0000
ENSG00000169084	DHRSX	dehydrogenase/reductase (SDR family) X-linked	-0,5161	0,6993	0,0000	0,0003

ENSG00000109606	DHX15	DEAH (Asp-Glu-Ala-His) box helicase 15	-0,4643	0,7248	0,0000	0,0000
ENSG00000135829	DHX9	DEAH (Asp-Glu-Ala-His) box helicase 9	-0,5045	0,7049	0,0000	0,0000
ENSG00000139734	DIAPH3	diaphanous-related formin 3	-0,5379	0,6888	0,0000	0,0000
ENSG00000100697	DICER1	dicer 1, ribonuclease type III	-0,4706	0,7217	0,0000	0,0000
ENSG00000235706	DICER1-AS1	DICER1 antisense RNA 1	0,5807	1,4956	0,0001	0,0008
ENSG00000165023	DIRAS2	DIRAS family, GTP-binding RAS-like 2	1,1778	2,2624	0,0000	0,0000
ENSG00000083520	DIS3	DIS3 homolog, exosome endoribonuclease and 3'-5' exoribonuclease	-0,8519	0,5540	0,0000	0,0000
ENSG00000150764	DIXDC1	DIX domain containing 1	0,5331	1,4471	0,0000	0,0001
ENSG00000130826	DKC1	dyskeratosis congenita 1, dyskerin	-0,6652	0,6306	0,0000	0,0000
ENSG00000050165	DKK3	dickkopf WNT signaling pathway inhibitor 3	1,2004	2,2981	0,0000	0,0000
ENSG00000104371	DKK4	dickkopf WNT signaling pathway inhibitor 4	1,2665	2,4057	0,0000	0,0000
ENSG00000164741	DLC1	DLC1 Rho GTPase activating protein	-0,5614	0,6777	0,0000	0,0000
ENSG00000176124	DLEU1	deleted in lymphocytic leukemia 1 (non-protein coding)	-0,8465	0,5561	0,0000	0,0000
ENSG00000231607	DLEU2	deleted in lymphocytic leukemia 2 (non-protein coding)	-0,8256	0,5643	0,0000	0,0000
ENSG00000075711	DLG1	discs, large homolog 1 (Drosophila)	-0,4733	0,7203	0,0000	0,0000
ENSG00000170579	DLGAP1	discs, large (Drosophila) homolog-associated protein 1	0,9236	1,8969	0,0005	0,0051
ENSG00000126787	DLGAP5	discs, large (Drosophila) homolog-associated protein 5	-0,5439	0,6859	0,0000	0,0000
ENSG00000128917	DLL4	delta-like 4 (Drosophila)	0,6792	1,6012	0,0008	0,0074
ENSG00000064195	DLX3	distal-less homeobox 3	0,9801	1,9726	0,0000	0,0000
ENSG00000108813	DLX4	distal-less homeobox 4	0,8424	1,7931	0,0000	0,0000
ENSG00000132837	DMGDH	dimethylglycine dehydrogenase	1,1116	2,1608	0,0000	0,0001
ENSG00000165506	DNAAF2	dynein, axonemal, assembly factor 2	-0,4512	0,7314	0,0000	0,0001
ENSG00000114841	DNAH1	dynein, axonemal, heavy chain 1	0,6791	1,6012	0,0000	0,0001
ENSG00000197653	DNAH10	dynein, axonemal, heavy chain 10	0,8414	1,7918	0,0005	0,0049
ENSG00000158486	DNAH3	dynein, axonemal, heavy chain 3	-0,8823	0,5425	0,0000	0,0000
ENSG00000086061	DNAJA1	DnaJ (Hsp40) homolog, subfamily A, member 1	-0,6202	0,6506	0,0000	0,0000
ENSG00000148719	DNAJB12	DnaJ (Hsp40) homolog, subfamily B, member 12	0,6347	1,5526	0,0000	0,0000
ENSG00000128590	DNAJB9	DnaJ (Hsp40) homolog, subfamily B, member 9	0,6496	1,5688	0,0000	0,0000
ENSG00000120675	DNAJC15	DnaJ (Hsp40) homolog, subfamily C, member 15	-0,5289	0,6931	0,0000	0,0000
ENSG00000105821	DNAJC2	DnaJ (Hsp40) homolog, subfamily C, member 2	-0,8995	0,5361	0,0000	0,0000
ENSG00000059769	DNAJC25	DnaJ (Hsp40) homolog, subfamily C , member 25	-0,4087	0,7533	0,0003	0,0038

ENSG00000115137	DNAJC27	DnaJ (Hsp40) homolog, subfamily C, member 27	0,4094	1,3281	0,0002	0,0028
ENSG00000102580	DNAJC3	DnaJ (Hsp40) homolog, subfamily C, member 3	-0,7331	0,6016	0,0000	0,0000
ENSG00000100246	DNAL4	dynein, axonemal, light chain 4	0,7568	1,6897	0,0000	0,0000
ENSG00000105612	DNASE2	deoxyribonuclease II, lysosomal	0,4931	1,4074	0,0000	0,0000
ENSG00000179532	DNHD1	dynein heavy chain domain 1	0,4344	1,3513	0,0009	0,0088
ENSG00000107554	DNMBP	dynamin binding protein	-0,5075	0,7035	0,0000	0,0000
ENSG00000067334	DNTTIP2	deoxynucleotidyltransferase, terminal, interacting protein 2	-0,4734	0,7203	0,0000	0,0000
ENSG00000149927	DOC2A	double C2-like domains, alpha	1,0402	2,0565	0,0000	0,0000
ENSG00000088538	DOCK3	dedicator of cytokinesis 3	0,4578	1,3734	0,0000	0,0002
ENSG00000130158	DOCK6	dedicator of cytokinesis 6	0,8858	1,8479	0,0000	0,0000
ENSG00000107099	DOCK8	dedicator of cytokinesis 8	-0,8140	0,5688	0,0000	0,0000
ENSG00000088387	DOCK9	dedicator of cytokinesis 9	-0,6298	0,6463	0,0000	0,0000
ENSG00000132768	DPH2	DPH2 homolog	-0,5357	0,6898	0,0000	0,0000
ENSG00000154813	DPH3	diphthamide biosynthesis 3	-0,4660	0,7240	0,0000	0,0000
ENSG00000156162	DPY19L4	dpy-19-like 4 (C. elegans)	-0,4270	0,7438	0,0000	0,0000
ENSG00000113657	DPYSL3	dihydropyrimidinase-like 3	1,2044	2,3043	0,0000	0,0000
ENSG00000151640	DPYSL4	dihydropyrimidinase-like 4	0,9219	1,8946	0,0008	0,0075
ENSG00000134755	DSC2	desmocollin 2	0,6320	1,5497	0,0001	0,0010
ENSG00000138101	DTNB	dystrobrevin, beta	0,4567	1,3724	0,0000	0,0001
ENSG00000178498	DTX3	deltex 3, E3 ubiquitin ligase	0,4003	1,3198	0,0000	0,0001
ENSG00000110042	DTX4	deltex 4, E3 ubiquitin ligase	-0,6408	0,6414	0,0000	0,0000
ENSG00000105865	DUS4L	dihydrouridine synthase 4-like	-0,5127	0,7009	0,0000	0,0000
ENSG00000120129	DUSP1	dual specificity phosphatase 1	0,5001	1,4143	0,0001	0,0011
ENSG00000079393	DUSP13	dual specificity phosphatase 13	1,7767	3,4263	0,0000	0,0000
ENSG00000139318	DUSP6	dual specificity phosphatase 6	-0,4084	0,7535	0,0000	0,0000
ENSG00000184545	DUSP8	dual specificity phosphatase 8	0,6386	1,5569	0,0008	0,0080
ENSG00000004975	DVL2	dishevelled segment polarity protein 2	0,5375	1,4514	0,0000	0,0000
ENSG00000138036	DYNC2LI1	dynein, cytoplasmic 2, light intermediate chain 1	0,7066	1,6320	0,0000	0,0000
ENSG00000088986	DYNLL1	dynein, light chain, LC8-type 1	-0,4995	0,7074	0,0000	0,0001
ENSG00000264364	DYNLL2	dynein, light chain, LC8-type 2	-0,4047	0,7554	0,0000	0,0000
ENSG00000105204	DYRK1B	dual-specificity tyrosine-(Y)-phosphorylation regulated kinase 1B	1,3801	2,6028	0,0000	0,0000

ENSG00000010219	DYRK4	dual-specificity tyrosine-(Y)-phosphorylation regulated kinase 4	0,4859	1,4004	0,0000	0,0000
ENSG00000135636	DYSF	dysferlin	1,5023	2,8329	0,0000	0,0000
ENSG00000112242	E2F3	E2F transcription factor 3	-0,4284	0,7431	0,0000	0,0000
ENSG00000133740	E2F5	E2F transcription factor 5, p130-binding	-0,7063	0,6129	0,0000	0,0000
ENSG00000088881	EBF4	early B-cell factor 4	0,6204	1,5373	0,0000	0,0005
ENSG00000117395	EBNA1BP2	EBNA1 binding protein 2	-0,4071	0,7542	0,0001	0,0018
ENSG00000171551	ECEL1	endothelin converting enzyme-like 1	0,7160	1,6426	0,0007	0,0068
ENSG00000093144	ECHDC1	ethylmalonyl-CoA decarboxylase 1	-0,5783	0,6698	0,0000	0,0000
ENSG00000167969	ECI1	enoyl-CoA delta isomerase 1	0,4590	1,3746	0,0001	0,0012
ENSG00000143369	ECM1	extracellular matrix protein 1	0,7873	1,7259	0,0000	0,0000
ENSG00000114346	ECT2	epithelial cell transforming 2	-0,5151	0,6998	0,0000	0,0000
ENSG00000158813	EDA	ectodysplasin A	0,4291	1,3464	0,0000	0,0003
ENSG00000131080	EDA2R	ectodysplasin A2 receptor	-0,7320	0,6021	0,0001	0,0011
ENSG00000078401	EDN1	endothelin 1	-1,1616	0,4470	0,0000	0,0000
ENSG00000127129	EDN2	endothelin 2	1,0398	2,0560	0,0000	0,0000
ENSG00000124205	EDN3	endothelin 3	0,9389	1,9171	0,0000	0,0003
ENSG00000124802	EEF1E1	eukaryotic translation elongation factor 1 epsilon 1	-0,4538	0,7301	0,0001	0,0017
ENSG00000118894	EEF2KMT	eukaryotic elongation factor 2 lysine methyltransferase	-0,4172	0,7489	0,0004	0,0042
ENSG00000203965	EFCAB7	EF-hand calcium binding domain 7	0,7210	1,6483	0,0000	0,0000
ENSG00000115468	EFHD1	EF-hand domain family, member D1	0,9877	1,9831	0,0000	0,0007
ENSG00000142634	EFHD2	EF-hand domain family, member D2	-0,4998	0,7072	0,0000	0,0001
ENSG00000143590	EFNA3	ephrin-A3	0,7361	1,6657	0,0000	0,0000
ENSG00000090776	EFNB1	ephrin-B1	-0,4040	0,7558	0,0000	0,0003
ENSG00000084710	EFR3B	EFR3 homolog B	0,8433	1,7941	0,0000	0,0000
ENSG00000138798	EGF	epidermal growth factor	0,8466	1,7983	0,0007	0,0071
ENSG00000164318	EGFLAM	EGF-like, fibronectin type III and laminin G domains	0,9757	1,9666	0,0000	0,0002
ENSG00000146648	EGFR	epidermal growth factor receptor	-0,7226	0,6060	0,0000	0,0000
ENSG00000129521	EGLN3	egl-9 family hypoxia-inducible factor 3	0,7709	1,7063	0,0000	0,0000
ENSG00000110047	EHD1	EH-domain containing 1	-0,4927	0,7107	0,0000	0,0000
ENSG00000024422	EHD2	EH-domain containing 2	1,1640	2,2408	0,0000	0,0000
ENSG00000176401	EID2B	EP300 interacting inhibitor of differentiation 2B	0,4720	1,3870	0,0002	0,0020

ENSG00000173674	EIF1AX	eukaryotic translation initiation factor 1A, X-linked	-0,4907	0,7117	0,0000	0,0000
ENSG00000134001	EIF2S1	eukaryotic translation initiation factor 2, subunit 1 alpha, 35kDa	-0,3994	0,7582	0,0000	0,0007
ENSG00000106263	EIF3B	eukaryotic translation initiation factor 3, subunit B	-0,4452	0,7345	0,0000	0,0000
ENSG00000104131	EIF3J	eukaryotic translation initiation factor 3, subunit J	-0,6040	0,6579	0,0000	0,0000
ENSG00000151247	EIF4E	eukaryotic translation initiation factor 4E	-0,4843	0,7148	0,0000	0,0000
ENSG00000187840	EIF4EBP1	eukaryotic translation initiation factor 4E binding protein 1	0,7659	1,7004	0,0000	0,0000
ENSG00000148730	EIF4EBP2	eukaryotic translation initiation factor 4E binding protein 2	-0,3862	0,7652	0,0000	0,0000
ENSG00000110321	EIF4G2	eukaryotic translation initiation factor 4 gamma, 2	-0,6080	0,6561	0,0000	0,0000
ENSG00000100664	EIF5	eukaryotic translation initiation factor 5	-0,6311	0,6457	0,0000	0,0000
ENSG00000163577	EIF5A2	eukaryotic translation initiation factor 5A2	-0,4177	0,7486	0,0000	0,0001
ENSG00000158417	EIF5B	eukaryotic translation initiation factor 5B	-0,4307	0,7419	0,0000	0,0001
ENSG00000107105	ELAVL2	ELAV like neuron-specific RNA binding protein 2	1,2589	2,3932	0,0000	0,0000
ENSG00000163435	ELF3	E74-like factor 3 (ets domain transcription factor, epithelial-specific)	0,9271	1,9014	0,0000	0,0000
ENSG00000236081	ELFN1-AS1	ELFN1 antisense RNA 1	0,7177	1,6446	0,0000	0,0000
ENSG00000154920	EME1	essential meiotic structure-specific endonuclease 1	0,4566	1,3723	0,0000	0,0001
ENSG00000186998	EMID1	EMI domain containing 1	0,6806	1,6028	0,0002	0,0024
ENSG00000132205	EMILIN2	elastin microfibril interfacer 2	-0,4301	0,7422	0,0004	0,0047
ENSG00000134531	EMP1	epithelial membrane protein 1	-0,4201	0,7474	0,0000	0,0000
ENSG00000164778	EN2	engrailed homeobox 2	-0,4218	0,7465	0,0000	0,0000
ENSG00000171617	ENC1	ectodermal-neural cortex 1 (with BTB domain)	-0,9416	0,5207	0,0000	0,0000
ENSG00000167136	ENDOG	endonuclease G	0,3980	1,3177	0,0008	0,0081
ENSG00000167280	ENGASE	endo-beta-N-acetylglucosaminidase	0,5240	1,4380	0,0000	0,0004
ENSG00000124074	ENKD1	enkurin domain containing 1	0,9385	1,9165	0,0000	0,0000
ENSG00000111674	ENO2	enolase 2 (gamma, neuronal)	0,7263	1,6544	0,0000	0,0000
ENSG00000154269	ENPP3	ectonucleotide pyrophosphatase/phosphodiesterase 3	1,7730	3,4175	0,0000	0,0000
ENSG00000112796	ENPP5	ectonucleotide pyrophosphatase/phosphodiesterase 5 (putative)	0,8988	1,8645	0,0000	0,0000
ENSG00000188833	ENTPD8	ectonucleoside triphosphate diphosphohydrolase 8	1,3956	2,6310	0,0000	0,0000
ENSG00000163508	EOMES	eomesodermin	-0,8984	0,5365	0,0010	0,0091
ENSG00000142627	EPHA2	EPH receptor A2	-0,4753	0,7193	0,0000	0,0001
ENSG00000187266	EPOR	erythropoietin receptor	0,5741	1,4888	0,0000	0,0004
ENSG00000177106	EPS8L2	EPS8-like 2	0,4297	1,3470	0,0003	0,0035

ENSG00000133106	EPSTI1	epithelial stromal interaction 1 (breast)	-1,2994	0,4063	0,0000	0,0000
ENSG00000065361	ERBB3	erb-b2 receptor tyrosine kinase 3	0,5027	1,4169	0,0000	0,0000
ENSG00000187672	ERC2	ELKS/RAB6-interacting/CAST family member 2	-0,9791	0,5073	0,0000	0,0000
ENSG00000134899	ERCC5	excision repair cross-complementation group 5	-0,8057	0,5721	0,0000	0,0000
ENSG00000182150	ERCC6L2	excision repair cross-complementation group 6-like 2	-0,4220	0,7464	0,0000	0,0001
ENSG00000100632	ERH	enhancer of rudimentary homolog (Drosophila)	-0,5000	0,7071	0,0000	0,0001
ENSG00000099219	ERMP1	endoplasmic reticulum metallopeptidase 1	0,4830	1,3977	0,0000	0,0000
ENSG00000116285	ERRFI1	ERBB receptor feedback inhibitor 1	0,4971	1,4114	0,0000	0,0000
ENSG00000139684	ESD	esterase D	-0,4664	0,7238	0,0000	0,0001
ENSG00000089048	ESF1	ESF1 nucleolar pre-rRNA processing protein homolog	-0,3808	0,7680	0,0003	0,0038
ENSG00000135476	ESPL1	extra spindle pole bodies like 1, separase	0,5444	1,4584	0,0000	0,0000
ENSG00000187017	ESPN	espin	0,9773	1,9688	0,0000	0,0000
ENSG00000268869	ESPNP	espin pseudogene	0,9298	1,9050	0,0000	0,0001
ENSG00000104413	ESRP1	epithelial splicing regulatory protein 1	0,4368	1,3536	0,0000	0,0000
ENSG00000120705	ETF1	eukaryotic translation termination factor 1	-0,5639	0,6765	0,0000	0,0000
ENSG00000232757	ETF1P1	eukaryotic translation termination factor 1 pseudogene 1	-0,6902	0,6197	0,0000	0,0005
ENSG00000105379	ETFB	electron-transfer-flavoprotein, beta polypeptide	0,7562	1,6891	0,0000	0,0000
ENSG00000134954	ETS1	v-ets avian erythroblastosis virus E26 oncogene homolog 1	-1,0868	0,4708	0,0000	0,0000
ENSG00000006468	ETV1	ets variant 1	0,5889	1,5041	0,0000	0,0000
ENSG00000117036	ETV3	ets variant 3	-0,6699	0,6286	0,0000	0,0000
ENSG00000142459	EVI5L	ecotropic viral integration site 5-like	0,7096	1,6354	0,0000	0,0001
ENSG00000106038	EVX1	even-skipped homeobox 1	0,9537	1,9369	0,0005	0,0052
ENSG00000130713	EXOSC2	exosome component 2	-0,4049	0,7553	0,0000	0,0002
ENSG00000077348	EXOSC5	exosome component 5	0,4358	1,3527	0,0001	0,0014
ENSG00000120699	EXOSC8	exosome component 8	-0,5409	0,6874	0,0000	0,0000
ENSG00000182197	EXT1	exostosin glycosyltransferase 1	-0,3937	0,7612	0,0000	0,0006
ENSG00000108799	EZH1	enhancer of zeste 1 polycomb repressive complex 2 subunit	0,4192	1,3372	0,0000	0,0005
ENSG00000126218	F10	coagulation factor X	0,8454	1,7967	0,0000	0,0001
ENSG00000124491	F13A1	coagulation factor XIII, A1 polypeptide	-0,7523	0,5937	0,0000	0,0000
ENSG00000164251	F2RL1	coagulation factor II (thrombin) receptor-like 1	-0,4336	0,7404	0,0000	0,0002
ENSG00000103089	FA2H	fatty acid 2-hydroxylase	0,7984	1,7391	0,0000	0,0000

ENSG00000221968	FADS3	fatty acid desaturase 3	0,4604	1,3759	0,0000	0,0002
ENSG00000103876	FAH	fumarylacetoacetate hydrolase (fumarylacetoacetase)	-0,4250	0,7448	0,0000	0,0000
ENSG00000231584	FAHD2CP	fumarylacetoacetate hydrolase domain containing 2C, pseudogene	0,5623	1,4766	0,0000	0,0003
ENSG00000158234	FAIM	Fas apoptotic inhibitory molecule	-0,4760	0,7190	0,0000	0,0001
ENSG00000184731	FAM110C	family with sequence similarity 110, member C	-0,5607	0,6780	0,0000	0,0000
ENSG00000189057	FAM111B	family with sequence similarity 111, member B	-0,6348	0,6440	0,0000	0,0000
ENSG00000197712	FAM114A1	family with sequence similarity 114, member A1	0,7975	1,7381	0,0000	0,0000
ENSG00000184083	FAM120C	family with sequence similarity 120C	-0,9701	0,5105	0,0000	0,0000
ENSG00000122591	FAM126A	family with sequence similarity 126, member A	-0,4372	0,7386	0,0000	0,0000
ENSG00000175182	FAM131A	family with sequence similarity 131, member A	0,6454	1,5642	0,0000	0,0000
ENSG00000179083	FAM133A	family with sequence similarity 133, member A	-2,4324	0,1853	0,0000	0,0000
ENSG00000234545	FAM133B	family with sequence similarity 133, member B	-0,3812	0,7678	0,0001	0,0011
ENSG00000144567	FAM134A	family with sequence similarity 134, member A	0,3908	1,3111	0,0000	0,0000
ENSG00000154153	FAM134B	family with sequence similarity 134, member B	0,6609	1,5811	0,0000	0,0000
ENSG00000035141	FAM136A	family with sequence similarity 136, member A	-0,3794	0,7688	0,0001	0,0013
ENSG00000138640	FAM13A	family with sequence similarity 13, member A	1,4074	2,6526	0,0000	0,0000
ENSG00000031003	FAM13B	family with sequence similarity 13, member B	-0,4564	0,7288	0,0000	0,0003
ENSG00000164142	FAM160A1	family with sequence similarity 160, member A1	0,6253	1,5425	0,0000	0,0000
ENSG00000170264	FAM161A	family with sequence similarity 161, member A	0,4711	1,3861	0,0000	0,0003
ENSG00000114023	FAM162A	family with sequence similarity 162, member A	0,4830	1,3976	0,0000	0,0000
ENSG00000196990	FAM163B	family with sequence similarity 163, member B	0,9396	1,9180	0,0003	0,0029
ENSG00000150756	FAM173B	family with sequence similarity 173, member B	-0,4754	0,7193	0,0000	0,0001
ENSG00000174132	FAM174A	family with sequence similarity 174, member A	0,4370	1,3538	0,0008	0,0074
ENSG00000168754	FAM178B	family with sequence similarity 178, member B	0,6789	1,6009	0,0001	0,0015
ENSG00000198718	FAM179B	family with sequence similarity 179, member B	-0,4255	0,7446	0,0001	0,0014
ENSG00000108021	FAM208B	family with sequence similarity 208, member B	-0,6826	0,6230	0,0000	0,0000
ENSG00000177706	FAM20C	family with sequence similarity 20, member C	-0,6214	0,6500	0,0000	0,0000
ENSG00000047346	FAM214A	family with sequence similarity 214, member A	0,5180	1,4320	0,0000	0,0002
ENSG00000167930	FAM234A	family with sequence similarity 234, member A	0,4580	1,3737	0,0001	0,0011
ENSG00000071889	FAM3A	family with sequence similarity 3, member A	0,4368	1,3536	0,0003	0,0030
ENSG00000185112	FAM43A	family with sequence similarity 43, member A	-0,5805	0,6687	0,0000	0,0002

ENSG00000153310	FAM49B	family with sequence similarity 49, member B	-0,3919	0,7621	0,0001	0,0010
ENSG00000149926	FAM57B	family with sequence similarity 57, member B	0,9978	1,9970	0,0000	0,0000
ENSG00000165716	FAM69B	family with sequence similarity 69, member B	0,7975	1,7380	0,0000	0,0000
ENSG00000119812	FAM98A	family with sequence similarity 98, member A	-0,5497	0,6831	0,0000	0,0000
ENSG00000197601	FAR1	fatty acyl CoA reductase 1	-0,4097	0,7528	0,0000	0,0001
ENSG00000180178	FAR2P1	fatty acyl CoA reductase 2 pseudogene 1	1,0910	2,1302	0,0000	0,0004
ENSG00000026103	FAS	Fas cell surface death receptor	-0,5282	0,6934	0,0000	0,0000
ENSG00000164896	FASTK	Fas-activated serine/threonine kinase	0,4307	1,3479	0,0001	0,0011
ENSG00000124279	FASTKD3	FAST kinase domains 3	-0,6515	0,6366	0,0000	0,0000
ENSG00000165323	FAT3	FAT atypical cadherin 3	0,9068	1,8748	0,0009	0,0087
ENSG00000188878	FBF1	Fas (TNFRSF6) binding factor 1	0,8105	1,7539	0,0000	0,0001
ENSG00000142449	FBN3	fibrillin 3	0,7476	1,6790	0,0001	0,0017
ENSG00000165140	FBP1	fructose-1,6-bisphosphatase 1	-1,4098	0,3764	0,0000	0,0000
ENSG00000153558	FBXL2	F-box and leucine-rich repeat protein 2	0,7975	1,7381	0,0000	0,0000
ENSG00000108306	FBXL20	F-box and leucine-rich repeat protein 20	0,6052	1,5212	0,0000	0,0000
ENSG00000005812	FBXL3	F-box and leucine-rich repeat protein 3	-0,7708	0,5861	0,0000	0,0000
ENSG00000214050	FBXO16	F-box protein 16	0,6880	1,6110	0,0003	0,0030
ENSG00000116661	FBXO2	F-box protein 2	0,3951	1,3150	0,0001	0,0018
ENSG00000156804	FBXO32	F-box protein 32	1,7855	3,4474	0,0000	0,0000
ENSG00000153832	FBXO36	F-box protein 36	0,6438	1,5624	0,0000	0,0002
ENSG00000132879	FBXO44	F-box protein 44	0,4627	1,3782	0,0000	0,0005
ENSG00000177051	FBXO46	F-box protein 46	0,3891	1,3095	0,0008	0,0078
ENSG00000119616	FCF1	FCF1 rRNA-processing protein	-0,4848	0,7146	0,0000	0,0000
ENSG00000197948	FCHSD1	FCH and double SH3 domains 1	0,5972	1,5127	0,0000	0,0000
ENSG00000160752	FDPS	farnesyl diphosphate synthase	-0,4025	0,7565	0,0000	0,0000
ENSG00000088340	FER1L4	fer-1-like family member 4, pseudogene (functional)	1,0404	2,0568	0,0000	0,0000
ENSG00000101311	FERMT1	fermitin family member 1	-1,0348	0,4881	0,0000	0,0000
ENSG00000154783	FGD5	FYVE, RhoGEF and PH domain containing 5	0,6948	1,6187	0,0000	0,0000
ENSG00000129682	FGF13	fibroblast growth factor 13	-0,6679	0,6294	0,0000	0,0000
ENSG00000162344	FGF19	fibroblast growth factor 19	-0,4708	0,7216	0,0000	0,0000
ENSG00000105550	FGF21	fibroblast growth factor 21	0,9443	1,9243	0,0004	0,0046

ENSG00000102678	FGF9	fibroblast growth factor 9	-1,3241	0,3994	0,0000	0,0000
ENSG00000127418	FGFRL1	fibroblast growth factor receptor-like 1	0,9539	1,9371	0,0000	0,0000
ENSG00000091483	FH	fumarate hydratase	-0,4145	0,7503	0,0001	0,0017
ENSG00000022267	FHL1	four and a half LIM domains 1	-0,4521	0,7310	0,0000	0,0002
ENSG00000134775	FHOD3	formin homology 2 domain containing 3	0,7470	1,6783	0,0000	0,0000
ENSG00000213468	FIRRE	firre intergenic repeating RNA element	0,3996	1,3192	0,0008	0,0078
ENSG00000179431	FJX1	four jointed box 1	-0,5376	0,6889	0,0000	0,0000
ENSG00000106080	FKBP14	FK506 binding protein 14, 22 kDa	-0,3950	0,7605	0,0000	0,0002
ENSG00000088832	FKBP1A	FK506 binding protein 1A, 12kDa	-0,4015	0,7571	0,0001	0,0012
ENSG00000100442	FKBP3	FK506 binding protein 3, 25kDa	-0,4317	0,7414	0,0000	0,0001
ENSG00000105701	FKBP8	FK506 binding protein 8, 38kDa	0,4446	1,3610	0,0004	0,0047
ENSG00000176826	FKBP9P1	FK506 binding protein 9 pseudogene 1	1,2356	2,3547	0,0000	0,0000
ENSG00000154803	FLCN	folliculin	0,5435	1,4575	0,0000	0,0000
ENSG00000232774	FLJ22447	uncharacterized LOC400221	-1,1352	0,4553	0,0000	0,0000
ENSG00000137312	FLOT1	flotillin 1	0,6650	1,5855	0,0000	0,0000
ENSG00000162769	FLVCR1	feline leukemia virus subgroup C cellular receptor 1	-0,3849	0,7658	0,0003	0,0035
ENSG00000161791	FMNL3	formin-like 3	-0,4129	0,7511	0,0000	0,0000
ENSG00000167363	FN3K	fructosamine 3 kinase	0,5400	1,4539	0,0001	0,0012
ENSG00000115226	FNDC4	fibronectin type III domain containing 4	0,5447	1,4588	0,0000	0,0004
ENSG00000110195	FOLR1	folate receptor 1 (adult)	1,1444	2,2105	0,0000	0,0000
ENSG00000125740	FOSB	FBJ murine osteosarcoma viral oncogene homolog B	0,4331	1,3502	0,0005	0,0052
ENSG00000075426	FOSL2	FOS-like antigen 2	0,4533	1,3692	0,0000	0,0000
ENSG00000125798	FOXA2	forkhead box A2	0,8079	1,7507	0,0000	0,0000
ENSG00000137273	FOXF2	forkhead box F2	-0,4274	0,7436	0,0000	0,0007
ENSG00000176678	FOXL1	forkhead box L1	0,4866	1,4012	0,0000	0,0001
ENSG00000150907	FOXO1	forkhead box O1	-0,4234	0,7457	0,0004	0,0043
ENSG00000187474	FPR3	formyl peptide receptor 3	1,7430	3,3474	0,0000	0,0000
ENSG00000151474	FRMD4A	FERM domain containing 4A	0,6439	1,5625	0,0000	0,0000
ENSG00000114541	FRMD4B	FERM domain containing 4B	0,5751	1,4898	0,0001	0,0018
ENSG00000070601	FRMPD1	FERM and PDZ domain containing 1	1,4760	2,7818	0,0000	0,0000
ENSG00000137218	FRS3	fibroblast growth factor receptor substrate 3	0,8000	1,7411	0,0000	0,0001

ENSG00000075618	FSCN1	fascin actin-bundling protein 1	-0,6567	0,6343	0,0000	0,0000
ENSG00000186765	FSCN2	fascin actin-bundling protein 2, retinal	1,1572	2,2302	0,0000	0,0000
ENSG00000070404	FSTL3	follistatin-like 3 (secreted glycoprotein)	0,5587	1,4729	0,0000	0,0000
ENSG00000053108	FSTL4	follistatin-like 4	1,3565	2,5607	0,0000	0,0000
ENSG00000162613	FUBP1	far upstream element (FUSE) binding protein 1	-0,5214	0,6967	0,0000	0,0000
ENSG00000179163	FUCA1	fucosidase, alpha-L- 1, tissue	-0,6777	0,6252	0,0000	0,0000
ENSG00000069509	FUNDC1	FUN14 domain containing 1	-0,4122	0,7515	0,0001	0,0016
ENSG00000196968	FUT11	fucosyltransferase 11 (alpha (1,3) fucosyltransferase)	0,4747	1,3896	0,0000	0,0000
ENSG00000196371	FUT4	fucosyltransferase 4 (alpha (1,3) fucosyltransferase, myeloid-specific)	-0,4925	0,7108	0,0000	0,0000
ENSG00000165060	FXN	frataxin	-0,4557	0,7291	0,0002	0,0028
ENSG00000137726	FXYD6	FXYD domain containing ion transport regulator 6	1,1970	2,2925	0,0000	0,0000
ENSG00000157240	FZD1	frizzled class receptor 1	-0,5499	0,6831	0,0000	0,0000
ENSG00000145907	G3BP1	GTPase activating protein (SH3 domain) binding protein 1	-0,4952	0,7095	0,0000	0,0000
ENSG00000171298	GAA	glucosidase, alpha; acid	0,9361	1,9133	0,0000	0,0000
ENSG00000033327	GAB2	GRB2-associated binding protein 2	0,9095	1,8784	0,0000	0,0000
ENSG00000170296	GABARAP	GABA(A) receptor-associated protein	0,3918	1,3120	0,0001	0,0014
ENSG00000011677	GABRA3	gamma-aminobutyric acid (GABA) A receptor, alpha 3	-0,6393	0,6420	0,0000	0,0000
ENSG00000099860	GADD45B	growth arrest and DNA-damage-inducible, beta	0,5068	1,4209	0,0000	0,0000
ENSG00000128242	GAL3ST1	galactose-3-O-sulfotransferase 1	1,5793	2,9883	0,0000	0,0000
ENSG00000054983	GALC	galactosylceramidase	-0,6172	0,6519	0,0000	0,0006
ENSG00000108479	GALK1	galactokinase 1	0,7215	1,6489	0,0000	0,0000
ENSG00000164574	GALNT10	polypeptide N-acetylgalactosaminyltransferase 10	-1,1689	0,4448	0,0000	0,0000
ENSG00000109586	GALNT7	polypeptide N-acetylgalactosaminyltransferase 7	-0,5238	0,6955	0,0000	0,0000
ENSG00000130035	GALNT8	polypeptide N-acetylgalactosaminyltransferase 8	0,7949	1,7350	0,0000	0,0000
ENSG00000130005	GAMT	guanidinoacetate N-methyltransferase	0,6968	1,6209	0,0000	0,0000
ENSG00000109534	GAR1	GAR1 homolog, ribonucleoprotein	-0,5497	0,6832	0,0000	0,0000
ENSG00000141441	GAREM	GRB2 associated, regulator of MAPK1	0,9584	1,9432	0,0004	0,0042
ENSG00000272695	GAS6-AS2	GAS6 antisense RNA 2 (head to head)	-0,6791	0,6246	0,0001	0,0011
ENSG00000141013	GAS8	growth arrest-specific 8	0,6015	1,5173	0,0000	0,0000
ENSG00000179348	GATA2	GATA binding protein 2	0,4215	1,3393	0,0001	0,0010
ENSG00000239521	GATS	GATS, stromal antigen 3 opposite strand	0,4424	1,3589	0,0003	0,0034

ENSG00000117226	GBP3	guanylate binding protein 3	-0,4974	0,7084	0,0000	0,0002
ENSG00000100116	GCAT	glycine C-acetyltransferase	1,2749	2,4198	0,0000	0,0000
ENSG00000023909	GCLM	glutamate-cysteine ligase, modifier subunit	-0,5614	0,6776	0,0000	0,0000
ENSG00000104381	GDAP1	ganglioside induced differentiation associated protein 1	-0,6567	0,6343	0,0000	0,0000
ENSG00000135414	GDF11	growth differentiation factor 11	-0,5945	0,6623	0,0000	0,0000
ENSG00000130513	GDF15	growth differentiation factor 15	1,8589	3,6274	0,0000	0,0000
ENSG00000203879	GDI1	GDP dissociation inhibitor 1	0,4324	1,3495	0,0000	0,0000
ENSG00000057608	GDI2	GDP dissociation inhibitor 2	-0,4366	0,7389	0,0000	0,0001
ENSG00000164949	GEM	GTP binding protein overexpressed in skeletal muscle	0,5845	1,4995	0,0009	0,0089
ENSG00000082516	GEMIN5	gem (nuclear organelle) associated protein 5	-0,7106	0,6111	0,0000	0,0000
ENSG00000046647	GEMIN8	gem (nuclear organelle) associated protein 8	0,4766	1,3914	0,0000	0,0002
ENSG00000178295	GEN1	GEN1 Holliday junction 5' flap endonuclease	-0,3943	0,7608	0,0000	0,0003
ENSG00000168827	GFM1	G elongation factor, mitochondrial 1	-0,4722	0,7209	0,0000	0,0000
ENSG00000131459	GFPT2	glutamine-fructose-6-phosphate transaminase 2	1,3354	2,5235	0,0000	0,0000
ENSG00000265107	GJA5	gap junction protein, alpha 5, 40kDa	2,6456	6,2574	0,0000	0,0000
ENSG00000189433	GJB4	gap junction protein, beta 4, 30.3kDa	1,1273	2,1845	0,0000	0,0000
ENSG00000149328	GLB1L2	galactosidase, beta 1-like 2	-0,3998	0,7579	0,0000	0,0000
ENSG00000107249	GLIS3	GLIS family zinc finger 3	-0,7676	0,5874	0,0007	0,0067
ENSG00000108010	GLRX3	glutaredoxin 3	-0,4139	0,7506	0,0001	0,0012
ENSG00000115419	GLS	glutaminase	-0,7107	0,6110	0,0000	0,0000
ENSG00000250903	GMDS-AS1	GMDS antisense RNA 1 (head to head)	0,5766	1,4914	0,0007	0,0067
ENSG00000101216	GMEB2	glucocorticoid modulatory element binding protein 2	0,4260	1,3435	0,0001	0,0008
ENSG00000197045	GMFB	glia maturation factor, beta	-0,4107	0,7523	0,0000	0,0001
ENSG00000137198	GMPR	guanosine monophosphate reductase	0,3988	1,3184	0,0010	0,0094
ENSG00000127955	GNAI1	guanine nucleotide binding protein (G protein), alpha inhibiting activity polypeptide	1,4466	2,7256	0,0000	0,0000
ENSG00000087258	GNAO1	guanine nucleotide binding protein (G protein), alpha activating activity polypeptide	0,7707	1,7061	0,0000	0,0000
ENSG00000128266	GNAZ	guanine nucleotide binding protein (G protein), alpha z polypeptide	1,1058	2,1522	0,0000	0,0000
ENSG00000168243	GNG4	guanine nucleotide binding protein (G protein), gamma 4	-0,6763	0,6258	0,0000	0,0000
ENSG00000134697	GNL2	guanine nucleotide binding protein-like 2 (nucleolar)	-0,4256	0,7445	0,0000	0,0001
ENSG00000130119	GNL3L	guanine nucleotide binding protein-like 3 (nucleolar)-like	-0,4357	0,7393	0,0000	0,0001
ENSG00000115523	GNLY	granulysin	1,3417	2,5344	0,0000	0,0000

ENSG00000100522	GNPNAT1	glucosamine-phosphate N-acetyltransferase 1	-0,4448	0,7347	0,0000	0,0001
ENSG00000167110	GOLGA2	golgin A2	0,5157	1,4297	0,0000	0,0000
ENSG00000238105	GOLGA2P5	golgin A2 pseudogene 5	0,6593	1,5793	0,0000	0,0000
ENSG00000261649	GOLGA6L7P	golgin A6 family-like 7, pseudogene	1,0916	2,1311	0,0000	0,0000
ENSG00000232653	GOLGA8N	golgin A8 family, member N	0,7292	1,6577	0,0000	0,0003
ENSG00000173905	GOLIM4	golgi integral membrane protein 4	-0,4572	0,7284	0,0000	0,0000
ENSG00000135052	GOLM1	golgi membrane protein 1	-0,3939	0,7611	0,0000	0,0000
ENSG00000160818	GPATCH4	G patch domain containing 4	-0,7598	0,5906	0,0000	0,0000
ENSG00000152642	GPD1L	glycerol-3-phosphate dehydrogenase 1-like	-0,4980	0,7081	0,0000	0,0001
ENSG00000183671	GPR1	G protein-coupled receptor 1	0,6224	1,5394	0,0005	0,0055
ENSG00000101850	GPR143	G protein-coupled receptor 143	-0,8690	0,5475	0,0000	0,0000
ENSG00000164849	GPR146	G protein-coupled receptor 146	0,6090	1,5252	0,0008	0,0076
ENSG00000158292	GPR153	G protein-coupled receptor 153	0,3977	1,3174	0,0008	0,0075
ENSG00000173890	GPR160	G protein-coupled receptor 160	-0,6482	0,6381	0,0000	0,0001
ENSG00000184194	GPR173	G protein-coupled receptor 173	-0,9857	0,5050	0,0000	0,0000
ENSG00000152749	GPR180	G protein-coupled receptor 180	-0,8465	0,5561	0,0000	0,0000
ENSG00000135898	GPR55	G protein-coupled receptor 55	-0,4017	0,7570	0,0002	0,0023
ENSG00000013588	GPRC5A	G protein-coupled receptor, class C, group 5, member A	-0,4263	0,7442	0,0000	0,0000
ENSG00000167191	GPRC5B	G protein-coupled receptor, class C, group 5, member B	0,8741	1,8329	0,0000	0,0000
ENSG00000160360	GPSM1	G-protein signaling modulator 1	0,7845	1,7225	0,0000	0,0000
ENSG00000166123	GPT2	glutamic pyruvate transaminase (alanine aminotransferase) 2	0,6273	1,5447	0,0000	0,0000
ENSG00000176153	GPX2	glutathione peroxidase 2	1,2888	2,4432	0,0000	0,0000
ENSG00000155324	GRAMD3	GRAM domain containing 3	0,8626	1,8183	0,0000	0,0000
ENSG00000154016	GRAP	GRB2-related adaptor protein	-0,8349	0,5606	0,0000	0,0000
ENSG00000100351	GRAP2	GRB2-related adaptor protein 2	0,6927	1,6163	0,0001	0,0008
ENSG00000189152	GRAPL	GRB2-related adaptor protein-like	-1,0132	0,4954	0,0001	0,0017
ENSG00000141738	GRB7	growth factor receptor-bound protein 7	1,1313	2,1905	0,0000	0,0000
ENSG00000196208	GREB1	growth regulation by estrogen in breast cancer 1	0,8058	1,7482	0,0000	0,0000
ENSG00000180875	GREM2	gremlin 2, DAN family BMP antagonist	1,6495	3,1373	0,0000	0,0000
ENSG00000149403	GRIK4	glutamate receptor, ionotropic, kainate 4	-0,9989	0,5004	0,0000	0,0001
ENSG00000273079	GRIN2B	glutamate receptor, ionotropic, N-methyl D-aspartate 2B	-0,8184	0,5671	0,0010	0,0090

ENSG00000179603	GRM8	glutamate receptor, metabotropic 8	1,4784	2,7863	0,0000	0,0000
ENSG00000109519	GRPEL1	GrpE-like 1, mitochondrial (E. coli)	-0,4423	0,7360	0,0000	0,0002
ENSG00000132463	GRSF1	G-rich RNA sequence binding factor 1	-0,3805	0,7682	0,0002	0,0021
ENSG00000105447	GRWD1	glutamate-rich WD repeat containing 1	-0,4552	0,7294	0,0000	0,0000
ENSG00000167914	GSDMA	gasdermin A	0,7292	1,6577	0,0000	0,0003
ENSG00000073605	GSDMB	gasdermin B	0,4570	1,3727	0,0008	0,0079
ENSG00000104518	GSDMD	gasdermin D	0,4278	1,3452	0,0007	0,0071
ENSG00000131149	GSE1	Gse1 coiled-coil protein	0,6436	1,5622	0,0000	0,0000
ENSG00000100744	GSKIP	GSK3B interacting protein	-0,5189	0,6979	0,0000	0,0000
ENSG00000148180	GSN	gelsolin	0,4479	1,3640	0,0002	0,0029
ENSG00000103342	GSPT1	G1 to S phase transition 1	-0,4825	0,7157	0,0000	0,0000
ENSG00000148834	GSTO1	glutathione S-transferase omega 1	-0,4099	0,7527	0,0000	0,0002
ENSG00000065621	GSTO2	glutathione S-transferase omega 2	0,4542	1,3700	0,0001	0,0009
ENSG00000188342	GTF2F2	general transcription factor IIF, polypeptide 2, 30kDa	-0,6450	0,6395	0,0000	0,0000
ENSG00000110768	GTF2H1	general transcription factor IIH, polypeptide 1, 62kDa	-0,4229	0,7459	0,0000	0,0000
ENSG00000227038	GTF2IP7	general transcription factor IIi pseudogene 7	0,9529	1,9357	0,0000	0,0000
ENSG00000006704	GTF2IRD1	GTF2I repeat domain containing 1	0,5489	1,4630	0,0000	0,0000
ENSG00000122034	GTF3A	general transcription factor IIIA	-0,5841	0,6671	0,0000	0,0000
ENSG00000172432	GTPBP2	GTP binding protein 2	0,5898	1,5050	0,0000	0,0000
ENSG00000107937	GTPBP4	GTP binding protein 4	-0,4549	0,7296	0,0000	0,0000
ENSG00000236296	GUSBP5	glucuronidase, beta pseudogene 5	0,7773	1,7139	0,0004	0,0039
ENSG00000151233	GXYLT1	glucoside xylosyltransferase 1	-0,6056	0,6572	0,0000	0,0000
ENSG00000172986	GXYLT2	glucoside xylosyltransferase 2	0,6213	1,5383	0,0000	0,0000
ENSG00000165905	GYLTL1B	glycosyltransferase-like 1B	0,7490	1,6807	0,0000	0,0000
ENSG00000189060	H1F0	H1 histone family, member 0	0,6490	1,5680	0,0000	0,0000
ENSG00000184897	H1FX	H1 histone family, member X	0,8790	1,8392	0,0000	0,0000
ENSG00000246705	H2AFJ	H2A histone family, member J	0,4813	1,3960	0,0000	0,0005
ENSG00000113648	H2AFY	H2A histone family, member Y	-0,4153	0,7499	0,0000	0,0000
ENSG00000206527	HACD2	3-hydroxyacyl-CoA dehydratase 2	-0,4935	0,7103	0,0000	0,0000
ENSG00000063854	HAGH	hydroxyacylglutathione hydrolase	0,4744	1,3894	0,0001	0,0008
ENSG00000224189	HAGLR	HOXD antisense growth-associated long non-coding RNA	0,6729	1,5943	0,0000	0,0000

ENSG00000170445	HARS	histidyl-tRNA synthetase	-0,4195	0,7477	0,0000	0,0000
ENSG00000170961	HAS2	hyaluronan synthase 2	-0,3996	0,7581	0,0002	0,0022
ENSG00000249115	HAUS5	HAUS augmin-like complex, subunit 5	0,4911	1,4056	0,0000	0,0001
ENSG00000213931	HBE1	hemoglobin, epsilon 1	2,6605	6,3224	0,0000	0,0000
ENSG00000196565	HBG2	hemoglobin, gamma G	1,6407	3,1181	0,0000	0,0000
ENSG00000105856	HBP1	HMG-box transcription factor 1	0,6292	1,5467	0,0000	0,0000
ENSG00000103145	HCFC1R1	host cell factor C1 regulator 1 (XPO1 dependent)	1,2520	2,3818	0,0000	0,0000
ENSG00000231074	HCG18	HLA complex group 18 (non-protein coding)	-0,3937	0,7612	0,0000	0,0001
ENSG00000099822	HCN2	hyperpolarization activated cyclic nucleotide gated potassium channel 2	0,9226	1,8955	0,0000	0,0000
ENSG00000126264	HCST	hematopoietic cell signal transducer	0,6353	1,5533	0,0001	0,0011
ENSG00000163517	HDAC11	histone deacetylase 11	0,7557	1,6884	0,0000	0,0000
ENSG00000068024	HDAC4	histone deacetylase 4	0,5563	1,4705	0,0000	0,0000
ENSG00000108840	HDAC5	histone deacetylase 5	0,6125	1,5289	0,0000	0,0000
ENSG00000048052	HDAC9	histone deacetylase 9	1,0921	2,1319	0,0000	0,0000
ENSG00000111906	HDDC2	HD domain containing 2	-0,4086	0,7533	0,0000	0,0003
ENSG00000155393	HEATR3	HEAT repeat containing 3	-0,4176	0,7487	0,0000	0,0006
ENSG00000173064	HECTD4	HECT domain containing E3 ubiquitin protein ligase 4	0,4125	1,3310	0,0000	0,0004
ENSG00000114735	HEMK1	HemK methyltransferase family member 1	0,6550	1,5747	0,0000	0,0000
ENSG00000181333	HEPHL1	hephaestin-like 1	-0,7241	0,6054	0,0000	0,0000
ENSG00000276550	HERC2P2	hect domain and RLD 2 pseudogene 2	-0,4754	0,7193	0,0003	0,0038
ENSG00000206149	HERC2P9	hect domain and RLD 2 pseudogene 9	-0,6849	0,6220	0,0000	0,0001
ENSG00000138641	HERC3	HECT and RLD domain containing E3 ubiquitin protein ligase 3	0,4721	1,3871	0,0000	0,0001
ENSG00000144485	HES6	hes family bHLH transcription factor 6	0,4583	1,3740	0,0005	0,0051
ENSG00000186834	HEXIM1	hexamethylene bis-acetamide inducible 1	-0,5136	0,7005	0,0000	0,0000
ENSG00000135547	HEY2	hes-related family bHLH transcription factor with YRPW motif 2	-0,6007	0,6594	0,0000	0,0000
ENSG00000163909	HEYL	hes-related family bHLH transcription factor with YRPW motif-like	0,8468	1,7985	0,0000	0,0000
ENSG00000152804	HHEX	hematopoietically expressed homeobox	-0,4294	0,7426	0,0005	0,0054
ENSG00000114455	HHLA2	HERV-H LTR-associating 2	-0,7434	0,5973	0,0001	0,0010
ENSG00000167861	HID1	HID1 domain containing	1,0115	2,0161	0,0000	0,0000
ENSG00000100644	HIF1A	hypoxia inducible factor 1, alpha subunit (basic helix-loop-helix transcription factor	-0,5106	0,7019	0,0000	0,0000
ENSG00000258667	HIF1A-AS2	HIF1A antisense RNA 2	0,8998	1,8658	0,0001	0,0011

ENSG00000135245	HILPDA	hypoxia inducible lipid droplet-associated	0,6771	1,5989	0,0000	0,0000
ENSG00000196787	HIST1H2AG	histone cluster 1, H2ag	-0,6073	0,6564	0,0000	0,0004
ENSG00000124635	HIST1H2BJ	histone cluster 1, H2bj	-1,1017	0,4660	0,0000	0,0000
ENSG00000197903	HIST1H2BK	histone cluster 1, H2bk	-0,9026	0,5349	0,0000	0,0000
ENSG00000273983	HIST1H3G	histone cluster 1, H3g	-0,7553	0,5924	0,0001	0,0017
ENSG00000196890	HIST3H2BB	histone cluster 3, H2bb	0,6649	1,5855	0,0001	0,0018
ENSG00000156510	HKDC1	hexokinase domain containing 1	0,8652	1,8215	0,0000	0,0000
ENSG00000234745	HLA-B	major histocompatibility complex, class I, B	-0,4841	0,7150	0,0000	0,0000
ENSG00000242574	HLA-DMB	major histocompatibility complex, class II, DM beta	-0,7665	0,5878	0,0011	0,0097
ENSG00000231389	HLA-DPA1	major histocompatibility complex, class II, DP alpha 1	-1,1949	0,4368	0,0000	0,0002
ENSG00000204287	HLA-DRA	major histocompatibility complex, class II, DR alpha	-1,6292	0,3233	0,0000	0,0000
ENSG00000196126	HLA-DRB1	major histocompatibility complex, class II, DR beta 1	-0,8724	0,5462	0,0007	0,0068
ENSG00000230795	HLA-K	major histocompatibility complex, class I, K (pseudogene)	0,4402	1,3568	0,0009	0,0083
ENSG00000181126	HLA-V	major histocompatibility complex, class I, V (pseudogene)	0,7602	1,6937	0,0000	0,0004
ENSG00000233155	HMGA1P8	high mobility group AT-hook 1 pseudogene 8 (functional)	1,0134	2,0187	0,0002	0,0020
ENSG00000149948	HMGA2	high mobility group AT-hook 2	-1,0575	0,4805	0,0000	0,0000
ENSG00000189403	HMGB1	high mobility group box 1	-0,7789	0,5828	0,0000	0,0000
ENSG00000132967	HMGB1P5	high mobility group box 1 pseudogene 5	-0,7521	0,5937	0,0001	0,0008
ENSG00000259699	HMGB1P8	high mobility group box 1 pseudogene 8	-0,7890	0,5787	0,0000	0,0000
ENSG00000029993	HMGB3	high mobility group box 3	-0,6415	0,6410	0,0000	0,0000
ENSG00000117305	HMGCL	3-hydroxymethyl-3-methylglutaryl-CoA lyase	0,5801	1,4949	0,0000	0,0000
ENSG00000205581	HMGN1	high mobility group nucleosome binding domain 1	-0,4099	0,7527	0,0000	0,0003
ENSG00000198830	HMGN2	high mobility group nucleosomal binding domain 2	-0,6119	0,6543	0,0000	0,0000
ENSG00000212769	HMGN2P8	high mobility group nucleosomal binding domain 2 pseudogene 8	-0,5641	0,6764	0,0000	0,0002
ENSG00000072571	HMMR	hyaluronan-mediated motility receptor (RHAMM)	-0,5451	0,6853	0,0000	0,0000
ENSG00000101076	HNF4A	hepatocyte nuclear factor 4, alpha	-0,3909	0,7626	0,0002	0,0022
ENSG00000213412	HNRNPA1P33	heterogeneous nuclear ribonucleoprotein A1 pseudogene 33	-0,7849	0,5804	0,0004	0,0043
ENSG00000122566	HNRNPA2B1	heterogeneous nuclear ribonucleoprotein A2/B1	-0,5514	0,6823	0,0000	0,0000
ENSG00000197451	HNRNPAB	heterogeneous nuclear ribonucleoprotein A/B	-0,4410	0,7366	0,0000	0,0001
ENSG00000169045	HNRNPH1	heterogeneous nuclear ribonucleoprotein H1 (H)	-0,3882	0,7641	0,0000	0,0001
ENSG00000096746	HNRNPH3	heterogeneous nuclear ribonucleoprotein H3 (2H9)	-0,3800	0,7684	0,0000	0,0003

ENSG00000165119	HNRNPK	heterogeneous nuclear ribonucleoprotein K	-0,5143	0,7001	0,0000	0,0000
ENSG00000099783	HNRNPM	heterogeneous nuclear ribonucleoprotein M	-0,4129	0,7511	0,0000	0,0000
ENSG00000241935	HOGA1	4-hydroxy-2-oxoglutarate aldolase 1	1,2605	2,3959	0,0000	0,0000
ENSG00000152413	HOMER1	homer scaffolding protein 1	-0,5849	0,6667	0,0000	0,0000
ENSG00000103942	HOMER2	homer scaffolding protein 2	0,5199	1,4339	0,0004	0,0042
ENSG00000051128	HOMER3	homer scaffolding protein 3	0,6821	1,6044	0,0000	0,0000
ENSG00000233429	HOTAIRM1	HOXA transcript antisense RNA, myeloid-specific 1	-0,7699	0,5865	0,0000	0,0000
ENSG00000105991	HOXA1	homeobox A1	-1,0670	0,4773	0,0000	0,0000
ENSG00000005073	HOXA11	homeobox A11	-0,4455	0,7343	0,0000	0,0002
ENSG00000105996	HOXA2	homeobox A2	-0,7262	0,6045	0,0007	0,0066
ENSG00000037965	HOXC8	homeobox C8	-0,5118	0,7013	0,0000	0,0005
ENSG00000128710	HOXD10	homeobox D10	-0,4766	0,7187	0,0002	0,0020
ENSG00000128713	HOXD11	homeobox D11	-0,7173	0,6082	0,0001	0,0014
ENSG00000128714	HOXD13	homeobox D13	-1,0443	0,4849	0,0000	0,0000
ENSG00000175879	HOXD8	homeobox D8	0,5823	1,4973	0,0002	0,0021
ENSG00000116983	HPCAL4	hippocalcin like 4	0,7873	1,7258	0,0001	0,0008
ENSG00000164120	HPGD	hydroxyprostaglandin dehydrogenase 15-(NAD)	-0,8197	0,5666	0,0000	0,0000
ENSG00000132541	HRSP12	heat-responsive protein 12	-0,4230	0,7459	0,0000	0,0005
ENSG00000118960	HS1BP3	HCLS1 binding protein 3	0,6284	1,5458	0,0000	0,0000
ENSG00000231948	HS1BP3-IT1	HS1BP3 intronic transcript 1	-0,7024	0,6145	0,0000	0,0000
ENSG00000149084	HSD17B12	hydroxysteroid (17-beta) dehydrogenase 12	-0,4101	0,7526	0,0000	0,0000
ENSG00000204228	HSD17B8	hydroxysteroid (17-beta) dehydrogenase 8	0,5467	1,4607	0,0000	0,0001
ENSG00000103160	HSDL1	hydroxysteroid dehydrogenase like 1	0,4130	1,3314	0,0000	0,0001
ENSG00000102878	HSF4	heat shock transcription factor 4	0,6240	1,5411	0,0005	0,0050
ENSG00000196684	HSH2D	hematopoietic SH2 domain containing	0,4523	1,3682	0,0001	0,0010
ENSG00000080824	HSP90AA1	heat shock protein 90kDa alpha (cytosolic), class A member 1	-0,5448	0,6855	0,0000	0,0000
ENSG00000109971	HSPA8	heat shock 70kDa protein 8	-0,9530	0,5165	0,0000	0,0000
ENSG00000106211	HSPB1	heat shock 27kDa protein 1	0,4949	1,4092	0,0001	0,0014
ENSG00000144381	HSPD1	heat shock 60kDa protein 1 (chaperonin)	-0,4179	0,7485	0,0001	0,0008
ENSG00000120694	HSPH1	heat shock 105kDa/110kDa protein 1	-0,7949	0,5764	0,0000	0,0000
ENSG00000166033	HTRA1	HtrA serine peptidase 1	0,4717	1,3868	0,0002	0,0023

ENSG00000114378	HYAL1	hyaluronoglucosaminidase 1	0,4949	1,4092	0,0001	0,0012
ENSG00000186792	HYAL3	hyaluronoglucosaminidase 3	-0,6495	0,6375	0,0000	0,0001
ENSG00000090339	ICAM1	intercellular adhesion molecule 1	-0,4179	0,7485	0,0000	0,0000
ENSG00000115738	ID2	inhibitor of DNA binding 2, dominant negative helix-loop-helix protein	-0,4570	0,7285	0,0000	0,0001
ENSG00000235092	ID2-AS1	ID2 antisense RNA 1 (head to head)	-0,6102	0,6551	0,0007	0,0073
ENSG00000119912	IDE	insulin-degrading enzyme	-0,4874	0,7133	0,0000	0,0000
ENSG00000166411	IDH3A	isocitrate dehydrogenase 3 (NAD+) alpha	-0,4769	0,7185	0,0000	0,0000
ENSG00000067064	IDI1	isopentenyl-diphosphate delta isomerase 1	-0,4758	0,7190	0,0000	0,0000
ENSG00000169991	IFFO2	intermediate filament family orphan 2	-0,4644	0,7248	0,0000	0,0000
ENSG00000165949	IFI27	interferon, alpha-inducible protein 27	1,4982	2,8248	0,0000	0,0000
ENSG00000165948	IFI27L1	interferon, alpha-inducible protein 27-like 1	0,3831	1,3042	0,0003	0,0038
ENSG00000119632	IFI27L2	interferon, alpha-inducible protein 27-like 2	0,6052	1,5212	0,0000	0,0000
ENSG00000068079	IFI35	interferon-induced protein 35	0,6815	1,6038	0,0000	0,0000
ENSG00000126709	IFI6	interferon, alpha-inducible protein 6	0,7195	1,6466	0,0000	0,0000
ENSG00000142166	IFNAR1	interferon (alpha, beta and omega) receptor 1	-0,3833	0,7667	0,0000	0,0000
ENSG00000006652	IFRD1	interferon-related developmental regulator 1	-0,3914	0,7624	0,0000	0,0005
ENSG00000187535	IFT140	intraflagellar transport 140	0,8043	1,7463	0,0000	0,0000
ENSG00000138002	IFT172	intraflagellar transport 172	0,6022	1,5181	0,0000	0,0000
ENSG00000032742	IFT88	intraflagellar transport 88	-0,4337	0,7403	0,0000	0,0006
ENSG00000017427	IGF1	insulin-like growth factor 1 (somatomedin C)	1,3973	2,6340	0,0000	0,0000
ENSG00000140443	IGF1R	insulin-like growth factor 1 receptor	0,6509	1,5701	0,0000	0,0000
ENSG00000115457	IGFBP2	insulin-like growth factor binding protein 2, 36kDa	0,8443	1,7954	0,0000	0,0000
ENSG00000163453	IGFBP7	insulin-like growth factor binding protein 7	-0,4416	0,7363	0,0000	0,0001
ENSG00000204869	IGFL4	IGF-like family member 4	0,5750	1,4896	0,0000	0,0000
ENSG00000163395	IGFN1	immunoglobulin-like and fibronectin type III domain containing 1	0,9078	1,8762	0,0006	0,0063
ENSG00000162729	IGSF8	immunoglobulin superfamily, member 8	0,6849	1,6076	0,0000	0,0000
ENSG00000166130	IKBIP	IKBKB interacting protein	-0,4618	0,7261	0,0000	0,0000
ENSG00000263528	IKBKE	inhibitor of kappa light polypeptide gene enhancer in B-cells, kinase epsilon	-0,7462	0,5962	0,0000	0,0000
ENSG00000110324	IL10RA	interleukin 10 receptor, alpha	1,0647	2,0917	0,0000	0,0000
ENSG00000081985	IL12RB2	interleukin 12 receptor, beta 2	0,6698	1,5909	0,0006	0,0061
ENSG00000172349	IL16	interleukin 16	0,6416	1,5600	0,0000	0,0000

ENSG00000056736	IL17RB	interleukin 17 receptor B	0,6547	1,5743	0,0000	0,0000
ENSG00000163702	IL17RC	interleukin 17 receptor C	0,6696	1,5907	0,0000	0,0000
ENSG00000144730	IL17RD	interleukin 17 receptor D	-0,4884	0,7128	0,0000	0,0000
ENSG00000142224	IL19	interleukin 19	-0,8174	0,5675	0,0000	0,0005
ENSG00000196083	IL1RAP	interleukin 1 receptor accessory protein	-0,8202	0,5664	0,0000	0,0000
ENSG00000103522	IL21R	interleukin 21 receptor	1,9933	3,9813	0,0000	0,0000
ENSG00000162892	IL24	interleukin 24	0,4183	1,3364	0,0001	0,0012
ENSG00000008517	IL32	interleukin 32	-0,7753	0,5843	0,0000	0,0003
ENSG00000134352	IL6ST	interleukin 6 signal transducer	-0,7050	0,6134	0,0000	0,0000
ENSG00000145103	ILDR1	immunoglobulin-like domain containing receptor 1	0,6597	1,5797	0,0000	0,0000
ENSG00000143621	ILF2	interleukin enhancer binding factor 2	-0,3835	0,7666	0,0001	0,0015
ENSG00000184903	IMMP2L	inner mitochondrial membrane peptidase subunit 2	0,4809	1,3956	0,0007	0,0071
ENSG00000178035	IMPDH2	IMP (inosine 5'-monophosphate) dehydrogenase 2	0,4311	1,3482	0,0000	0,0000
ENSG00000259330	INAFM2	InaF-motif containing 2	-0,6596	0,6331	0,0000	0,0000
ENSG00000153487	ING1	inhibitor of growth family, member 1	-0,4963	0,7089	0,0000	0,0000
ENSG00000111653	ING4	inhibitor of growth family, member 4	0,7785	1,7153	0,0000	0,0000
ENSG00000123999	INHA	inhibin, alpha	0,5159	1,4299	0,0006	0,0061
ENSG00000163083	INHBB	inhibin, beta B	0,8206	1,7661	0,0000	0,0000
ENSG00000139269	INHBE	inhibin, beta E	2,0149	4,0417	0,0000	0,0000
ENSG00000151689	INPP1	inositol polyphosphate-1-phosphatase	-0,5939	0,6625	0,0000	0,0000
ENSG00000109452	INPP4B	inositol polyphosphate-4-phosphatase type II B	0,7158	1,6424	0,0000	0,0000
ENSG00000148384	INPP5E	inositol polyphosphate-5-phosphatase E	0,4350	1,3520	0,0000	0,0007
ENSG00000185133	INPP5J	inositol polyphosphate-5-phosphatase J	1,1211	2,1752	0,0000	0,0000
ENSG00000186480	INSIG1	insulin induced gene 1	0,4489	1,3650	0,0000	0,0000
ENSG00000171105	INSR	insulin receptor	0,3954	1,3153	0,0001	0,0010
ENSG00000185085	INTS5	integrator complex subunit 5	0,4734	1,3884	0,0000	0,0000
ENSG00000086200	IPO11	importin 11	-0,4175	0,7487	0,0000	0,0002
ENSG00000196497	IPO4	importin 4	-0,4572	0,7284	0,0006	0,0061
ENSG00000065150	IPO5	importin 5	-1,0791	0,4733	0,0000	0,0000
ENSG00000166578	IQCD	IQ motif containing D	0,8049	1,7471	0,0000	0,0006
ENSG00000103599	IQCH	IQ motif containing H	0,5758	1,4905	0,0007	0,0071

ENSG00000120645	IQSEC3	IQ motif and Sec7 domain 3	0,9142	1,8846	0,0003	0,0031
ENSG00000146243	IRAK1BP1	interleukin-1 receptor-associated kinase 1 binding protein 1	0,5779	1,4927	0,0000	0,0000
ENSG00000128604	IRF5	interferon regulatory factor 5	0,4979	1,4122	0,0000	0,0002
ENSG00000117595	IRF6	interferon regulatory factor 6	0,7027	1,6275	0,0000	0,0000
ENSG00000185507	IRF7	interferon regulatory factor 7	0,6191	1,5359	0,0001	0,0015
ENSG00000169047	IRS1	insulin receptor substrate 1	-0,3979	0,7590	0,0003	0,0034
ENSG00000185950	IRS2	insulin receptor substrate 2	-0,9762	0,5083	0,0000	0,0000
ENSG00000176842	IRX5	iroquois homeobox 5	0,8698	1,8274	0,0000	0,0000
ENSG00000143319	ISG20L2	interferon stimulated exonuclease gene 20kDa-like 2	-0,5136	0,7005	0,0000	0,0000
ENSG00000016082	ISL1	ISL LIM homeobox 1	-1,0922	0,4690	0,0000	0,0000
ENSG00000100593	ISM2	isthmin 2	0,6262	1,5435	0,0000	0,0000
ENSG00000143127	ITGA10	integrin, alpha 10	1,2484	2,3758	0,0000	0,0000
ENSG00000164171	ITGA2	integrin, alpha 2 (CD49B, alpha 2 subunit of VLA-2 receptor)	-0,5529	0,6817	0,0000	0,0000
ENSG00000161638	ITGA5	integrin, alpha 5 (fibronectin receptor, alpha polypeptide)	0,5860	1,5011	0,0000	0,0000
ENSG00000091409	ITGA6	integrin, alpha 6	-0,6145	0,6531	0,0000	0,0000
ENSG00000140678	ITGAX	integrin, alpha X (complement component 3 receptor 4 subunit)	1,0175	2,0245	0,0001	0,0011
ENSG00000147166	ITGB1BP2	integrin beta 1 binding protein (melusin) 2	-0,6688	0,6290	0,0002	0,0027
ENSG00000115221	ITGB6	integrin, beta 6	1,0503	2,0710	0,0000	0,0000
ENSG00000136156	ITM2B	integral membrane protein 2B	-0,4180	0,7484	0,0000	0,0002
ENSG00000123104	ITPR2	inositol 1,4,5-trisphosphate receptor, type 2	-0,6747	0,6265	0,0000	0,0000
ENSG00000099840	IZUMO4	IZUMO family member 4	1,1640	2,2408	0,0000	0,0000
ENSG00000101384	JAG1	jagged 1	-0,4288	0,7429	0,0000	0,0000
ENSG00000184916	JAG2	jagged 2	0,8444	1,7955	0,0000	0,0000
ENSG00000105639	JAK3	Janus kinase 3	0,6257	1,5430	0,0000	0,0001
ENSG00000280780	JAKMIP2-AS1	JAKMIP2 antisense RNA 1	0,9677	1,9558	0,0003	0,0035
ENSG00000188385	JAKMIP3	Janus kinase and microtubule interacting protein 3	1,1107	2,1595	0,0000	0,0000
ENSG00000168970	JMJD7-PLA2G4B	JMJD7-PLA2G4B readthrough	0,6922	1,6158	0,0001	0,0017
ENSG00000152409	JMY	junction mediating and regulatory protein, p53 cofactor	0,5110	1,4250	0,0000	0,0000
ENSG00000130522	JUND	jun D proto-oncogene	0,4942	1,4085	0,0000	0,0000
ENSG00000173801	JUP	junction plakoglobin	0,4398	1,3564	0,0001	0,0019
ENSG00000197256	KANK2	KN motif and ankyrin repeat domains 2	0,5316	1,4455	0,0000	0,0000

ENSG00000132854	KANK4	KN motif and ankyrin repeat domains 4	-0,9543	0,5161	0,0002	0,0029
ENSG00000144445	KANSL1L	KAT8 regulatory NSL complex subunit 1-like	0,4201	1,3380	0,0000	0,0006
ENSG00000165572	KBTBD6	kelch repeat and BTB (POZ) domain containing 6	-0,4741	0,7199	0,0000	0,0000
ENSG00000163376	KBTBD8	kelch repeat and BTB (POZ) domain containing 8	-0,9048	0,5341	0,0000	0,0000
ENSG00000069424	KCNAB2	potassium channel, voltage gated subfamily A regulatory beta subunit 2	1,3254	2,5060	0,0000	0,0000
ENSG00000116396	KCNC4	potassium channel, voltage gated Shaw related subfamily C, member 4	0,5051	1,4192	0,0000	0,0000
ENSG00000026559	KCNG1	potassium channel, voltage gated modifier subfamily G, member 1	0,6705	1,5916	0,0000	0,0001
ENSG00000055118	KCNH2	potassium channel, voltage gated eag related subfamily H, member 2	1,0624	2,0884	0,0000	0,0000
ENSG00000177807	KCNJ10	potassium channel, inwardly rectifying subfamily J, member 10	0,6878	1,6109	0,0000	0,0000
ENSG00000187486	KCNJ11	potassium channel, inwardly rectifying subfamily J, member 11	1,0039	2,0055	0,0000	0,0000
ENSG00000184185	KCNJ12	potassium channel, inwardly rectifying subfamily J, member 12	0,6162	1,5329	0,0000	0,0000
ENSG00000120457	KCNJ5	potassium channel, inwardly rectifying subfamily J, member 5	-0,4304	0,7421	0,0000	0,0004
ENSG00000100433	KCNK10	potassium channel, two pore domain subfamily K, member 10	1,0181	2,0252	0,0000	0,0005
ENSG00000171303	KCNK3	potassium channel, two pore domain subfamily K, member 3	0,7403	1,6705	0,0005	0,0049
ENSG00000099337	KCNK6	potassium channel, two pore domain subfamily K, member 6	-0,6149	0,6530	0,0000	0,0000
ENSG00000171121	KCNMB3	potassium channel subfamily M regulatory beta subunit 3	-0,7917	0,5777	0,0008	0,0075
ENSG00000105642	KCNN1	potassium channel, calcium activated intermediate/small conductance subfamily N	0,8823	1,8433	0,0006	0,0058
ENSG00000185760	KCNQ5	potassium channel, voltage gated KQT-like subfamily Q, member 5	0,9937	1,9913	0,0003	0,0032
ENSG00000170745	KCNS3	potassium voltage-gated channel, modifier subfamily S, member 3	0,5230	1,4370	0,0000	0,0000
ENSG00000178695	KCTD12	potassium channel tetramerization domain containing 12	-1,0463	0,4842	0,0000	0,0000
ENSG00000134901	KDELC1	KDEL (Lys-Asp-Glu-Leu) containing 1	-0,5748	0,6714	0,0002	0,0022
ENSG00000136240	KDELR2	KDEL (Lys-Asp-Glu-Leu) endoplasmic reticulum protein retention receptor 2	-0,4154	0,7498	0,0000	0,0001
ENSG00000066135	KDM4A	lysine (K)-specific demethylase 4A	0,3789	1,3004	0,0000	0,0001
ENSG00000127663	KDM4B	lysine (K)-specific demethylase 4B	0,5338	1,4477	0,0000	0,0004
ENSG00000132510	KDM6B	lysine (K)-specific demethylase 6B	0,5724	1,4870	0,0000	0,0000
ENSG00000131773	KHDRBS3	KH domain containing, RNA binding, signal transduction associated 3	-0,4240	0,7454	0,0002	0,0019
ENSG00000102445	KIAA0226L	KIAA0226-like	-0,6403	0,6416	0,0000	0,0000
ENSG00000100890	KIAA0391	KIAA0391	-0,6428	0,6405	0,0000	0,0000
ENSG00000166783	KIAA0430	KIAA0430	0,3883	1,3089	0,0000	0,0001
ENSG00000135709	KIAA0513	KIAA0513	0,7582	1,6914	0,0000	0,0002
ENSG00000069712	KIAA1107	KIAA1107	1,2222	2,3330	0,0000	0,0000

ENSG00000109265	KIAA1211	KIAA1211	0,4853	1,3999	0,0000	0,0004
ENSG00000134444	KIAA1468	KIAA1468	-0,4080	0,7537	0,0001	0,0012
ENSG00000163507	KIAA1524	KIAA1524	-0,4484	0,7328	0,0000	0,0000
ENSG00000110427	KIAA1549L	KIAA1549-like	-1,4684	0,3614	0,0000	0,0000
ENSG00000173214	KIAA1919	KIAA1919	-0,3916	0,7623	0,0009	0,0086
ENSG00000136883	KIF12	kinesin family member 12	0,7888	1,7276	0,0009	0,0081
ENSG00000197892	KIF13B	kinesin family member 13B	0,3894	1,3099	0,0001	0,0016
ENSG00000118193	KIF14	kinesin family member 14	-0,4372	0,7386	0,0000	0,0000
ENSG00000130294	KIF1A	kinesin family member 1A	0,5009	1,4151	0,0000	0,0003
ENSG00000138182	KIF20B	kinesin family member 20B	-0,5176	0,6985	0,0000	0,0000
ENSG00000186638	KIF24	kinesin family member 24	0,3894	1,3099	0,0000	0,0002
ENSG00000066735	KIF26A	kinesin family member 26A	0,7187	1,6457	0,0000	0,0005
ENSG00000084731	KIF3C	kinesin family member 3C	0,7699	1,7052	0,0000	0,0000
ENSG00000090889	KIF4A	kinesin family member 4A	-0,5830	0,6676	0,0000	0,0000
ENSG00000155980	KIF5A	kinesin family member 5A	0,8616	1,8170	0,0010	0,0095
ENSG00000167702	KIFC2	kinesin family member C2	0,5177	1,4317	0,0001	0,0011
ENSG00000140859	KIFC3	kinesin family member C3	-0,4130	0,7510	0,0007	0,0069
ENSG00000116014	KISS1R	KISS1 receptor	2,0113	4,0314	0,0000	0,0000
ENSG00000157404	KIT	v-kit Hardy-Zuckerman 4 feline sarcoma viral oncogene homolog	1,3402	2,5318	0,0000	0,0000
ENSG00000104892	KLC3	kinesin light chain 3	0,5871	1,5022	0,0002	0,0024
ENSG00000137171	KLC4	kinesin light chain 4	0,5766	1,4913	0,0000	0,0000
ENSG00000127528	KLF2	Kruppel-like factor 2	0,7418	1,6723	0,0003	0,0038
ENSG00000118263	KLF7	Kruppel-like factor 7 (ubiquitous)	0,8986	1,8642	0,0000	0,0000
ENSG00000130487	KLHDC7B	kelch domain containing 7B	1,6540	3,1471	0,0000	0,0000
ENSG00000003096	KLHL13	kelch-like family member 13	-0,9538	0,5163	0,0000	0,0007
ENSG00000099910	KLHL22	kelch-like family member 22	0,5206	1,4345	0,0000	0,0000
ENSG00000114796	KLHL24	kelch-like family member 24	1,0190	2,0265	0,0000	0,0000
ENSG00000087448	KLHL42	kelch-like family member 42	-0,5136	0,7005	0,0000	0,0000
ENSG00000167748	KLK1	kallikrein 1	0,4674	1,3826	0,0000	0,0001
ENSG00000169035	KLK7	kallikrein-related peptidase 7	1,3280	2,5105	0,0000	0,0000
ENSG00000182481	KPNA2	karyopherin alpha 2 (RAG cohort 1, importin alpha 1)	-0,4382	0,7380	0,0000	0,0001

ENSG00000102753	KPNA3	karyopherin alpha 3 (importin alpha 4)	-0,6941	0,6181	0,0000	0,0000
ENSG00000186432	KPNA4	karyopherin alpha 4 (importin alpha 3)	-0,5941	0,6625	0,0000	0,0000
ENSG00000184619	KRBA2	KRAB-A domain containing 2	0,8643	1,8205	0,0010	0,0094
ENSG00000183762	KREMEN1	kringle containing transmembrane protein 1	1,0806	2,1149	0,0000	0,0000
ENSG00000226145	KRT16P6	keratin 16 pseudogene 6	1,2013	2,2995	0,0000	0,0000
ENSG00000128422	KRT17	keratin 17, type I	-1,1967	0,4363	0,0000	0,0000
ENSG00000094796	KRT31	keratin 31, type I	0,9947	1,9926	0,0001	0,0013
ENSG00000171360	KRT38	keratin 38, type I	1,0283	2,0396	0,0002	0,0019
ENSG00000186081	KRT5	keratin 5, type II	-0,6905	0,6196	0,0000	0,0000
ENSG00000205420	KRT6A	keratin 6A, type II	-0,9534	0,5164	0,0000	0,0000
ENSG00000139648	KRT71	keratin 71, type II	-1,1752	0,4428	0,0000	0,0000
ENSG00000205426	KRT81	keratin 81, type II	1,2035	2,3030	0,0000	0,0000
ENSG00000170442	KRT86	keratin 86, type II	1,0528	2,0745	0,0000	0,0000
ENSG00000229320	KRT8P12	keratin 8 pseudogene 12	-0,4080	0,7537	0,0001	0,0017
ENSG00000212901	KRTAP3-1	keratin associated protein 3-1	0,8087	1,7517	0,0001	0,0013
ENSG00000126777	KTN1	kinectin 1 (kinesin receptor)	-0,6386	0,6424	0,0000	0,0000
ENSG00000126790	L3HYPDH	L-3-hydroxyproline dehydratase (trans-)	-0,4991	0,7076	0,0000	0,0002
ENSG00000179630	LACC1	laccase (multicopper oxidoreductase) domain containing 1	-0,7611	0,5900	0,0000	0,0000
ENSG00000159166	LAD1	ladinin 1	-0,5597	0,6784	0,0000	0,0000
ENSG00000196976	LAGE3	L antigen family, member 3	0,4336	1,3506	0,0000	0,0003
ENSG00000167618	LAIR2	leukocyte-associated immunoglobulin-like receptor 2	1,0624	2,0883	0,0001	0,0014
ENSG00000172037	LAMB2	laminin, beta 2 (laminin S)	0,4622	1,3776	0,0002	0,0024
ENSG00000196878	LAMB3	laminin, beta 3	0,4501	1,3661	0,0001	0,0014
ENSG00000185896	LAMP1	lysosomal-associated membrane protein 1	-0,8993	0,5361	0,0000	0,0000
ENSG00000078081	LAMP3	lysosomal-associated membrane protein 3	1,0368	2,0517	0,0000	0,0000
ENSG00000132434	LANCL2	LanC lantibiotic synthetase component C-like 2 (bacterial)	-0,4530	0,7305	0,0000	0,0001
ENSG00000162511	LAPTM5	lysosomal protein transmembrane 5	-0,4657	0,7241	0,0000	0,0005
ENSG00000161813	LARP4	La ribonucleoprotein domain family, member 4	-0,4167	0,7491	0,0000	0,0000
ENSG00000150457	LATS2	large tumor suppressor kinase 2	-0,6754	0,6262	0,0000	0,0000
ENSG00000143815	LBR	lamin B receptor	-0,7694	0,5867	0,0000	0,0000
ENSG00000157578	LCA5L	Leber congenital amaurosis 5-like	0,7497	1,6815	0,0000	0,0000

ENSG00000168806	LCMT2	leucine carboxyl methyltransferase 2	-0,3976	0,7591	0,0001	0,0014
ENSG00000184925	LCN12	lipocalin 12	1,3753	2,5942	0,0000	0,0000
ENSG00000148346	LCN2	lipocalin 2	-2,4442	0,1837	0,0000	0,0000
ENSG00000136167	LCP1	lymphocyte cytosolic protein 1 (L-plastin)	-0,6790	0,6246	0,0004	0,0043
ENSG00000043462	LCP2	lymphocyte cytosolic protein 2 (SH2 domain containing leukocyte protein of 76kDa	-1,1200	0,4601	0,0000	0,0006
ENSG00000198728	LDB1	LIM domain binding 1	0,3890	1,3094	0,0000	0,0001
ENSG00000166816	LDHD	lactate dehydrogenase D	0,9822	1,9754	0,0003	0,0037
ENSG00000203985	LDLRAD1	low density lipoprotein receptor class A domain containing 1	2,5875	6,0104	0,0000	0,0000
ENSG00000186007	LEMD1	LEM domain containing 1	0,9654	1,9526	0,0000	0,0000
ENSG00000226696	LENG8-AS1	LENG8 antisense RNA 1	0,6261	1,5433	0,0001	0,0009
ENSG00000166477	LEO1	LEO1 homolog, Paf1/RNA polymerase II complex component	-0,5716	0,6729	0,0000	0,0000
ENSG00000100097	LGALS1	lectin, galactoside-binding, soluble, 1	0,4904	1,4048	0,0000	0,0002
ENSG00000131981	LGALS3	lectin, galactoside-binding, soluble, 3	0,5241	1,4380	0,0000	0,0000
ENSG00000168961	LGALS9	lectin, galactoside-binding, soluble, 9	1,1129	2,1628	0,0000	0,0000
ENSG00000139292	LGR5	leucine-rich repeat containing G protein-coupled receptor 5	1,1100	2,1585	0,0000	0,0003
ENSG00000183722	LHFP	lipoma HMGIC fusion partner	-0,6109	0,6548	0,0000	0,0000
ENSG00000107902	LHPP	phospholysine phosphohistidine inorganic pyrophosphate phosphatase	0,4729	1,3879	0,0001	0,0019
ENSG00000174405	LIG4	ligase IV, DNA, ATP-dependent	-1,3754	0,3854	0,0000	0,0000
ENSG00000239998	LILRA2	leukocyte immunoglobulin-like receptor, subfamily A (with TM domain), member 2	2,2220	4,6655	0,0000	0,0000
ENSG00000205659	LIN52	lin-52 DREAM MuvB core complex component	0,3871	1,3078	0,0000	0,0007
ENSG00000104863	LIN7B	lin-7 homolog B (C. elegans)	0,6208	1,5378	0,0009	0,0087
ENSG00000175701	LINC00116	long intergenic non-protein coding RNA 116	0,4224	1,3401	0,0008	0,0077
ENSG00000222041	LINC00152	long intergenic non-protein coding RNA 152	-0,4709	0,7215	0,0000	0,0000
ENSG00000196668	LINC00173	long intergenic non-protein coding RNA 173	-0,8621	0,5501	0,0000	0,0000
ENSG00000179406	LINC00174	long intergenic non-protein coding RNA 174	0,6664	1,5871	0,0000	0,0005
ENSG00000175699	LINC00521	long intergenic non-protein coding RNA 521	1,6133	3,0595	0,0000	0,0000
ENSG00000276476	LINC00540	long intergenic non-protein coding RNA 540	1,1043	2,1499	0,0001	0,0008
ENSG00000258441	LINC00641	long intergenic non-protein coding RNA 641	-0,4589	0,7276	0,0000	0,0007
ENSG00000263753	LINC00667	long intergenic non-protein coding RNA 667	-0,5470	0,6844	0,0000	0,0003
ENSG00000267535	LINC00868	long intergenic non-protein coding RNA 868	-0,7632	0,5892	0,0000	0,0000
ENSG00000235703	LINC00894	long intergenic non-protein coding RNA 894	0,6572	1,5770	0,0004	0,0042

ENSG00000231711	LINC00899	long intergenic non-protein coding RNA 899	0,5708	1,4854	0,0000	0,0002
ENSG00000188825	LINC00910	long intergenic non-protein coding RNA 910	0,5797	1,4946	0,0005	0,0056
ENSG00000235884	LINC00941	long intergenic non-protein coding RNA 941	-0,4888	0,7126	0,0000	0,0004
ENSG00000256128	LINC00944	long intergenic non-protein coding RNA 944	-0,9775	0,5079	0,0004	0,0039
ENSG00000281205	LINC00950	long intergenic non-protein coding RNA 950	1,7012	3,2517	0,0000	0,0000
ENSG00000228784	LINC00954	long intergenic non-protein coding RNA 954	0,9011	1,8675	0,0002	0,0026
ENSG00000235314	LINC00957	long intergenic non-protein coding RNA 957	0,8077	1,7505	0,0000	0,0000
ENSG00000204054	LINC00963	long intergenic non-protein coding RNA 963	0,5079	1,4219	0,0000	0,0000
ENSG00000240476	LINC00973	long intergenic non-protein coding RNA 973	0,7144	1,6408	0,0001	0,0011
ENSG00000244041	LINC01011	long intergenic non-protein coding RNA 1011	0,7661	1,7007	0,0000	0,0004
ENSG00000249378	LINC01060	long intergenic non-protein coding RNA 1060	-0,7160	0,6088	0,0008	0,0077
ENSG00000222033	LINC01124	long intergenic non-protein coding RNA 1124	0,7131	1,6393	0,0000	0,0000
ENSG00000232987	LINC01219	long intergenic non-protein coding RNA 1219	1,6591	3,1582	0,0000	0,0000
ENSG00000231742	LINC01273	long intergenic non-protein coding RNA 1273	0,7320	1,6609	0,0000	0,0001
ENSG00000280587	LINC01348	long intergenic non-protein coding RNA 1348	0,7868	1,7252	0,0000	0,0006
ENSG00000262468	LINC01569	long intergenic non-protein coding RNA 1569	0,6608	1,5810	0,0000	0,0007
ENSG00000259518	LINC01583	long intergenic non-protein coding RNA 1583	1,3173	2,4921	0,0000	0,0000
ENSG00000169783	LINGO1	leucine rich repeat and Ig domain containing 1	1,8391	3,5779	0,0000	0,0000
ENSG00000174482	LINGO2	leucine rich repeat and Ig domain containing 2	0,9997	1,9995	0,0002	0,0019
ENSG00000073350	LLGL2	lethal giant larvae homolog 2 (Drosophila)	0,5204	1,4343	0,0003	0,0034
ENSG00000139233	LLPH	LLP homolog, long-term synaptic facilitation (Aplysia)	-0,4966	0,7088	0,0000	0,0000
ENSG00000139636	LMBR1L	limb development membrane protein 1-like	0,4029	1,3222	0,0003	0,0034
ENSG00000071282	LMCD1	LIM and cysteine-rich domains 1	0,6279	1,5454	0,0000	0,0001
ENSG00000135363	LMO2	LIM domain only 2 (rhombotin-like 1)	-0,4129	0,7511	0,0003	0,0032
ENSG00000136153	LMO7	LIM domain 7	-0,6155	0,6527	0,0000	0,0000
ENSG00000163431	LMOD1	leiomodin 1 (smooth muscle)	1,1826	2,2698	0,0000	0,0002
ENSG00000142235	LMTK3	lemur tyrosine kinase 3	0,6799	1,6020	0,0000	0,0002
ENSG00000196365	LONP1	lon peptidase 1, mitochondrial	0,4293	1,3466	0,0001	0,0011
ENSG00000113083	LOX	lysyl oxidase	1,6207	3,0752	0,0000	0,0000
ENSG00000134013	LOXL2	lysyl oxidase-like 2	0,5349	1,4488	0,0000	0,0002
ENSG00000213071	LPAL2	lipoprotein, Lp(a)-like 2, pseudogene	-0,7271	0,6041	0,0007	0,0068

ENSG00000198121	LPAR1	lysophosphatidic acid receptor 1	0,7740	1,7099	0,0000	0,0005
ENSG00000087253	LPCAT2	lysophosphatidylcholine acyltransferase 2	-0,4923	0,7109	0,0000	0,0000
ENSG00000132793	LPIN3	lipin 3	0,4905	1,4050	0,0000	0,0004
ENSG00000175445	LPL	lipoprotein lipase	-1,3799	0,3843	0,0000	0,0000
ENSG00000136141	LRCH1	leucine-rich repeats and calponin homology (CH) domain containing 1	-0,7111	0,6109	0,0000	0,0000
ENSG00000144749	LRIG1	leucine-rich repeats and immunoglobulin-like domains 1	-0,4082	0,7536	0,0000	0,0000
ENSG00000123384	LRP1	low density lipoprotein receptor-related protein 1	0,8748	1,8337	0,0001	0,0012
ENSG00000081479	LRP2	low density lipoprotein receptor-related protein 2	1,1130	2,1630	0,0000	0,0000
ENSG00000247675	LRP4-AS1	LRP4 antisense RNA 1	0,8274	1,7745	0,0003	0,0036
ENSG00000165501	LRR1	leucine rich repeat protein 1	-0,4400	0,7371	0,0000	0,0001
ENSG00000204950	LRRC10B	leucine rich repeat containing 10B	0,9750	1,9657	0,0000	0,0000
ENSG00000010626	LRRC23	leucine rich repeat containing 23	0,5211	1,4350	0,0000	0,0000
ENSG00000268324	LRRC2-AS1	LRRC2 antisense RNA 1	0,8889	1,8517	0,0010	0,0096
ENSG00000137507	LRRC32	leucine rich repeat containing 32	2,2318	4,6972	0,0000	0,0000
ENSG00000171757	LRRC34	leucine rich repeat containing 34	-0,5718	0,6728	0,0000	0,0000
ENSG00000108829	LRRC59	leucine rich repeat containing 59	-0,5823	0,6679	0,0000	0,0000
ENSG00000178026	LRRC75B	leucine rich repeat containing 75B	0,9352	1,9122	0,0000	0,0000
ENSG00000197147	LRRC8B	leucine rich repeat containing 8 family, member B	-0,5210	0,6969	0,0000	0,0000
ENSG00000171488	LRRC8C	leucine rich repeat containing 8 family, member C	-1,5061	0,3521	0,0000	0,0000
ENSG00000124831	LRRFIP1	leucine rich repeat (in FLII) interacting protein 1	-0,5687	0,6742	0,0000	0,0000
ENSG00000148356	LRSAM1	leucine rich repeat and sterile alpha motif containing 1	0,6958	1,6198	0,0000	0,0000
ENSG00000041802	LSG1	large 60S subunit nuclear export GTPase 1	-0,4269	0,7439	0,0000	0,0002
ENSG00000106355	LSM5	LSM5 homolog, U6 small nuclear RNA and mRNA degradation associated	-0,5290	0,6930	0,0000	0,0000
ENSG00000181016	LSMEM1	leucine-rich single-pass membrane protein 1	-0,6714	0,6279	0,0001	0,0015
ENSG00000227507	LTB	lymphotoxin beta (TNF superfamily, member 3)	-0,9869	0,5046	0,0001	0,0008
ENSG00000049323	LTBP1	latent transforming growth factor beta binding protein 1	-0,5981	0,6606	0,0000	0,0000
ENSG00000119681	LTBP2	latent transforming growth factor beta binding protein 2	-0,8883	0,5403	0,0000	0,0000
ENSG00000012223	LTF	lactotransferrin	0,9523	1,9350	0,0005	0,0051
ENSG00000135521	LTV1	LTV1 ribosome biogenesis factor	-0,5732	0,6721	0,0000	0,0000
ENSG00000248323	LUCAT1	lung cancer associated transcript 1 (non-protein coding)	0,4061	1,3251	0,0005	0,0056
ENSG00000169641	LUZP1	leucine zipper protein 1	-0,5074	0,7035	0,0000	0,0000

ENSG00000204421	LY6G6C	lymphocyte antigen 6 complex, locus G6C	0,9833	1,9769	0,0001	0,0014
ENSG00000145220	LYAR	Ly1 antibody reactive	-0,6826	0,6230	0,0000	0,0000
ENSG00000159871	LYPD5	LY6/PLAUR domain containing 5	0,6097	1,5259	0,0002	0,0027
ENSG00000150556	LYPD6B	LY6/PLAUR domain containing 6B	1,3396	2,5307	0,0000	0,0000
ENSG00000102897	LYRM1	LYR motif containing 1	-0,4194	0,7477	0,0001	0,0015
ENSG00000232859	LYRM9	LYR motif containing 9	0,8432	1,7940	0,0000	0,0000
ENSG00000140280	LYSMD2	LysM, putative peptidoglycan-binding, domain containing 2	-0,6445	0,6397	0,0000	0,0000
ENSG00000099949	LZTR1	leucine-zipper-like transcription regulator 1	0,5044	1,4185	0,0000	0,0001
ENSG00000107816	LZTS2	leucine zipper, putative tumor suppressor 2	0,4318	1,3489	0,0002	0,0022
ENSG00000183742	MACC1	metastasis associated in colon cancer 1	2,4979	5,6487	0,0000	0,0000
ENSG00000133315	MACROD1	MACRO domain containing 1	0,4802	1,3949	0,0000	0,0003
ENSG00000178573	MAF	v-maf avian musculoaponeurotic fibrosarcoma oncogene homolog	0,8344	1,7831	0,0005	0,0048
ENSG00000204103	MAFB	v-maf avian musculoaponeurotic fibrosarcoma oncogene homolog B	0,5983	1,5139	0,0000	0,0000
ENSG00000046774	MAGEC2	melanoma antigen family C2	0,4904	1,4049	0,0000	0,0000
ENSG00000198042	MAK16	MAK16 homolog	-0,5996	0,6599	0,0000	0,0000
ENSG00000172175	MALT1	MALT1 paracaspase	-0,5727	0,6724	0,0000	0,0000
ENSG00000177239	MAN1B1	mannosidase, alpha, class 1B, member 1	0,3979	1,3176	0,0000	0,0007
ENSG00000140400	MAN2C1	mannosidase, alpha, class 2C, member 1	0,5614	1,4757	0,0000	0,0000
ENSG00000109323	MANBA	mannosidase, beta A, lysosomal	0,4951	1,4094	0,0000	0,0000
ENSG00000189221	MAOA	monoamine oxidase A	0,5503	1,4644	0,0001	0,0013
ENSG00000166963	MAP1A	microtubule-associated protein 1A	0,7829	1,7206	0,0009	0,0089
ENSG00000131711	MAP1B	microtubule-associated protein 1B	0,5085	1,4226	0,0000	0,0000
ENSG00000130758	MAP3K10	mitogen-activated protein kinase kinase kinase 10	0,5253	1,4392	0,0010	0,0091
ENSG00000168067	MAP4K2	mitogen-activated protein kinase kinase kinase kinase 2	0,5975	1,5131	0,0000	0,0001
ENSG00000180834	MAP6D1	MAP6 domain containing 1	-0,4191	0,7479	0,0004	0,0045
ENSG00000181085	MAPK15	mitogen-activated protein kinase 15	0,9136	1,8837	0,0008	0,0079
ENSG00000102882	MAPK3	mitogen-activated protein kinase 3	0,4007	1,3202	0,0002	0,0019
ENSG00000121653	MAPK8IP1	mitogen-activated protein kinase 8 interacting protein 1	1,0478	2,0673	0,0000	0,0000
ENSG00000084764	MAPRE3	microtubule-associated protein, RP/EB family, member 3	0,7547	1,6873	0,0000	0,0000
ENSG00000186868	MAPT	microtubule-associated protein tau	1,0253	2,0354	0,0000	0,0000
ENSG00000144583	MARCH4	membrane-associated ring finger (C3HC4) 4, E3 ubiquitin protein ligase	-1,0267	0,4908	0,0000	0,0000

ENSG00000165406	MARCH8	membrane-associated ring finger (C3HC4) 8, E3 ubiquitin protein ligase	0,5123	1,4263	0,0000	0,0000
ENSG00000166986	MARS	methionyl-tRNA synthetase	0,5071	1,4212	0,0000	0,0000
ENSG00000247626	MARS2	methionyl-tRNA synthetase 2, mitochondrial	-0,6793	0,6245	0,0000	0,0000
ENSG00000105613	MAST1	microtubule associated serine/threonine kinase 1	0,6761	1,5978	0,0005	0,0051
ENSG00000151224	MAT1A	methionine adenosyltransferase I, alpha	1,7079	3,2670	0,0000	0,0000
ENSG00000168906	MAT2A	methionine adenosyltransferase II, alpha	-0,6718	0,6277	0,0000	0,0000
ENSG00000280987	MATR3	matrin 3	-0,6925	0,6188	0,0000	0,0001
ENSG00000166987	MBD6	methyl-CpG binding domain protein 6	0,4413	1,3578	0,0000	0,0001
ENSG00000151332	MBIP	MAP3K12 binding inhibitory protein 1	-0,4389	0,7377	0,0000	0,0003
ENSG00000171444	MCC	mutated in colorectal cancers	-0,3900	0,7632	0,0000	0,0001
ENSG00000180398	MCFD2	multiple coagulation factor deficiency 2	-0,5454	0,6852	0,0000	0,0000
ENSG00000175471	MCTP1	multiple C2 domains, transmembrane 1	0,6895	1,6127	0,0000	0,0000
ENSG00000140563	MCTP2	multiple C2 domains, transmembrane 2	-0,5571	0,6797	0,0001	0,0017
ENSG00000232119	MCTS1	malignant T cell amplified sequence 1	-0,4180	0,7485	0,0003	0,0031
ENSG00000144893	MED12L	mediator complex subunit 12-like	-0,7277	0,6039	0,0000	0,0000
ENSG00000123066	MED13L	mediator complex subunit 13-like	-0,3905	0,7629	0,0000	0,0000
ENSG00000175221	MED16	mediator complex subunit 16	0,5303	1,4442	0,0003	0,0033
ENSG00000112282	MED23	mediator complex subunit 23	0,4152	1,3335	0,0000	0,0000
ENSG00000104973	MED25	mediator complex subunit 25	0,4206	1,3385	0,0005	0,0056
ENSG00000136146	MED4	mediator complex subunit 4	-0,4462	0,7340	0,0000	0,0001
ENSG00000145794	MEGF10	multiple EGF-like-domains 10	-1,5085	0,3515	0,0000	0,0000
ENSG00000157890	MEGF11	multiple EGF-like-domains 11	1,1302	2,1889	0,0000	0,0000
ENSG00000162591	MEGF6	multiple EGF-like-domains 6	0,8465	1,7981	0,0003	0,0036
ENSG00000111142	METAP2	methionyl aminopeptidase 2	-0,4661	0,7239	0,0000	0,0001
ENSG00000037897	METTL1	methyltransferase like 1	-0,5990	0,6602	0,0000	0,0000
ENSG00000010165	METTL13	methyltransferase like 13	-0,4397	0,7373	0,0000	0,0000
ENSG00000139160	METTL20	methyltransferase like 20	0,8470	1,7987	0,0003	0,0031
ENSG00000165819	METTL3	methyltransferase like 3	-0,4044	0,7556	0,0000	0,0001
ENSG00000185432	METTL7A	methyltransferase like 7A	0,9397	1,9181	0,0000	0,0003
ENSG00000123600	METTL8	methyltransferase like 8	-0,3795	0,7687	0,0001	0,0015
ENSG00000197006	METTL9	methyltransferase like 9	-0,4160	0,7495	0,0000	0,0006

ENSG00000254726	MEX3A	mex-3 RNA binding family member A	-0,4737	0,7201	0,0000	0,0000
ENSG00000117122	MFAP2	microfibrillar-associated protein 2	0,7675	1,7023	0,0000	0,0000
ENSG00000198948	MFAP3L	microfibrillar-associated protein 3-like	-0,9080	0,5329	0,0000	0,0000
ENSG00000140545	MFGE8	milk fat globule-EGF factor 8 protein	0,4232	1,3409	0,0004	0,0041
ENSG00000168389	MFSD2A	major facilitator superfamily domain containing 2A	-0,7125	0,6102	0,0000	0,0000
ENSG00000128268	MGAT3	mannosyl (beta-1,4-)-glycoprotein beta-1,4-N-acetylglucosaminyltransferase	0,9438	1,9236	0,0000	0,0000
ENSG00000167889	MGAT5B	mannosyl (alpha-1,6-)-glycoprotein beta-1,6-N-acetyl-glucosaminyltransferase, iso	-0,5431	0,6863	0,0010	0,0093
ENSG00000170430	MGMT	O-6-methylguanine-DNA methyltransferase	0,6526	1,5720	0,0000	0,0004
ENSG00000154305	MIA3	melanoma inhibitory activity family, member 3	0,3793	1,3007	0,0000	0,0001
ENSG00000225783	MIAT	myocardial infarction associated transcript (non-protein coding)	1,1191	2,1721	0,0000	0,0000
ENSG00000165487	MICU2	mitochondrial calcium uptake 2	-0,6377	0,6427	0,0000	0,0000
ENSG00000177427	MIEF2	mitochondrial elongation factor 2	0,3996	1,3191	0,0003	0,0030
ENSG00000125457	MIF4GD	MIF4G domain containing	0,4916	1,4060	0,0000	0,0000
ENSG00000170854	MINA	MYC induced nuclear antigen	-0,4486	0,7327	0,0001	0,0009
ENSG00000173436	MINOS1	mitochondrial inner membrane organizing system 1	-0,6551	0,6350	0,0000	0,0000
ENSG00000027001	MIPEP	mitochondrial intermediate peptidase	-0,4202	0,7473	0,0001	0,0018
ENSG00000215417	MIR17HG	miR-17-92 cluster host gene	-1,2412	0,4230	0,0000	0,0000
ENSG00000247095	MIR210HG	MIR210 host gene	1,0848	2,1210	0,0000	0,0000
ENSG00000270069	MIR222HG	MIR222 host gene	-0,7921	0,5775	0,0000	0,0000
ENSG00000186594	MIR22HG	MIR22 host gene	-0,5278	0,6936	0,0000	0,0000
ENSG00000253522	MIR3142HG	MIR3142 host gene	-0,8441	0,5571	0,0000	0,0000
ENSG00000172965	MIR4435-2HG	MIR4435-2 host gene	-0,4891	0,7125	0,0000	0,0000
ENSG00000223749	MIR503HG	MIR503 host gene	0,4410	1,3576	0,0001	0,0012
ENSG00000148773	MKI67	marker of proliferation Ki-67	-0,5533	0,6814	0,0000	0,0000
ENSG00000099875	MKNK2	MAP kinase interacting serine/threonine kinase 2	0,4374	1,3542	0,0001	0,0016
ENSG00000179455	MKRN3	makorin ring finger protein 3	-0,9323	0,5240	0,0000	0,0000
ENSG00000011143	MKS1	Meckel syndrome, type 1	0,4573	1,3730	0,0000	0,0003
ENSG00000150051	MKX	mohawk homeobox	0,6819	1,6042	0,0004	0,0043
ENSG00000110917	MLEC	malectin	-0,3989	0,7584	0,0000	0,0000
ENSG00000178053	MLF1	myeloid leukemia factor 1	0,4599	1,3755	0,0000	0,0004
ENSG00000168404	MLKL	mixed lineage kinase domain-like	-0,5908	0,6640	0,0000	0,0000

ENSG00000171843	MLLT3	myeloid/lymphoid or mixed-lineage leukemia; translocated to, 3	0,4389	1,3556	0,0002	0,0025
ENSG00000115648	MLPH	melanophilin	-0,4749	0,7195	0,0000	0,0000
ENSG00000167965	MLST8	MTOR associated protein, LST8 homolog	0,5433	1,4573	0,0000	0,0005
ENSG00000009950	MLXIPL	MLX interacting protein-like	0,7676	1,7024	0,0000	0,0000
ENSG00000099953	MMP11	matrix metallopeptidase 11	1,1746	2,2573	0,0000	0,0000
ENSG00000102996	MMP15	matrix metallopeptidase 15 (membrane-inserted)	0,5047	1,4188	0,0002	0,0026
ENSG00000125966	MMP24	matrix metallopeptidase 24 (membrane-inserted)	0,5156	1,4296	0,0005	0,0053
ENSG00000126005	MMP24-AS1	MMP24 antisense RNA 1	0,8548	1,8085	0,0000	0,0000
ENSG00000100985	MMP9	matrix metallopeptidase 9	-1,7019	0,3074	0,0000	0,0000
ENSG00000173269	MMRN2	multimerin 2	0,8309	1,7787	0,0000	0,0000
ENSG00000106384	MOGAT3	monoacylglycerol O-acyltransferase 3	1,0698	2,0992	0,0000	0,0000
ENSG00000164077	MON1A	MON1 secretory trafficking family member A	-0,3906	0,7628	0,0006	0,0063
ENSG00000171160	MORN4	MORN repeat containing 4	0,7250	1,6529	0,0000	0,0000
ENSG00000101928	MOSPD1	motile sperm domain containing 1	-0,3871	0,7647	0,0001	0,0010
ENSG00000103152	MPG	N-methylpurine DNA glycosylase	0,4886	1,4031	0,0007	0,0068
ENSG00000124383	MPHOSPH10	M-phase phosphoprotein 10 (U3 small nucleolar ribonucleoprotein)	-0,4848	0,7146	0,0000	0,0001
ENSG00000135698	MPHOSPH6	M-phase phosphoprotein 6	-0,4868	0,7136	0,0000	0,0003
ENSG00000196199	MPHOSPH8	M-phase phosphoprotein 8	-0,5361	0,6896	0,0000	0,0000
ENSG00000178802	MPI	mannose phosphate isomerase	0,5177	1,4317	0,0000	0,0000
ENSG00000108852	MPP2	membrane protein, palmitoylated 2 (MAGUK p55 subfamily member 2)	0,5665	1,4809	0,0000	0,0001
ENSG00000072415	MPP5	membrane protein, palmitoylated 5 (MAGUK p55 subfamily member 5)	-0,5452	0,6853	0,0000	0,0000
ENSG00000105926	MPP6	membrane protein, palmitoylated 6 (MAGUK p55 subfamily member 6)	-0,6262	0,6479	0,0000	0,0000
ENSG00000154889	MPPE1	metallophosphoesterase 1	0,4103	1,3290	0,0002	0,0025
ENSG00000160588	MPZL3	myelin protein zero-like 3	0,5610	1,4752	0,0000	0,0000
ENSG00000153029	MR1	major histocompatibility complex, class I-related	0,5641	1,4785	0,0000	0,0000
ENSG00000118242	MREG	melanoregulin	-0,5490	0,6835	0,0000	0,0000
ENSG00000179832	MROH1	maestro heat-like repeat family member 1	0,5949	1,5104	0,0000	0,0002
ENSG00000184313	MROH7	maestro heat-like repeat family member 7	0,8990	1,8647	0,0010	0,0091
ENSG00000169288	MRPL1	mitochondrial ribosomal protein L1	-0,4173	0,7488	0,0004	0,0046
ENSG00000114686	MRPL3	mitochondrial ribosomal protein L3	-0,4156	0,7497	0,0001	0,0012
ENSG00000106591	MRPL32	mitochondrial ribosomal protein L32	-0,4869	0,7135	0,0000	0,0001

ENSG00000132313	MRPL35	mitochondrial ribosomal protein L35	-0,3925	0,7618	0,0002	0,0027
ENSG00000102738	MRPS31	mitochondrial ribosomal protein S31	-0,5440	0,6858	0,0000	0,0000
ENSG00000135972	MRPS9	mitochondrial ribosomal protein S9	-0,4230	0,7459	0,0000	0,0003
ENSG00000053372	MRTO4	MRT4 homolog, ribosome maturation factor	-0,6979	0,6165	0,0000	0,0000
ENSG00000120458	MSANTD2	Myb/SANT-like DNA-binding domain containing 2	-0,6832	0,6228	0,0000	0,0000
ENSG00000204410	MSH5	mutS homolog 5	0,5424	1,4564	0,0007	0,0071
ENSG00000174099	MSRB3	methionine sulfoxide reductase B3	-0,4149	0,7501	0,0000	0,0001
ENSG00000173531	MST1	macrophage stimulating 1	1,1666	2,2448	0,0000	0,0000
ENSG00000163132	MSX1	msh homeobox 1	-0,8682	0,5478	0,0000	0,0000
ENSG00000205362	MT1A	metallothionein 1A	0,9716	1,9610	0,0000	0,0000
ENSG00000187193	MT1X	metallothionein 1X	0,8607	1,8159	0,0000	0,0000
ENSG00000087250	MT3	metallothionein 3	1,1910	2,2830	0,0000	0,0000
ENSG00000237973	MTCO1P12	MT-CO1 pseudogene 12	-0,3790	0,7689	0,0007	0,0073
ENSG00000147649	MTDH	metadherin	-0,4058	0,7548	0,0000	0,0001
ENSG00000127989	MTERF1	mitochondrial transcription termination factor 1	-0,4793	0,7173	0,0000	0,0006
ENSG00000163738	MTHFD2L	methylenetetrahydrofolate dehydrogenase (NADP+ dependent) 2-like	-0,5713	0,6730	0,0000	0,0000
ENSG00000132749	MTL5	metallothionein-like 5, testis-specific (tesmin)	0,4950	1,4093	0,0000	0,0002
ENSG00000014914	MTMR11	myotubularin related protein 11	0,4797	1,3945	0,0002	0,0024
ENSG00000100330	MTMR3	myotubularin related protein 3	0,3821	1,3033	0,0000	0,0001
ENSG00000139505	MTMR6	myotubularin related protein 6	-0,7404	0,5986	0,0000	0,0000
ENSG00000220785	MTMR9LP	myotubularin related protein 9-like, pseudogene	0,8233	1,7695	0,0001	0,0011
ENSG00000198888	MT-ND1	mitochondrially encoded NADH dehydrogenase 1	-0,4631	0,7254	0,0000	0,0000
ENSG00000210082	MT-RNR2	mitochondrially encoded 16S RNA	0,6561	1,5759	0,0000	0,0000
ENSG00000124275	MTRR	5-methyltetrahydrofolate-homocysteine methyltransferase reductase	-0,5470	0,6844	0,0000	0,0000
ENSG00000170873	MTSS1	metastasis suppressor 1	-0,4226	0,7461	0,0000	0,0000
ENSG00000132613	MTSS1L	metastasis suppressor 1-like	0,7013	1,6260	0,0000	0,0000
ENSG00000210194	MT-TE	mitochondrially encoded tRNA glutamic acid	-0,7239	0,6055	0,0000	0,0006
ENSG00000129422	MTUS1	microtubule associated tumor suppressor 1	0,5609	1,4752	0,0000	0,0000
ENSG00000185499	MUC1	mucin 1, cell surface associated	0,5682	1,4826	0,0008	0,0079
ENSG00000181143	MUC16	mucin 16, cell surface associated	1,4143	2,6653	0,0000	0,0000
ENSG00000204544	MUC21	mucin 21, cell surface associated	0,9458	1,9263	0,0006	0,0059

ENSG00000132781	MUTYH	mutY DNA glycosylase	0,4612	1,3767	0,0003	0,0029
ENSG00000013364	MVP	major vault protein	1,2045	2,3045	0,0000	0,0000
ENSG00000059728	MXD1	MAX dimerization protein 1	0,7205	1,6477	0,0000	0,0000
ENSG00000213347	MXD3	MAX dimerization protein 3	0,8272	1,7743	0,0000	0,0000
ENSG00000123933	MXD4	MAX dimerization protein 4	1,1963	2,2915	0,0000	0,0000
ENSG00000119950	MXI1	MAX interactor 1, dimerization protein	0,8633	1,8192	0,0000	0,0000
ENSG00000179820	MYADM	myeloid-associated differentiation marker	0,4444	1,3607	0,0000	0,0000
ENSG00000086967	MYBPC2	myosin binding protein C, fast type	1,1251	2,1811	0,0000	0,0000
ENSG00000214114	MYCBP	MYC binding protein	-0,5054	0,7044	0,0000	0,0001
ENSG00000005810	MYCBP2	MYC binding protein 2, E3 ubiquitin protein ligase	-0,8720	0,5464	0,0000	0,0000
ENSG00000172927	MYEOV	myeloma overexpressed	-0,4289	0,7428	0,0000	0,0000
ENSG00000144821	MYH15	myosin, heavy chain 15	-2,0542	0,2408	0,0000	0,0000
ENSG00000078814	MYH7B	myosin, heavy chain 7B, cardiac muscle, beta	1,3042	2,4694	0,0000	0,0000
ENSG00000272975	MYHAS	myosin heavy chain gene cluster antisense RNA	0,9810	1,9739	0,0002	0,0027
ENSG00000101608	MYL12A	myosin, light chain 12A, regulatory, non-sarcomeric	-0,4518	0,7311	0,0000	0,0000
ENSG00000118680	MYL12B	myosin, light chain 12B, regulatory	-0,4358	0,7393	0,0000	0,0001
ENSG00000215375	MYL5	myosin, light chain 5, regulatory	0,6596	1,5796	0,0000	0,0004
ENSG00000101335	MYL9	myosin, light chain 9, regulatory	0,6245	1,5417	0,0000	0,0000
ENSG00000065534	MYLK	myosin light chain kinase	1,0508	2,0716	0,0000	0,0000
ENSG00000091536	MYO15A	myosin XVA	0,9991	1,9987	0,0000	0,0003
ENSG00000266714	MYO15B	myosin XVB	0,6083	1,5244	0,0000	0,0005
ENSG00000196535	MYO18A	myosin XVIIIA	0,7761	1,7125	0,0000	0,0000
ENSG00000124920	MYRF	myelin regulatory factor	0,6735	1,5950	0,0000	0,0000
ENSG00000196132	MYT1	myelin transcription factor 1	2,0029	4,0080	0,0000	0,0000
ENSG00000204899	MZT1	mitotic spindle organizing protein 1	-0,9575	0,5150	0,0000	0,0000
ENSG00000139597	N4BP2L1	NEDD4 binding protein 2-like 1	0,5845	1,4995	0,0006	0,0060
ENSG00000145911	N4BP3	NEDD4 binding protein 3	0,7209	1,6482	0,0000	0,0005
ENSG00000150456	N6AMT2	N-6 adenine-specific DNA methyltransferase 2 (putative)	-0,8565	0,5523	0,0000	0,0000
ENSG00000164134	NAA15	N(alpha)-acetyltransferase 15, NatA auxiliary subunit	-0,4979	0,7081	0,0000	0,0000
ENSG00000172766	NAA16	N(alpha)-acetyltransferase 16, NatA auxiliary subunit	-0,4147	0,7502	0,0002	0,0021
ENSG00000139977	NAA30	N(alpha)-acetyltransferase 30, NatC catalytic subunit	-0,4755	0,7192	0,0000	0,0000

ENSG00000121579	NAA50	N(alpha)-acetyltransferase 50, NatE catalytic subunit	-0,4053	0,7551	0,0000	0,0005
ENSG00000237886	NALT1	NOTCH1 associated lncRNA in T cell acute lymphoblastic leukemia 1	0,9374	1,9151	0,0000	0,0006
ENSG00000105835	NAMPT	nicotinamide phosphoribosyltransferase	-0,6989	0,6160	0,0000	0,0000
ENSG00000229644	NAMPTP1	nicotinamide phosphoribosyltransferase pseudogene 1	-0,6928	0,6187	0,0000	0,0000
ENSG00000188613	NANOS1	nanos homolog 1 (Drosophila)	-0,8164	0,5679	0,0000	0,0000
ENSG00000268061	NAPA-AS1	NAPA antisense RNA 1	0,9276	1,9021	0,0000	0,0001
ENSG00000161048	NAPEPLD	N-acyl phosphatidylethanolamine phospholipase D	-0,4838	0,7151	0,0000	0,0000
ENSG00000134265	NAPG	N-ethylmaleimide-sensitive factor attachment protein, gamma	-0,3976	0,7591	0,0000	0,0000
ENSG00000103245	NARFL	nuclear prelamin A recognition factor-like	0,4694	1,3845	0,0002	0,0020
ENSG00000167011	NAT16	N-acetyltransferase 16 (GCN5-related, putative)	0,6070	1,5231	0,0000	0,0000
ENSG00000243477	NAT6	N-acetyltransferase 6 (GCN5-related)	0,4762	1,3911	0,0000	0,0003
ENSG00000274180	NATD1	N-acetyltransferase domain containing 1	0,4462	1,3625	0,0000	0,0004
ENSG00000172915	NBEA	neurobeachin	-1,4272	0,3718	0,0000	0,0000
ENSG00000160796	NBEAL2	neurobeachin-like 2	0,7693	1,7045	0,0000	0,0000
ENSG00000104320	NBN	nibrin	-0,4078	0,7538	0,0001	0,0008
ENSG00000104490	NCALD	neurocalcin delta	-1,2876	0,4096	0,0000	0,0000
ENSG00000149294	NCAM1	neural cell adhesion molecule 1	1,1600	2,2346	0,0000	0,0000
ENSG00000136937	NCBP1	nuclear cap binding protein subunit 1, 80kDa	-0,4446	0,7348	0,0000	0,0000
ENSG00000144959	NCEH1	neutral cholesterol ester hydrolase 1	-0,5217	0,6965	0,0000	0,0000
ENSG00000116701	NCF2	neutrophil cytosolic factor 2	1,0394	2,0553	0,0000	0,0001
ENSG00000239213	NCK1-AS1	NCK1 antisense RNA 1 (head to head)	0,4759	1,3907	0,0003	0,0032
ENSG00000213672	NCKIPSD	NCK interacting protein with SH3 domain	0,5665	1,4809	0,0000	0,0000
ENSG00000115053	NCL	nucleolin	-0,5501	0,6830	0,0000	0,0000
ENSG00000266412	NCOA4	nuclear receptor coactivator 4	-0,4332	0,7406	0,0000	0,0000
ENSG00000124160	NCOA5	nuclear receptor coactivator 5	-0,4221	0,7463	0,0000	0,0000
ENSG00000240108	NCOR1P1	nuclear receptor corepressor 1 pseudogene 1	1,2944	2,4527	0,0000	0,0000
ENSG00000188211	NCR3LG1	natural killer cell cytotoxicity receptor 3 ligand 1	-0,5768	0,6704	0,0000	0,0000
ENSG00000166579	NDEL1	nudE neurodevelopment protein 1-like 1	-0,3874	0,7645	0,0000	0,0000
ENSG00000102471	NDFIP2	Nedd4 family interacting protein 2	-0,9094	0,5324	0,0000	0,0000
ENSG00000185115	NDNL2	necdin-like 2, SMC5-SMC6 complex component	-0,4634	0,7253	0,0000	0,0004
ENSG00000124479	NDP	Norrie disease (pseudoglioma)	-1,0871	0,4707	0,0000	0,0000

ENSG00000104419	NDRG1	N-myc downstream regulated 1	0,9860	1,9808	0,0000	0,0000
ENSG00000103034	NDRG4	NDRG family member 4	0,7671	1,7019	0,0000	0,0000
ENSG00000185633	NDUFA4L2	NADH dehydrogenase (ubiquinone) 1 alpha subcomplex, 4-like 2	0,8035	1,7454	0,0006	0,0060
ENSG00000123545	NDUFAF4	NADH dehydrogenase (ubiquinone) complex I, assembly factor 4	-0,6985	0,6162	0,0000	0,0000
ENSG00000245532	NEAT1	nuclear paraspeckle assembly transcript 1 (non-protein coding)	0,9105	1,8797	0,0000	0,0000
ENSG00000078114	NEBL	nebulette	-0,5334	0,6909	0,0000	0,0000
ENSG00000125967	NECAB3	N-terminal EF-hand calcium binding protein 3	0,5378	1,4518	0,0000	0,0007
ENSG00000140398	NEIL1	nei-like DNA glycosylase 1	0,4342	1,3511	0,0005	0,0052
ENSG00000160602	NEK8	NIMA-related kinase 8	0,5875	1,5026	0,0000	0,0000
ENSG00000165525	NEMF	nuclear export mediator factor	-0,4395	0,7374	0,0000	0,0001
ENSG00000173848	NET1	neuroepithelial cell transforming 1	-0,7381	0,5995	0,0000	0,0000
ENSG00000171208	NETO2	neuropilin (NRP) and tolloid (TLL)-like 2	-0,4531	0,7305	0,0000	0,0002
ENSG00000124257	NEURL2	neuralized E3 ubiquitin protein ligase 2	0,7956	1,7357	0,0003	0,0030
ENSG00000100968	NFATC4	nuclear factor of activated T-cells, cytoplasmic, calcineurin-dependent 4	0,5069	1,4210	0,0000	0,0005
ENSG00000050344	NFE2L3	nuclear factor, erythroid 2-like 3	-0,7718	0,5857	0,0000	0,0000
ENSG00000162599	NFIA	nuclear factor I/A	-0,4607	0,7266	0,0002	0,0025
ENSG00000147862	NFIB	nuclear factor I/B	0,5041	1,4182	0,0000	0,0001
ENSG00000165030	NFIL3	nuclear factor, interleukin 3 regulated	0,4233	1,3410	0,0000	0,0002
ENSG00000008441	NFIX	nuclear factor I/X (CCAAT-binding transcription factor)	0,5380	1,4520	0,0000	0,0000
ENSG00000109320	NFKB1	nuclear factor of kappa light polypeptide gene enhancer in B-cells 1	-0,5318	0,6917	0,0000	0,0000
ENSG00000100906	NFKBIA	nuclear factor of kappa light polypeptide gene enhancer in B-cells inhibitor, alpha	-0,4122	0,7515	0,0000	0,0000
ENSG00000146232	NFKBIE	nuclear factor of kappa light polypeptide gene enhancer in B-cells inhibitor, epsilon	-0,7802	0,5823	0,0000	0,0000
ENSG00000144802	NFKBIZ	nuclear factor of kappa light polypeptide gene enhancer in B-cells inhibitor, zeta	-0,6065	0,6568	0,0004	0,0046
ENSG00000120837	NFYB	nuclear transcription factor Y, beta	-0,3864	0,7650	0,0001	0,0011
ENSG00000066248	NGEF	neuronal guanine nucleotide exchange factor	0,7871	1,7256	0,0000	0,0000
ENSG00000064300	NGFR	nerve growth factor receptor	0,5318	1,4458	0,0002	0,0019
ENSG00000155438	NIFK	nucleolar protein interacting with the FHA domain of MKI67	-0,5729	0,6723	0,0000	0,0000
ENSG00000131669	NINJ1	ninjurin 1	0,4396	1,3563	0,0008	0,0076
ENSG00000132603	NIP7	NIP7, nucleolar pre-rRNA processing protein	-0,7054	0,6133	0,0000	0,0000
ENSG00000140157	NIPA2	non imprinted in Prader-Willi/Angelman syndrome 2	-0,8018	0,5736	0,0000	0,0000
ENSG00000163293	NIPAL1	NIPA-like domain containing 1	-0,7362	0,6003	0,0000	0,0000

ENSG00000104361	NIPAL2	NIPA-like domain containing 2	-0,4991	0,7076	0,0002	0,0020
ENSG00000172548	NIPAL4	NIPA-like domain containing 4	-1,2509	0,4202	0,0000	0,0000
ENSG00000184117	NIPSNAP1	nipsnap homolog 1 (C. elegans)	0,5783	1,4931	0,0000	0,0000
ENSG00000158793	NIT1	nitrilase 1	0,5371	1,4511	0,0000	0,0000
ENSG00000140807	NKD1	naked cuticle homolog 1 (Drosophila)	0,6214	1,5384	0,0000	0,0000
ENSG00000145506	NKD2	naked cuticle homolog 2 (Drosophila)	1,0421	2,0592	0,0000	0,0000
ENSG00000169992	NLGN2	neuroligin 2	0,5343	1,4482	0,0000	0,0000
ENSG00000196338	NLGN3	neuroligin 3	0,9647	1,9517	0,0002	0,0021
ENSG00000167984	NLRC3	NLR family, CARD domain containing 3	0,8298	1,7774	0,0006	0,0062
ENSG00000091592	NLRP1	NLR family, pyrin domain containing 1	1,2860	2,4385	0,0000	0,0000
ENSG00000160703	NLRX1	NLR family member X1	0,5819	1,4968	0,0000	0,0002
ENSG00000239672	NME1	NME/NM23 nucleoside diphosphate kinase 1	-0,4883	0,7129	0,0000	0,0002
ENSG00000103024	NME3	NME/NM23 nucleoside diphosphate kinase 3	0,9364	1,9137	0,0000	0,0001
ENSG00000103202	NME4	NME/NM23 nucleoside diphosphate kinase 4	0,5336	1,4475	0,0000	0,0000
ENSG00000157064	NMNAT2	nicotinamide nucleotide adenylyltransferase 2	-0,4146	0,7502	0,0003	0,0030
ENSG00000153406	NMRAL1	NmrA-like family domain containing 1	0,4906	1,4050	0,0000	0,0000
ENSG00000109255	NMU	neuromedin U	-0,5196	0,6976	0,0001	0,0014
ENSG00000173145	NOC3L	NOC3-like DNA replication regulator	-0,6650	0,6307	0,0000	0,0000
ENSG00000167207	NOD2	nucleotide-binding oligomerization domain containing 2	0,4601	1,3756	0,0000	0,0000
ENSG00000115761	NOL10	nucleolar protein 10	-0,4864	0,7138	0,0000	0,0000
ENSG00000130935	NOL11	nucleolar protein 11	-0,4564	0,7288	0,0000	0,0002
ENSG00000140939	NOL3	nucleolar protein 3 (apoptosis repressor with CARD domain)	0,4137	1,3321	0,0000	0,0007
ENSG00000165271	NOL6	nucleolar protein 6 (RNA-associated)	-0,5063	0,7040	0,0000	0,0000
ENSG00000198000	NOL8	nucleolar protein 8	-0,4970	0,7086	0,0000	0,0000
ENSG00000166197	NOLC1	nucleolar and coiled-body phosphoprotein 1	-0,5073	0,7035	0,0000	0,0000
ENSG00000103512	NOMO1	NODAL modulator 1	-0,4199	0,7475	0,0000	0,0003
ENSG00000048162	NOP16	NOP16 nucleolar protein	-0,5494	0,6833	0,0000	0,0000
ENSG00000101361	NOP56	NOP56 ribonucleoprotein	-0,5184	0,6981	0,0000	0,0000
ENSG00000055044	NOP58	NOP58 ribonucleoprotein	-0,4449	0,7347	0,0000	0,0002
ENSG00000196943	NOP9	NOP9 nucleolar protein	-0,4648	0,7246	0,0000	0,0000
ENSG00000185269	NOTUM	notum pectinacetylesterase homolog (Drosophila)	0,9134	1,8835	0,0000	0,0004

ENSG00000119655	NPC2	Niemann-Pick disease, type C2	-0,7041	0,6138	0,0000	0,0000
ENSG00000107281	NPDC1	neural proliferation, differentiation and control, 1	0,4920	1,4064	0,0002	0,0027
ENSG00000215440	NPEPL1	aminopeptidase-like 1	0,4600	1,3756	0,0004	0,0041
ENSG00000144061	NPHP1	nephronophthisis 1 (juvenile)	0,5211	1,4350	0,0002	0,0020
ENSG00000161270	NPHS1	nephrosis 1, congenital, Finnish type (nephrin)	0,8967	1,8618	0,0001	0,0013
ENSG00000181163	NPM1	nucleophosmin (nucleolar phosphoprotein B23, numatrin)	-0,4572	0,7284	0,0000	0,0005
ENSG00000168743	NPNT	nephronectin	1,2528	2,3830	0,0000	0,0000
ENSG00000126368	NR1D1	nuclear receptor subfamily 1, group D, member 1	1,0727	2,1033	0,0000	0,0000
ENSG00000237187	NR2F1-AS1	NR2F1 antisense RNA 1	-0,4788	0,7176	0,0011	0,0099
ENSG00000185551	NR2F2	nuclear receptor subfamily 2, group F, member 2	-0,8872	0,5407	0,0000	0,0000
ENSG00000247809	NR2F2-AS1	NR2F2 antisense RNA 1	-0,7222	0,6062	0,0003	0,0029
ENSG00000153234	NR4A2	nuclear receptor subfamily 4, group A, member 2	0,6870	1,6099	0,0000	0,0002
ENSG00000185189	NRBP2	nuclear receptor binding protein 2	0,6154	1,5320	0,0000	0,0001
ENSG00000134986	NREP	neuronal regeneration related protein	0,7820	1,7196	0,0000	0,0000
ENSG00000180530	NRIP1	nuclear receptor interacting protein 1	-0,8064	0,5718	0,0000	0,0000
ENSG00000124785	NRN1	neuritin 1	0,9796	1,9719	0,0000	0,0000
ENSG00000099250	NRP1	neuropilin 1	-0,9289	0,5253	0,0000	0,0000
ENSG00000125841	NRSN2	neurensin 2	0,3809	1,3021	0,0000	0,0002
ENSG00000147383	NSDHL	NAD(P) dependent steroid dehydrogenase-like	-0,4265	0,7441	0,0000	0,0000
ENSG00000073969	NSF	N-ethylmaleimide-sensitive factor	-0,4032	0,7562	0,0002	0,0020
ENSG00000165802	NSMF	NMDA receptor synaptonuclear signaling and neuronal migration factor	0,5020	1,4162	0,0000	0,0000
ENSG00000037474	NSUN2	NOP2/Sun RNA methyltransferase family, member 2	-0,4805	0,7167	0,0000	0,0000
ENSG00000144130	NT5DC4	5'-nucleotidase domain containing 4	0,5746	1,4893	0,0000	0,0002
ENSG00000135318	NT5E	5'-nucleotidase, ecto (CD73)	-1,6011	0,3296	0,0000	0,0000
ENSG00000205309	NT5M	5',3'-nucleotidase, mitochondrial	0,6808	1,6030	0,0000	0,0001
ENSG00000185652	NTF3	neurotrophin 3	-1,0463	0,4842	0,0000	0,0000
ENSG00000182667	NTM	neurotrimin	1,0089	2,0124	0,0000	0,0000
ENSG00000196358	NTNG2	netrin G2	0,9081	1,8766	0,0000	0,0000
ENSG00000148053	NTRK2	neurotrophic tyrosine kinase, receptor, type 2	-0,6725	0,6274	0,0000	0,0000
ENSG00000070081	NUCB2	nucleobindin 2	0,4778	1,3926	0,0000	0,0000
ENSG00000069275	NUCKS1	nuclear casein kinase and cyclin-dependent kinase substrate 1	-0,4901	0,7120	0,0000	0,0000

ENSG00000120526	NUDCD1	NudC domain containing 1	-0,5699	0,6737	0,0000	0,0000
ENSG00000106268	NUDT1	nudix (nucleoside diphosphate linked moiety X)-type motif 1	0,5491	1,4632	0,0000	0,0001
ENSG00000136159	NUDT15	nudix (nucleoside diphosphate linked moiety X)-type motif 15	-0,7160	0,6088	0,0000	0,0000
ENSG00000186364	NUDT17	nudix (nucleoside diphosphate linked moiety X)-type motif 17	0,7344	1,6638	0,0000	0,0000
ENSG00000140876	NUDT7	nudix (nucleoside diphosphate linked moiety X)-type motif 7	0,5157	1,4297	0,0009	0,0089
ENSG00000083635	NUFIP1	nuclear fragile X mental retardation protein interacting protein 1	-0,5722	0,6726	0,0000	0,0000
ENSG00000105245	NUMBL	numb homolog (Drosophila)-like	0,5946	1,5101	0,0000	0,0000
ENSG00000124789	NUP153	nucleoporin 153kDa	-0,4191	0,7479	0,0000	0,0000
ENSG00000163002	NUP35	nucleoporin 35kDa	-0,4589	0,7275	0,0000	0,0005
ENSG00000139496	NUP58	nucleoporin 58kDa	-0,4352	0,7396	0,0000	0,0000
ENSG00000136243	NUPL2	nucleoporin like 2	-0,7391	0,5991	0,0000	0,0000
ENSG00000153989	NUS1	NUS1 dehydrodolichyl diphosphate synthase subunit	-0,5213	0,6967	0,0000	0,0000
ENSG00000182575	NXPH3	neurexophilin 3	1,3719	2,5881	0,0000	0,0000
ENSG00000111335	OAS2	2'-5'-oligoadenylate synthetase 2, 69/71kDa	0,3914	1,3116	0,0000	0,0006
ENSG00000111331	OAS3	2'-5'-oligoadenylate synthetase 3, 100kDa	-0,4733	0,7203	0,0000	0,0000
ENSG00000135114	OASL	2'-5'-oligoadenylate synthetase-like	0,8850	1,8467	0,0002	0,0021
ENSG00000107960	OBFC1	oligonucleotide/oligosaccharide-binding fold containing 1	0,3937	1,3138	0,0002	0,0020
ENSG00000124006	OBSL1	obscurin-like 1	0,4409	1,3574	0,0002	0,0027
ENSG00000099330	OCEL1	occludin/ELL domain containing 1	0,9396	1,9179	0,0000	0,0000
ENSG00000115758	ODC1	ornithine decarboxylase 1	-0,5836	0,6673	0,0000	0,0000
ENSG00000197444	OGDHL	oxoglutarate dehydrogenase-like	0,5309	1,4448	0,0000	0,0000
ENSG00000119900	OGFRL1	opioid growth factor receptor-like 1	-0,4879	0,7131	0,0000	0,0001
ENSG00000130558	OLFM1	olfactomedin 1	-0,6353	0,6438	0,0000	0,0000
ENSG00000185585	OLFML2A	olfactomedin-like 2A	0,8802	1,8406	0,0000	0,0000
ENSG00000116774	OLFML3	olfactomedin-like 3	0,9431	1,9227	0,0002	0,0026
ENSG00000198836	OPA1	optic atrophy 1 (autosomal dominant)	-0,4181	0,7484	0,0000	0,0000
ENSG00000178814	OPLAH	5-oxoprolinase (ATP-hydrolysing)	1,0086	2,0120	0,0000	0,0000
ENSG00000125510	OPRL1	opiate receptor-like 1	0,9605	1,9459	0,0000	0,0003
ENSG00000183251	OR51B4	olfactory receptor, family 51, subfamily B, member 4	1,6260	3,0866	0,0000	0,0000
ENSG00000175938	ORAI3	ORAI calcium release-activated calcium modulator 3	0,4907	1,4051	0,0000	0,0000
ENSG00000164815	ORC5	origin recognition complex, subunit 5	-0,4116	0,7518	0,0000	0,0003

ENSG00000184792	OSBP2	oxysterol binding protein 2	0,7144	1,6408	0,0000	0,0000
ENSG00000021762	OSBPL5	oxysterol binding protein-like 5	1,1804	2,2663	0,0000	0,0000
ENSG00000116885	OSCP1	organic solute carrier partner 1	0,8533	1,8067	0,0000	0,0000
ENSG00000223891	OSER1-AS1	OSER1 antisense RNA 1 (head to head)	0,5422	1,4562	0,0000	0,0001
ENSG00000140961	OSGIN1	oxidative stress induced growth inhibitor 1	0,6282	1,5456	0,0000	0,0007
ENSG00000081087	OSTM1	osteopetrosis associated transmembrane protein 1	-0,4304	0,7421	0,0000	0,0001
ENSG00000089723	OTUB2	OTU deubiquitinase, ubiquitin aldehyde binding 2	-0,4209	0,7470	0,0001	0,0015
ENSG00000165312	OTUD1	OTU deubiquitinase 1	0,4905	1,4049	0,0003	0,0031
ENSG00000164164	OTUD4	OTU deubiquitinase 4	-0,3915	0,7623	0,0000	0,0001
ENSG00000155100	OTUD6B	OTU domain containing 6B	-0,6011	0,6593	0,0000	0,0000
ENSG00000083720	OXCT1	3-oxoacid CoA transferase 1	-0,6981	0,6164	0,0000	0,0000
ENSG00000108405	P2RX1	purinergic receptor P2X, ligand gated ion channel, 1	1,2804	2,4290	0,0000	0,0000
ENSG00000083454	P2RX5	purinergic receptor P2X, ligand gated ion channel, 5	-0,5168	0,6989	0,0000	0,0003
ENSG00000099957	P2RX6	purinergic receptor P2X, ligand gated ion channel, 6	1,2458	2,3715	0,0000	0,0000
ENSG00000089041	P2RX7	purinergic receptor P2X, ligand gated ion channel, 7	1,3688	2,5825	0,0000	0,0000
ENSG00000101104	PABPC1L	poly(A) binding protein, cytoplasmic 1-like	0,4161	1,3343	0,0001	0,0018
ENSG00000165912	PACSIN3	protein kinase C and casein kinase substrate in neurons 3	0,3950	1,3149	0,0006	0,0062
ENSG00000142619	PADI3	peptidyl arginine deiminase, type III	0,6593	1,5793	0,0000	0,0000
ENSG00000122133	PAEP	progestagen-associated endometrial protein	1,4327	2,6995	0,0000	0,0000
ENSG00000149269	PAK1	p21 protein (Cdc42/Rac)-activated kinase 1	0,6520	1,5714	0,0000	0,0000
ENSG00000111845	PAK1IP1	PAK1 interacting protein 1	-0,5501	0,6830	0,0000	0,0000
ENSG00000145730	PAM	peptidylglycine alpha-amidating monooxygenase	0,4962	1,4105	0,0000	0,0000
ENSG00000135473	PAN2	PAN2 poly(A) specific ribonuclease subunit	0,5605	1,4748	0,0000	0,0000
ENSG00000261485	PAN3-AS1	PAN3 antisense RNA 1	-0,7869	0,5796	0,0000	0,0003
ENSG00000110218	PANX1	pannexin 1	-0,6964	0,6171	0,0000	0,0000
ENSG00000090060	PAPOLA	poly(A) polymerase alpha	-0,6351	0,6439	0,0000	0,0000
ENSG00000198682	PAPSS2	3'-phosphoadenosine 5'-phosphosulfate synthase 2	-0,8830	0,5422	0,0000	0,0000
ENSG00000160781	PAQR6	progestin and adipoQ receptor family member VI	0,5886	1,5038	0,0008	0,0074
ENSG00000170915	PAQR8	progestin and adipoQ receptor family member VIII	0,9587	1,9435	0,0000	0,0000
ENSG00000102981	PARD6A	par-6 family cell polarity regulator alpha	0,6714	1,5926	0,0007	0,0068
ENSG00000169116	PARM1	prostate androgen-regulated mucin-like protein 1	1,0471	2,0663	0,0000	0,0000

ENSG00000143799	PARP1	poly (ADP-ribose) polymerase 1	-0,4217	0,7465	0,0000	0,0000
ENSG00000178685	PARP10	poly (ADP-ribose) polymerase family, member 10	0,6373	1,5554	0,0001	0,0015
ENSG00000138617	PARP16	poly (ADP-ribose) polymerase family, member 16	0,3913	1,3115	0,0003	0,0033
ENSG00000041880	PARP3	poly (ADP-ribose) polymerase family, member 3	0,4209	1,3387	0,0000	0,0002
ENSG00000102699	PARP4	poly (ADP-ribose) polymerase family, member 4	-0,8754	0,5451	0,0000	0,0000
ENSG00000224976	PARP4P2	poly (ADP-ribose) polymerase family, member 4 pseudogene 2	-0,8731	0,5460	0,0001	0,0008
ENSG00000151883	PARP8	poly (ADP-ribose) polymerase family, member 8	-0,4975	0,7083	0,0000	0,0000
ENSG00000196092	PAX5	paired box 5	0,4520	1,3679	0,0003	0,0031
ENSG00000009709	PAX7	paired box 7	1,5064	2,8410	0,0000	0,0000
ENSG00000189223	PAX8-AS1	PAX8 antisense RNA 1	-0,4163	0,7493	0,0000	0,0000
ENSG00000273344	PAXIP1-AS1	PAXIP1 antisense RNA 1 (head to head)	0,4478	1,3640	0,0000	0,0002
ENSG00000102390	PBDC1	polysaccharide biosynthesis domain containing 1	-0,4438	0,7352	0,0000	0,0000
ENSG00000185630	PBX1	pre-B-cell leukemia homeobox 1	-0,8185	0,5670	0,0005	0,0055
ENSG00000163346	PBXIP1	pre-B-cell leukemia homeobox interacting protein 1	0,9408	1,9196	0,0000	0,0000
ENSG00000090097	PCBP4	poly(rC) binding protein 4	0,3811	1,3023	0,0003	0,0030
ENSG00000175198	PCCA	propionyl CoA carboxylase, alpha polypeptide	-0,4375	0,7384	0,0002	0,0027
ENSG00000197479	PCDHB11	protocadherin beta 11	0,8839	1,8454	0,0010	0,0089
ENSG00000187372	PCDHB13	protocadherin beta 13	0,7492	1,6809	0,0010	0,0096
ENSG00000240184	PCDHGC3	protocadherin gamma subfamily C, 3	-0,6153	0,6528	0,0000	0,0000
ENSG00000132635	PCED1A	PC-esterase domain containing 1A	0,5043	1,4184	0,0000	0,0001
ENSG00000179715	PCED1B	PC-esterase domain containing 1B	1,5470	2,9222	0,0000	0,0000
ENSG00000126226	PCID2	PCI domain containing 2	-0,7582	0,5913	0,0000	0,0000
ENSG00000100982	PCIF1	PDX1 C-terminal inhibiting factor 1	0,5574	1,4716	0,0000	0,0000
ENSG00000124253	PCK1	phosphoenolpyruvate carboxykinase 1 (soluble)	1,1173	2,1695	0,0000	0,0006
ENSG00000100889	PCK2	phosphoenolpyruvate carboxykinase 2 (mitochondrial)	0,5449	1,4589	0,0000	0,0000
ENSG00000186472	PCLO	piccolo presynaptic cytomatrix protein	-1,0835	0,4719	0,0000	0,0000
ENSG00000168300	PCMTD1	protein-L-isoaspartate (D-aspartate) O-methyltransferase domain containing 1	0,6115	1,5279	0,0000	0,0000
ENSG00000183036	PCP4	Purkinje cell protein 4	2,7178	6,5787	0,0000	0,0000
ENSG00000115257	PCSK4	proprotein convertase subtilisin/kexin type 4	0,9902	1,9865	0,0001	0,0007
ENSG00000140479	PCSK6	proprotein convertase subtilisin/kexin type 6	-0,6284	0,6469	0,0000	0,0000
ENSG00000145882	PCYOX1L	prenylcysteine oxidase 1 like	0,7782	1,7150	0,0000	0,0000

ENSG00000150593	PDCD4	programmed cell death 4 (neoplastic transformation inhibitor)	0,4043	1,3235	0,0000	0,0000
ENSG00000186642	PDE2A	phosphodiesterase 2A, cGMP-stimulated	0,6423	1,5608	0,0003	0,0036
ENSG00000152270	PDE3B	phosphodiesterase 3B, cGMP-inhibited	-0,8676	0,5481	0,0000	0,0000
ENSG00000160191	PDE9A	phosphodiesterase 9A	-1,0734	0,4752	0,0000	0,0000
ENSG00000145431	PDGFC	platelet derived growth factor C	1,1743	2,2569	0,0000	0,0000
ENSG00000113721	PDGFRB	platelet-derived growth factor receptor, beta polypeptide	0,8929	1,8569	0,0008	0,0074
ENSG00000083642	PDS5B	PDS5 cohesin associated factor B	-0,4595	0,7272	0,0000	0,0000
ENSG00000148459	PDSS1	prenyl (decaprenyl) diphosphate synthase, subunit 1	-0,6245	0,6487	0,0000	0,0000
ENSG00000139515	PDX1	pancreatic and duodenal homeobox 1	-0,5649	0,6760	0,0000	0,0006
ENSG00000186862	PDZD7	PDZ domain containing 7	0,8434	1,7942	0,0000	0,0000
ENSG00000121440	PDZRN3	PDZ domain containing ring finger 3	0,5918	1,5071	0,0000	0,0000
ENSG00000162734	PEA15	phosphoprotein enriched in astrocytes 15	-0,3978	0,7590	0,0000	0,0001
ENSG00000187800	PEAR1	platelet endothelial aggregation receptor 1	0,6430	1,5616	0,0000	0,0004
ENSG00000139946	PELI2	pellino E3 ubiquitin protein ligase family member 2	-0,6567	0,6343	0,0000	0,0000
ENSG00000174516	PELI3	pellino E3 ubiquitin protein ligase family member 3	0,5794	1,4942	0,0001	0,0012
ENSG00000133027	PEMT	phosphatidylethanolamine N-methyltransferase	0,5015	1,4157	0,0002	0,0021
ENSG00000179094	PER1	period circadian clock 1	1,3530	2,5545	0,0000	0,0000
ENSG00000112378	PERP	PERP, TP53 apoptosis effector	-0,5820	0,6680	0,0000	0,0000
ENSG00000114268	PFKFB4	6-phosphofructo-2-kinase/fructose-2,6-biphosphatase 4	0,7575	1,6905	0,0000	0,0000
ENSG00000070087	PFN2	profilin 2	-0,4125	0,7513	0,0001	0,0007
ENSG00000161395	PGAP3	post-GPI attachment to proteins 3	0,4569	1,3726	0,0006	0,0061
ENSG00000096088	PGC	progastricsin (pepsinogen C)	0,8877	1,8502	0,0004	0,0044
ENSG00000119630	PGF	placental growth factor	0,5958	1,5113	0,0007	0,0069
ENSG00000169299	PGM2	phosphoglucomutase 2	-0,4547	0,7297	0,0000	0,0001
ENSG00000101856	PGRMC1	progesterone receptor membrane component 1	-0,5380	0,6887	0,0000	0,0000
ENSG00000112511	PHF1	PHD finger protein 1	0,6075	1,5237	0,0000	0,0000
ENSG00000106443	PHF14	PHD finger protein 14	-0,6423	0,6407	0,0000	0,0000
ENSG00000135365	PHF21A	PHD finger protein 21A	0,6918	1,6154	0,0000	0,0000
ENSG00000226057	PHF2P2	PHD finger protein 2 pseudogene 2	1,2885	2,4427	0,0000	0,0000
ENSG00000010318	PHF7	PHD finger protein 7	0,3815	1,3027	0,0007	0,0068
ENSG00000181649	PHLDA2	pleckstrin homology-like domain, family A, member 2	-0,5111	0,7017	0,0004	0,0045

ENSG00000144824	PHLDB2	pleckstrin homology-like domain, family B, member 2	-0,3937	0,7612	0,0000	0,0001
ENSG00000081913	PHLPP1	PH domain and leucine rich repeat protein phosphatase 1	0,3951	1,3150	0,0001	0,0008
ENSG00000040199	PHLPP2	PH domain and leucine rich repeat protein phosphatase 2	-0,4041	0,7557	0,0000	0,0002
ENSG00000054148	PHPT1	phosphohistidine phosphatase 1	0,4193	1,3373	0,0002	0,0021
ENSG00000038210	PI4K2B	phosphatidylinositol 4-kinase type 2 beta	-0,4383	0,7380	0,0000	0,0003
ENSG00000105229	PIAS4	protein inhibitor of activated STAT, 4	0,4818	1,3964	0,0001	0,0008
ENSG00000083535	PIBF1	progesterone immunomodulatory binding factor 1	-0,4457	0,7342	0,0000	0,0005
ENSG00000173947	PIFO	primary cilia formation	0,9013	1,8677	0,0005	0,0051
ENSG00000101464	PIGU	phosphatidylinositol glycan anchor biosynthesis, class U	-0,3893	0,7635	0,0001	0,0007
ENSG00000277161	PIGW	phosphatidylinositol glycan anchor biosynthesis, class W	-0,7840	0,5808	0,0000	0,0000
ENSG00000155629	PIK3AP1	phosphoinositide-3-kinase adaptor protein 1	-0,8254	0,5643	0,0000	0,0000
ENSG00000100100	PIK3IP1	phosphoinositide-3-kinase interacting protein 1	1,6895	3,2255	0,0000	0,0000
ENSG00000228839	PIK3IP1-AS1	PIK3IP1 antisense RNA 1 (head to head)	0,9295	1,9046	0,0006	0,0061
ENSG00000145675	PIK3R1	phosphoinositide-3-kinase, regulatory subunit 1 (alpha)	-0,6235	0,6491	0,0000	0,0000
ENSG00000198355	PIM3	Pim-3 proto-oncogene, serine/threonine kinase	0,4646	1,3799	0,0001	0,0011
ENSG00000158828	PINK1	PTEN induced putative kinase 1	0,5325	1,4465	0,0000	0,0000
ENSG00000117242	PINK1-AS	PINK1 antisense RNA	-0,6003	0,6596	0,0000	0,0000
ENSG00000107242	PIP5K1B	phosphatidylinositol-4-phosphate 5-kinase, type I, beta	-0,8859	0,5412	0,0000	0,0000
ENSG00000167103	PIP5KL1	phosphatidylinositol-4-phosphate 5-kinase-like 1	1,1579	2,2313	0,0000	0,0000
ENSG00000180957	PITPNB	phosphatidylinositol transfer protein, beta	-0,4737	0,7201	0,0000	0,0000
ENSG00000154217	PITPNC1	phosphatidylinositol transfer protein, cytoplasmic 1	-0,4606	0,7267	0,0000	0,0000
ENSG00000090975	PITPNM2	phosphatidylinositol transfer protein, membrane-associated 2	0,4960	1,4103	0,0002	0,0027
ENSG00000091622	PITPNM3	PITPNM family member 3	0,8899	1,8531	0,0000	0,0000
ENSG00000164093	PITX2	paired-like homeodomain 2	0,7123	1,6384	0,0004	0,0040
ENSG00000125207	PIWIL1	piwi-like RNA-mediated gene silencing 1	-1,3850	0,3829	0,0000	0,0000
ENSG00000198961	PJA2	praja ring finger 2, E3 ubiquitin protein ligase	0,3953	1,3152	0,0000	0,0000
ENSG00000008710	PKD1	polycystic kidney disease 1 (autosomal dominant)	0,6304	1,5480	0,0000	0,0002
ENSG00000166473	PKD1L2	polycystic kidney disease 1-like 2 (gene/pseudogene)	0,8091	1,7521	0,0002	0,0022
ENSG00000118762	PKD2	polycystic kidney disease 2 (autosomal dominant)	-0,4818	0,7161	0,0000	0,0000
ENSG00000170927	PKHD1	polycystic kidney and hepatic disease 1 (autosomal recessive)	0,9587	1,9435	0,0000	0,0000
ENSG00000260804	PKI55	DKFZp434H1419	-1,1800	0,4413	0,0000	0,0000

ENSG00000127564	PKMYT1	protein kinase, membrane associated tyrosine/threonine 1	0,4950	1,4094	0,0000	0,0003
ENSG00000081277	PKP1	plakophilin 1	0,5702	1,4848	0,0000	0,0001
ENSG00000159337	PLA2G4D	phospholipase A2, group IVD (cytosolic)	0,9572	1,9416	0,0000	0,0000
ENSG00000168907	PLA2G4F	phospholipase A2, group IVF	1,6325	3,1005	0,0000	0,0000
ENSG00000184381	PLA2G6	phospholipase A2, group VI (cytosolic, calcium-independent)	0,7202	1,6474	0,0000	0,0003
ENSG00000170965	PLAC1	placenta-specific 1	0,9031	1,8700	0,0002	0,0020
ENSG00000181690	PLAG1	pleiomorphic adenoma gene 1	0,4576	1,3733	0,0001	0,0014
ENSG00000104368	PLAT	plasminogen activator, tissue	-0,4794	0,7173	0,0009	0,0083
ENSG00000011422	PLAUR	plasminogen activator, urokinase receptor	-0,5572	0,6796	0,0000	0,0000
ENSG00000163803	PLB1	phospholipase B1	0,7388	1,6688	0,0000	0,0000
ENSG00000187091	PLCD1	phospholipase C, delta 1	0,9170	1,8881	0,0000	0,0000
ENSG00000149527	PLCH2	phospholipase C, eta 2	1,3480	2,5456	0,0000	0,0000
ENSG00000075651	PLD1	phospholipase D1, phosphatidylcholine-specific	0,5936	1,5090	0,0000	0,0000
ENSG00000105223	PLD3	phospholipase D family, member 3	0,7724	1,7082	0,0000	0,0000
ENSG00000115956	PLEK	pleckstrin	-0,6106	0,6549	0,0000	0,0000
ENSG00000021300	PLEKHB1	pleckstrin homology domain containing, family B (evectins) member 1	1,3020	2,4658	0,0000	0,0000
ENSG00000175985	PLEKHD1	pleckstrin homology domain containing, family D (with coiled-coil domains) membe	0,8138	1,7579	0,0002	0,0025
ENSG00000068137	PLEKHH3	pleckstrin homology domain containing, family H (with MyTH4 domain) member 3	0,9100	1,8790	0,0000	0,0000
ENSG00000225190	PLEKHM1	pleckstrin homology domain containing, family M (with RUN domain) member 1	0,5454	1,4595	0,0000	0,0006
ENSG00000178385	PLEKHM3	pleckstrin homology domain containing, family M, member 3	0,9615	1,9473	0,0000	0,0000
ENSG00000145632	PLK2	polo-like kinase 2	-0,6202	0,6506	0,0000	0,0000
ENSG00000162407	PLPP3	phospholipid phosphatase 3	-0,4283	0,7431	0,0004	0,0045
ENSG00000105520	PLPPR2	phospholipid phosphatase related 2	0,6215	1,5385	0,0000	0,0005
ENSG00000102024	PLS3	plastin 3	-0,3942	0,7609	0,0000	0,0001
ENSG00000221866	PLXNA4	plexin A4	0,8548	1,8085	0,0008	0,0079
ENSG00000136040	PLXNC1	plexin C1	-0,7205	0,6069	0,0002	0,0023
ENSG00000124225	PMEPA1	prostate transmembrane protein, androgen induced 1	-0,9484	0,5182	0,0000	0,0000
ENSG00000100417	PMM1	phosphomannomutase 1	0,4946	1,4090	0,0000	0,0002
ENSG00000140650	PMM2	phosphomannomutase 2	-0,4496	0,7323	0,0000	0,0004
ENSG00000240694	PNMA2	paraneoplastic Ma antigen 2	-0,8564	0,5523	0,0000	0,0000
ENSG00000141744	PNMT	phenylethanolamine N-methyltransferase	1,0949	2,1360	0,0001	0,0009

ENSG00000100941	PNN	pinin, desmosome associated protein	-0,4846	0,7147	0,0000	0,0000
ENSG00000115946	PNO1	partner of NOB1 homolog	-0,6879	0,6207	0,0000	0,0000
ENSG00000198805	PNP	purine nucleoside phosphorylase	-0,3878	0,7643	0,0001	0,0009
ENSG00000177666	PNPLA2	patatin-like phospholipase domain containing 2	0,5327	1,4466	0,0000	0,0000
ENSG00000100344	PNPLA3	patatin-like phospholipase domain containing 3	-0,4773	0,7183	0,0008	0,0079
ENSG00000032444	PNPLA6	patatin-like phospholipase domain containing 6	0,5328	1,4467	0,0001	0,0017
ENSG00000130653	PNPLA7	patatin-like phospholipase domain containing 7	0,9156	1,8863	0,0001	0,0011
ENSG00000138035	PNPT1	polyribonucleotide nucleotidyltransferase 1	-0,4466	0,7338	0,0000	0,0001
ENSG00000146278	PNRC1	proline-rich nuclear receptor coactivator 1	0,7030	1,6279	0,0000	0,0000
ENSG00000139323	POC1B	POC1 centriolar protein B	-0,5151	0,6997	0,0000	0,0000
ENSG00000163389	POGLUT1	protein O-glucosyltransferase 1	-0,4029	0,7563	0,0006	0,0063
ENSG00000175482	POLD4	polymerase (DNA-directed), delta 4, accessory subunit	0,7973	1,7379	0,0000	0,0000
ENSG00000148229	POLE3	polymerase (DNA directed), epsilon 3, accessory subunit	-0,4720	0,7209	0,0000	0,0000
ENSG00000166169	POLL	polymerase (DNA directed), lambda	0,5256	1,4395	0,0000	0,0000
ENSG00000125630	POLR1B	polymerase (RNA) I polypeptide B, 128kDa	-0,5463	0,6848	0,0000	0,0000
ENSG00000100413	POLR3H	polymerase (RNA) III (DNA directed) polypeptide H (22.9kD)	-0,4584	0,7278	0,0000	0,0001
ENSG00000161980	POLR3K	polymerase (RNA) III (DNA directed) polypeptide K, 12.3 kDa	-0,4355	0,7394	0,0002	0,0019
ENSG00000132963	POMP	proteasome maturation protein	-0,6845	0,6222	0,0000	0,0000
ENSG00000130714	POMT1	protein-O-mannosyltransferase 1	0,3907	1,3111	0,0002	0,0020
ENSG00000009830	POMT2	protein-O-mannosyltransferase 2	-0,4060	0,7547	0,0000	0,0000
ENSG00000105854	PON2	paraoxonase 2	-0,5433	0,6862	0,0000	0,0000
ENSG00000104356	POP1	POP1 homolog, ribonuclease P/MRP subunit	-0,7906	0,5781	0,0000	0,0000
ENSG00000172336	POP7	POP7 homolog, ribonuclease P/MRP subunit	-0,4256	0,7445	0,0000	0,0003
ENSG00000128513	POT1	protection of telomeres 1	0,4541	1,3699	0,0000	0,0001
ENSG00000152192	POU4F1	POU class 4 homeobox 1	-1,6260	0,3240	0,0000	0,0000
ENSG00000184271	POU6F1	POU class 6 homeobox 1	0,5895	1,5047	0,0001	0,0011
ENSG00000186951	PPARA	peroxisome proliferator-activated receptor alpha	0,7040	1,6291	0,0000	0,0000
ENSG00000155846	PPARGC1B	peroxisome proliferator-activated receptor gamma, coactivator 1 beta	-0,7360	0,6004	0,0000	0,0000
ENSG00000143847	PPFIA4	protein tyrosine phosphatase, receptor type, f polypeptide (PTPRF), interacting pro	0,9423	1,9216	0,0000	0,0000
ENSG00000166387	PPFIBP2	PTPRF interacting protein, binding protein 2 (liprin beta 2)	0,9112	1,8806	0,0000	0,0000
ENSG00000134283	PPHLN1	periphilin 1	-0,4004	0,7576	0,0001	0,0012

ENSG00000196262	PPIA	peptidylprolyl isomerase A (cyclophilin A)	-0,4105	0,7524	0,0001	0,0008
ENSG00000219797	PPIAP9	peptidylprolyl isomerase A (cyclophilin A) pseudogene 9	-0,5935	0,6627	0,0008	0,0073
ENSG00000108179	PPIF	peptidylprolyl isomerase F	-0,5073	0,7035	0,0000	0,0000
ENSG00000145725	PPIP5K2	diphosphoinositol pentakisphosphate kinase 2	-0,4907	0,7117	0,0000	0,0000
ENSG00000118898	PPL	periplakin	0,5760	1,4907	0,0000	0,0000
ENSG00000115241	PPM1G	protein phosphatase, Mg2+/Mn2+ dependent, 1G	-0,3787	0,7692	0,0000	0,0003
ENSG00000163590	PPM1L	protein phosphatase, Mg2+/Mn2+ dependent, 1L	-0,4465	0,7338	0,0000	0,0001
ENSG00000164088	PPM1M	protein phosphatase, Mg2+/Mn2+ dependent, 1M	0,4206	1,3384	0,0000	0,0006
ENSG00000143224	PPOX	protoporphyrinogen oxidase	0,5143	1,4283	0,0000	0,0000
ENSG00000186298	PPP1CC	protein phosphatase 1, catalytic subunit, gamma isozyme	-0,5288	0,6931	0,0000	0,0000
ENSG00000087074	PPP1R15A	protein phosphatase 1, regulatory subunit 15A	0,6901	1,6134	0,0000	0,0000
ENSG00000101445	PPP1R16B	protein phosphatase 1, regulatory subunit 16B	1,0476	2,0671	0,0001	0,0017
ENSG00000162148	PPP1R32	protein phosphatase 1, regulatory subunit 32	1,3744	2,5926	0,0000	0,0000
ENSG00000104866	PPP1R37	protein phosphatase 1, regulatory subunit 37	0,4501	1,3662	0,0009	0,0085
ENSG00000173281	PPP1R3B	protein phosphatase 1, regulatory subunit 3B	1,0150	2,0209	0,0000	0,0000
ENSG00000119938	PPP1R3C	protein phosphatase 1, regulatory subunit 3C	0,9782	1,9700	0,0000	0,0000
ENSG00000137713	PPP2R1B	protein phosphatase 2, regulatory subunit A, beta	-0,4375	0,7384	0,0000	0,0000
ENSG00000092020	PPP2R3C	protein phosphatase 2, regulatory subunit B'', gamma	-0,4970	0,7086	0,0000	0,0001
ENSG00000068971	PPP2R5B	protein phosphatase 2, regulatory subunit B', beta	0,3995	1,3191	0,0011	0,0099
ENSG00000154845	PPP4R1	protein phosphatase 4, regulatory subunit 1	-0,5348	0,6903	0,0000	0,0000
ENSG00000100796	PPP4R3A	protein phosphatase 4, regulatory subunit 3A	-0,4240	0,7454	0,0000	0,0001
ENSG00000119698	PPP4R4	protein phosphatase 4, regulatory subunit 4	-0,3860	0,7652	0,0002	0,0026
ENSG00000162976	PQLC3	PQ loop repeat containing 3	0,4170	1,3351	0,0003	0,0030
ENSG00000165828	PRAP1	proline-rich acidic protein 1	1,2843	2,4357	0,0000	0,0001
ENSG00000142611	PRDM16	PR domain containing 16	0,8345	1,7832	0,0000	0,0000
ENSG00000124126	PREX1	phosphatidylinositol-3,4,5-trisphosphate-dependent Rac exchange factor 1	0,8202	1,7657	0,0000	0,0000
ENSG00000131791	PRKAB2	protein kinase, AMP-activated, beta 2 non-catalytic subunit	0,4362	1,3530	0,0000	0,0000
ENSG00000106617	PRKAG2	protein kinase, AMP-activated, gamma 2 non-catalytic subunit	-0,5335	0,6909	0,0000	0,0000
ENSG00000224424	PRKAR2A-AS1	PRKAR2A antisense RNA 1	1,2083	2,3106	0,0000	0,0000
ENSG00000005249	PRKAR2B	protein kinase, cAMP-dependent, regulatory, type II, beta	-1,1241	0,4588	0,0000	0,0000
ENSG00000126583	PRKCG	protein kinase C, gamma	-0,8602	0,5509	0,0000	0,0000

ENSG00000067606	PRKCZ	protein kinase C, zeta	0,4428	1,3593	0,0001	0,0008
ENSG00000183943	PRKX	protein kinase, X-linked	-0,8941	0,5381	0,0000	0,0000
ENSG00000167525	PROCA1	protein interacting with cyclin A1	0,5396	1,4536	0,0002	0,0022
ENSG00000162997	PRORSD1P	prolyl-tRNA synthetase associated domain containing 1, pseudogene	0,5546	1,4688	0,0003	0,0030
ENSG00000147471	PROSC	proline synthetase co-transcribed homolog (bacterial)	-0,4962	0,7090	0,0000	0,0000
ENSG00000167595	PROSER3	proline and serine rich 3	0,5125	1,4265	0,0000	0,0005
ENSG00000117707	PROX1	prospero homeobox 1	0,8966	1,8617	0,0000	0,0000
ENSG00000230461	PROX1-AS1	PROX1 antisense RNA 1	0,5727	1,4873	0,0004	0,0043
ENSG00000134186	PRPF38B	pre-mRNA processing factor 38B	-0,4975	0,7083	0,0000	0,0000
ENSG00000110844	PRPF40B	PRP40 homolog, pre-mRNA processing factor B	0,5162	1,4301	0,0000	0,0000
ENSG00000135406	PRPH	peripherin	0,8300	1,7777	0,0000	0,0002
ENSG00000068489	PRR11	proline rich 11	-0,3969	0,7595	0,0000	0,0005
ENSG00000183248	PRR36	proline rich 36	0,6527	1,5721	0,0000	0,0006
ENSG00000204469	PRRC2A	proline-rich coiled-coil 2A	0,3841	1,3051	0,0004	0,0044
ENSG00000224940	PRRT4	proline-rich transmembrane protein 4	0,9508	1,9329	0,0001	0,0015
ENSG00000112812	PRSS16	protease, serine, 16 (thymus)	0,4882	1,4027	0,0000	0,0003
ENSG00000275896	PRSS2	protease, serine, 2 (trypsin 2)	2,1131	4,3263	0,0000	0,0000
ENSG00000103355	PRSS33	protease, serine, 33	1,2703	2,4121	0,0000	0,0000
ENSG00000052344	PRSS8	protease, serine, 8	1,0239	2,0335	0,0002	0,0023
ENSG00000099256	PRTFDC1	phosphoribosyl transferase domain containing 1	-0,5884	0,6651	0,0000	0,0001
ENSG00000196415	PRTN3	proteinase 3	0,9680	1,9561	0,0004	0,0044
ENSG00000106772	PRUNE2	prune homolog 2 (Drosophila)	0,7797	1,7168	0,0000	0,0004
ENSG00000105227	PRX	periaxin	0,6290	1,5465	0,0000	0,0002
ENSG00000146005	PSD2	pleckstrin and Sec7 domain containing 2	1,9523	3,8700	0,0000	0,0000
ENSG00000231924	PSG1	pregnancy specific beta-1-glycoprotein 1	1,0987	2,1416	0,0000	0,0000
ENSG00000243130	PSG11	pregnancy specific beta-1-glycoprotein 11	1,1239	2,1793	0,0000	0,0000
ENSG00000204941	PSG5	pregnancy specific beta-1-glycoprotein 5	1,4477	2,7277	0,0000	0,0000
ENSG00000106588	PSMA2	proteasome subunit alpha 2	-0,6132	0,6537	0,0000	0,0000
ENSG00000100567	PSMA3	proteasome subunit alpha 3	-0,4880	0,7130	0,0000	0,0000
ENSG00000143106	PSMA5	proteasome subunit alpha 5	-0,4596	0,7272	0,0000	0,0001
ENSG00000100764	PSMC1	proteasome 26S subunit, ATPase 1	-0,7064	0,6128	0,0000	0,0000

ENSG00000161057	PSMC2	proteasome 26S subunit, ATPase 2	-0,6962	0,6172	0,0000	0,0000
ENSG00000087191	PSMC5	proteasome 26S subunit, ATPase 5	-0,4379	0,7382	0,0000	0,0001
ENSG00000100519	PSMC6	proteasome 26S subunit, ATPase 6	-0,5877	0,6654	0,0000	0,0000
ENSG00000173692	PSMD1	proteasome 26S subunit, non-ATPase 1	-0,4791	0,7174	0,0000	0,0000
ENSG00000197170	PSMD12	proteasome 26S subunit, non-ATPase 12	-0,4249	0,7449	0,0000	0,0001
ENSG00000115233	PSMD14	proteasome 26S subunit, non-ATPase 14	-0,4157	0,7496	0,0000	0,0001
ENSG00000175166	PSMD2	proteasome 26S subunit, non-ATPase 2	-0,3837	0,7665	0,0000	0,0003
ENSG00000226752	PSMD5-AS1	PSMD5 antisense RNA 1 (head to head)	0,3906	1,3109	0,0000	0,0005
ENSG00000163636	PSMD6	proteasome 26S subunit, non-ATPase 6	-0,3791	0,7689	0,0001	0,0018
ENSG00000131467	PSME3	proteasome activator subunit 3	-0,4782	0,7179	0,0000	0,0001
ENSG00000230487	PSMG3-AS1	PSMG3 antisense RNA 1 (head to head)	-0,4878	0,7131	0,0004	0,0043
ENSG00000204540	PSORS1C1	psoriasis susceptibility 1 candidate 1	0,5523	1,4664	0,0000	0,0005
ENSG00000121390	PSPC1	paraspeckle component 1	-0,4269	0,7439	0,0000	0,0001
ENSG00000140368	PSTPIP1	proline-serine-threonine phosphatase interacting protein 1	1,4427	2,7182	0,0000	0,0000
ENSG00000152229	PSTPIP2	proline-serine-threonine phosphatase interacting protein 2	-0,8557	0,5526	0,0000	0,0000
ENSG00000156471	PTDSS1	phosphatidylserine synthase 1	-0,6583	0,6336	0,0000	0,0000
ENSG00000165983	PTER	phosphotriesterase related	-0,4491	0,7325	0,0000	0,0001
ENSG00000125384	PTGER2	prostaglandin E receptor 2 (subtype EP2), 53kDa	-0,9557	0,5156	0,0001	0,0011
ENSG00000106853	PTGR1	prostaglandin reductase 1	-0,4021	0,7567	0,0000	0,0002
ENSG00000095303	PTGS1	prostaglandin-endoperoxide synthase 1 (prostaglandin G/H synthase and cyclooxyg	1,2577	2,3912	0,0000	0,0000
ENSG00000160801	PTH1R	parathyroid hormone 1 receptor	0,9235	1,8968	0,0000	0,0005
ENSG00000112655	PTK7	protein tyrosine kinase 7 (inactive)	0,4188	1,3368	0,0002	0,0020
ENSG00000184007	PTP4A2	protein tyrosine phosphatase type IVA, member 2	-0,4449	0,7347	0,0000	0,0000
ENSG00000184489	PTP4A3	protein tyrosine phosphatase type IVA, member 3	1,2096	2,3128	0,0000	0,0000
ENSG00000163629	PTPN13	protein tyrosine phosphatase, non-receptor type 13 (APO-1/CD95 (Fas)-associated	1,3501	2,5493	0,0000	0,0000
ENSG00000111679	PTPN6	protein tyrosine phosphatase, non-receptor type 6	0,5544	1,4685	0,0000	0,0000
ENSG00000143851	PTPN7	protein tyrosine phosphatase, non-receptor type 7	-0,8293	0,5628	0,0000	0,0000
ENSG00000142949	PTPRF	protein tyrosine phosphatase, receptor type, F	0,6399	1,5582	0,0000	0,0000
ENSG00000080031	PTPRH	protein tyrosine phosphatase, receptor type, H	0,7365	1,6661	0,0000	0,0000
ENSG00000155093	PTPRN2	protein tyrosine phosphatase, receptor type, N polypeptide 2	-0,6162	0,6524	0,0000	0,0000
ENSG00000177469	PTRF	polymerase I and transcript release factor	-0,4324	0,7410	0,0000	0,0000

ENSG00000141378	PTRH2	peptidyl-tRNA hydrolase 2	-0,5669	0,6751	0,0000	0,0000
ENSG00000080608	PUM3	pumilio RNA-binding family member 3	-0,8936	0,5383	0,0000	0,0000
ENSG00000146676	PURB	purine-rich element binding protein B	-0,5507	0,6827	0,0000	0,0000
ENSG00000177192	PUS1	pseudouridylate synthase 1	-0,4131	0,7510	0,0000	0,0006
ENSG00000091127	PUS7	pseudouridylate synthase 7 (putative)	-0,6607	0,6326	0,0000	0,0000
ENSG00000129317	PUS7L	pseudouridylate synthase 7-like	-0,5686	0,6743	0,0000	0,0000
ENSG00000177707	PVRL3	poliovirus receptor-related 3	-1,2898	0,4090	0,0000	0,0000
ENSG00000136045	PWP1	PWP1 homolog, endonuclein	-0,3878	0,7643	0,0000	0,0007
ENSG00000130508	PXDN	peroxidasin	1,0600	2,0849	0,0001	0,0014
ENSG00000183010	PYCR1	pyrroline-5-carboxylate reductase 1	0,7647	1,6990	0,0000	0,0000
ENSG00000145337	PYURF	PIGY upstream reading frame	-0,4448	0,7347	0,0001	0,0009
ENSG00000131096	PYY	peptide YY	-0,9026	0,5349	0,0002	0,0025
ENSG00000237575	PYY2	peptide YY, 2 (pseudogene)	-0,8438	0,5572	0,0003	0,0032
ENSG00000112531	QKI	QKI, KH domain containing, RNA binding	-0,8607	0,5507	0,0000	0,0000
ENSG00000103485	QPRT	quinolinate phosphoribosyltransferase	0,4683	1,3834	0,0000	0,0000
ENSG00000151576	QTRTD1	queuine tRNA-ribosyltransferase domain containing 1	-0,5046	0,7048	0,0000	0,0000
ENSG00000179912	R3HDM2	R3H domain containing 2	0,4726	1,3876	0,0000	0,0000
ENSG00000131242	RAB11FIP4	RAB11 family interacting protein 4 (class II)	0,7664	1,7011	0,0000	0,0000
ENSG00000139832	RAB20	RAB20, member RAS oncogene family	-0,6547	0,6352	0,0000	0,0000
ENSG00000167964	RAB26	RAB26, member RAS oncogene family	1,0868	2,1240	0,0000	0,0000
ENSG00000041353	RAB27B	RAB27B, member RAS oncogene family	-0,6190	0,6511	0,0004	0,0044
ENSG00000137502	RAB30	RAB30, member RAS oncogene family	0,5860	1,5010	0,0000	0,0000
ENSG00000168461	RAB31	RAB31, member RAS oncogene family	-0,6907	0,6196	0,0000	0,0000
ENSG00000179331	RAB39A	RAB39A, member RAS oncogene family	-1,1415	0,4533	0,0000	0,0001
ENSG00000155961	RAB39B	RAB39B, member RAS oncogene family	0,7648	1,6991	0,0000	0,0000
ENSG00000105649	RAB3A	RAB3A, member RAS oncogene family	0,9123	1,8821	0,0000	0,0000
ENSG00000169213	RAB3B	RAB3B, member RAS oncogene family	-0,4321	0,7412	0,0000	0,0000
ENSG00000197562	RAB40C	RAB40C, member RAS oncogene family	0,5417	1,4557	0,0002	0,0020
ENSG00000154917	RAB6B	RAB6B, member RAS oncogene family	0,7481	1,6796	0,0000	0,0000
ENSG00000276600	RAB7B	RAB7B, member RAS oncogene family	1,4672	2,7648	0,0000	0,0000
ENSG00000136933	RABEPK	Rab9 effector protein with kelch motifs	-0,4912	0,7114	0,0000	0,0001

ENSG00000137955	RABGGTB	Rab geranylgeranyltransferase, beta subunit	-0,4308	0,7418	0,0000	0,0002
ENSG00000144840	RABL3	RAB, member of RAS oncogene family-like 3	-0,4118	0,7517	0,0002	0,0026
ENSG00000136238	RAC1	ras-related C3 botulinum toxin substrate 1 (rho family, small GTP binding protein R	-0,4734	0,7203	0,0000	0,0000
ENSG00000244588	RAD21L1	RAD21 cohesin complex component like 1	-0,6829	0,6229	0,0003	0,0029
ENSG00000245849	RAD51-AS1	RAD51 antisense RNA 1 (head to head)	0,6263	1,5436	0,0000	0,0003
ENSG00000172613	RAD9A	RAD9 checkpoint clamp component A	0,4004	1,3199	0,0005	0,0055
ENSG00000164520	RAET1E	retinoic acid early transcript 1E	-0,7319	0,6021	0,0000	0,0000
ENSG00000155918	RAET1L	retinoic acid early transcript 1L	1,2697	2,4110	0,0000	0,0000
ENSG00000039560	RAI14	retinoic acid induced 14	-0,4614	0,7263	0,0000	0,0000
ENSG00000131831	RAI2	retinoic acid induced 2	-0,9965	0,5012	0,0000	0,0000
ENSG00000160271	RALGDS	ral guanine nucleotide dissociation stimulator	0,5886	1,5038	0,0000	0,0000
ENSG00000136828	RALGPS1	Ral GEF with PH domain and SH3 binding motif 1	0,8119	1,7555	0,0000	0,0000
ENSG00000132329	RAMP1	receptor (G protein-coupled) activity modifying protein 1	0,5528	1,4669	0,0010	0,0090
ENSG00000132341	RAN	RAN, member RAS oncogene family	-0,5503	0,6829	0,0000	0,0000
ENSG00000137040	RANBP6	RAN binding protein 6	-0,5997	0,6599	0,0000	0,0000
ENSG00000076864	RAP1GAP	RAP1 GTPase activating protein	1,2025	2,3013	0,0000	0,0000
ENSG00000125249	RAP2A	RAP2A, member of RAS oncogene family	-0,7490	0,5950	0,0000	0,0000
ENSG00000181467	RAP2B	RAP2B, member of RAS oncogene family	-0,5677	0,6747	0,0000	0,0000
ENSG00000131759	RARA	retinoic acid receptor, alpha	0,3867	1,3074	0,0006	0,0060
ENSG00000106538	RARRES2	retinoic acid receptor responder (tazarotene induced) 2	0,6975	1,6217	0,0002	0,0019
ENSG00000133321	RARRES3	retinoic acid receptor responder (tazarotene induced) 3	1,4385	2,7104	0,0000	0,0000
ENSG00000113643	RARS	arginyl-tRNA synthetase	-0,4384	0,7379	0,0000	0,0002
ENSG00000257037	RARSP1	arginyl-tRNA synthetase pseudogene 1	1,1815	2,2681	0,0000	0,0001
ENSG00000185989	RASA3	RAS p21 protein activator 3	-0,9594	0,5143	0,0000	0,0000
ENSG00000111344	RASAL1	RAS protein activator like 1 (GAP1 like)	0,7583	1,6915	0,0000	0,0000
ENSG00000138670	RASGEF1B	RasGEF domain family, member 1B	0,4628	1,3782	0,0005	0,0049
ENSG00000146090	RASGEF1C	RasGEF domain family, member 1C	1,2373	2,3576	0,0000	0,0000
ENSG00000058335	RASGRF1	Ras protein-specific guanine nucleotide-releasing factor 1	0,4694	1,3845	0,0000	0,0000
ENSG00000152689	RASGRP3	RAS guanyl releasing protein 3 (calcium and DAG-regulated)	-1,2626	0,4168	0,0000	0,0000
ENSG00000122035	RASL11A	RAS-like, family 11, member A	-1,0533	0,4819	0,0000	0,0000
ENSG00000169435	RASSF6	Ras association (RalGDS/AF-6) domain family member 6	1,6425	3,1220	0,0000	0,0000

ENSG00000139687	RB1	retinoblastoma 1	-0,5478	0,6840	0,0000	0,0000
ENSG00000125826	RBCK1	RanBP-type and C3HC4-type zinc finger containing 1	0,7604	1,6939	0,0000	0,0000
ENSG00000171174	RBKS	ribokinase	0,7400	1,6701	0,0000	0,0004
ENSG00000244462	RBM12	RNA binding motif protein 12	-0,3851	0,7657	0,0000	0,0000
ENSG00000119707	RBM25	RNA binding motif protein 25	-0,6288	0,6467	0,0000	0,0000
ENSG00000139746	RBM26	RNA binding motif protein 26	-0,9297	0,5250	0,0000	0,0000
ENSG00000227354	RBM26-AS1	RBM26 antisense RNA 1	-0,6120	0,6543	0,0009	0,0082
ENSG00000184898	RBM43	RNA binding motif protein 43	0,6417	1,5601	0,0000	0,0003
ENSG00000147274	RBMX	RNA binding motif protein, X-linked	-0,4007	0,7575	0,0000	0,0002
ENSG00000166831	RBPMS2	RNA binding protein with multiple splicing 2	-0,8808	0,5431	0,0000	0,0000
ENSG00000136161	RCBTB2	regulator of chromosome condensation (RCC1) and BTB (POZ) domain containing p	-0,8739	0,5457	0,0000	0,0000
ENSG00000120158	RCL1	RNA terminal phosphate cyclase-like 1	-0,5463	0,6848	0,0000	0,0000
ENSG00000049449	RCN1	reticulocalbin 1, EF-hand calcium binding domain	-0,3878	0,7643	0,0000	0,0002
ENSG00000142552	RCN3	reticulocalbin 3, EF-hand calcium binding domain	0,7807	1,7179	0,0000	0,0001
ENSG00000167771	RCOR2	REST corepressor 2	0,7706	1,7059	0,0001	0,0011
ENSG00000198570	RD3	retinal degeneration 3	1,4912	2,8113	0,0000	0,0000
ENSG00000240857	RDH14	retinol dehydrogenase 14 (all-trans/9-cis/11-cis)	-0,4702	0,7219	0,0000	0,0006
ENSG00000278023	RDM1	RAD52 motif containing 1	0,6223	1,5393	0,0000	0,0001
ENSG00000132563	REEP2	receptor accessory protein 2	0,7194	1,6465	0,0000	0,0000
ENSG00000142599	RERE	arginine-glutamic acid dipeptide (RE) repeats	0,6450	1,5637	0,0000	0,0000
ENSG00000134533	RERG	RAS-like, estrogen-regulated, growth inhibitor	0,8718	1,8300	0,0008	0,0078
ENSG00000133119	RFC3	replication factor C (activator 1) 3, 38kDa	-0,6805	0,6240	0,0000	0,0000
ENSG00000132005	RFX1	regulatory factor X, 1 (influences HLA class II expression)	0,6926	1,6162	0,0000	0,0000
ENSG00000087903	RFX2	regulatory factor X, 2 (influences HLA class II expression)	0,5828	1,4977	0,0000	0,0000
ENSG00000196460	RFX8	RFX family member 8, lacking RFX DNA binding domain	0,5060	1,4201	0,0001	0,0012
ENSG00000143344	RGL1	ral guanine nucleotide dissociation stimulator-like 1	0,5550	1,4692	0,0000	0,0000
ENSG00000205517	RGL3	ral guanine nucleotide dissociation stimulator-like 3	0,5243	1,4382	0,0002	0,0027
ENSG00000182175	RGMA	repulsive guidance molecule family member a	0,8241	1,7705	0,0003	0,0033
ENSG00000076344	RGS11	regulator of G-protein signaling 11	1,8350	3,5677	0,0000	0,0000
ENSG00000159788	RGS12	regulator of G-protein signaling 12	0,5463	1,4603	0,0000	0,0000
ENSG00000138835	RGS3	regulator of G-protein signaling 3	0,5577	1,4720	0,0000	0,0000

ENSG00000005486	RHBDD2	rhomboid domain containing 2	0,4418	1,3583	0,0000	0,0002
ENSG00000129667	RHBDF2	rhomboid 5 homolog 2 (Drosophila)	0,4954	1,4097	0,0003	0,0032
ENSG00000132677	RHBG	Rh family, B glycoprotein (gene/pseudogene)	1,7409	3,3425	0,0000	0,0000
ENSG00000143878	RHOB	ras homolog family member B	-0,6917	0,6191	0,0000	0,0000
ENSG00000072422	RHOBTB1	Rho-related BTB domain containing 1	1,4099	2,6572	0,0000	0,0000
ENSG00000119729	RHOQ	ras homolog family member Q	0,3914	1,3117	0,0000	0,0001
ENSG00000116574	RHOU	ras homolog family member U	-1,0161	0,4944	0,0000	0,0000
ENSG00000128408	RIBC2	RIB43A domain with coiled-coils 2	0,6668	1,5876	0,0000	0,0000
ENSG00000060709	RIMBP2	RIMS binding protein 2	0,5482	1,4623	0,0000	0,0000
ENSG00000117016	RIMS3	regulating synaptic membrane exocytosis 3	1,4987	2,8259	0,0000	0,0000
ENSG00000204227	RING1	ring finger protein 1	0,4371	1,3539	0,0000	0,0000
ENSG00000135249	RINT1	RAD50 interactor 1	-0,6311	0,6457	0,0000	0,0000
ENSG00000124784	RIOK1	RIO kinase 1	-0,4602	0,7269	0,0000	0,0006
ENSG00000058729	RIOK2	RIO kinase 2	-0,4121	0,7515	0,0001	0,0010
ENSG00000240250	RN7SL541P	RNA, 7SL, cytoplasmic 541, pseudogene	0,8617	1,8171	0,0005	0,0054
ENSG00000136104	RNASEH2B	ribonuclease H2, subunit B	-0,6409	0,6413	0,0000	0,0000
ENSG00000133135	RNF128	ring finger protein 128, E3 ubiquitin protein ligase	0,8454	1,7968	0,0000	0,0000
ENSG00000110315	RNF141	ring finger protein 141	-0,4701	0,7219	0,0000	0,0000
ENSG00000151692	RNF144A	ring finger protein 144A	-1,8745	0,2727	0,0000	0,0000
ENSG00000168159	RNF187	ring finger protein 187	0,4848	1,3994	0,0000	0,0000
ENSG00000121481	RNF2	ring finger protein 2	-0,5078	0,7033	0,0000	0,0001
ENSG00000212864	RNF208	ring finger protein 208	0,9610	1,9467	0,0000	0,0000
ENSG00000178222	RNF212	ring finger protein 212	0,5677	1,4821	0,0003	0,0037
ENSG00000099999	RNF215	ring finger protein 215	0,6936	1,6173	0,0000	0,0000
ENSG00000152193	RNF219	ring finger protein 219	-0,9844	0,5054	0,0000	0,0000
ENSG00000237330	RNF223	ring finger protein 223	1,3571	2,5618	0,0000	0,0000
ENSG00000127870	RNF6	ring finger protein (C3H2C3 type) 6	-0,5500	0,6830	0,0000	0,0000
ENSG00000169855	ROBO1	roundabout guidance receptor 1	2,4076	5,3059	0,0000	0,0000
ENSG00000185008	ROBO2	roundabout guidance receptor 2	1,5053	2,8388	0,0000	0,0000
ENSG00000134318	ROCK2	Rho-associated, coiled-coil containing protein kinase 2	-0,5566	0,6799	0,0000	0,0000
ENSG00000067836	ROGDI	rogdi homolog	0,6433	1,5619	0,0000	0,0000

ENSG00000125995	ROMO1	reactive oxygen species modulator 1	0,4978	1,4121	0,0000	0,0007
ENSG00000185483	ROR1	receptor tyrosine kinase-like orphan receptor 1	-0,9264	0,5262	0,0000	0,0000
ENSG00000069667	RORA	RAR-related orphan receptor A	0,4228	1,3405	0,0000	0,0001
ENSG00000143365	RORC	RAR-related orphan receptor C	0,6065	1,5226	0,0001	0,0013
ENSG00000088899	RP5-1187M17.10	Leucine zipper putative tumor suppressor 3	0,9999	1,9999	0,0000	0,0000
ENSG00000197498	RPF2	ribosome production factor 2 homolog	-0,4588	0,7276	0,0000	0,0002
ENSG00000153574	RPIA	ribose 5-phosphate isomerase A	-0,4992	0,7075	0,0000	0,0000
ENSG00000213228	RPL12P38	ribosomal protein L12 pseudogene 38	-0,6264	0,6478	0,0000	0,0000
ENSG00000236509	RPL21P133	ribosomal protein L21 pseudogene 133	1,8989	3,7292	0,0000	0,0000
ENSG00000163584	RPL22L1	ribosomal protein L22-like 1	-0,7076	0,6123	0,0000	0,0000
ENSG00000148688	RPP30	ribonuclease P/MRP 30kDa subunit	-0,4584	0,7278	0,0000	0,0007
ENSG00000115268	RPS15	ribosomal protein S15	0,5036	1,4177	0,0000	0,0000
ENSG00000233927	RPS28	ribosomal protein S28	0,4138	1,3322	0,0004	0,0042
ENSG00000237432	RPS7P12	ribosomal protein S7 pseudogene 12	1,8118	3,5107	0,0000	0,0000
ENSG00000165526	RPUSD4	RNA pseudouridylate synthase domain containing 4	-0,4409	0,7367	0,0000	0,0001
ENSG00000048392	RRM2B	ribonucleotide reductase M2 B (TP53 inducible)	-0,5776	0,6701	0,0000	0,0000
ENSG00000143303	RRNAD1	ribosomal RNA adenine dimethylase domain containing 1	0,4157	1,3340	0,0000	0,0004
ENSG00000067533	RRP15	ribosomal RNA processing 15 homolog	-0,6874	0,6210	0,0000	0,0000
ENSG00000160208	RRP1B	ribosomal RNA processing 1B	-0,4715	0,7212	0,0000	0,0000
ENSG00000124541	RRP36	ribosomal RNA processing 36	-0,5120	0,7012	0,0000	0,0000
ENSG00000179041	RRS1	ribosome biogenesis regulator homolog	-0,8565	0,5523	0,0000	0,0000
ENSG00000171490	RSL1D1	ribosomal L1 domain containing 1	-0,3827	0,7670	0,0001	0,0019
ENSG00000148484	RSU1	Ras suppressor protein 1	-0,7312	0,6024	0,0000	0,0000
ENSG00000115310	RTN4	reticulon 4	-0,4751	0,7194	0,0000	0,0000
ENSG00000163825	RTP3	receptor (chemosensory) transporter protein 3	1,3880	2,6171	0,0000	0,0000
ENSG00000020633	RUNX3	runt-related transcription factor 3	-0,4065	0,7544	0,0001	0,0011
ENSG00000198853	RUSC2	RUN and SH3 domain containing 2	0,4261	1,3436	0,0000	0,0006
ENSG00000111832	RWDD1	RWD domain containing 1	-0,3999	0,7579	0,0001	0,0010
ENSG00000013392	RWDD2A	RWD domain containing 2A	0,6111	1,5274	0,0000	0,0000
ENSG00000196218	RYR1	ryanodine receptor 1 (skeletal)	-0,4764	0,7188	0,0001	0,0012
ENSG00000198626	RYR2	ryanodine receptor 2 (cardiac)	0,5438	1,4578	0,0000	0,0000

ENSG00000189334	S100A14	S100 calcium binding protein A14	1,3400	2,5315	0,0000	0,0000
ENSG00000188015	S100A3	S100 calcium binding protein A3	0,5804	1,4953	0,0000	0,0000
ENSG00000125910	S1PR4	sphingosine-1-phosphate receptor 4	1,0487	2,0687	0,0001	0,0010
ENSG00000180739	S1PR5	sphingosine-1-phosphate receptor 5	-0,6374	0,6429	0,0000	0,0001
ENSG00000173432	SAA1	serum amyloid A1	-1,1029	0,4656	0,0000	0,0000
ENSG00000134339	SAA2	serum amyloid A2	-1,2079	0,4329	0,0000	0,0000
ENSG00000148965	SAA4	serum amyloid A4, constitutive	-0,9179	0,5293	0,0007	0,0072
ENSG00000211456	SACM1L	SAC1 suppressor of actin mutations 1-like (yeast)	-0,3992	0,7583	0,0000	0,0001
ENSG00000151835	SACS	sacsin molecular chaperone	-0,6013	0,6592	0,0000	0,0000
ENSG00000020577	SAMD4A	sterile alpha motif domain containing 4A	-0,4390	0,7376	0,0000	0,0000
ENSG00000101347	SAMHD1	SAM domain and HD domain 1	-0,6496	0,6375	0,0000	0,0000
ENSG00000150459	SAP18	Sin3A-associated protein, 18kDa	-0,3786	0,7692	0,0001	0,0019
ENSG00000164576	SAP30L	SAP30-like	-0,5463	0,6848	0,0000	0,0000
ENSG00000123453	SARDH	sarcosine dehydrogenase	1,6640	3,1689	0,0000	0,0000
ENSG00000130066	SAT1	spermidine/spermine N1-acetyltransferase 1	0,3789	1,3004	0,0000	0,0001
ENSG00000182568	SATB1	SATB homeobox 1	-1,1964	0,4364	0,0000	0,0000
ENSG00000225648	SBDSP1	Shwachman-Bodian-Diamond syndrome pseudogene 1	-0,5214	0,6967	0,0000	0,0000
ENSG00000188322	SBK1	SH3 domain binding kinase 1	0,5902	1,5054	0,0000	0,0000
ENSG00000109929	SC5D	sterol-C5-desaturase	-0,5204	0,6972	0,0000	0,0000
ENSG00000214279	SCART1	scavenger receptor protein family member	0,8959	1,8608	0,0000	0,0003
ENSG00000145284	SCD5	stearoyl-CoA desaturase 5	-0,7757	0,5841	0,0000	0,0000
ENSG00000184178	SCFD2	sec1 family domain containing 2	-0,5366	0,6894	0,0000	0,0001
ENSG00000124939	SCGB2A1	secretoglobin, family 2A, member 1	2,1269	4,3678	0,0000	0,0000
ENSG00000047634	SCML1	sex comb on midleg-like 1 (Drosophila)	-0,3942	0,7609	0,0000	0,0004
ENSG00000007314	SCN4A	sodium channel, voltage gated, type IV alpha subunit	1,2948	2,4535	0,0000	0,0000
ENSG00000183873	SCN5A	sodium channel, voltage gated, type V alpha subunit	1,2196	2,3288	0,0000	0,0000
ENSG00000111319	SCNN1A	sodium channel, non voltage gated 1 alpha subunit	-0,6156	0,6526	0,0000	0,0000
ENSG00000162572	SCNN1D	sodium channel, non voltage gated 1 delta subunit	0,7248	1,6527	0,0003	0,0038
ENSG00000141295	SCRN2	secernin 2	0,4142	1,3325	0,0006	0,0061
ENSG00000198301	SDAD1	SDA1 domain containing 1	-0,4589	0,7275	0,0000	0,0001
ENSG00000124145	SDC4	syndecan 4	-0,8398	0,5587	0,0000	0,0000

ENSG00000137575	SDCBP	syndecan binding protein (syntenin)	-0,4766	0,7187	0,0000	0,0000
ENSG00000154079	SDHAF4	succinate dehydrogenase complex assembly factor 4	0,4828	1,3975	0,0001	0,0015
ENSG00000069188	SDK2	sidekick cell adhesion molecule 2	0,9580	1,9427	0,0002	0,0022
ENSG00000214491	SEC14L6	SEC14-like lipid binding 6	0,4038	1,3230	0,0006	0,0058
ENSG00000008952	SEC62	SEC62 homolog, preprotein translocation factor	-0,3856	0,7655	0,0000	0,0001
ENSG00000141574	SECTM1	secreted and transmembrane 1	0,5773	1,4921	0,0000	0,0007
ENSG00000085415	SEH1L	SEH1-like nucleoporin	-0,6409	0,6413	0,0000	0,0000
ENSG00000143416	SELENBP1	selenium binding protein 1	0,7012	1,6259	0,0000	0,0000
ENSG00000110876	SELPLG	selectin P ligand	0,5110	1,4250	0,0001	0,0009
ENSG00000075213	SEMA3A	sema domain, immunoglobulin domain (Ig), short basic domain, secreted, (semaph	1,2613	2,3972	0,0000	0,0000
ENSG00000012171	SEMA3B	sema domain, immunoglobulin domain (Ig), short basic domain, secreted, (semaph	0,5895	1,5048	0,0000	0,0001
ENSG00000232352	SEMA3B-AS1	SEMA3B antisense RNA 1 (head to head)	0,8775	1,8372	0,0009	0,0082
ENSG00000001617	SEMA3F	sema domain, immunoglobulin domain (Ig), short basic domain, secreted, (semaph	0,8080	1,7507	0,0000	0,0000
ENSG00000185033	SEMA4B	sema domain, immunoglobulin domain (Ig), transmembrane domain (TM) and shor	0,5035	1,4176	0,0000	0,0003
ENSG00000095539	SEMA4G	sema domain, immunoglobulin domain (Ig), transmembrane domain (TM) and shor	1,2970	2,4571	0,0000	0,0000
ENSG00000112902	SEMA5A	sema domain, seven thrombospondin repeats (type 1 and type 1-like), transmembr	-0,8828	0,5423	0,0005	0,0054
ENSG00000092421	SEMA6A	sema domain, transmembrane domain (TM), and cytoplasmic domain, (semaphori	0,9629	1,9492	0,0004	0,0039
ENSG00000143434	SEMA6C	sema domain, transmembrane domain (TM), and cytoplasmic domain, (semaphori	0,9371	1,9147	0,0000	0,0005
ENSG00000166192	SENP8	SUMO/sentrin peptidase family member, NEDD8 specific	0,5719	1,4865	0,0010	0,0093
ENSG00000162430	SEPN1	selenoprotein N, 1	0,6222	1,5392	0,0000	0,0000
ENSG00000180096	SEPT1	septin 1	0,7636	1,6977	0,0008	0,0077
ENSG00000186522	SEPT10	septin 10	-0,4755	0,7192	0,0000	0,0000
ENSG00000138758	SEPT11	septin 11	-0,5407	0,6875	0,0000	0,0000
ENSG00000108387	SEPT4	septin 4	0,6718	1,5931	0,0000	0,0000
ENSG00000122545	SEPT7	septin 7	-0,4477	0,7332	0,0000	0,0000
ENSG00000164300	SERINC5	serine incorporator 5	0,4230	1,3407	0,0000	0,0000
ENSG00000151778	SERP2	stress-associated endoplasmic reticulum protein family member 2	-2,2973	0,2034	0,0000	0,0000
ENSG00000206075	SERPINB5	serpin peptidase inhibitor, clade B (ovalbumin), member 5	-1,0648	0,4780	0,0000	0,0000
ENSG00000166401	SERPINB8	serpin peptidase inhibitor, clade B (ovalbumin), member 8	-0,7528	0,5934	0,0000	0,0000
ENSG00000170542	SERPINB9	serpin peptidase inhibitor, clade B (ovalbumin), member 9	-0,7510	0,5942	0,0000	0,0000
ENSG00000106366	SERPINE1	serpin peptidase inhibitor, clade E (nexin, plasminogen activator inhibitor type 1), r	0,6750	1,5966	0,0000	0,0000

ENSG00000135919	SERPINE2	serpin peptidase inhibitor, clade E (nexin, plasminogen activator inhibitor type 1), n	-0,3922	0,7620	0,0008	0,0074
ENSG00000149257	SERPINH1	serpin peptidase inhibitor, clade H (heat shock protein 47), member 1, (collagen bi	-0,5319	0,6916	0,0000	0,0000
ENSG00000163536	SERPINI1	serpin peptidase inhibitor, clade I (neuroserpin), member 1	0,8199	1,7653	0,0000	0,0000
ENSG00000130766	SESN2	sestrin 2	0,7974	1,7379	0,0000	0,0000
ENSG00000149212	SESN3	sestrin 3	0,6504	1,5696	0,0000	0,0000
ENSG00000187231	SESTD1	SEC14 and spectrin domains 1	0,5205	1,4344	0,0001	0,0015
ENSG00000119335	SET	SET nuclear proto-oncogene	-0,4840	0,7150	0,0000	0,0000
ENSG00000183576	SETD3	SET domain containing 3	-0,5508	0,6826	0,0000	0,0000
ENSG00000136169	SETDB2	SET domain, bifurcated 2	-0,4953	0,7094	0,0000	0,0000
ENSG00000170364	SETMAR	SET domain and mariner transposase fusion gene	-0,5646	0,6761	0,0001	0,0011
ENSG00000174938	SEZ6L2	seizure related 6 homolog (mouse)-like 2	0,4269	1,3443	0,0005	0,0050
ENSG00000183431	SF3A3	splicing factor 3a, subunit 3, 60kDa	-0,4821	0,7159	0,0000	0,0001
ENSG00000198089	SFI1	SFI1 centrin binding protein	0,5395	1,4535	0,0000	0,0000
ENSG00000116560	SFPQ	splicing factor proline/glutamine-rich	-0,5679	0,6746	0,0000	0,0000
ENSG00000107819	SFXN3	sideroflexin 3	0,6170	1,5337	0,0000	0,0000
ENSG00000226200	SGMS1-AS1	SGMS1 antisense RNA 1	0,4800	1,3948	0,0007	0,0068
ENSG00000164023	SGMS2	sphingomyelin synthase 2	-0,4655	0,7242	0,0000	0,0000
ENSG00000163535	SGOL2	shugoshin-like 2 (S. pombe)	-0,4662	0,7239	0,0000	0,0000
ENSG00000163082	SGPP2	sphingosine-1-phosphate phosphatase 2	-2,3119	0,2014	0,0000	0,0000
ENSG00000167037	SGSM1	small G protein signaling modulator 1	0,9522	1,9349	0,0002	0,0026
ENSG00000141258	SGSM2	small G protein signaling modulator 2	0,4339	1,3509	0,0002	0,0026
ENSG00000100359	SGSM3	small G protein signaling modulator 3	0,4666	1,3819	0,0001	0,0015
ENSG00000027869	SH2D2A	SH2 domain containing 2A	0,5645	1,4788	0,0005	0,0051
ENSG00000189410	SH2D5	SH2 domain containing 5	-0,5635	0,6766	0,0000	0,0000
ENSG00000214193	SH3D21	SH3 domain containing 21	1,0205	2,0286	0,0000	0,0000
ENSG00000141985	SH3GL1	SH3-domain GRB2-like 1	0,4473	1,3635	0,0005	0,0056
ENSG00000280693	SH3PXD2A-AS1	SH3PXD2A antisense RNA 1	-0,5761	0,6708	0,0000	0,0000
ENSG00000154447	SH3RF1	SH3 domain containing ring finger 1	-0,8140	0,5688	0,0000	0,0000
ENSG00000035115	SH3YL1	SH3 and SYLF domain containing 1	-0,5420	0,6868	0,0000	0,0000
ENSG00000164690	SHH	sonic hedgehog	-1,7918	0,2888	0,0000	0,0000
ENSG00000180730	SHISA2	shisa family member 2	-0,8991	0,5362	0,0000	0,0002

ENSG00000198892	SHISA4	shisa family member 4	0,8947	1,8592	0,0000	0,0000
ENSG00000182199	SHMT2	serine hydroxymethyltransferase 2 (mitochondrial)	0,4865	1,4010	0,0000	0,0000
ENSG00000144736	SHQ1	SHQ1, H/ACA ribonucleoprotein assembly factor	-0,4279	0,7434	0,0001	0,0019
ENSG00000164403	SHROOM1	shroom family member 1	0,4315	1,3486	0,0000	0,0004
ENSG00000149577	SIDT2	SID1 transmembrane family, member 2	0,5331	1,4470	0,0000	0,0000
ENSG00000120725	SIL1	SIL1 nucleotide exchange factor	0,7176	1,6445	0,0000	0,0000
ENSG00000213445	SIPA1	signal-induced proliferation-associated 1	0,5252	1,4392	0,0000	0,0001
ENSG00000124523	SIRT5	sirtuin 5	0,4579	1,3735	0,0000	0,0001
ENSG00000177045	SIX5	SIX homeobox 5	0,7600	1,6935	0,0000	0,0000
ENSG00000165480	SKA3	spindle and kinetochore associated complex subunit 3	-0,8872	0,5407	0,0000	0,0000
ENSG00000180592	SKIDA1	SKI/DACH domain containing 1	-0,8583	0,5516	0,0000	0,0003
ENSG00000155926	SLA	Src-like-adaptor	0,8720	1,8302	0,0000	0,0000
ENSG00000139737	SLAIN1	SLAIN motif family, member 1	-1,3797	0,3843	0,0000	0,0000
ENSG00000064651	SLC12A2	solute carrier family 12 (sodium/potassium/chloride transporter), member 2	-0,4062	0,7546	0,0000	0,0000
ENSG00000140199	SLC12A6	solute carrier family 12 (potassium/chloride transporter), member 6	0,4062	1,3252	0,0001	0,0016
ENSG00000113504	SLC12A7	solute carrier family 12 (potassium/chloride transporter), member 7	0,5606	1,4749	0,0000	0,0001
ENSG00000146828	SLC12A9	solute carrier family 12, member 9	-0,4061	0,7546	0,0009	0,0087
ENSG00000088386	SLC15A1	solute carrier family 15 (oligopeptide transporter), member 1	-1,4470	0,3668	0,0000	0,0000
ENSG00000155380	SLC16A1	solute carrier family 16 (monocarboxylate transporter), member 1	-0,6478	0,6383	0,0000	0,0000
ENSG00000174327	SLC16A13	solute carrier family 16, member 13	0,4911	1,4055	0,0002	0,0028
ENSG00000147100	SLC16A2	solute carrier family 16, member 2 (thyroid hormone transporter)	-1,2278	0,4270	0,0000	0,0000
ENSG00000168679	SLC16A4	solute carrier family 16, member 4	1,1850	2,2736	0,0000	0,0000
ENSG00000165449	SLC16A9	solute carrier family 16, member 9	-1,0038	0,4987	0,0000	0,0000
ENSG00000104888	SLC17A7	solute carrier family 17 (vesicular glutamate transporter), member 7	1,0197	2,0274	0,0000	0,0000
ENSG00000106688	SLC1A1	solute carrier family 1 (neuronal/epithelial high affinity glutamate transporter, syst	-0,5432	0,6862	0,0003	0,0038
ENSG00000110436	SLC1A2	solute carrier family 1 (glial high affinity glutamate transporter), member 2	0,7361	1,6657	0,0000	0,0000
ENSG00000144136	SLC20A1	solute carrier family 20 (phosphate transporter), member 1	-0,5321	0,6915	0,0000	0,0000
ENSG00000110628	SLC22A18	solute carrier family 22, member 18	0,8527	1,8059	0,0000	0,0000
ENSG00000197847	SLC22A20	solute carrier family 22, member 20	0,6809	1,6031	0,0002	0,0027
ENSG00000137266	SLC22A23	solute carrier family 22, member 23	0,6064	1,5224	0,0000	0,0000
ENSG00000183048	SLC25A10	solute carrier family 25 (mitochondrial carrier; dicarboxylate transporter), member	0,4479	1,3640	0,0002	0,0026

ENSG00000004864	SLC25A13	solute carrier family 25 (aspartate/glutamate carrier), member 13	-0,4264	0,7441	0,0000	0,0001
ENSG00000102743	SLC25A15	solute carrier family 25 (mitochondrial carrier; ornithine transporter) member 15	-0,5112	0,7017	0,0000	0,0000
ENSG00000178537	SLC25A20	solute carrier family 25 (carnitine/acylcarnitine translocase), member 20	-0,6115	0,6545	0,0000	0,0002
ENSG00000085491	SLC25A24	solute carrier family 25 (mitochondrial carrier; phosphate carrier), member 24	-0,5126	0,7009	0,0000	0,0000
ENSG00000234771	SLC25A25-AS1	SLC25A25 antisense RNA 1	-0,7033	0,6142	0,0000	0,0000
ENSG00000153291	SLC25A27	solute carrier family 25, member 27	0,6789	1,6009	0,0000	0,0000
ENSG00000075415	SLC25A3	solute carrier family 25 (mitochondrial carrier; phosphate carrier), member 3	-0,3907	0,7628	0,0001	0,0016
ENSG00000164933	SLC25A32	solute carrier family 25 (mitochondrial folate carrier), member 32	-0,6018	0,6589	0,0000	0,0000
ENSG00000171612	SLC25A33	solute carrier family 25 (pyrimidine nucleotide carrier), member 33	-0,4319	0,7413	0,0002	0,0028
ENSG00000125434	SLC25A35	solute carrier family 25, member 35	0,4955	1,4098	0,0001	0,0011
ENSG00000147454	SLC25A37	solute carrier family 25 (mitochondrial iron transporter), member 37	-0,5537	0,6813	0,0000	0,0000
ENSG00000181035	SLC25A42	solute carrier family 25, member 42	0,8657	1,8222	0,0000	0,0000
ENSG00000174502	SLC26A9	solute carrier family 26 (anion exchanger), member 9	0,6652	1,5858	0,0002	0,0021
ENSG00000130304	SLC27A1	solute carrier family 27 (fatty acid transporter), member 1	0,5450	1,4591	0,0000	0,0001
ENSG00000140284	SLC27A2	solute carrier family 27 (fatty acid transporter), member 2	-0,3881	0,7641	0,0004	0,0048
ENSG00000164638	SLC29A4	solute carrier family 29 (equilibrative nucleoside transporter), member 4	0,8735	1,8321	0,0000	0,0000
ENSG00000197496	SLC2A10	solute carrier family 2 (facilitated glucose transporter), member 10	0,5222	1,4361	0,0000	0,0000
ENSG00000133460	SLC2A11	solute carrier family 2 (facilitated glucose transporter), member 11	0,4414	1,3579	0,0002	0,0022
ENSG00000146411	SLC2A12	solute carrier family 2 (facilitated glucose transporter), member 12	0,7185	1,6455	0,0000	0,0000
ENSG00000173262	SLC2A14	solute carrier family 2 (facilitated glucose transporter), member 14	0,5404	1,4544	0,0002	0,0023
ENSG00000131183	SLC34A1	solute carrier family 34 (type II sodium/phosphate cotransporter), member 1	-1,0747	0,4748	0,0000	0,0000
ENSG00000181830	SLC35C1	solute carrier family 35 (GDP-fucose transporter), member C1	-0,4680	0,7230	0,0000	0,0001
ENSG00000080189	SLC35C2	solute carrier family 35 (GDP-fucose transporter), member C2	0,6055	1,5215	0,0000	0,0000
ENSG00000110660	SLC35F2	solute carrier family 35, member F2	-0,6980	0,6164	0,0000	0,0000
ENSG00000111371	SLC38A1	solute carrier family 38, member 1	-0,4260	0,7443	0,0000	0,0000
ENSG00000017483	SLC38A5	solute carrier family 38, member 5	-0,4429	0,7357	0,0000	0,0000
ENSG00000139974	SLC38A6	solute carrier family 38, member 6	-0,5602	0,6782	0,0000	0,0000
ENSG00000196950	SLC39A10	solute carrier family 39 (zinc transporter), member 10	-0,4146	0,7502	0,0000	0,0000
ENSG00000138821	SLC39A8	solute carrier family 39 (zinc transporter), member 8	-0,4899	0,7121	0,0000	0,0000
ENSG00000029364	SLC39A9	solute carrier family 39, member 9	-0,3968	0,7595	0,0000	0,0000
ENSG00000149150	SLC43A1	solute carrier family 43 (amino acid system L transporter), member 1	0,3892	1,3097	0,0000	0,0004

ENSG00000162426	SLC45A1	solute carrier family 45, member 1	1,1983	2,2947	0,0000	0,0000
ENSG00000076351	SLC46A1	solute carrier family 46 (folate transporter), member 1	-0,3788	0,7691	0,0000	0,0003
ENSG00000142494	SLC47A1	solute carrier family 47 (multidrug and toxin extrusion), member 1	-0,7900	0,5783	0,0001	0,0012
ENSG00000100170	SLC5A1	solute carrier family 5 (sodium/glucose cotransporter), member 1	0,7920	1,7315	0,0000	0,0000
ENSG00000196517	SLC6A9	solute carrier family 6 (neurotransmitter transporter, glycine), member 9	1,5431	2,9141	0,0000	0,0000
ENSG00000151012	SLC7A11	solute carrier family 7 (anionic amino acid transporter light chain, xc- system), mem	0,8578	1,8123	0,0000	0,0000
ENSG00000099960	SLC7A4	solute carrier family 7, member 4	0,8644	1,8206	0,0001	0,0013
ENSG00000103064	SLC7A6	solute carrier family 7 (amino acid transporter light chain, y+L system), member 6	-0,6956	0,6174	0,0000	0,0000
ENSG00000155465	SLC7A7	solute carrier family 7 (amino acid transporter light chain, y+L system), member 7	-0,4812	0,7164	0,0000	0,0000
ENSG00000205045	SLFN12L	schlafen family member 12-like	-0,6896	0,6200	0,0002	0,0027
ENSG00000188827	SLX4	SLX4 structure-specific endonuclease subunit	0,5366	1,4506	0,0000	0,0000
ENSG00000170545	SMAGP	small cell adhesion glycoprotein	-0,6493	0,6376	0,0000	0,0000
ENSG00000102038	SMARCA1	SWI/SNF related, matrix associated, actin dependent regulator of chromatin, subfa	-0,6067	0,6567	0,0000	0,0000
ENSG00000113810	SMC4	structural maintenance of chromosomes 4	-0,3791	0,7689	0,0000	0,0001
ENSG00000165935	SMCO2	single-pass membrane protein with coiled-coil domains 2	1,1394	2,2029	0,0000	0,0000
ENSG00000235169	SMIM1	small integral membrane protein 1 (Vel blood group)	1,6231	3,0804	0,0000	0,0000
ENSG00000163683	SMIM14	small integral membrane protein 14	0,9145	1,8850	0,0000	0,0000
ENSG00000188725	SMIM15	small integral membrane protein 15	-0,4516	0,7313	0,0000	0,0001
ENSG00000267795	SMIM22	small integral membrane protein 22	0,4366	1,3534	0,0007	0,0068
ENSG00000128602	SMO	smoothened, frizzled class receptor	-0,5551	0,6806	0,0004	0,0047
ENSG00000103056	SMPD3	sphingomyelin phosphodiesterase 3, neutral membrane (neutral sphingomyelinase	1,5645	2,9578	0,0000	0,0000
ENSG00000130768	SMPDL3B	sphingomyelin phosphodiesterase, acid-like 3B	-0,5308	0,6922	0,0001	0,0011
ENSG00000108854	SMURF2	SMAD specific E3 ubiquitin protein ligase 2	-0,4238	0,7454	0,0000	0,0000
ENSG00000185420	SMYD3	SET and MYND domain containing 3	0,5345	1,4485	0,0000	0,0000
ENSG00000124216	SNAI1	snail family zinc finger 1	0,4404	1,3569	0,0007	0,0067
ENSG00000185669	SNAI3	snail family zinc finger 3	1,0756	2,1077	0,0000	0,0002
ENSG00000174446	SNAPC5	small nuclear RNA activating complex, polypeptide 5, 19kDa	0,5543	1,4685	0,0000	0,0000
ENSG00000173267	SNCG	synuclein, gamma (breast cancer-specific protein 1)	1,1340	2,1947	0,0000	0,0000
ENSG00000247092	SNHG10	small nucleolar RNA host gene 10	-0,8594	0,5512	0,0000	0,0000
ENSG00000163597	SNHG16	small nucleolar RNA host gene 16	-0,5243	0,6953	0,0000	0,0000
ENSG00000260260	SNHG19	small nucleolar RNA host gene 19	-0,6730	0,6272	0,0000	0,0000

ENSG00000234912	SNHG20	small nucleolar RNA host gene 20	-0,4962	0,7090	0,0000	0,0006
ENSG00000242125	SNHG3	small nucleolar RNA host gene 3	-0,5998	0,6598	0,0000	0,0000
ENSG00000281398	SNHG4	small nucleolar RNA host gene 4	-0,8366	0,5600	0,0000	0,0000
ENSG00000245910	SNHG6	small nucleolar RNA host gene 6	0,6370	1,5551	0,0000	0,0000
ENSG00000131876	SNRPA1	small nuclear ribonucleoprotein polypeptide A'	-0,4641	0,7249	0,0000	0,0000
ENSG00000167088	SNRPD1	small nuclear ribonucleoprotein D1 polypeptide 16kDa	-0,4061	0,7547	0,0001	0,0009
ENSG00000101400	SNTA1	syntrophin, alpha 1	0,5347	1,4486	0,0000	0,0002
ENSG00000172164	SNTB1	syntrophin, beta 1 (dystrophin-associated protein A1, 59kDa, basic component 1)	0,3801	1,3015	0,0000	0,0001
ENSG00000168807	SNTB2	syntrophin, beta 2 (dystrophin-associated protein A1, 59kDa, basic component 2)	-0,3986	0,7586	0,0000	0,0000
ENSG00000173548	SNX33	sorting nexin 33	0,4403	1,3569	0,0000	0,0001
ENSG00000162627	SNX7	sorting nexin 7	-0,5617	0,6775	0,0000	0,0001
ENSG00000057252	SOAT1	sterol O-acyltransferase 1	-0,4343	0,7401	0,0000	0,0001
ENSG00000120833	SOCS2	suppressor of cytokine signaling 2	-0,6502	0,6372	0,0000	0,0000
ENSG00000171150	SOCS5	suppressor of cytokine signaling 5	-0,3940	0,7610	0,0000	0,0004
ENSG00000154556	SORBS2	sorbin and SH3 domain containing 2	0,5584	1,4726	0,0000	0,0000
ENSG00000184985	SORCS2	sortilin-related VPS10 domain containing receptor 2	0,5992	1,5149	0,0000	0,0005
ENSG00000137642	SORL1	sortilin-related receptor, L(DLR class) A repeats containing	0,5659	1,4803	0,0000	0,0000
ENSG00000198142	SOWAHC	sosondowah ankyrin repeat domain family member C	-0,6061	0,6570	0,0000	0,0000
ENSG00000187808	SOWAHD	sosondowah ankyrin repeat domain family member D	0,9898	1,9859	0,0001	0,0009
ENSG00000177732	SOX12	SRY (sex determining region Y)-box 12	0,5012	1,4154	0,0000	0,0000
ENSG00000168875	SOX14	SRY (sex determining region Y)-box 14	1,0568	2,0803	0,0000	0,0004
ENSG00000181449	SOX2	SRY (sex determining region Y)-box 2	-0,6242	0,6488	0,0001	0,0014
ENSG00000061656	SPAG4	sperm associated antigen 4	0,9060	1,8739	0,0000	0,0000
ENSG00000227234	SPANXB1	SPANX family, member B1	-0,8948	0,5378	0,0004	0,0044
ENSG00000113140	SPARC	secreted protein, acidic, cysteine-rich (osteonectin)	-1,3882	0,3820	0,0000	0,0000
ENSG00000186451	SPATA12	spermatogenesis associated 12	0,7167	1,6434	0,0000	0,0005
ENSG00000182957	SPATA13	spermatogenesis associated 13	-0,5372	0,6891	0,0000	0,0000
ENSG00000006282	SPATA20	spermatogenesis associated 20	0,4913	1,4057	0,0000	0,0000
ENSG00000171763	SPATA5L1	spermatogenesis associated 5-like 1	-0,4565	0,7287	0,0002	0,0026
ENSG00000196141	SPATS2L	spermatogenesis associated, serine-rich 2-like	-0,4773	0,7183	0,0000	0,0000
ENSG00000161888	SPC24	SPC24, NDC80 kinetochore complex component	0,4852	1,3998	0,0000	0,0000

ENSG00000072195	SPEG	SPEG complex locus	0,5486	1,4627	0,0002	0,0024
ENSG00000183018	SPNS2	spinster homolog 2 (Drosophila)	1,7086	3,2684	0,0000	0,0000
ENSG00000262655	SPON1	spondin 1, extracellular matrix protein	-2,4823	0,1790	0,0000	0,0000
ENSG00000138600	SPPL2A	signal peptide peptidase like 2A	-0,4360	0,7392	0,0000	0,0000
ENSG00000005206	SPPL2B	signal peptide peptidase like 2B	0,4677	1,3829	0,0008	0,0078
ENSG00000136158	SPRY2	sprouty RTK signaling antagonist 2	-0,9074	0,5331	0,0000	0,0000
ENSG00000123178	SPRYD7	SPRY domain containing 7	-0,5977	0,6608	0,0000	0,0000
ENSG00000111671	SPSB2	splA/ryanodine receptor domain and SOCS box containing 2	0,5571	1,4713	0,0001	0,0015
ENSG00000162032	SPSB3	splA/ryanodine receptor domain and SOCS box containing 3	0,7103	1,6361	0,0000	0,0000
ENSG00000070182	SPTB	spectrin, beta, erythrocytic	0,8824	1,8434	0,0000	0,0000
ENSG00000173898	SPTBN2	spectrin, beta, non-erythrocytic 2	0,5730	1,4876	0,0000	0,0003
ENSG00000100596	SPTLC2	serine palmitoyltransferase, long chain base subunit 2	-0,4155	0,7498	0,0000	0,0000
ENSG00000104549	SQLE	squalene epoxidase	-0,4555	0,7293	0,0000	0,0000
ENSG00000277363	SRCIN1	SRC kinase signaling inhibitor 1	0,6179	1,5346	0,0000	0,0001
ENSG00000072310	SREBF1	sterol regulatory element binding transcription factor 1	0,5995	1,5152	0,0000	0,0000
ENSG00000151304	SRFBP1	serum response factor binding protein 1	-0,4050	0,7552	0,0000	0,0004
ENSG00000248508	SRP14-AS1	SRP14 antisense RNA1 (head to head)	0,6274	1,5448	0,0002	0,0020
ENSG00000096063	SRPK1	SRSF protein kinase 1	-0,4036	0,7560	0,0000	0,0001
ENSG00000101955	SRPX	sushi-repeat containing protein, X-linked	1,4225	2,6804	0,0000	0,0000
ENSG00000087087	SRRT	serrate, RNA effector molecule	-0,3927	0,7617	0,0000	0,0001
ENSG00000136450	SRSF1	serine/arginine-rich splicing factor 1	-0,6769	0,6255	0,0000	0,0000
ENSG00000161547	SRSF2	serine/arginine-rich splicing factor 2	-0,7494	0,5949	0,0000	0,0000
ENSG00000115875	SRSF7	serine/arginine-rich splicing factor 7	-0,4115	0,7518	0,0000	0,0001
ENSG00000141380	SS18	synovial sarcoma translocation, chromosome 18	-0,3786	0,7692	0,0000	0,0000
ENSG00000146700	SSC4D	scavenger receptor cysteine rich family, 4 domains	1,0435	2,0612	0,0000	0,0000
ENSG00000197558	SSPO	SCO-spondin	1,1125	2,1622	0,0000	0,0000
ENSG00000114850	SSR3	signal sequence receptor, gamma (translocon-associated protein gamma)	-0,4537	0,7302	0,0000	0,0000
ENSG00000126091	ST3GAL3	ST3 beta-galactoside alpha-2,3-sialyltransferase 3	0,4477	1,3639	0,0007	0,0067
ENSG00000073849	ST6GAL1	ST6 beta-galactosamide alpha-2,6-sialyltranferase 1	-0,3865	0,7650	0,0000	0,0007
ENSG00000070731	ST6GALNAC2	ST6 (alpha-N-acetyl-neuraminyl-2,3-beta-galactosyl-1,3)-N-acetylgalactosaminide a	1,6301	3,0953	0,0000	0,0000
ENSG00000160408	ST6GALNAC6	ST6 (alpha-N-acetyl-neuraminyl-2,3-beta-galactosyl-1,3)-N-acetylgalactosaminide a	0,7776	1,7143	0,0000	0,0000

ENSG00000118007	STAG1	stromal antigen 1	-0,5442	0,6858	0,0000	0,0000
ENSG00000214530	STARD10	StAR-related lipid transfer (START) domain containing 10	0,3866	1,3073	0,0004	0,0045
ENSG00000084090	STARD7	StAR-related lipid transfer (START) domain containing 7	-0,5940	0,6625	0,0000	0,0000
ENSG00000130052	STARD8	StAR-related lipid transfer (START) domain containing 8	0,9634	1,9500	0,0000	0,0000
ENSG00000159433	STARD9	StAR-related lipid transfer (START) domain containing 9	0,5174	1,4314	0,0000	0,0002
ENSG00000170581	STAT2	signal transducer and activator of transcription 2, 113kDa	0,4014	1,3208	0,0001	0,0008
ENSG00000159167	STC1	stanniocalcin 1	0,7976	1,7382	0,0000	0,0000
ENSG00000113739	STC2	stanniocalcin 2	0,6261	1,5433	0,0000	0,0000
ENSG00000164647	STEAP1	six transmembrane epithelial antigen of the prostate 1	-0,6685	0,6292	0,0000	0,0000
ENSG00000102572	STK24	serine/threonine kinase 24	-0,7866	0,5797	0,0000	0,0000
ENSG00000169302	STK32A	serine/threonine kinase 32A	0,9740	1,9643	0,0000	0,0000
ENSG00000101109	STK4	serine/threonine kinase 4	-0,4134	0,7508	0,0000	0,0000
ENSG00000197457	STMN3	stathmin-like 3	-0,3942	0,7609	0,0002	0,0024
ENSG00000001460	STPG1	sperm-tail PG-rich repeat containing 1	0,4671	1,3824	0,0010	0,0090
ENSG00000169689	STRA13	stimulated by retinoic acid 13	0,5026	1,4167	0,0000	0,0000
ENSG00000082146	STRADB	STE20-related kinase adaptor beta	-0,5231	0,6959	0,0000	0,0003
ENSG00000090372	STRN4	striatin, calmodulin binding protein 4	0,3824	1,3035	0,0006	0,0060
ENSG00000106089	STX1A	syntaxin 1A (brain)	0,5252	1,4392	0,0000	0,0000
ENSG00000162236	STX5	syntaxin 5	0,3818	1,3030	0,0000	0,0002
ENSG00000164506	STXBP5	syntaxin binding protein 5 (tomosyn)	-0,4662	0,7239	0,0000	0,0000
ENSG00000113387	SUB1	SUB1 homolog, transcriptional regulator	-0,4247	0,7450	0,0000	0,0001
ENSG00000136143	SUCLA2	succinate-CoA ligase, ADP-forming, beta subunit	-0,6764	0,6257	0,0000	0,0000
ENSG00000165416	SUGT1	SGT1 homolog, MIS12 kinetochore complex assembly cochaperone	-0,6400	0,6417	0,0000	0,0000
ENSG00000198203	SULT1C2	sulfotransferase family, cytosolic, 1C, member 2	1,1948	2,2891	0,0000	0,0000
ENSG00000139531	SUOX	sulfite oxidase	0,4470	1,3632	0,0000	0,0001
ENSG00000102710	SUPT20H	SPT20 homolog, SAGA complex component	-0,4359	0,7392	0,0000	0,0000
ENSG00000152455	SUV39H2	suppressor of variegation 3-9 homolog 2 (Drosophila)	-0,4863	0,7138	0,0000	0,0000
ENSG00000133247	SUV420H2	suppressor of variegation 4-20 homolog 2 (Drosophila)	0,9417	1,9207	0,0000	0,0000
ENSG00000147642	SYBU	syntabulin (syntaxin-interacting)	1,0094	2,0130	0,0000	0,0000
ENSG00000161860	SYCE2	synaptonemal complex central element protein 2	0,8713	1,8293	0,0000	0,0003
ENSG00000196074	SYCP2	synaptonemal complex protein 2	-0,9056	0,5338	0,0006	0,0057

ENSG00000135316	SYNCRIP	synaptotagmin binding, cytoplasmic RNA interacting protein	-0,5106	0,7019	0,0000	0,0000
ENSG00000131018	SYNE1	spectrin repeat containing, nuclear envelope 1	0,8703	1,8281	0,0000	0,0000
ENSG00000181392	SYNE4	spectrin repeat containing, nuclear envelope family member 4	1,4858	2,8007	0,0000	0,0000
ENSG00000100321	SYNGR1	synaptogyrin 1	0,6259	1,5432	0,0000	0,0001
ENSG00000171992	SYNPO	synaptopodin	0,9236	1,8969	0,0000	0,0001
ENSG00000008282	SYPL1	synaptophysin-like 1	-0,6051	0,6574	0,0000	0,0000
ENSG00000204070	SYS1	Sys1 golgi trafficking protein	-0,4162	0,7494	0,0000	0,0003
ENSG00000019505	SYT13	synaptotagmin XIII	1,4885	2,8059	0,0000	0,0000
ENSG00000011347	SYT7	synaptotagmin VII	0,7554	1,6881	0,0000	0,0000
ENSG00000142765	SYTL1	synaptotagmin-like 1	0,7967	1,7371	0,0003	0,0029
ENSG00000164674	SYTL3	synaptotagmin-like 3	1,0198	2,0277	0,0000	0,0000
ENSG00000164458	T	T brachyury transcription factor	-0,9598	0,5141	0,0000	0,0000
ENSG00000100324	TAB1	TGF-beta activated kinase 1/MAP3K7 binding protein 1	0,5640	1,4783	0,0000	0,0000
ENSG00000197780	TAF13	TAF13 RNA polymerase II, TATA box binding protein (TBP)-associated factor, 18kDa	-0,4880	0,7130	0,0000	0,0005
ENSG00000166012	TAF1D	TATA box binding protein (TBP)-associated factor, RNA polymerase I, D, 41kDa	-0,4729	0,7205	0,0000	0,0001
ENSG00000141384	TAF4B	TAF4b RNA polymerase II, TATA box binding protein (TBP)-associated factor, 105kD	-0,3853	0,7656	0,0005	0,0051
ENSG00000187325	TAF9B	TAF9B RNA polymerase II, TATA box binding protein (TBP)-associated factor, 31kDa	-0,5898	0,6644	0,0000	0,0000
ENSG00000164691	TAGAP	T-cell activation RhoGTPase activating protein	-1,2178	0,4299	0,0000	0,0001
ENSG00000204267	TAP2	transporter 2, ATP-binding cassette, sub-family B (MDR/TAP)	-0,5615	0,6776	0,0000	0,0000
ENSG00000157014	TATDN2	TatD DNase domain containing 2	-0,4726	0,7207	0,0000	0,0000
ENSG00000203705	TATDN3	TatD DNase domain containing 3	0,6303	1,5479	0,0000	0,0000
ENSG00000102125	TAZ	tafazzin	0,4265	1,3440	0,0000	0,0005
ENSG00000175463	TBC1D10C	TBC1 domain family, member 10C	1,5037	2,8357	0,0000	0,0000
ENSG00000104946	TBC1D17	TBC1 domain family, member 17	0,5864	1,5015	0,0000	0,0000
ENSG00000095383	TBC1D2	TBC1 domain family, member 2	-0,3905	0,7629	0,0007	0,0068
ENSG00000068354	TBC1D25	TBC1 domain family, member 25	0,4060	1,3250	0,0002	0,0021
ENSG00000136111	TBC1D4	TBC1 domain family, member 4	-0,7459	0,5963	0,0000	0,0000
ENSG00000177565	TBL1XR1	transducin (beta)-like 1 X-linked receptor 1	-0,4249	0,7449	0,0000	0,0000
ENSG00000112837	TBX18	T-box 18	0,5884	1,5036	0,0000	0,0000
ENSG00000165929	TC2N	tandem C2 domains, nuclear	-0,5292	0,6929	0,0000	0,0000
ENSG00000204219	TCEA3	transcription elongation factor A (SII), 3	0,8319	1,7800	0,0000	0,0000

ENSG00000154582	TCEB1	transcription elongation factor B (SIII), polypeptide 1 (15kDa, elongin C)	-0,4105	0,7524	0,0001	0,0015
ENSG00000103363	TCEB2	transcription elongation factor B (SIII), polypeptide 2 (18kDa, elongin B)	0,4007	1,3202	0,0008	0,0080
ENSG00000152284	TCF7L1	transcription factor 7-like 1 (T-cell specific, HMG-box)	0,6541	1,5736	0,0000	0,0000
ENSG00000159450	TCHH	trichohyalin	-0,6663	0,6301	0,0003	0,0036
ENSG00000166046	TCP11L2	t-complex 11, testis-specific-like 2	1,2275	2,3416	0,0000	0,0000
ENSG00000213123	TCTEX1D2	Tctex1 domain containing 2	-0,7220	0,6063	0,0000	0,0000
ENSG00000168778	TCTN2	tectonic family member 2	0,9045	1,8719	0,0000	0,0000
ENSG00000139372	TDG	thymine DNA glycosylase	-0,4687	0,7226	0,0000	0,0001
ENSG00000083544	TDRD3	tudor domain containing 3	-0,4426	0,7358	0,0000	0,0002
ENSG00000205356	TECPR1	tectonin beta-propeller repeat containing 1	0,5110	1,4250	0,0001	0,0010
ENSG00000167074	TEF	thyrotrophic embryonic factor	0,5679	1,4824	0,0000	0,0000
ENSG00000172171	TEFM	transcription elongation factor, mitochondrial	-0,4742	0,7199	0,0002	0,0019
ENSG00000009694	TENM1	teneurin transmembrane protein 1	-0,6312	0,6457	0,0005	0,0055
ENSG00000145934	TENM2	teneurin transmembrane protein 2	-1,5582	0,3396	0,0000	0,0000
ENSG00000182459	TEX19	testis expressed 19	1,0054	2,0075	0,0000	0,0000
ENSG00000144043	TEX261	testis expressed 261	-0,4379	0,7382	0,0000	0,0000
ENSG00000151287	TEX30	testis expressed 30	-0,8045	0,5726	0,0000	0,0000
ENSG00000226674	TEX41	testis expressed 41 (non-protein coding)	-0,9465	0,5189	0,0003	0,0037
ENSG00000108064	TFAM	transcription factor A, mitochondrial	-0,5242	0,6953	0,0000	0,0000
ENSG00000087510	TFAP2C	transcription factor AP-2 gamma (activating enhancer binding protein 2 gamma)	0,3806	1,3019	0,0001	0,0013
ENSG00000162851	TFB2M	transcription factor B2, mitochondrial	-0,4296	0,7425	0,0001	0,0009
ENSG00000198176	TFDP1	transcription factor Dp-1	-0,9771	0,5080	0,0000	0,0000
ENSG00000068323	TFE3	transcription factor binding to IGHM enhancer 3	-0,4606	0,7267	0,0000	0,0000
ENSG00000072274	TFRC	transferrin receptor	-0,6613	0,6323	0,0000	0,0000
ENSG00000184436	THAP7	THAP domain containing 7	0,4581	1,3737	0,0006	0,0061
ENSG00000161277	THAP8	THAP domain containing 8	0,9090	1,8777	0,0000	0,0000
ENSG00000169231	THBS3	thrombospondin 3	0,9606	1,9461	0,0000	0,0000
ENSG00000130775	THEMIS2	thymocyte selection associated family member 2	0,6899	1,6132	0,0000	0,0000
ENSG00000131652	THOC6	THO complex 6	0,9347	1,9115	0,0000	0,0000
ENSG00000126351	THRA	thyroid hormone receptor, alpha	0,5170	1,4310	0,0000	0,0000
ENSG00000259431	THTPA	thiamine triphosphatase	0,5587	1,4729	0,0001	0,0018

ENSG00000066654	THUMPD1	THUMP domain containing 1	-0,4263	0,7442	0,0000	0,0000
ENSG00000078237	TIGAR	TP53 induced glycolysis regulatory phosphatase	-0,4293	0,7426	0,0000	0,0002
ENSG00000180346	TIGD2	tigger transposable element derived 2	-1,2305	0,4262	0,0000	0,0000
ENSG00000173825	TIGD3	tigger transposable element derived 3	0,7096	1,6353	0,0003	0,0030
ENSG00000134809	TIMM10	translocase of inner mitochondrial membrane 10 homolog (yeast)	-0,4442	0,7350	0,0000	0,0004
ENSG00000132286	TIMM10B	translocase of inner mitochondrial membrane 10 homolog B (yeast)	-0,4257	0,7445	0,0000	0,0001
ENSG00000134375	TIMM17A	translocase of inner mitochondrial membrane 17 homolog A (yeast)	-0,4460	0,7341	0,0001	0,0008
ENSG00000126768	TIMM17B	translocase of inner mitochondrial membrane 17 homolog B (yeast)	0,4925	1,4069	0,0000	0,0000
ENSG00000126953	TIMM8A	translocase of inner mitochondrial membrane 8 homolog A (yeast)	-0,3849	0,7658	0,0002	0,0020
ENSG00000100575	TIMM9	translocase of inner mitochondrial membrane 9 homolog (yeast)	-0,4263	0,7442	0,0000	0,0005
ENSG00000100234	TIMP3	TIMP metallopeptidase inhibitor 3	0,5923	1,5076	0,0000	0,0000
ENSG00000142910	TINAGL1	tubulointerstitial nephritis antigen-like 1	-0,6417	0,6410	0,0000	0,0000
ENSG00000223573	TINCR	tissue differentiation-inducing non-protein coding RNA	0,8740	1,8328	0,0002	0,0023
ENSG00000105289	TJP3	tight junction protein 3	0,5284	1,4424	0,0004	0,0047
ENSG00000149476	TKFC	triokinase/FMN cyclase	0,6523	1,5717	0,0000	0,0000
ENSG00000160606	TLCD1	TLC domain containing 1	0,8851	1,8469	0,0000	0,0000
ENSG00000185561	TLCD2	TLC domain containing 2	0,7908	1,7300	0,0000	0,0000
ENSG00000101342	TLDC2	TBC/LysM-associated domain containing 2	0,4850	1,3996	0,0000	0,0006
ENSG00000104953	TLE6	transducin-like enhancer of split 6	0,7526	1,6849	0,0004	0,0047
ENSG00000095587	TLL2	tolloid-like 2	-0,9348	0,5231	0,0000	0,0000
ENSG00000137462	TLR2	toll-like receptor 2	-0,8153	0,5683	0,0000	0,0002
ENSG00000164342	TLR3	toll-like receptor 3	0,8363	1,7855	0,0000	0,0001
ENSG00000136869	TLR4	toll-like receptor 4	-0,7498	0,5947	0,0000	0,0000
ENSG00000169490	TM2D2	TM2 domain containing 2	-0,4136	0,7508	0,0000	0,0002
ENSG00000149809	TM7SF2	transmembrane 7 superfamily member 2	0,5295	1,4434	0,0000	0,0001
ENSG00000064115	TM7SF3	transmembrane 7 superfamily member 3	-0,4095	0,7529	0,0000	0,0001
ENSG00000125304	TM9SF2	transmembrane 9 superfamily member 2	-1,0136	0,4953	0,0000	0,0000
ENSG00000077147	TM9SF3	transmembrane 9 superfamily member 3	-0,5254	0,6948	0,0000	0,0000
ENSG00000155957	TMBIM4	transmembrane BAX inhibitor motif containing 4	-0,5775	0,6701	0,0000	0,0003
ENSG00000139644	TMBIM6	transmembrane BAX inhibitor motif containing 6	-0,4825	0,7158	0,0000	0,0000
ENSG00000167608	TMC4	transmembrane channel-like 4	1,3333	2,5199	0,0000	0,0000

ENSG00000141524	TMC6	transmembrane channel-like 6	0,4852	1,3998	0,0002	0,0026
ENSG00000167895	TMC8	transmembrane channel-like 8	0,7234	1,6510	0,0000	0,0000
ENSG00000172765	TMCC1	transmembrane and coiled-coil domain family 1	0,3910	1,3113	0,0000	0,0002
ENSG00000150403	TMCO3	transmembrane and coiled-coil domains 3	-0,6074	0,6564	0,0000	0,0000
ENSG00000099203	TMED1	transmembrane p24 trafficking protein 1	0,4306	1,3478	0,0010	0,0092
ENSG00000248592	TMEM110-MUSTN1	TMEM110-MUSTN1 readthrough	0,9629	1,9492	0,0003	0,0036
ENSG00000139173	TMEM117	transmembrane protein 117	0,4593	1,3749	0,0001	0,0012
ENSG00000179178	TMEM125	transmembrane protein 125	0,7928	1,7324	0,0000	0,0007
ENSG00000172663	TMEM134	transmembrane protein 134	0,7609	1,6945	0,0000	0,0000
ENSG00000244187	TMEM141	transmembrane protein 141	0,6826	1,6051	0,0000	0,0000
ENSG00000161558	TMEM143	transmembrane protein 143	1,0148	2,0206	0,0000	0,0000
ENSG00000249242	TMEM150C	transmembrane protein 150C	0,6593	1,5793	0,0000	0,0000
ENSG00000249992	TMEM158	transmembrane protein 158 (gene/pseudogene)	-1,0546	0,4814	0,0000	0,0000
ENSG00000064545	TMEM161A	transmembrane protein 161A	0,6367	1,5548	0,0000	0,0000
ENSG00000134851	TMEM165	transmembrane protein 165	-0,4692	0,7224	0,0000	0,0000
ENSG00000205269	TMEM170B	transmembrane protein 170B	-0,6642	0,6310	0,0000	0,0000
ENSG00000177854	TMEM187	transmembrane protein 187	0,5957	1,5112	0,0000	0,0006
ENSG00000182796	TMEM198B	transmembrane protein 198B, pseudogene	0,5136	1,4276	0,0000	0,0000
ENSG00000135048	TMEM2	transmembrane protein 2	0,4754	1,3904	0,0000	0,0000
ENSG00000164484	TMEM200A	transmembrane protein 200A	-2,3101	0,2016	0,0000	0,0000
ENSG00000253304	TMEM200B	transmembrane protein 200B	-0,5695	0,6739	0,0009	0,0082
ENSG00000205084	TMEM231	transmembrane protein 231	0,3927	1,3128	0,0002	0,0029
ENSG00000135185	TMEM243	transmembrane protein 243, mitochondrial	-0,4465	0,7338	0,0000	0,0002
ENSG00000133678	TMEM254	transmembrane protein 254	0,6875	1,6105	0,0000	0,0000
ENSG00000205544	TMEM256	transmembrane protein 256	0,5682	1,4826	0,0000	0,0000
ENSG00000182087	TMEM259	transmembrane protein 259	0,4395	1,3561	0,0005	0,0052
ENSG00000182107	TMEM30B	transmembrane protein 30B	-0,5811	0,6685	0,0001	0,0008
ENSG00000088726	TMEM40	transmembrane protein 40	0,7279	1,6563	0,0000	0,0000
ENSG00000169964	TMEM42	transmembrane protein 42	0,5773	1,4921	0,0000	0,0000
ENSG00000231770	TMEM44-AS1	TMEM44 antisense RNA 1	0,6275	1,5449	0,0001	0,0009
ENSG00000147027	TMEM47	transmembrane protein 47	-0,5550	0,6806	0,0000	0,0000

ENSG00000118600	TMEM5	transmembrane protein 5	-0,5543	0,6810	0,0000	0,0001
ENSG00000175147	TMEM51-AS1	TMEM51 antisense RNA 1	-0,4484	0,7328	0,0007	0,0067
ENSG00000178821	TMEM52	transmembrane protein 52	0,5803	1,4952	0,0003	0,0031
ENSG00000126106	TMEM53	transmembrane protein 53	0,5375	1,4515	0,0002	0,0024
ENSG00000121900	TMEM54	transmembrane protein 54	0,6850	1,6078	0,0000	0,0000
ENSG00000165782	TMEM55B	transmembrane protein 55B	0,5485	1,4626	0,0000	0,0000
ENSG00000177042	TMEM80	transmembrane protein 80	0,4840	1,3986	0,0000	0,0001
ENSG00000151117	TMEM86A	transmembrane protein 86A	0,9045	1,8718	0,0000	0,0000
ENSG00000153214	TMEM87B	transmembrane protein 87B	-0,3955	0,7602	0,0000	0,0002
ENSG00000137103	TMEM8B	transmembrane protein 8B	0,9378	1,9156	0,0000	0,0000
ENSG00000167105	TMEM92	transmembrane protein 92	-0,5579	0,6793	0,0000	0,0000
ENSG00000177728	TMEM94	transmembrane protein 94	0,3970	1,3168	0,0004	0,0044
ENSG00000109084	TMEM97	transmembrane protein 97	-0,7451	0,5966	0,0000	0,0000
ENSG00000254860	TMEM9B-AS1	TMEM9B antisense RNA 1	0,9583	1,9430	0,0000	0,0003
ENSG00000136842	TMOD1	tropomodulin 1	0,9497	1,9314	0,0000	0,0000
ENSG00000128872	TMOD2	tropomodulin 2 (neuronal)	-0,3858	0,7654	0,0002	0,0023
ENSG00000160183	TMPRSS3	transmembrane protease, serine 3	1,0553	2,0782	0,0000	0,0000
ENSG00000137648	TMPRSS4	transmembrane protease, serine 4	0,9301	1,9054	0,0003	0,0031
ENSG00000187045	TMPRSS6	transmembrane protease, serine 6	1,0260	2,0364	0,0002	0,0022
ENSG00000125247	TMTC4	transmembrane and tetratricopeptide repeat containing 4	-0,4838	0,7151	0,0001	0,0009
ENSG00000139921	TMX1	thioredoxin-related transmembrane protein 1	-0,4620	0,7260	0,0000	0,0000
ENSG00000213593	TMX2	thioredoxin-related transmembrane protein 2	-0,5075	0,7034	0,0000	0,0000
ENSG00000041982	TNC	tenascin C	-1,1911	0,4380	0,0000	0,0000
ENSG00000185215	TNFAIP2	tumor necrosis factor, alpha-induced protein 2	-0,4474	0,7334	0,0000	0,0001
ENSG00000118503	TNFAIP3	tumor necrosis factor, alpha-induced protein 3	-0,5841	0,6671	0,0001	0,0017
ENSG00000173530	TNFRSF10D	tumor necrosis factor receptor superfamily, member 10d, decoy with truncated de	-0,5235	0,6957	0,0000	0,0000
ENSG00000164761	TNFRSF11B	tumor necrosis factor receptor superfamily, member 11b	0,5601	1,4743	0,0000	0,0000
ENSG00000157873	TNFRSF14	tumor necrosis factor receptor superfamily, member 14	0,6743	1,5958	0,0000	0,0005
ENSG00000186891	TNFRSF18	tumor necrosis factor receptor superfamily, member 18	0,8199	1,7653	0,0000	0,0001
ENSG00000127863	TNFRSF19	tumor necrosis factor receptor superfamily, member 19	0,4327	1,3498	0,0000	0,0000
ENSG00000049249	TNFRSF9	tumor necrosis factor receptor superfamily, member 9	-0,7409	0,5984	0,0000	0,0000

ENSG00000121858	TNFSF10	tumor necrosis factor (ligand) superfamily, member 10	1,0816	2,1163	0,0000	0,0000
ENSG00000125657	TNFSF9	tumor necrosis factor (ligand) superfamily, member 9	0,5246	1,4385	0,0000	0,0005
ENSG00000114854	TNNC1	troponin C type 1 (slow)	0,3932	1,3133	0,0001	0,0019
ENSG00000083312	TNPO1	transportin 1	-0,6451	0,6395	0,0000	0,0000
ENSG00000204282	TNRC6C-AS1	TNRC6C antisense RNA 1	0,3966	1,3164	0,0006	0,0058
ENSG00000079308	TNS1	tensin 1	1,1719	2,2531	0,0000	0,0000
ENSG00000136205	TNS3	tensin 3	-0,5744	0,6716	0,0000	0,0000
ENSG00000100284	TOM1	target of myb1 membrane trafficking protein	0,6289	1,5463	0,0000	0,0000
ENSG00000158882	TOMM40L	translocase of outer mitochondrial membrane 40 homolog (yeast)-like	-0,4018	0,7569	0,0000	0,0007
ENSG00000198900	TOP1	topoisomerase (DNA) I	-0,6262	0,6479	0,0000	0,0000
ENSG00000164938	TP53INP1	tumor protein p53 inducible nuclear protein 1	0,9021	1,8688	0,0000	0,0000
ENSG00000172315	TP53RK	TP53 regulating kinase	-0,4369	0,7387	0,0002	0,0019
ENSG00000111907	TPD52L1	tumor protein D52-like 1	-0,4591	0,7274	0,0000	0,0000
ENSG00000143549	TPM3	tropomyosin 3	-0,4704	0,7217	0,0000	0,0000
ENSG00000134900	TPP2	tripeptidyl peptidase II	-1,0476	0,4838	0,0000	0,0000
ENSG00000171368	TPPP	tubulin polymerization promoting protein	1,1490	2,2177	0,0000	0,0000
ENSG00000159713	TPPP3	tubulin polymerization-promoting protein family member 3	1,3749	2,5935	0,0000	0,0000
ENSG00000180438	TPRXL	tetra-peptide repeat homeobox-like	0,4592	1,3747	0,0009	0,0082
ENSG00000116176	TPSG1	tryptase gamma 1	1,8164	3,5221	0,0000	0,0000
ENSG00000260284	TPSP2	tryptase pseudogene 2	0,9841	1,9781	0,0003	0,0037
ENSG00000133112	TPT1	tumor protein, translationally-controlled 1	-0,4138	0,7506	0,0000	0,0001
ENSG00000100181	TPTEP1	transmembrane phosphatase with tensin homology pseudogene 1	-0,4648	0,7246	0,0001	0,0017
ENSG00000186854	TRABD2A	TraB domain containing 2A	-0,5106	0,7019	0,0000	0,0000
ENSG00000102871	TRADD	TNFRSF1A-associated via death domain	0,4270	1,3444	0,0003	0,0038
ENSG00000131323	TRAF3	TNF receptor-associated factor 3	-0,5273	0,6939	0,0000	0,0000
ENSG00000056972	TRAF3IP2	TRAF3 interacting protein 2	0,8102	1,7535	0,0008	0,0081
ENSG00000009790	TRAF3IP3	TRAF3 interacting protein 3	0,9999	1,9998	0,0000	0,0000
ENSG00000168016	TRANK1	tetratricopeptide repeat and ankyrin repeat containing 1	0,9916	1,9883	0,0000	0,0000
ENSG00000007255	TRAPPC6A	trafficking protein particle complex 6A	0,5557	1,4699	0,0001	0,0014
ENSG00000211829	TRDC	T cell receptor delta constant	2,6047	6,0824	0,0000	0,0000
ENSG00000095970	TREM2	triggering receptor expressed on myeloid cells 2	1,2047	2,3049	0,0000	0,0000

ENSG00000173334	TRIB1	tribbles pseudokinase 1	-0,4151	0,7500	0,0000	0,0000
ENSG00000101255	TRIB3	tribbles pseudokinase 3	1,0338	2,0474	0,0000	0,0000
ENSG00000204977	TRIM13	tripartite motif containing 13	-0,4828	0,7156	0,0000	0,0000
ENSG00000221926	TRIM16	tripartite motif containing 16	-0,4110	0,7521	0,0000	0,0000
ENSG00000104228	TRIM35	tripartite motif containing 35	-0,6466	0,6388	0,0000	0,0000
ENSG00000134253	TRIM45	tripartite motif containing 45	0,6740	1,5955	0,0000	0,0000
ENSG00000138100	TRIM54	tripartite motif containing 54	0,9313	1,9070	0,0005	0,0055
ENSG00000147573	TRIM55	tripartite motif containing 55	-1,0805	0,4729	0,0001	0,0011
ENSG00000178809	TRIM73	tripartite motif containing 73	1,0326	2,0457	0,0000	0,0001
ENSG00000100815	TRIP11	thyroid hormone receptor interactor 11	-0,4226	0,7461	0,0000	0,0000
ENSG00000205133	TRIQK	triple QxxK/R motif containing	0,4124	1,3309	0,0002	0,0028
ENSG00000174173	TRMT10C	tRNA methyltransferase 10C, mitochondrial RNase P subunit	-0,4328	0,7408	0,0006	0,0060
ENSG00000122435	TRMT13	tRNA methyltransferase 13 homolog (S. cerevisiae)	-0,4298	0,7424	0,0001	0,0018
ENSG00000089195	TRMT6	tRNA methyltransferase 6	-0,3948	0,7606	0,0004	0,0042
ENSG00000166166	TRMT61A	tRNA methyltransferase 61A	-0,4862	0,7139	0,0001	0,0016
ENSG00000253368	TRNP1	TMF1-regulated nuclear protein 1	-0,4615	0,7262	0,0001	0,0010
ENSG00000072756	TRNT1	tRNA nucleotidyl transferase, CCA-adding, 1	-0,5742	0,6717	0,0000	0,0000
ENSG00000230061	TRPM2-AS	TRPM2 antisense RNA	0,4687	1,3839	0,0001	0,0018
ENSG00000119121	TRPM6	transient receptor potential cation channel, subfamily M, member 6	-0,6280	0,6471	0,0004	0,0040
ENSG00000144481	TRPM8	transient receptor potential cation channel, subfamily M, member 8	1,2398	2,3617	0,0000	0,0000
ENSG00000104447	TRPS1	trichorhinophalangeal syndrome I	-2,1598	0,2238	0,0000	0,0000
ENSG00000149743	TRPT1	tRNA phosphotransferase 1	0,4049	1,3240	0,0000	0,0005
ENSG00000196689	TRPV1	transient receptor potential cation channel, subfamily V, member 1	0,8912	1,8548	0,0000	0,0000
ENSG00000167723	TRPV3	transient receptor potential cation channel, subfamily V, member 3	0,9679	1,9559	0,0001	0,0009
ENSG00000111199	TRPV4	transient receptor potential cation channel, subfamily V, member 4	0,9840	1,9779	0,0000	0,0000
ENSG00000165125	TRPV6	transient receptor potential cation channel, subfamily V, member 6	2,5499	5,8559	0,0000	0,0000
ENSG00000102804	TSC22D1	TSC22 domain family, member 1	-0,4485	0,7328	0,0000	0,0000
ENSG00000157514	TSC22D3	TSC22 domain family, member 3	1,6942	3,2359	0,0000	0,0000
ENSG00000182463	TSHZ2	teashirt zinc finger homeobox 2	0,9215	1,8941	0,0000	0,0000
ENSG00000121297	TSHZ3	teashirt zinc finger homeobox 3	-1,2474	0,4212	0,0000	0,0000
ENSG00000145777	TSLP	thymic stromal lymphopoietin	0,9787	1,9707	0,0000	0,0001

ENSG00000171045	TSNARE1	t-SNARE domain containing 1	0,8877	1,8502	0,0000	0,0000
ENSG00000117472	TSPAN1	tetraspanin 1	0,8184	1,7634	0,0000	0,0000
ENSG00000106537	TSPAN13	tetraspanin 13	-0,5822	0,6679	0,0000	0,0000
ENSG00000099282	TSPAN15	tetraspanin 15	0,5660	1,4804	0,0000	0,0006
ENSG00000134198	TSPAN2	tetraspanin 2	-0,7159	0,6088	0,0000	0,0000
ENSG00000000003	TSPAN6	tetraspanin 6	-0,5614	0,6776	0,0000	0,0000
ENSG00000127324	TSPAN8	tetraspanin 8	2,2302	4,6919	0,0000	0,0000
ENSG00000167721	TSR1	TSR1, 20S rRNA accumulation, homolog (S. cerevisiae)	-0,4121	0,7515	0,0000	0,0002
ENSG00000178093	TSSK6	testis-specific serine kinase 6	0,7898	1,7288	0,0010	0,0096
ENSG00000128881	TTBK2	tau tubulin kinase 2	0,3802	1,3015	0,0000	0,0001
ENSG00000113312	TTC1	tetratricopeptide repeat domain 1	-0,4172	0,7489	0,0000	0,0007
ENSG00000143643	TTC13	tetratricopeptide repeat domain 13	-0,5234	0,6957	0,0000	0,0000
ENSG00000183891	TTC32	tetratricopeptide repeat domain 32	0,4146	1,3329	0,0002	0,0020
ENSG00000113638	TTC33	tetratricopeptide repeat domain 33	0,4152	1,3335	0,0006	0,0065
ENSG00000215912	TTC34	tetratricopeptide repeat domain 34	0,8815	1,8423	0,0000	0,0002
ENSG00000085831	TTC39A	tetratricopeptide repeat domain 39A	0,5349	1,4489	0,0007	0,0072
ENSG00000168234	TTC39C	tetratricopeptide repeat domain 39C	-0,7451	0,5966	0,0000	0,0000
ENSG00000116830	TTF2	transcription termination factor, RNA polymerase II	-0,4696	0,7222	0,0000	0,0000
ENSG00000100271	TTLL1	tubulin tyrosine ligase-like family member 1	0,8429	1,7936	0,0000	0,0001
ENSG00000137561	TTPA	tocopherol (alpha) transfer protein	0,6804	1,6025	0,0000	0,0000
ENSG00000141540	TTYH2	tweety family member 2	0,9255	1,8994	0,0001	0,0011
ENSG00000123416	TUBA1B	tubulin, alpha 1b	-0,4540	0,7300	0,0000	0,0000
ENSG00000167553	TUBA1C	tubulin, alpha 1c	-0,4734	0,7203	0,0000	0,0000
ENSG00000127824	TUBA4A	tubulin, alpha 4a	-0,3850	0,7658	0,0000	0,0001
ENSG00000074935	TUBE1	tubulin, epsilon 1	0,4395	1,3561	0,0001	0,0014
ENSG00000037042	TUBG2	tubulin, gamma 2	0,6043	1,5202	0,0000	0,0000
ENSG00000126216	TUBGCP3	tubulin, gamma complex associated protein 3	-0,4833	0,7154	0,0000	0,0001
ENSG00000149016	TUT1	terminal uridylyl transferase 1, U6 snRNA-specific	0,6162	1,5328	0,0000	0,0000
ENSG00000151239	TWF1	twinfilin actin binding protein 1	-0,3888	0,7638	0,0000	0,0005
ENSG00000105849	TWISTNB	TWIST neighbor	-0,6054	0,6573	0,0000	0,0000
ENSG00000128791	TWSG1	twisted gastrulation BMP signaling modulator 1	-0,4646	0,7247	0,0000	0,0000

ENSG00000086712	TXLNG	taxilin gamma	-0,4367	0,7388	0,0000	0,0002
ENSG00000115514	TXNDC9	thioredoxin domain containing 9	-0,4736	0,7202	0,0000	0,0001
ENSG00000265972	TXNIP	thioredoxin interacting protein	0,6031	1,5189	0,0000	0,0000
ENSG00000163714	U2SURP	U2 snRNP-associated SURP domain containing	-0,5130	0,7008	0,0000	0,0000
ENSG00000197355	UAP1L1	UDP-N-acetylglucosamine pyrophosphorylase 1 like 1	0,4383	1,3550	0,0001	0,0012
ENSG00000182179	UBA7	ubiquitin-like modifier activating enzyme 7	1,0867	2,1239	0,0000	0,0000
ENSG00000134882	UBAC2	UBA domain containing 2	-0,6122	0,6542	0,0000	0,0000
ENSG00000185262	UBALD2	UBA-like domain containing 2	0,4658	1,3811	0,0000	0,0004
ENSG00000154127	UBASH3B	ubiquitin associated and SH3 domain containing B	-0,3971	0,7594	0,0000	0,0000
ENSG00000170142	UBE2E1	ubiquitin-conjugating enzyme E2E 1	-0,4186	0,7482	0,0000	0,0002
ENSG00000170035	UBE2E3	ubiquitin-conjugating enzyme E2E 3	-0,5022	0,7061	0,0000	0,0001
ENSG00000184182	UBE2F	ubiquitin-conjugating enzyme E2F (putative)	-0,3791	0,7689	0,0004	0,0043
ENSG00000184787	UBE2G2	ubiquitin-conjugating enzyme E2G 2	-0,4252	0,7447	0,0000	0,0000
ENSG00000156587	UBE2L6	ubiquitin-conjugating enzyme E2L 6	0,4017	1,3210	0,0008	0,0078
ENSG00000177889	UBE2N	ubiquitin-conjugating enzyme E2N	-0,5654	0,6758	0,0000	0,0000
ENSG00000177414	UBE2U	ubiquitin-conjugating enzyme E2U (putative)	1,8627	3,6368	0,0000	0,0000
ENSG00000169139	UBE2V2	ubiquitin-conjugating enzyme E2 variant 2	-0,4363	0,7390	0,0000	0,0005
ENSG00000114062	UBE3A	ubiquitin protein ligase E3A	-0,5582	0,6792	0,0000	0,0000
ENSG00000122042	UBL3	ubiquitin-like 3	-0,4060	0,7547	0,0000	0,0002
ENSG00000138629	UBL7	ubiquitin-like 7	0,4103	1,3290	0,0000	0,0003
ENSG00000164332	UBLCP1	ubiquitin-like domain containing CTD phosphatase 1	-0,4124	0,7514	0,0000	0,0003
ENSG00000153560	UBP1	upstream binding protein 1 (LBP-1a)	-0,4150	0,7500	0,0000	0,0000
ENSG00000225986	UBXN10-AS1	UBXN10 antisense RNA 1	-0,7027	0,6144	0,0000	0,0001
ENSG00000158062	UBXN11	UBX domain protein 11	0,3982	1,3179	0,0008	0,0080
ENSG00000167671	UBXN6	UBX domain protein 6	0,4450	1,3613	0,0002	0,0027
ENSG00000104691	UBXN8	UBX domain protein 8	-0,7827	0,5813	0,0000	0,0000
ENSG00000118939	UCHL3	ubiquitin carboxyl-terminal esterase L3 (ubiquitin thiolesterase)	-0,8655	0,5488	0,0000	0,0000
ENSG00000175567	UCP2	uncoupling protein 2 (mitochondrial, proton carrier)	0,7050	1,6301	0,0000	0,0000
ENSG00000120686	UFM1	ubiquitin-fold modifier 1	-0,4947	0,7097	0,0000	0,0000
ENSG00000148154	UGCG	UDP-glucose ceramide glucosyltransferase	-0,6886	0,6204	0,0000	0,0000
ENSG00000102595	UGGT2	UDP-glucose glycoprotein glucosyltransferase 2	-0,8820	0,5426	0,0000	0,0000

ENSG00000065060	UHRF1BP1	UHRF1 binding protein 1	0,4226	1,3403	0,0000	0,0000
ENSG00000111981	ULBP1	UL16 binding protein 1	1,0220	2,0307	0,0000	0,0000
ENSG00000131019	ULBP3	UL16 binding protein 3	0,5154	1,4294	0,0000	0,0005
ENSG00000177169	ULK1	unc-51 like autophagy activating kinase 1	1,0403	2,0567	0,0000	0,0000
ENSG00000083290	ULK2	unc-51 like autophagy activating kinase 2	0,4478	1,3640	0,0003	0,0036
ENSG00000168038	ULK4	unc-51 like kinase 4	-0,4417	0,7363	0,0000	0,0001
ENSG00000114491	UMPS	uridine monophosphate synthetase	-0,4326	0,7409	0,0000	0,0000
ENSG00000130477	UNC13A	unc-13 homolog A (C. elegans)	-0,6040	0,6579	0,0007	0,0071
ENSG00000169062	UPF3A	UPF3 regulator of nonsense transcripts homolog A (yeast)	-0,8380	0,5594	0,0000	0,0000
ENSG00000226510	UPK1A-AS1	UPK1A antisense RNA 1	1,0619	2,0877	0,0000	0,0006
ENSG00000137288	UQCC2	ubiquinol-cytochrome c reductase complex assembly factor 2	0,3807	1,3019	0,0000	0,0006
ENSG00000169021	UQCRFS1	ubiquinol-cytochrome c reductase, Rieske iron-sulfur polypeptide 1	-0,4052	0,7551	0,0002	0,0021
ENSG00000135763	URB2	URB2 ribosome biogenesis 2 homolog (S. cerevisiae)	-0,6961	0,6172	0,0000	0,0000
ENSG00000159650	UROC1	urocanate hydratase 1	0,6902	1,6135	0,0000	0,0000
ENSG00000103005	USB1	U6 snRNA biogenesis 1	-0,4142	0,7504	0,0000	0,0000
ENSG00000006611	USH1C	Usher syndrome 1C	0,9880	1,9835	0,0000	0,0000
ENSG00000152484	USP12	ubiquitin specific peptidase 12	-0,6692	0,6288	0,0000	0,0000
ENSG00000058056	USP13	ubiquitin specific peptidase 13 (isopeptidase T-3)	-0,4402	0,7371	0,0000	0,0000
ENSG00000101557	USP14	ubiquitin specific peptidase 14 (tRNA-guanine transglycosylase)	-0,3959	0,7600	0,0000	0,0004
ENSG00000135655	USP15	ubiquitin specific peptidase 15	-0,4778	0,7181	0,0000	0,0000
ENSG00000156256	USP16	ubiquitin specific peptidase 16	-0,4020	0,7568	0,0000	0,0001
ENSG00000136878	USP20	ubiquitin specific peptidase 20	0,4460	1,3623	0,0002	0,0024
ENSG00000273820	USP27X	ubiquitin specific peptidase 27, X-linked	-0,6645	0,6309	0,0000	0,0000
ENSG00000135093	USP30	ubiquitin specific peptidase 30	0,4247	1,3423	0,0005	0,0049
ENSG00000148429	USP6NL	USP6 N-terminal like	-0,4408	0,7367	0,0000	0,0000
ENSG00000156697	UTP14A	UTP14A small subunit (SSU) processome component	-0,8124	0,5694	0,0000	0,0000
ENSG00000253797	UTP14C	UTP14, U3 small nucleolar ribonucleoprotein, homolog C (yeast)	-0,7174	0,6082	0,0000	0,0000
ENSG00000164338	UTP15	UTP15, U3 small nucleolar ribonucleoprotein, homolog (S. cerevisiae)	-0,5710	0,6731	0,0000	0,0000
ENSG00000011260	UTP18	UTP18 small subunit (SSU) processome component	-0,3790	0,7690	0,0003	0,0032
ENSG00000120800	UTP20	UTP20 small subunit (SSU) processome component	-0,7533	0,5932	0,0000	0,0000
ENSG00000147679	UTP23	UTP23, small subunit (SSU) processome component, homolog (yeast)	-0,5322	0,6915	0,0000	0,0000

ENSG00000132467	UTP3	UTP3, small subunit (SSU) processome component, homolog (S. cerevisiae)	-0,4049	0,7553	0,0000	0,0003
ENSG00000220205	VAMP2	vesicle-associated membrane protein 2 (synaptobrevin 2)	0,4657	1,3810	0,0000	0,0000
ENSG00000112715	VEGFA	vascular endothelial growth factor A	0,5386	1,4525	0,0000	0,0000
ENSG00000197415	VEPH1	ventricular zone expressed PH domain-containing 1	0,4728	1,3878	0,0010	0,0091
ENSG00000028203	VEZT	vezatin, adherens junctions transmembrane protein	-0,3899	0,7632	0,0000	0,0000
ENSG00000128564	VGF	VGF nerve growth factor inducible	-0,4118	0,7517	0,0002	0,0020
ENSG00000206538	VGLL3	vestigial-like family member 3	0,8543	1,8078	0,0010	0,0094
ENSG00000127831	VIL1	villin 1	1,7526	3,3695	0,0000	0,0000
ENSG00000136059	VILL	villin-like	1,3568	2,5612	0,0000	0,0000
ENSG00000229124	VIM-AS1	VIM antisense RNA 1	-0,4616	0,7262	0,0005	0,0048
ENSG00000147852	VLDLR	very low density lipoprotein receptor	-0,5560	0,6802	0,0000	0,0000
ENSG00000062716	VMP1	vacuole membrane protein 1	-0,4841	0,7150	0,0000	0,0000
ENSG00000160948	VPS28	vacuolar protein sorting 28 homolog (S. cerevisiae)	0,4626	1,3780	0,0001	0,0011
ENSG00000136100	VPS36	vacuolar protein sorting 36 homolog (S. cerevisiae)	-0,6301	0,6461	0,0000	0,0000
ENSG00000176428	VPS37D	vacuolar protein sorting 37 homolog D (S. cerevisiae)	1,0828	2,1182	0,0000	0,0000
ENSG00000100749	VRK1	vaccinia related kinase 1	-0,4499	0,7321	0,0000	0,0005
ENSG00000186806	VSIG10L	V-set and immunoglobulin domain containing 10 like	0,6164	1,5330	0,0000	0,0000
ENSG00000100568	VTI1B	vesicle transport through interaction with t-SNAREs 1B	-0,3942	0,7609	0,0000	0,0005
ENSG00000204396	VWA7	von Willebrand factor A domain containing 7	0,8048	1,7469	0,0001	0,0010
ENSG00000102763	VWA8	von Willebrand factor A domain containing 8	-0,4849	0,7145	0,0000	0,0000
ENSG00000146530	VWDE	von Willebrand factor D and EGF domains	0,6176	1,5343	0,0002	0,0020
ENSG00000110799	VWF	von Willebrand factor	1,0256	2,0357	0,0000	0,0000
ENSG00000140105	WARS	tryptophanyl-tRNA synthetase	0,4795	1,3942	0,0000	0,0000
ENSG00000132970	WASF3	WAS protein family, member 3	-0,4161	0,7494	0,0000	0,0000
ENSG00000132471	WBP2	WW domain binding protein 2	0,5475	1,4615	0,0000	0,0000
ENSG00000120688	WBP4	WW domain binding protein 4	-0,4218	0,7465	0,0000	0,0004
ENSG00000165171	WBSCR27	Williams Beuren syndrome chromosome region 27	1,2611	2,3968	0,0000	0,0000
ENSG00000101940	WDR13	WD repeat domain 13	0,4708	1,3859	0,0002	0,0020
ENSG00000157796	WDR19	WD repeat domain 19	0,7034	1,6284	0,0000	0,0000
ENSG00000140153	WDR20	WD repeat domain 20	-0,3868	0,7648	0,0000	0,0002
ENSG00000065183	WDR3	WD repeat domain 3	-0,4725	0,7207	0,0000	0,0002

ENSG00000148225	WDR31	WD repeat domain 31	0,6162	1,5328	0,0001	0,0017
ENSG00000118965	WDR35	WD repeat domain 35	-0,3992	0,7583	0,0000	0,0001
ENSG00000134987	WDR36	WD repeat domain 36	-0,5117	0,7014	0,0000	0,0000
ENSG00000160193	WDR4	WD repeat domain 4	-0,6207	0,6504	0,0000	0,0000
ENSG00000163811	WDR43	WD repeat domain 43	-0,7418	0,5980	0,0000	0,0000
ENSG00000005448	WDR54	WD repeat domain 54	0,6423	1,5608	0,0000	0,0000
ENSG00000116455	WDR77	WD repeat domain 77	-0,7116	0,6106	0,0000	0,0000
ENSG00000123154	WDR83	WD repeat domain 83	0,5032	1,4174	0,0001	0,0010
ENSG00000228775	WEE2-AS1	WEE2 antisense RNA 1	0,9110	1,8804	0,0009	0,0084
ENSG00000101443	WFDC2	WAP four-disulfide core domain 2	-0,7827	0,5813	0,0000	0,0000
ENSG00000109501	WFS1	Wolfram syndrome 1 (wolframin)	0,4698	1,3849	0,0001	0,0008
ENSG00000070540	WIPI1	WD repeat domain, phosphoinositide interacting 1	0,6702	1,5913	0,0000	0,0000
ENSG00000085741	WNT11	wingless-type MMTV integration site family, member 11	0,9689	1,9573	0,0000	0,0000
ENSG00000114251	WNT5A	wingless-type MMTV integration site family, member 5A	-0,9559	0,5155	0,0000	0,0000
ENSG00000182093	WRB	tryptophan rich basic protein	0,3882	1,3087	0,0000	0,0007
ENSG00000151718	WWC2	WW and C2 domain containing 2	-0,4827	0,7156	0,0000	0,0000
ENSG00000047644	WWC3	WWC family member 3	-0,4198	0,7475	0,0000	0,0002
ENSG00000018408	WWTR1	WW domain containing transcription regulator 1	-0,5161	0,6993	0,0000	0,0000
ENSG00000076924	XAB2	XPA binding protein 2	0,4867	1,4012	0,0000	0,0002
ENSG00000260903	XKR7	XK, Kell blood group complex subunit-related family, member 7	-1,5288	0,3466	0,0000	0,0000
ENSG00000122121	XPNPEP2	X-prolyl aminopeptidase (aminopeptidase P) 2, membrane-bound	0,7450	1,6760	0,0007	0,0068
ENSG00000132953	XPO4	exportin 4	-0,5928	0,6630	0,0000	0,0000
ENSG00000212556	Y_RNA	Y RNA	-1,3027	0,4054	0,0000	0,0000
ENSG00000241127	YAE1D1	Yae1 domain containing 1	-0,4077	0,7538	0,0011	0,0098
ENSG00000006047	YBX2	Y box binding protein 2	0,3798	1,3012	0,0000	0,0001
ENSG00000167645	YIF1B	Yip1 interacting factor homolog B (S. cerevisiae)	0,4055	1,3246	0,0002	0,0021
ENSG00000130733	YIPF2	Yip1 domain family, member 2	0,4524	1,3683	0,0001	0,0013
ENSG00000100027	YPEL1	yippee-like 1	1,8051	3,4945	0,0000	0,0000
ENSG00000175155	YPEL2	yippee-like 2	1,0255	2,0356	0,0000	0,0000
ENSG00000090238	YPEL3	yippee-like 3	1,1430	2,2084	0,0000	0,0000
ENSG00000166793	YPEL4	yippee-like 4	1,3391	2,5299	0,0000	0,0000

ENSG00000166913	YWHAB	tyrosine 3-monooxygenase/tryptophan 5-monooxygenase activation protein, beta	-0,5156	0,6995	0,0000	0,0000
ENSG00000128245	YWHAH	tyrosine 3-monooxygenase/tryptophan 5-monooxygenase activation protein, eta	-0,4129	0,7511	0,0000	0,0007
ENSG00000164924	YWHAZ	tyrosine 3-monooxygenase/tryptophan 5-monooxygenase activation protein, zeta	-0,4446	0,7348	0,0000	0,0000
ENSG00000221886	ZBED8	zinc finger, BED-type containing 8	0,4137	1,3321	0,0001	0,0012
ENSG00000236104	ZBTB22	zinc finger and BTB domain containing 22	0,7610	1,6947	0,0000	0,0000
ENSG00000112365	ZBTB24	zinc finger and BTB domain containing 24	-0,4570	0,7285	0,0000	0,0003
ENSG00000114853	ZBTB47	zinc finger and BTB domain containing 47	0,6227	1,5398	0,0008	0,0079
ENSG00000104427	ZC2HC1A	zinc finger, C2HC-type containing 1A	0,6447	1,5634	0,0000	0,0000
ENSG00000149289	ZC3H12C	zinc finger CCCH-type containing 12C	-0,4550	0,7295	0,0000	0,0000
ENSG00000123200	ZC3H13	zinc finger CCCH-type containing 13	-0,4889	0,7126	0,0000	0,0000
ENSG00000100722	ZC3H14	zinc finger CCCH-type containing 14	-0,4004	0,7577	0,0000	0,0001
ENSG00000065548	ZC3H15	zinc finger CCCH-type containing 15	-0,5097	0,7023	0,0000	0,0000
ENSG00000188177	ZC3H6	zinc finger CCCH-type containing 6	0,7820	1,7195	0,0000	0,0000
ENSG00000126970	ZC4H2	zinc finger, C4H2 domain containing	-0,9079	0,5330	0,0000	0,0004
ENSG00000160446	ZDHHC12	zinc finger, DHHC-type containing 12	0,5028	1,4169	0,0006	0,0059
ENSG00000175048	ZDHHC14	zinc finger, DHHC-type containing 14	-0,9099	0,5322	0,0000	0,0000
ENSG00000180776	ZDHHC20	zinc finger, DHHC-type containing 20	-0,7445	0,5969	0,0000	0,0000
ENSG00000023041	ZDHHC6	zinc finger, DHHC-type containing 6	-0,4732	0,7204	0,0000	0,0000
ENSG00000133519	ZDHHC8P1	zinc finger, DHHC-type containing 8 pseudogene 1	0,7589	1,6922	0,0000	0,0000
ENSG00000237036	ZEB1-AS1	ZEB1 antisense RNA 1	0,4160	1,3342	0,0003	0,0037
ENSG00000160445	ZER1	zyg-11 related, cell cycle regulator	0,6815	1,6038	0,0000	0,0000
ENSG00000107372	ZFAND5	zinc finger, AN1-type domain 5	-0,3889	0,7637	0,0000	0,0003
ENSG00000128016	ZFP36	ZFP36 ring finger protein	0,5526	1,4667	0,0000	0,0000
ENSG00000165861	ZFYVE1	zinc finger, FYVE domain containing 1	0,5870	1,5021	0,0000	0,0000
ENSG00000043355	ZIC2	Zic family member 2	-0,9916	0,5029	0,0000	0,0000
ENSG00000139800	ZIC5	Zic family member 5	-1,0040	0,4986	0,0000	0,0000
ENSG00000166432	ZMAT1	zinc finger, matrin-type 1	-0,8042	0,5727	0,0001	0,0012
ENSG00000100319	ZMAT5	zinc finger, matrin-type 5	0,5404	1,4543	0,0000	0,0003
ENSG00000224596	ZMIZ1-AS1	ZMIZ1 antisense RNA 1	1,0261	2,0365	0,0002	0,0021
ENSG00000084073	ZMPSTE24	zinc metallopeptidase STE24	-0,6692	0,6289	0,0000	0,0000
ENSG00000197020	ZNF100	zinc finger protein 100	-0,5232	0,6958	0,0000	0,0002

ENSG00000152926	ZNF117	zinc finger protein 117	-0,5035	0,7054	0,0004	0,0042
ENSG00000167635	ZNF146	zinc finger protein 146	-0,4038	0,7558	0,0000	0,0006
ENSG00000197279	ZNF165	zinc finger protein 165	0,4267	1,3441	0,0001	0,0008
ENSG00000154957	ZNF18	zinc finger protein 18	0,4604	1,3760	0,0001	0,0009
ENSG00000167384	ZNF180	zinc finger protein 180	-0,6710	0,6281	0,0000	0,0000
ENSG00000005801	ZNF195	zinc finger protein 195	-0,4436	0,7353	0,0000	0,0002
ENSG00000166261	ZNF202	zinc finger protein 202	-0,4075	0,7539	0,0000	0,0001
ENSG00000204789	ZNF204P	zinc finger protein 204, pseudogene	1,0207	2,0288	0,0000	0,0000
ENSG00000165512	ZNF22	zinc finger protein 22	-0,4535	0,7303	0,0000	0,0000
ENSG00000213096	ZNF254	zinc finger protein 254	-0,3839	0,7664	0,0003	0,0034
ENSG00000198393	ZNF26	zinc finger protein 26	-0,5839	0,6672	0,0000	0,0000
ENSG00000063587	ZNF275	zinc finger protein 275	-0,5671	0,6749	0,0000	0,0000
ENSG00000137871	ZNF280D	zinc finger protein 280D	-0,3811	0,7679	0,0004	0,0041
ENSG00000167637	ZNF283	zinc finger protein 283	-0,4165	0,7492	0,0002	0,0020
ENSG00000130844	ZNF331	zinc finger protein 331	-0,4388	0,7377	0,0000	0,0002
ENSG00000230753	ZNF341-AS1	ZNF341 antisense RNA 1	-0,4907	0,7117	0,0002	0,0020
ENSG00000169981	ZNF35	zinc finger protein 35	-0,5638	0,6765	0,0000	0,0000
ENSG00000198816	ZNF358	zinc finger protein 358	0,7406	1,6708	0,0000	0,0000
ENSG00000144331	ZNF385B	zinc finger protein 385B	1,4305	2,6955	0,0000	0,0000
ENSG00000186918	ZNF395	zinc finger protein 395	0,5820	1,4969	0,0000	0,0000
ENSG00000186812	ZNF397	zinc finger protein 397	0,6208	1,5378	0,0000	0,0001
ENSG00000083838	ZNF446	zinc finger protein 446	0,6333	1,5511	0,0000	0,0001
ENSG00000148143	ZNF462	zinc finger protein 462	-0,4466	0,7338	0,0000	0,0000
ENSG00000198464	ZNF480	zinc finger protein 480	-0,4059	0,7548	0,0001	0,0008
ENSG00000243943	ZNF512	zinc finger protein 512	-0,5054	0,7045	0,0000	0,0001
ENSG00000163795	ZNF513	zinc finger protein 513	0,4878	1,4023	0,0002	0,0020
ENSG00000172006	ZNF554	zinc finger protein 554	0,5165	1,4305	0,0000	0,0007
ENSG00000142556	ZNF614	zinc finger protein 614	-0,4581	0,7279	0,0000	0,0001
ENSG00000198093	ZNF649	zinc finger protein 649	-0,3926	0,7618	0,0004	0,0042
ENSG00000277462	ZNF670	zinc finger protein 670	-0,4642	0,7249	0,0007	0,0066
ENSG00000230844	ZNF674-AS1	ZNF674 antisense RNA 1 (head to head)	-0,7297	0,6030	0,0000	0,0000

ENSG00000229809	ZNF688	zinc finger protein 688	0,5718	1,4864	0,0001	0,0015
ENSG00000143067	ZNF697	zinc finger protein 697	-0,5418	0,6869	0,0000	0,0000
ENSG00000183779	ZNF703	zinc finger protein 703	0,4551	1,3709	0,0000	0,0000
ENSG00000147180	ZNF711	zinc finger protein 711	-0,7743	0,5847	0,0001	0,0011
ENSG00000178665	ZNF713	zinc finger protein 713	0,9307	1,9062	0,0000	0,0000
ENSG00000162086	ZNF75A	zinc finger protein 75a	-0,4450	0,7346	0,0003	0,0038
ENSG00000169957	ZNF768	zinc finger protein 768	0,3851	1,3059	0,0001	0,0016
ENSG00000196456	ZNF775	zinc finger protein 775	0,8823	1,8434	0,0000	0,0001
ENSG00000170100	ZNF778	zinc finger protein 778	-0,4160	0,7495	0,0003	0,0034
ENSG00000128000	ZNF780B	zinc finger protein 780B	-0,4051	0,7552	0,0001	0,0010
ENSG00000180884	ZNF792	zinc finger protein 792	-0,6142	0,6533	0,0000	0,0000
ENSG00000151612	ZNF827	zinc finger protein 827	-0,5340	0,6907	0,0000	0,0000
ENSG00000124203	ZNF831	zinc finger protein 831	2,8018	6,9731	0,0000	0,0000
ENSG00000196605	ZNF846	zinc finger protein 846	0,6047	1,5206	0,0003	0,0036
ENSG00000236609	ZNF853	zinc finger protein 853	-0,8119	0,5696	0,0000	0,0000
ENSG00000106479	ZNF862	zinc finger protein 862	0,5758	1,4905	0,0000	0,0000
ENSG00000117174	ZNHIT6	zinc finger, HIT-type containing 6	-0,4673	0,7233	0,0000	0,0002
ENSG00000149506	ZP1	zona pellucida glycoprotein 1 (sperm receptor)	1,3227	2,5013	0,0000	0,0000
ENSG00000188372	ZP3	zona pellucida glycoprotein 3 (sperm receptor)	0,5107	1,4248	0,0001	0,0007
ENSG00000132485	ZRANB2	zinc finger, RAN-binding domain containing 2	-0,4070	0,7542	0,0000	0,0006
ENSG00000086827	ZW10	zw10 kinetochore protein	-0,4146	0,7502	0,0001	0,0009
ENSG00000174442	ZWILCH	zwilch kinetochore protein	-0,4541	0,7300	0,0000	0,0000
